# Transcriptomics-based analysis of muscle injury mechanism mediated by inflammatory imbalance in the Duchenne muscular dystrophy muscle microenvironment

**DOI:** 10.3389/fneur.2026.1905030

**Published:** 2026-09-15

**Authors:** Chunhui Shan, Yu Li, Nan Li, Zhe Zhao, Yinhong Chen, Qi Bing

**Affiliations:** 1 Department of Medical Imaging, Hebei General Hospital, Shijiazhuang, Hebei, China; 2 Department 1 of Cardiovascular Medicine, Hebei General Hospital, Shijiazhuang, Hebei, China; 3 Department of Neurology, Hebei Medical University Third Hospital, Shijiazhuang, Hebei, China

**Keywords:** biomarker, Duchenne muscular dystrophy, inflammatory imbalance, PTPRC, transcriptomics

## Abstract

**Background:**

Duchenne muscular dystrophy (DMD) is a hereditary neuromuscular disease characterized by progressive myofiber injury, chronic inflammatory response, and persistent fibrotic remodeling, for which no universal clinical treatment is currently available. The inflammatory-fibrotic microenvironment is regarded as a critical driver of DMD progression; nevertheless, the key molecules governing extracellular matrix (ECM) remodeling and immune imbalance remain incompletely elucidated.

**Methods:**

Two transcriptomic datasets were integrated to construct an expression matrix containing DMD patients and healthy controls. Extracellular matrix (ECM) activation scores were calculated based on three representative ECM gene sets. Differential analysis combined with weighted gene co-expression network analysis (WGCNA) was performed to identify core modules related to DMD and ECM activation. A protein–protein interaction (PPI) network was built for the intersecting genes, and hub genes were screened by integrating five network topology algorithms. Immune cell infiltration characteristics were analyzed in protein tyrosine phosphatase receptor type C (PTPRC) high- and low-expression groups, followed by gene set enrichment analysis (GSEA). A PTPRC knockdown cell model was established, and quantitative real-time PCR (qRT-PCR), Western blotting (WB), and immunofluorescence (IF) were applied to verify the effect of PTPRC on C2C12 myoblast differentiation.

**Results:**

ECM activation scores were elevated in the DMD group and showed favorable diagnostic efficacy. After stratification by ECM score, the ECM-High group exhibited activation of fibrosis/epithelial–mesenchymal transition (EMT) and immune/inflammatory pathways, accompanied by metabolic dysfunction and aberrant activation of myogenesis-related programs. WGCNA further identified the MEturquoise module, which was positively correlated with both DMD status and ECM score. Intersection of this module with DMD and ECM differential genes yielded PTPRC. Immune infiltration analysis showed increased proportions of M2 macrophages and regulatory T cells (Tregs) in the PTPRC_High group. GSEA confirmed significant activation of 12 inflammatory pathways. *In vitro* experiments demonstrated that PTPRC knockdown markedly promoted myogenic differentiation and alleviated fibrotic phenotypes in myoblasts.

**Conclusion:**

PTPRC serves as a candidate biomarker for DMD. Its high expression participates in DMD-associated fibrosis, likely linked to an immunosuppressive microenvironment.

## 1 Introduction

Muscular dystrophy comprises a group of hereditary neuromuscular disorders that compromise the structure and function of skeletal muscle, characterized by muscle degeneration, atrophy, and functional decline (1). Among these myopathies, Duchenne muscular dystrophy (DMD) is a severe X-linked recessive disorder with a global incidence of approximately 1 in 3,300 male live births, manifesting as progressive muscle weakness and loss of motor function (2, 3). In most cases, DMD arises from mutations in the gene encoding dystrophin, a cytoskeletal protein that links the intracellular actin cytoskeleton to the dystrophin-associated glycoprotein complex and contributes to maintaining the integrity of the myofiber sarcolemma (4). These mutations result in reduced or absent dystrophin expression (5). The pathological progression of DMD is also accompanied by persistent inflammation (6) and extracellular matrix (ECM) deposition (7), which accelerate muscle injury and fibrosis while impairing regenerative capacity. Although corticosteroids such as prednisolone and deflazacort are used for palliative care (8), their long-term efficacy and safety remain unclear, and curative therapies are still lacking. There is thus an urgent need to develop novel biomarkers and therapeutic targets to improve DMD diagnosis and management.

Inflammation is a host defense mechanism triggered by injury or other stimuli, aimed at eliminating endogenous or exogenous damage (9). However, sustained or dysregulated inflammation leads to chronic inflammatory states and subsequent tissue fibrosis (10, 11). In DMD, ongoing muscle injury elicits inflammatory responses marked by increased inflammatory cell infiltration, elevated pro-inflammatory factors, and accelerated muscle fibrosis (12, 13). Immune cells play indispensable roles in this process: M1 macrophages dominate inflammatory responses in early disease, whereas M2 macrophages accumulate progressively with disease progression and promote fibroblast proliferation and collagen deposition via secretion of mediators such as TGF-β (14, 15). While these observations link immune cells to the inflammatory microenvironment in DMD, the key molecules governing inflammatory regulation remain incompletely defined.

Transcriptomics has become instrumental in identifying key genes across diverse diseases, relying mainly on microarray and RNA-Seq platforms (16). Advances in microarray and high-throughput RNA sequencing have further established transcriptomics as a powerful tool for dissecting the molecular mechanisms and immune microenvironments of complex disorders (17). In DMD research, transcriptomic analyses provide robust technical support for systematically characterizing pathological remodeling in the muscle microenvironment (18).

This work delineates the correlations between ECM activation, inflammatory pathway signaling, and myogenesis, and screens for core genes associated with DMD. Immune infiltration analysis is applied to reveal immune cell composition differences across subgroups. Cell-based assays further explore the effects of the core gene on myoblast differentiation and fibrosis. These findings may provide a candidate biomarker for DMD-related fibrotic progression.

## 2 Methods

### 2.1 Data sources

Two public gene expression datasets were integrated: GSE38417 (microarray platform) and GSE109178 (RNA-seq platform). GSE38417 included 22 samples, and GSE109178 included 49 samples, covering DMD patients and healthy controls. Other muscular dystrophy subtypes in GSE109178 were grouped into the DMD cohort for analysis. Common genes from both datasets were extracted, and expression matrices were merged column-wise (Supplementary Table S1). Owing to pronounced batch effects from different platforms, the ComBat algorithm in the R package “sva” (19) was applied for batch correction. ComBat uses a parametric empirical Bayesian framework to correct batch effects while preserving genuine biological variation by incorporating a covariate matrix for biological grouping (DMD vs. Control) to avoid over-correction. Principal component analysis (PCA) was performed on the merged expression matrix before and after batch correction to evaluate batch removal and retention of biological signals.

Furthermore, to enhance the robustness and generalizability of our conclusions, the GSE6011 (microarray platform) dataset was additionally downloaded as an external validation set, which comprised a total of 36 samples.

### 2.2 ECM gene set acquisition

ECM-related gene sets were retrieved from the MSigDB database for *Homo sapiens*. Three representative ECM-related pathway gene sets were selected: KEGG_ECM_RECEPTOR_INTERACTION, KEGG_FOCAL_ADHESION, and REACTOME_COLLAGEN_FORMATION. Genes from these sets were combined and deduplicated to construct a comprehensive ECM signature gene set (ECM_signature) (Supplementary Table S2) for subsequent enrichment scoring.

### 2.3 Single-sample gene set enrichment analysis (ssGSEA)

Based on the batch-corrected merged expression matrix, the ssgseaParam function in the Bioconductor package “GSVA” (20) was used to calculate ssGSEA enrichment scores for the ECM signature gene set in each sample. ssGSEA quantifies the overall activation level of a given gene set in a single sample by integrating the cumulative distribution function of gene expression ranks. The normalize parameter was set to TRUE, which normalized the original ssGSEA score based on the difference between the maximum and minimum values within the gene set. All ssGSEA scores were further Min–Max normalized (linearly scaled to the interval [0, 1]) to eliminate unit differences across samples, yielding the final ECM activation score (ECM score). The ECM score was compared between DMD patients (*n* = 59) and healthy controls (*n* = 12) using the two-sided Wilcoxon rank-sum test and visualized as a boxplot overlaid with a scatter plot.

### 2.4 ROC curve analysis

To evaluate the diagnostic performance of the ECM score for DMD, the R package “pROC” (21) was applied to generate receiver operating characteristic (ROC) curves. The area under the curve (AUC) and its 95% confidence interval (95% CI) were estimated to assess the ability to distinguish DMD from control samples.

### 2.5 Differential expression analysis

Differential expression analysis between DMD patients and healthy controls was performed using the Bioconductor package “limma” (22). The matrix included biological grouping (DMD vs. Control, with Control as reference) and dataset origin (GSE38417 vs. GSE109178) as covariates to control residual batch effects. Linear models were fitted with lmFit, empirical Bayes moderation was applied with eBayes (trend = TRUE, robust = TRUE), and results were extracted with topTable and adjusted for multiple testing using the Benjamini–Hochberg (BH) method. Significantly differentially expressed genes (DEGs) were defined with an adjusted *p*-value (adj.P.Val) ≤ 0.05 and |log₂FC| ≥ 1.

Samples were divided into ECM-High (score above median) and ECM-Low (score ≤ median) groups based on the median ECM activation score. Differential expression analysis between ECM subgroups (ECM-Low as reference) was conducted using limma with the same thresholds (adj.P.Val ≤ 0.05, |logFC| ≥ 1) to generate a ranked gene list for GSEA.

### 2.6 GSEA

GSEA was performed for the MSigDB Hallmark gene set (50 gene sets, obtained via msigdbr) using the R package “clusterProfiler” (23) (1,000 permutations, BH correction, FDR < 0.05, gene set size 15–500). Significant pathways were manually annotated into three biological categories: Immune/Inflammation, Fibrosis/EMT, and Myogenesis/Metabolism, and visualized as bar plots.

### 2.7 WGCNA and key gene identification

Weighted gene co-expression network analysis was conducted using the R package “WGCNA” (24). Genes with > 10% missing values or zero variance were removed, and the top 5,000 most variable genes were selected for network construction. Sample quality was assessed with the goodSamplesGenes function to exclude outliers. The pickSoftThreshold function (networkType = “signed”) was used to evaluate scale-free topology fitting (R^2^) across powers 1–20; the power yielding R^2^ > 0.85 was chosen (soft threshold = 4, R^2^ = 0.892). A co-expression network was constructed with the blockwiseModules function (networkType = “signed,” TOMType = “signed,” minModuleSize = 30, mergeCutHeight = 0.25). Hierarchical clustering and dynamic tree cutting based on the topological overlap matrix (TOM) identified modules, which were merged if highly similar (correlation > 0.75). Modules significantly correlated with DMD status and the ECM score were identified. Candidate ECM hub genes were obtained by intersecting module genes with ECM differential genes.

### 2.8 PPI network construction via STRING

PPI data for candidate genes were retrieved via the STRING database REST API (v12.0, combined score ≥ 0.4, medium confidence), and the PPI network was built using the R package “igraph” (25). Only the largest connected component (LCC) of the network was retained for downstream analysis to avoid isolated nodes affecting centrality calculations.

Five network topology algorithms in the cytoHubba plugin were used to score hub genes: Degree (number of direct interactions); Betweenness Centrality (normalized ratio of shortest paths passing through the node); Closeness Centrality (normalized reciprocal of average shortest path length); Maximal Clique Centrality (MCC; log-scale scores calculated with lgamma to avoid factorial overflow); Density of Maximum Neighborhood Component (DMNC; edges in the largest connected component of the neighbor subgraph divided by node count^1.5). Genes were ranked by each algorithm, and a composite rank score (sum of five ranks) was computed. Genes appearing in the Top 10 of ≥ 4 algorithms were defined as core hub genes.

### 2.9 Immune infiltration analysis stratified by PTPRC expression

Samples were divided into PTPRC-High (≥ median) and PTPRC-Low (< median) groups based on the median PTPRC expression in the batch-corrected expression matrix. The CIBERSORT algorithm with the LM22 signature matrix (22 immune cell types) was used to estimate immune cell composition, with 100 permutations and quantile normalization (QN = TRUE). The two-sided Wilcoxon rank-sum test was used to compare cell proportions between PTPRC-High and PTPRC-Low groups for all 22 immune cell types, with BH correction for multiple testing; adj.P.Val < 0.05 indicated significance. Boxplots were generated for significantly different cell types.

### 2.10 Differential expression and pathway enrichment in PTPRC subgroups

Differential expression analysis between PTPRC-High and PTPRC-Low subgroups (Low as reference) was performed with the “limma” package (adj.P.Val ≤ 0.05, |log₂FC| ≥ 1). KEGG pathway over-representation analysis (ORA) was conducted for up- and down-regulated DEGs separately using “clusterProfiler,” with significance defined as BH-adjusted P and q ≤ 0.05. Gene symbols were converted to Entrez IDs via org. Hs.eg.db, using all detected genes as background.

GSEA was performed for the combined MSigDB Hallmark (50 gene sets) and KEGG_LEGACY (186 gene sets) using clusterProfiler. The gseaplot2 function in the “enrichplot” package (26) was used to plot enrichment curves for key inflammation-related pathways, and bubble charts summarized significantly activated and suppressed pathways.

### 2.11 snRNA-seq sequencing data analysis

The single-cell transcriptomic data used in this study were obtained from the public dataset GSE288958 in the GEO database^1^, which contains single-nucleus RNA-seq (snRNA-seq) data of skeletal muscle from patients with DMD. This dataset comprised a total of 8 samples, including 5 healthy control (Normal) subjects and 3 DMD patients. Quality control (QC), normalization, and integration of single-cell data were performed using the R package Seurat (v5.5.1). The detailed analytical pipeline was as follows. First, quality-control filtering was applied to the expression matrix of each sample to remove low-quality nuclei and aberrantly expressed genes, with filtering criteria set as: *nFeature_RNA* > 200; *nFeature_RNA* < 5,000; *percent.mt* < 5. Subsequently, the expression matrix was normalized via the LogNormalize method, highly variable features were identified, and principal-component analysis (PCA) was implemented for linear dimensionality reduction. Given that data were derived from multiple samples, the Harmony algorithm was used for batch-effect correction to eliminate technical variation across samples while preserving genuine biological differences. On the basis of the corrected embedding space, Uniform Manifold Approximation and Projection (UMAP) was adopted for nonlinear dimensionality-reduction visualization, and unsupervised cell clustering was conducted using graph-based clustering. Fifteen cell clusters were finally obtained at a resolution of 0.3. To identify marker genes for each cell cluster, differential expression analysis was carried out with the FindAllMarkers function embedded in the Seurat package. The screening thresholds were configured as: only.pos = TRUE, min.pct = 0.25, and logfc.threshold = 0.25. The resulting differentially expressed genes (DEGs) served as supporting evidence for cell-type annotation. Marker genes for cell-type annotation were retrieved from two sources: (1) published snRNA-seq studies on DMD/skeletal muscle, including work by Suárez-Calvet et al. (27), Scripture-Adams et al. (28), and Chemello et al. (29); (2) the CellMarker 2.0 database^2^. A manual annotation strategy based on dual validation of marker-gene expression patterns and DEGs was utilized.

To characterize the expression profile of PTPRC in the DMD muscle microenvironment, log-normalized expression values (data layer of the RNA assay) of PTPRC were extracted from the annotated Seurat object for visualization and statistical analysis at two levels: (1) group-wise comparison and (2) cell-type-by-group comparison. The Wilcoxon rank-sum test was applied for between-group comparison, followed by Bonferroni correction for multiple testing.

Non-parametric Wilcoxon rank-sum test, the standard approach for single-cell data, was used for all statistical tests, and the Bonferroni method was adopted for multiple-comparison correction to rigorously control false-positive rates. Significance was annotated as follows: * for *p* < 0.05, ** for *p* < 0.01, *** for *p* < 0.001, and **** for *p* < 0.0001.

### 2.12 Cell cultivation and treatment

Mouse C2C12 myoblasts (SCSP-505) were purchased from the Cell Bank of the Chinese Academy of Sciences. Cells were cultured in Dulbecco’s Modified Eagle’s Medium (DMEM) (12,491,015, Gibco, USA) supplemented with 10% fetal bovine serum (FBS) (A5669701, Gibco, USA) and 1% penicillin–streptomycin (P/S) (15,140,148, Gibco, USA) in a 37 °C incubator with 5% CO₂. Cells were seeded in 6-well plates at 1 × 10^5^ cells/well and incubated for 24 h before transfection. At 80–90% confluence, the medium was switched from 10% FBS to 2% horse serum (26,050,088, Gibco, USA) for 3 days to induce myogenic differentiation. Transfected cells were treated with 10 ng/mL TGF-β1 (100-21C, Peprotech, UK) for 48 h to establish an *in vitro* DMD inflammatory and fibrotic model (30).

Previous studies have demonstrated that TGF-β1 stimulation can rapidly trigger C-terminal phosphorylation and activation of SMAD2/3, and this phosphorylation event generally occurs within an early time window post-stimulation (30, 31). Short-term TGF-β1 stimulation was applied in this study to assess the early activation status of the TGF-β/SMAD signaling pathway. Referring to experimental conditions reported in prior studies for SMAD phosphorylation induction by TGF-β1 (10 ng/mL), whole-cell protein lysates were harvested 1 h after TGF-β1 treatment to detect the levels of p-SMAD2 (Ser465/467) and total SMAD2, so as to evaluate the activation of the TGF-β/SMAD pathway.

Meanwhile, to investigate the effect of PTPRC knockdown on TGF-β1-induced myogenic injury and fibrotic phenotypes, cells were treated with TGF-β1 (10 ng/mL) for 48 h, followed by detection of expression alterations in myogenic markers Myh6 and MyoG, as well as fibrosis-associated proteins COL1A1, MMP9 and CTGF.

### 2.13 Cell transfection

To investigate the role of PTPRC in C2C12 myoblast differentiation, cells were transfected with PTPRC-targeting siRNA (si-NC, si-PTPRC) using Lipofectamine 3,000 (L3000015, Gibco, USA). Before transfection, cells were seeded in 6-well plates and grown to 70–80% confluence. siRNA and Lipo3000 were diluted separately in 125 μL Opti-MEM per tube. Diluted siRNA was transferred to Lipo3000-containing tubes and incubated for 15 min at room temperature. The siRNA–Lipo3000 complex was added to each well and incubated for 12 h. Transfected cells were used for subsequent experiments, in which si-PTPRC denotes PTPRC-targeting small-interfering RNA, and si-NC represents negative-control siRNA.

### 2.14 qRT-PCR

Total RNA was extracted from cells with Trizol reagent (R0016, Beyotime, China). RNA quality and concentration were measured by a NanoDrop2000 spectrophotometer (Thermo Fisher Scientific, USA). 1 μg of total RNA was reverse-transcribed to cDNA using the Evo M-MLV RT Reaction Mix Kit (AG11728, Accurate Biology, China). qPCR was performed on an ABI 7500 PCR system using the SYBR Green Premix Pro Taq HS qPCR Kit (AG11719, Accurate Biology, China). GAPDH served as the internal control. Primer sequences are listed in Table 1.

**Table 1:** Primer sequence.

Gene	Forward primer (5′-3′)	Reverse primer (5′-3′)
PTPRC (*Mus musculus*)	CTTTGCTTATGTGGCGTGTGT	TTATCCCCTTCTGATGCGCC
GAPDH (*Mus musculus*)	AAGAGGGATGCTGCCCTTAC	GTTCACACCGACCTTCACCA

### 2.15 Western blotting (WB)

Total protein was extracted with RIPA lysis buffer (89,900, Thermo Fisher Scientific, USA) containing 1 mM PMSF (ST506, Beyotime, China) and PhosSTOP (4,906,845,001, Roche, USA). Protein concentration was determined using a BCA assay kit (23,227, Thermo Fisher Scientific, USA). Equal amounts of protein were separated by 12% SDS-PAGE and transferred to PVDF membranes (Millipore, USA). Membranes were blocked with 5% BSA in TBST buffer (500 mM NaCl, 20 mM Tris–HCl, 0.2% Tween) for 1 h, then incubated overnight at 4 °C with primary antibodies against Myh6 (ab185967, Abcam, UK), MyoG (ab124800, Abcam, UK), PTPRC (ab281586, Abcam, UK), COL1A1 (ab260043, Abcam, UK), MMP9 (ab228402, Abcam, UK), and CTGF (ab6992, Abcam, UK), Phospho-SMAD2 (Ser465/467)(#3,108, CST, USA), SMAD2/3 (#3102, CST, USA). Membranes were then incubated with horseradish peroxidase (HRP)-conjugated anti-rabbit IgG antibody (7074S, CST, USA) for 1 h. Protein bands were visualized by a chemiluminescence detection system, with GAPDH (2118S, CST, USA) as the loading control.

For phosphorylated protein detection, TBST buffer containing 5% BSA was used for both blocking and primary-antibody incubation to minimize potential interference of casein in non-fat dried milk with phosphorylated-signal detection. After electrophoresis and membrane transfer of cellular proteins, the level of phosphorylated SMAD2 (Ser465/467) was first determined. Thereafter, stripping buffer (ST204, Beyotime, China) was applied to remove primary and secondary antibody signals, and the membrane was re-incubated for detection of the corresponding total SMAD2 protein. WB results were used to evaluate the activation status of the TGF-β/SMAD signaling pathway.

### 2.16 Immunofluorescence (IF)

Cells were rinsed twice with phosphate-buffered saline (PBS), immobilized with 4% paraformaldehyde (P0099, Beyotime, China) at room temperature for 20 min, blocked with QuickBlock™ Immunostaining Blocking Buffer (P0260, Beyotime, China) for 30 min, and incubated overnight at 4 °C with MHC antibody (PA5-115216, Thermo Fisher Scientific, USA). Cells were washed three times with PBS and incubated with Alexa Fluor 488-conjugated anti-rabbit antibody (4412S, CST, USA) for 45 min. After three additional PBS washes, nuclei were stained with DAPI (F6057, Sigma, USA). Images were acquired and analyzed under a fluorescence microscope (Nikon, Japan). For quantitative analysis, multinucleated myotubes were defined as MHC-positive cells containing three or more nuclei. The fusion index was calculated as the percentage of nuclei within myotubes with three or more nuclei relative to the total number of nuclei (32).

### 2.17 Statistical analysis

Student’s *t*-test was applied in the difference assessment between two groups in cell-based experiments. ANOVA was applied for comparisons involving more than two groups (*p* < 0.05: statistically significant). Statistical analyses were processed by GraphPad Prism (version 8.0, GraphPad, USA). Data were presented as mean ± standard deviation.

In bioinformatics analyses, two-tailed Wilcoxon rank-sum test (Mann–Whitney U test) was used for comparisons of continuous variables between two groups, including comparisons of ECM activation score, PTPRC expression level and M2 macrophage score between DMD and control groups, as well as between PTPRC-High and PTPRC-Low subgroups. For diagnostic performance evaluation, the pROC package was utilized to calculate the area under the ROC curve (AUC) together with 95% confidence intervals. The Benjamini-Hochberg method was adopted for multiple-testing correction. *p* < 0.05 was considered statistically significant. All the above-mentioned analyses were performed using R software (version 4.3.0).

## 3 Results

### 3.1 Cross-platform data integration and batch correction using the ComBat algorithm

This work integrated the GSE38417 (microarray, *n* = 22) and GSE109178 (RNA-seq, *n* = 49) datasets, including samples from DMD patients and healthy controls. PCA was performed to assess batch effects. Before correction, samples from the two datasets were completely separated by batch (Figure 1A). When colored by biological group, DMD and Control samples were poorly distinguished (Figure 1B). After ComBat correction, samples from both datasets were well mixed in PCA space (Figure 1C). Meanwhile, PCA by biological group showed clear separation between DMD and Control samples after correction, confirming that ComBat eliminated batch effects without over-correction (Figure 1D). These results validated successful batch correction, supporting the use of the integrated dataset for downstream analyses.

**Figure 1:**
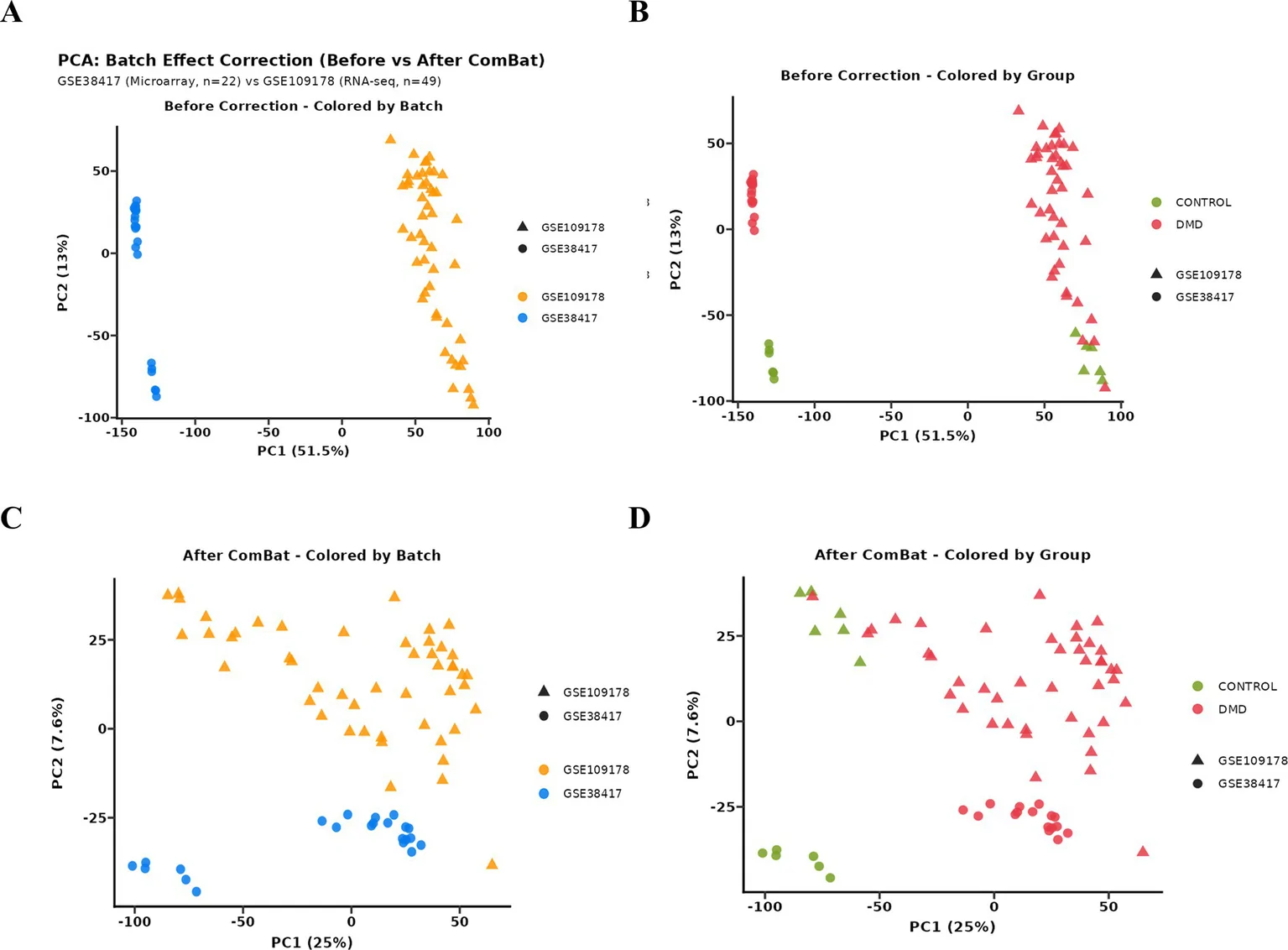
Multi-platform transcriptomic data integration and batch effect correction. **(A,B)** PCA plots before correction, colored by batch (left) and biological group (right). **(C,D)** PCA plots after ComBat correction, colored by batch (left) and biological group (right).

### 3.2 Aberrant activation of inflammatory/fibrotic pathways in the DMD pathological microenvironment

Excessive and disorganized ECM deposition is a key pathological feature of DMD (7). The ssGSEA algorithm was applied to calculate ECM activation scores for each sample. ECM scores were significantly higher in DMD patients (*n* = 59) than in healthy controls (*n* = 12) (*p* < 0.001) (Figure 2A; Supplementary Table S3), indicating widespread ECM activation in DMD muscle tissue. ROC curve analysis showed that the composite ECM score achieved an AUC of 0.956 (95% CI: 0.908–1.000) for DMD diagnosis (Figure 2B), supporting its robust biological diagnostic value. To further validate the stability of ECM-activation signatures, we performed validation in the independent external validation cohort GSE6011. The results demonstrated that the ECM activation score was also significantly elevated in DMD patients within the GSE6011 cohort (*p* = 6.13 × 10^−5^) (Supplementary Figure S1A). ROC analysis revealed that the composite ECM score exhibited favorable discriminatory power (AUC = 0.903, 95% CI: 0.780–1.000) (Supplementary Figure S1B). Samples were split into ECM-High and ECM-Low groups by median ECM score, and GSEA was performed on DEGs between subgroups. In the ECM-High group, fibrosis/epithelial–mesenchymal transition (EMT)-related pathways were markedly activated, including EMT (NES = 3.30), angiogenesis (NES = 2.28), coagulation (NES = 2.18), and TGF-β signaling (NES = 1.86). Immune/inflammatory pathways were also significantly upregulated, including interferon-γ response (NES = 2.96), interferon-α response (NES = 2.85), allograft rejection (NES = 2.51), complement (NES = 2.50), and TNF-α/NF-κB signaling (NES = 2.20). In contrast, the ECM-High group displayed prominent features of metabolic reprogramming. Metabolism-related pathways including oxidative phosphorylation (NES = −2.88), Myc targets V2 (NES = −2.02), and fatty-acid metabolism (NES = −1.38) were significantly negatively enriched, indicating that high ECM-activation status is accompanied by impaired muscular energy metabolism. Nevertheless, the Myogenesis gene set was positively enriched in the ECM-High group (NES = 1.53), suggesting that ECM remodeling may be accompanied by injury-triggered regenerative myogenic responses under the pathological context of DMD (Figure 2C). Differential expression analysis between DMD patients and controls (adj.P.Val ≤ 0.05, |logFC| ≥ 0.5) identified 6,087 DEGs (Supplementary Table S4), including 3,030 upregulated genes (red) such as COL3A1, MYL5, HOXB3, and RIPK2 (consistent with activated collagen synthesis, muscle structural remodeling, and inflammatory signaling) and 3,057 downregulated genes (blue) such as MYH14 and PNPLA6 (consistent with suppressed muscle contractile function) (Figure 2D). Collectively, these data indicate that the inflammatory-fibrotic axis is activated within the DMD microenvironment, accompanied by metabolic dysfunction and compensatory regenerative responses.

**Figure 2:**
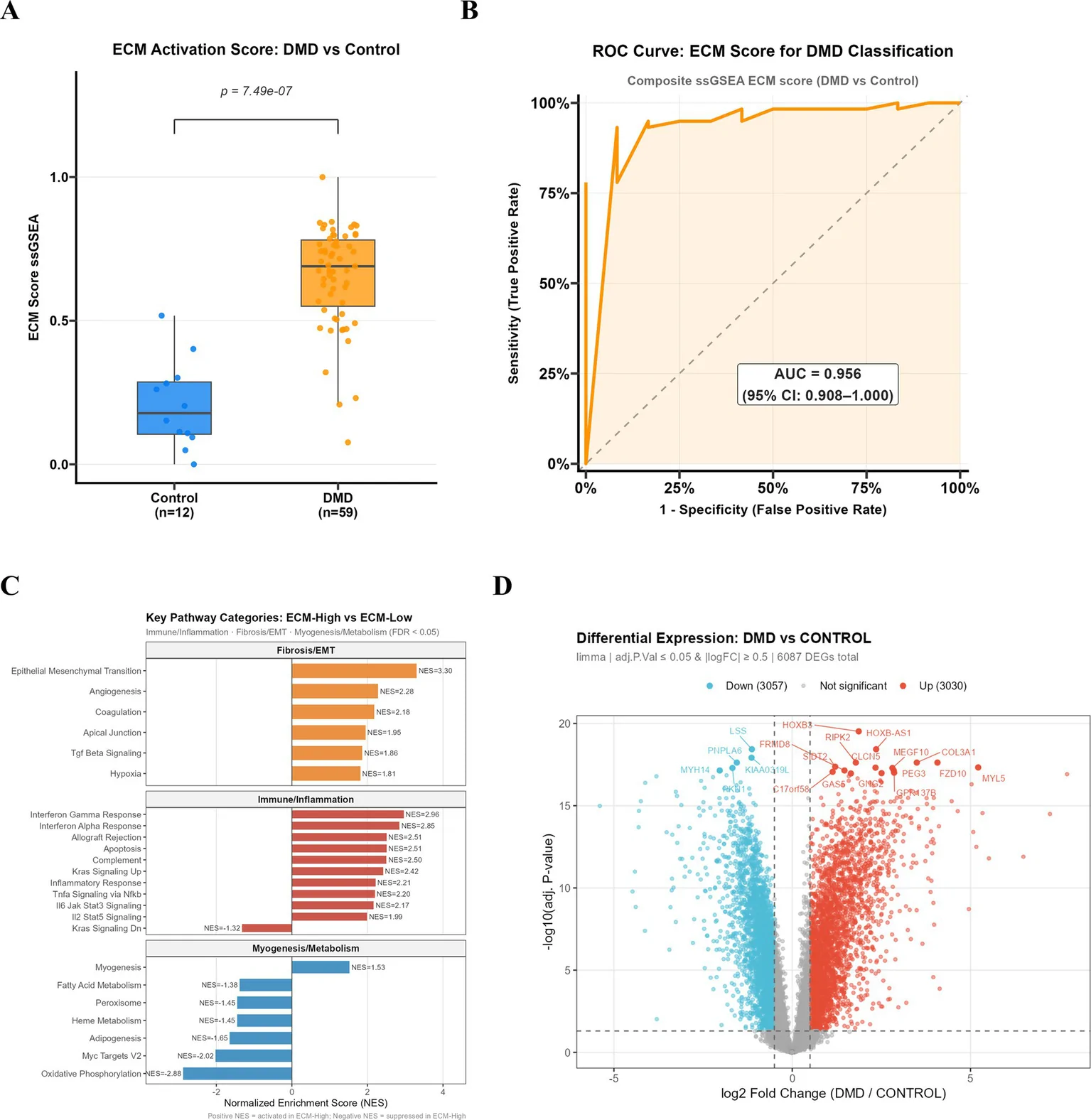
ECM activation score, diagnostic value, and pathway enrichment in ECM high vs low groups. **(A)** Boxplot of composite ssGSEA ECM scores comparing DMD patients (*n* = 59, yellow) and healthy controls (*n* = 12, blue). **(B)** ROC curve of the ECM score for DMD classification. **(C)** GSEA of MSigDB Hallmark gene sets in ECM-High vs. ECM-Low groups, stratified by ECM score. **(D)** Volcano plot of DEGs between DMD and control groups.

### 3.3 Identification of core co-expression modules and candidate genes in DMD progression

To identify gene modules co-regulated with DMD progression and ECM activation, WGCNA was performed on the top 5,000 most variable genes with a soft threshold β = 4 and scale-free topology fit R^2^ > 0.85 (Figure 3A). Dynamic tree cutting identified five gene co-expression modules (Figure 3B). Module–trait correlation analysis revealed that the MEturquoise module was strongly positively linked with both DMD status (*r* = 0.78, *p* = 7e-16) and ECM score (*r* = 0.93, *p* = 2e-31), suggesting a central driver role in DMD fibrosis (Figure 3C). Intersection of DMD DEGs, ECM subgroup DEGs, and MEturquoise module genes yielded 746 candidate hub genes (Figure 3D; Supplementary Table S5) for subsequent analysis.

**Figure 3:**
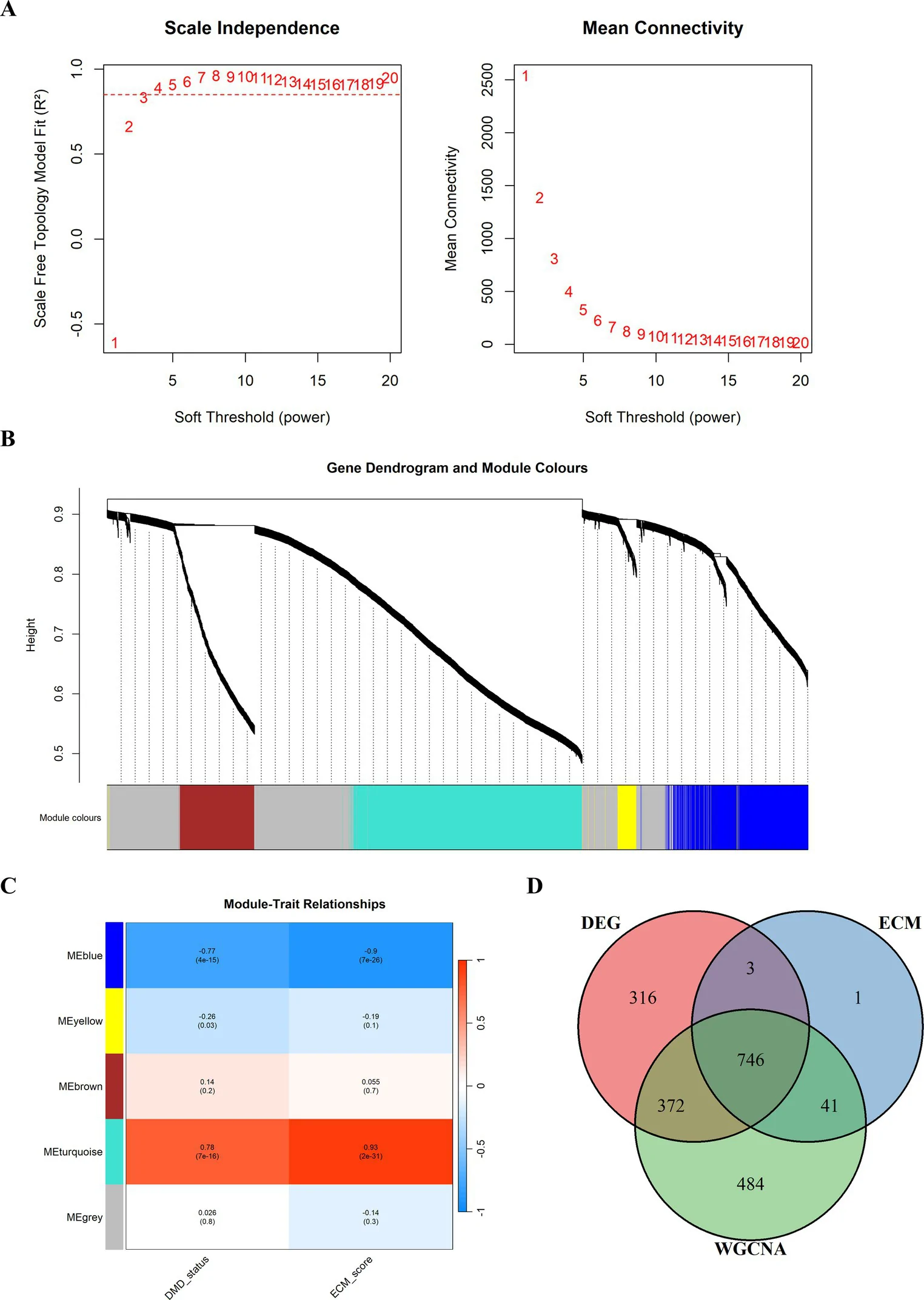
WGCNA and Multi-Dimensional Intersection Screening for candidate genes. **(A)** Soft threshold (β) selection for WGCNA. Left: Scale-free topology fit (*R*^2^) with soft threshold variation. Right: Mean connectivity with soft threshold variation. **(B)** Gene dendrogram and module color assignment; five co-expression modules identified by dynamic tree cutting. **(C)** Module–trait correlation heatmap. **(D)** Venn diagram of DEGs, ECM differential genes, and WGCNA MEturquoise module genes.

### 3.4 Identification of the core hub gene PTPRC via multi-algorithm screening

To refine core hub genes, the 746 candidate genes were scored using five network topology algorithms (Degree, MCC, Betweenness, Closeness, DMNC). Bar plots and heatmaps revealed that PTPRC was the only gene ranked in the Top 10 by four algorithms (Degree, MCC, Betweenness, Closeness) (Figures 4A,B). This gene also exhibited significant differential expression in the validation cohort (Supplementary Figure S1C). The PPI network constructed via STRING revealed that PTPRC formed a highly interconnected network with 30 interacting proteins, including multiple immune regulators such as CD4, ITGAM, CSF1R, ITGB2, CCL2, CD8A, COL3A1, and COL1A2 (Figure 4C), supporting a central hub role for PTPRC in the DMD muscle microenvironment.

**Figure 4:**
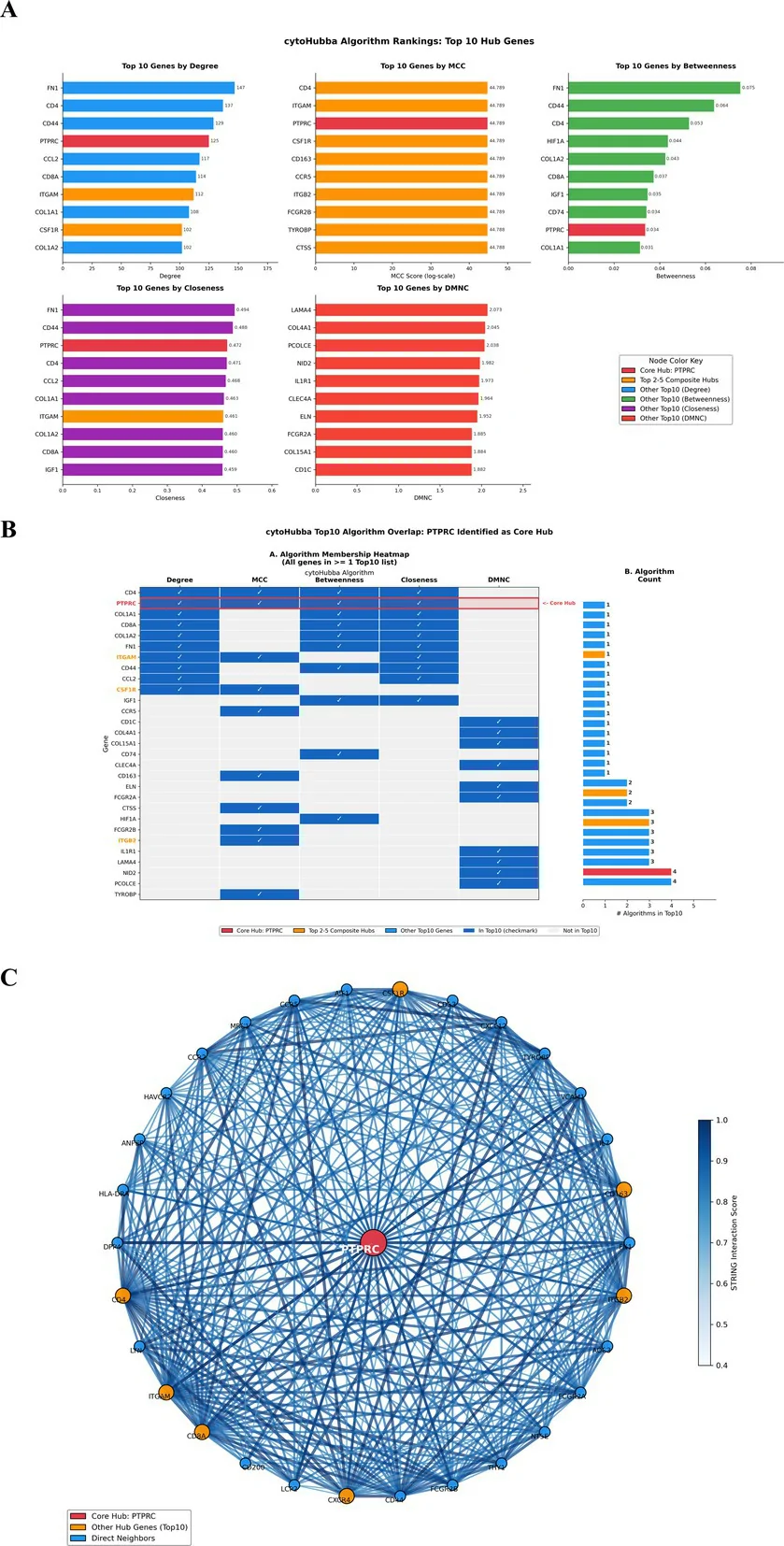
Identification of the core hub gene PTPRC. **(A)** Bar plot of Top10 hub genes ranked by five cytoHubba algorithms (Degree, MCC, Betweenness, Closeness, DMNC). **(B)** Heatmap of hub gene rankings across five topology algorithms. **(C)** PPI network of the top 30 PTPRC-interacting proteins from STRING.

To determine whether elevated PTPRC expression in DMD skeletal muscle originates from infiltrating immune cells or from myofibers and other resident cell populations, we performed single-cell transcriptomic analysis on skeletal-muscle samples from DMD patients and control subjects. Following stringent quality-control filtering (Supplementary Figures S2A–C), eight major cell clusters were identified: myofibers, satellite cells, fibroblasts/fibroblast-associated pericytes (FAPs), immune cells, endothelial cells, smooth-muscle cells, adipocytes, and mast cells. Their marker-gene expression profiles were consistent with well-established cell-type signatures (Supplementary Figures S2D,E). At the single-cell level, PTPRC expression was markedly higher in DMD than in normal controls (Supplementary Figure S2F, left panel). Cell-type-specific analysis demonstrated that PTPRC was predominantly expressed in immune cells and mast cells, with low-to-moderate expression in fibroblasts/FAPs, adipocytes, endothelial cells, and other cell populations (Supplementary Figure S2F, right panel). These findings suggest that increased bulk-tissue PTPRC expression in DMD mainly reflects immune-cell infiltration rather than intrinsic expression within myofibers, providing a cellular basis for subsequent pathway and immune-infiltration analyses.

### 3.5 Pathway enrichment analysis in PTPRC high- and low-expression groups

Building on the finding that high PTPRC expression drives an immunosuppressive microenvironment, pathway enrichment was carried out between PTPRC-High and PTPRC-Low groups to dissect downstream regulatory mechanisms. In KEGG analysis, upregulated genes were enriched in multiple immune/inflammatory pathways: phagosome, complement and coagulation cascades, cell adhesion molecule (CAM) interactions, integrin signaling, and chemokine signaling (Figure 5A). GSEA of the MSigDB Hallmark gene set further revealed significant activation (FDR < 0.05) of 12 key inflammation-related pathways in the PTPRC-High group (Figure 5B), with the highest enrichment for Interferon Gamma Response and EMT. Cell adhesion molecules, inflammatory response, and TNF-α/NF-κB signaling were also strongly activated, collectively forming an inflammatory–fibrotic signaling network driven by PTPRC. Activation of JAK–STAT, TGF-β, and chemokine signaling further suggests that high-PTPRC expression status is associated with enhanced inflammatory- and fibrosis-related signaling networks.

**Figure 5:**
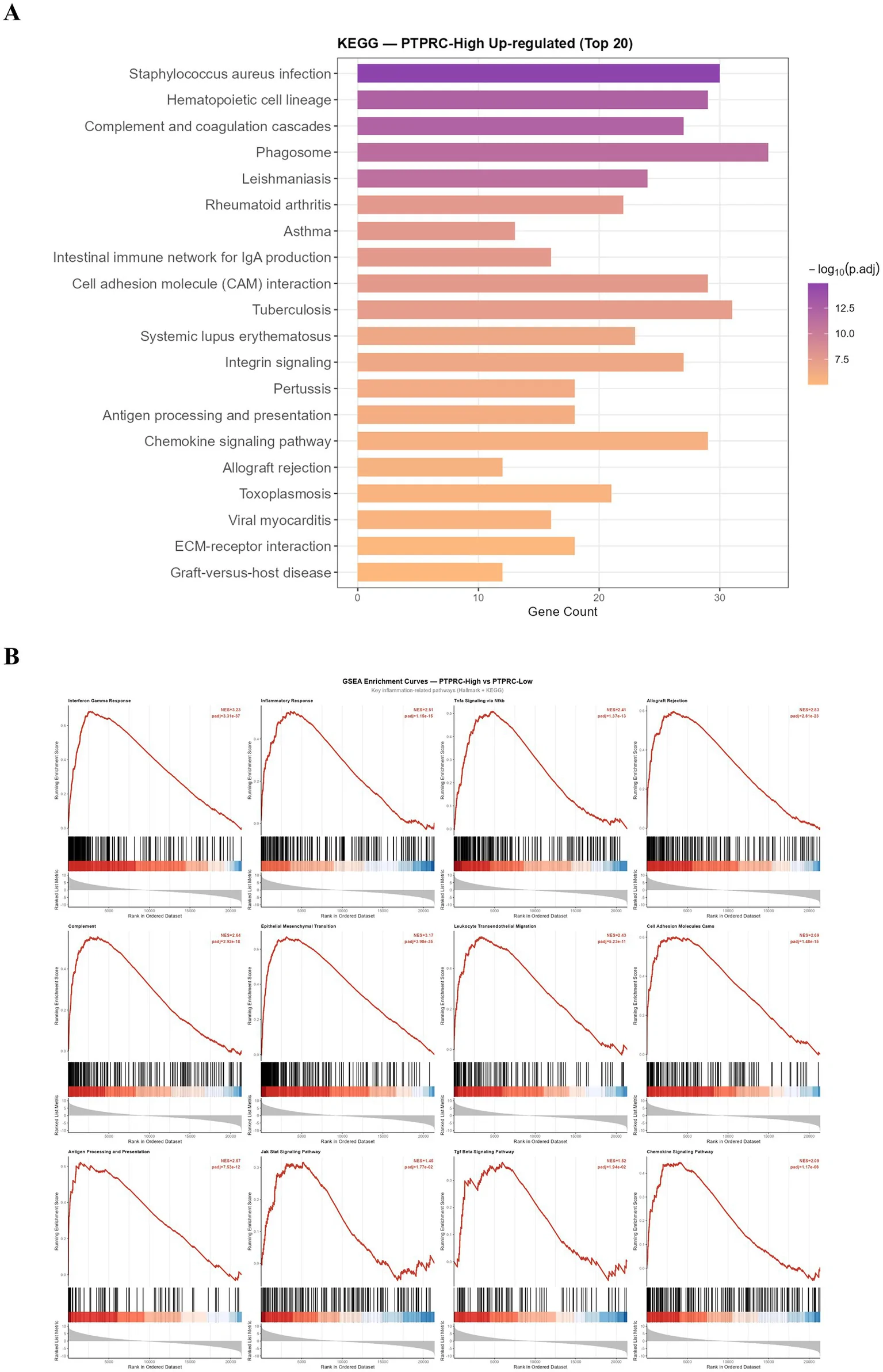
Pathway enrichment analysis in PTPRC high vs low groups. **(A)** KEGG enrichment results for upregulated genes in the PTPRC-high group. **(B)** GSEA enrichment curves for key inflammatory pathways in PTPRC-high vs. PTPRC-low groups.

### 3.6 Immune infiltration analysis in PTPRC high- and low-expression groups

Fibrosis arises from complex crosstalk between immune responses and tissue repair, with immune cells shaping local microenvironments via dynamic functional programming to modulate inflammation and fibrosis (33, 34). To explore the relationship between PTPRC expression level and the characteristics of the immune microenvironment in DMD skeletal muscle, samples were split into PTPRC-High and PTPRC-Low groups by median expression, and immune cell fractions were estimated using CIBERSORT. Stacked bar charts of immune cell composition showed a marked increase in M2 macrophages in the PTPRC-High group, linking high PTPRC expression to an immunosuppressive microenvironment (Figure 6A). Wilcoxon rank-sum testing confirmed that M2 macrophages (adj.*p* = 6.4e-07) were the most significantly increased cell type in the PTPRC-High group (Figure 6B). The above results suggest that high-PTPRC expression status is correlated with increased M2-type macrophage infiltration and features of an immunosuppressive microenvironment, which may reflect concomitant changes in immune-cell recruitment and ECM remodeling in DMD skeletal muscle.

**Figure 6:**
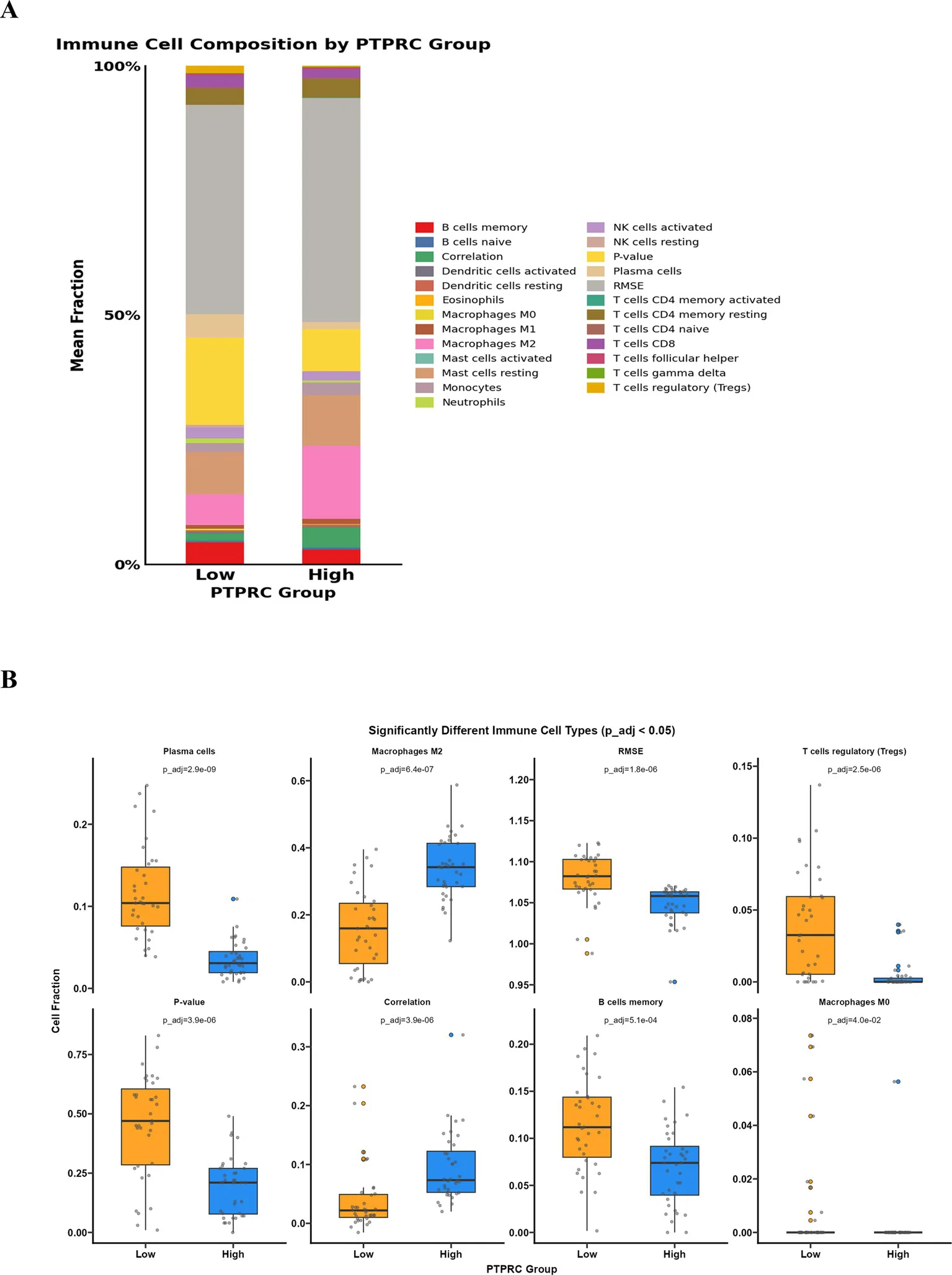
Immune cell infiltration analysis stratified by PTPRC expression. **(A)** Stacked bar chart of 22 immune cell types in PTPRC-High and PTPRC-Low groups. **(B)** Boxplots of immune cell types with significant differences between PTPRC-High and PTPRC-Low groups.

### 3.7 PTPRC knockdown promotes C2C12 myoblast differentiation and inhibits fibrosis

To validate the regulatory role of PTPRC in myogenic differentiation, dynamic PTPRC expression during C2C12 myoblast differentiation was examined. WB showed gradual upregulation of myogenic markers Myh6 and MyoG with differentiation time, confirming successful C2C12 myogenic differentiation, whereas PTPRC expression decreased progressively (Figure 7A), implying that low PTPRC supports myogenic differentiation. To mimic the DMD inflammatory and fibrotic milieu, differentiated C2C12 myotubes (3 days) were treated with 10 ng/mL TGF-β1. TGF-β1 significantly suppressed Myh6 and MyoG expression and upregulated PTPRC (Figure 7B). To define PTPRC function in this context, C2C12 myoblasts were transfected with si-NC or si-PTPRC, differentiated for 3 days, and treated with TGF-β1. qRT-PCR confirmed efficient si-PTPRC transfection and markedly reduced PTPRC mRNA expression (Figure 7C). WB showed that si-PTPRC significantly reversed TGF-β1-mediated downregulation of myogenic markers (Myh6, MyoG) and inhibited upregulation of fibrosis-related proteins (COL1A1, MMP9, CTGF) (Figures 7D,E). IF further demonstrated a significantly higher myotube fusion index in the si-PTPRC group compared with si-NC (Figure 7F). These results confirm that PTPRC knockdown effectively antagonizes TGF-β1-induced myogenic inhibition and fibrotic phenotypes, identifying PTPRC as a potential key regulator in the DMD muscle microenvironment.

**Figure 7:**
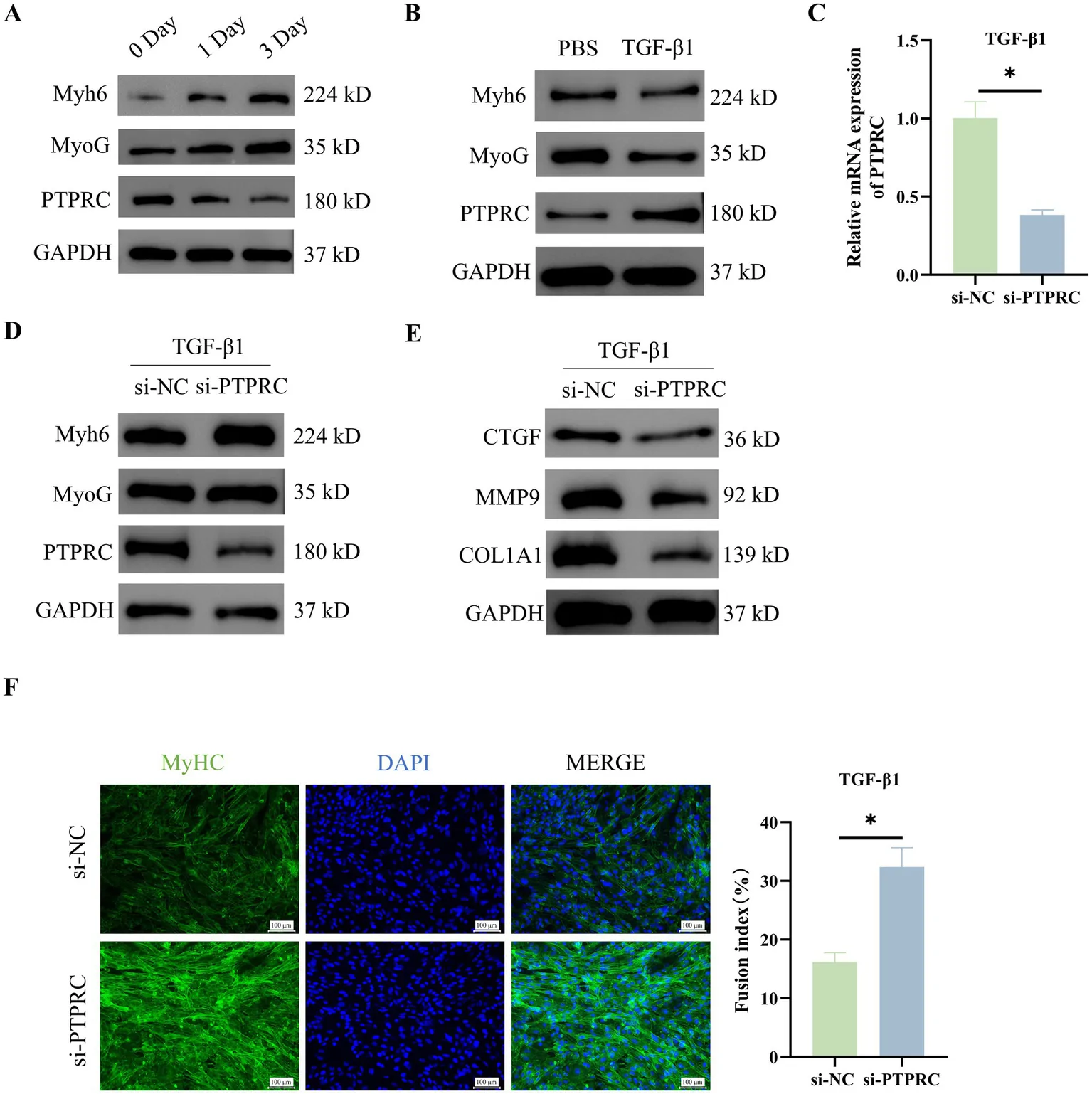
Effects of PTPRC on myogenesis and fibrosis in C2C12 cells. **(A)** WB detection of myogenic differentiation markers (Myh6, MyoG) and PTPRC expression. **(B)** WB detection of Myh6, MyoG, and PTPRC expression in C2C12 cells treated with or without TGF-β1. C2C12 cells transfected with si-NC or si-PTPRC were treated with TGF-β1; **(C)** qRT-PCR validation of PTPRC knockdown efficiency. **(D)** WB detection of Myh6, MyoG, and PTPRC expression in si-NC- or si-PTPRC-transfected C2C12 cells treated with TGF-β1. **(E)** WB detection of fibrosis markers (COL1A1, MMP9, CTGF). **(F)** IF analysis of myotube fusion index. *p < 0.05.

### 3.8 Mechanistic study on the involvement of PTPRC in TGF-β/SMAD Signaling response

The foregoing GSEA analysis (Section 3.5) revealed significant enrichment of the TGF-β signaling pathway, JAK–STAT signaling pathway, and TNF-α/NF-κB-related inflammatory pathways in the PTPRC-High group, implying that high-PTPRC expression may be associated with enhanced inflammation- and fibrosis-related signaling networks. Given that we further established an *in vitro* model of TGF-β1-induced myogenic injury and fibrotic-like phenotype in this study, the canonical TGF-β/SMAD signaling axis was selected for mechanistic validation. The TGF-β/SMAD pathway serves as a central regulatory cascade governing fibrotic remodeling in skeletal muscle, and C-terminal phosphorylation of its core effector SMAD2 reflects the activation status of TGF-β signaling (35, 36). Under si-NC- and si-PTPRC-transfected backgrounds, the protein levels of p-SMAD2 (Ser465/467) and total SMAD2 were detected following short-term TGF-β1 stimulation to evaluate the effect of PTPRC on TGF-β/SMAD signaling activity.

WB results (Figure 8) showed that p-SMAD2 (Ser465/467) protein signal was intensified in the si-NC group upon TGF-β1 stimulation, indicating that this stimulation condition effectively activated the canonical TGF-β/SMAD signaling pathway. Meanwhile, total SMAD2 protein abundance exhibited no obvious alterations across groups. Compared with the si-NC + TGF-β1 group, the si-PTPRC + TGF-β1 group displayed attenuated p-SMAD2 (Ser465/467) signal, whereas total SMAD2 protein levels remained relatively stable, suggesting that PTPRC knockdown may dampen TGF-β1-triggered SMAD2 activation. Combined with the inhibitory effect of PTPRC knockdown on the expression of fibrosis-associated molecules including CTGF, COL1A1, and MMP9 observed above, these data further support that PTPRC participates in modulating TGF-β-dependent fibrotic responses. Whether JAK–STAT- and NF-κB-related pathways are also involved in PTPRC-mediated regulatory processes remains to be further investigated.

**Figure 8:**
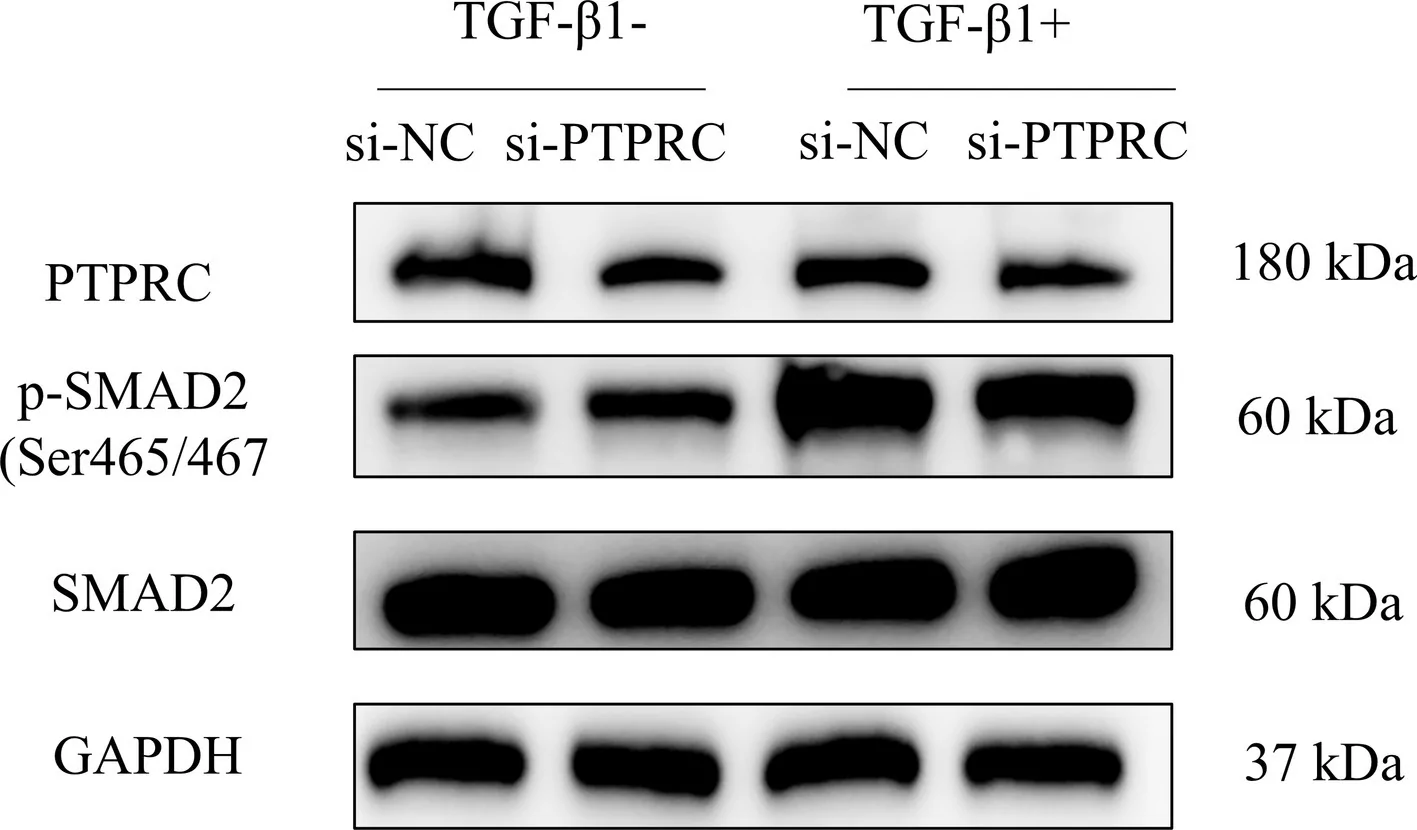
PTPRC participates in TGF-β/SMAD signaling response representative WB bands for p-SMAD2 (Ser465/467), total-SMAD2 (t-SMAD2), and PTPRC across four experimental groups (si-NC / si-PTPRC ± TGF-β1), with GAPDH as the internal reference.

## 4 Discussion

DMD is a progressive and fatal X-linked recessive myopathy (37). Current DMD therapies, including oral corticosteroids and gene therapies, remain insufficient to restore a healthy phenotype in the clinic (38, 39). This work integrated two public transcriptomic datasets and revealed that ECM-related pathways are markedly activated in muscle tissues from DMD patients, accompanied by suppression of metabolism-related pathways and activation of myogenic programs, suggesting a complex pathological state in DMD muscle wherein fibrosis, metabolic abnormalities, and compensatory regenerative responses coexist. PTPRC was identified as a core hub gene in DMD progression, and its knockdown enhanced myoblast myogenic differentiation, supporting PTPRC as a potential molecular biomarker for DMD.

PTPRC (also known as CD45) is a tyrosine phosphatase receptor widely expressed on hematopoietic cells, involved in the activation and signal transduction of T cells, B cells, and macrophages (40, 41). Dysregulated PTPRC is closely linked to multiple immune-related disorders, including rheumatoid arthritis (42), systemic lupus erythematosus (43), and dermatomyositis (43). Moreover, PTPRC has gained increasing attention for its roles in inflammatory diseases and fibrosis. In a murine model of inflammatory arthritis, PTPRC deficiency attenuated neutrophil migration to inflammatory sites (44). In models of pulmonary inflammation and fibrosis, PTPRC negatively regulates ILC2 activation to suppress airway inflammation and lung fibrosis (45). PTPRC has also been identified as a key immune-related hub gene in combined bioinformatic analyses of rheumatoid arthritis and non-small cell lung cancer, indicating broad regulatory potential in inflammation-driven pathological remodeling (46). Our multi-dimensional bioinformatic screening establishes PTPRC as a core hub gene in DMD, with abnormally high expression in DMD patient muscle, suggesting that PTPRC contributes to DMD pathogenesis by modulating local inflammatory status. Our single-cell transcriptomic analysis further validated the immune-biased expression of PTPRC. Predominant PTPRC expression in immune cells is consistent with its canonical identity as CD45, a common leukocyte antigen, providing single-cell-level evidence supporting that elevated PTPRC in DMD tissues mainly originates from immune-cell infiltration rather than altered intrinsic expression within myofibers. Notably, PTPRC-associated signals were detected in subsets of stromal cell populations, such as fibro-adipogenic progenitor cells (FAPs). Existing single-cell studies have demonstrated that FAPs are not merely structural supporting cells, but represent a critical cell population participating in extracellular matrix (ECM) remodeling and the regulation of fibrotic microenvironments during DMD pathogenesis (47). Recent single-cell and spatial transcriptomic studies have further uncovered the vital roles of immune cells, particularly specific macrophage subsets, in DMD-associated muscular fibrosis (48). Inflammation and fibrosis are two major drivers of DMD progression, forming a self-reinforcing vicious cycle that accelerates loss of muscle function. Persistent muscle injury in DMD triggers pro-inflammatory cytokine release, exacerbating muscle fiber degeneration and collagen deposition (49, 50). Fibrosis itself further amplifies local inflammation, establishing a positive feedback loop of inflammation–fibrosis that drives relentless DMD progression (51). Moreover, our present findings are highly consistent with the above-mentioned mechanisms: fibrotic/EMT pathways and immune/inflammatory pathways are synergistically activated in the ECM-High group, accompanied by prominent metabolic dysfunction characterized by the repression of oxidative phosphorylation- and lipid metabolism-related pathways. Notably, myogenic gene programs tend to be enhanced in the ECM-High group, which may reflect compensatory regenerative responses under the injurious microenvironment of DMD skeletal muscle. Nevertheless, such regenerative responses may fail to be sustainably maintained under persistent stimulation by inflammatory and fibrotic signals. *In vitro* experiments further showed that TGF-β1 upregulates PTPRC and inhibits myogenic differentiation markers, whereas PTPRC knockdown partially reverses TGF-β1-induced fibrosis, placing PTPRC at the core of the inflammation–fibrosis regulatory axis.

Notably, immune cells are indispensable in the inflammatory and fibrotic progression of the DMD muscle microenvironment (52). Macrophages, with their high plasticity, are considered central regulators of the DMD muscle microenvironment (53). Macrophages are broadly classified into classically activated M1 (pro-inflammatory) and alternatively activated M2 (anti-inflammatory/pro-repair) phenotypes (54). In normal muscle injury repair, M1 macrophages clear necrotic tissue in the early stage; however, in the chronic injury setting of DMD, sustained muscle fiber necrosis maintains macrophages in a pro-inflammatory state, while aberrant M2 macrophage accumulation drives muscle fibrosis via secretion of pro-fibrotic factors such as TGF-β1 (14). Single-cell analyses have identified pro-fibrotic macrophage subsets expressing galectin-3 and osteopontin in the DMD microenvironment, which directly participate in fibrotic remodeling by directing mesenchymal progenitor cell differentiation (48). Capote et al. (55) demonstrated that reducing macrophage infiltration or modulating their polarization ameliorates muscle pathology in DMD mouse models. Our immune infiltration analysis revealed significantly elevated M2 macrophages in the PTPRC-High group, suggesting that high-PTPRC expression status is associated with increased M2-type macrophage infiltration and features of an immunosuppressive microenvironment. As PTPRC/CD45 serves as a pan-leukocyte marker, its expression level in bulk transcriptomic data can be confounded by the degree of immune-cell infiltration. The observed correlation between PTPRC and M2-macrophage proportion in this study may partially reflect alterations in immune-cell composition, and whether PTPRC directly modulates macrophage polarization still requires further cell-type-specific experimental validation.

In summary, this transcriptomic-based study reveals synergistic interactions among ECM activation, abnormal inflammation, and immune cell infiltration in DMD muscle and identifies PTPRC as a core hub gene. Several limitations should be noted: first, functional validation was performed only in C2C12 myoblasts, lacking confirmation in animal models or patient samples. Second, the impact of immune cells in DMD was inferred bioinformatically without experimental support. Future studies should integrate clinical samples and animal models to further clarify the dynamic regulatory mechanisms of the inflammation–fibrosis network in the DMD microenvironment.

## Data Availability

The original contributions presented in the study are included in the article/Supplementary material, further inquiries can be directed to the corresponding author.

## Footnotes

1
https://www.ncbi.nlm.nih.gov/geo/query/acc.cgi?acc=GSE288958

2
http://bio-bigdata.hrbmu.edu.cn/CellMarker/

## Supplementary material

SUPPLEMENTARY FIGURE S1 **(A)** Comparison of ECM activation scores between DMD and control groups in the validation cohort. **(B)** ROC curve derived from the validation cohort. **(C)** Comparison of PTPRC expression in the validation cohort.

SUPPLEMENTARY FIGURE S2 **(A)** Violin and scatter plots of *nFeature*, *nCount*, and *percent.mt* before quality‑control filtering. **(B)** Violin and scatter plots of *nFeature*, *nCount*, and *percent.mt* after quality‑control filtering. **(C)** Elbow plot and JackStraw plot. **(D)** Batch‑effect correction plot. **(E)** Dot plot and UMAP plot for cell‑type annotation. **(F)** Violin plot showing PTPRC expression distribution.
