# Supplementary material for: Transcriptomics-based analysis of muscle injury mechanism mediated by inflammatory imbalance in the Duchenne muscular dystrophy muscle microenvironment
Source: Front Neurol. 2026 Sep 15;17:1905030. doi: 10.3389/fneur.2026.1905030 (PMC13619489; doi:10.3389/fneur.2026.1905030)
Supplement: Supplementary file 1 [file Data_sheet_2.pdf]

Supplementary Table S2 Comprehensive ECM signature gene set.
ECM_signatureECM_related_signatureAGRNCD36CD44CD47CHADCOL11A1COL11A2COL1A1C

OL1A2COL2A1COL3A1COL4A1COL4A2COL4A4COL4A6COL5A1COL5A2COL5A3COL6A1COL6A

\2COL6A3COL6A6COMPDAG1FN1GP1BAGP1BBGP5GP6GP9HMMRHSPG2IBSPITGA1ITGA10ITG/

A11ITGA2ITGA2BITGA3ITGA4ITGA5ITGA6ITGA7ITGA8ITGA9ITGAVITGB1ITGB3ITGB4ITGB5ITGB6

ITGB7ITGB8LAMA1LAMA2LAMA3LAMA4LAMA5LAMB1LAMB2LAMB3LAMB4LAMC1LAMC2LAM

C3RELNSDC1SDC2SDC3SDC4SPP1SV2ASV2BSV2CTHBS1THBS2THBS3THBS4TNCTNNTNRTNXB

3VTNVWFACTBACTG1ACTN1ACTN2ACTN3ACTN4AKT1AKT2AKT3ARHGAP35ARHGAP5BADBCA

R1BCL2BIRC2BIRC3BRAFCAPN2CAV1CAV2CAV3CCND1CCND2CCND3CDC42CRKCRKLCTNNB1

DIAPH1DOCK1EGFEGFRELK1ERBB2FLNAFLNBFLNCFLT1FLT4FYNGRB2GSK3BHGFHRASIGF1IGF1

RILKJUNKDRMAP2K1MAPK1MAPK10MAPK3MAPK8MAPK9METMYL10MYL11MYL12AMYL12BMY

YL2MYL5MYL7MYL9MYLKMYLK2MYLK3PAK1PAK2PAK3PAK4PAK5PAK6PARVAPARVBPARVGPD(

GFAPDGFBPDGFCPDGFDPDGFRAPDGFRBPDPK1PGFPIK3CAPIK3CBPIK3CDPIK3CGPIK3R
