# Supplementary material for: Transcriptomics-based analysis of muscle injury mechanism mediated by inflammatory imbalance in the Duchenne muscular dystrophy muscle microenvironment
Source: Front Neurol. 2026 Sep 15;17:1905030. doi: 10.3389/fneur.2026.1905030 (PMC13619489; doi:10.3389/fneur.2026.1905030)
Supplement: Supplementary file 2 [file Data_sheet_3.pdf]

Supplementary Table S3 ECM scores in Control and DMD groups.

sample	group	ECM_score
GSM9418:	CONTROL	0
GSM9418:	CONTROL	0.04917
GSM9418:	CONTROL	0.401703
GSM9418:	CONTROL	0.108401
GSM9418:	CONTROL	0.094253
GSM9418:	CONTROL	0.113254
GSM9418:	DMD	0.758704
GSM9418:	DMD	0.715546
GSM9418:	DMD	0.61724
GSM9418:	DMD	0.50751
GSM9418<	DMD	0.73793
GSM9418<	DMD	0.706682
GSM9418<	DMD	0.657454
GSM9418<	DMD	0.794043
GSM9418<	DMD	0.630999
GSM9418<	DMD	0.693195
GSM9418<	DMD	0.611009
GSM9418<	DMD	0.523092
GSM9418<	DMD	0.797152
GSM9418<	DMD	0.67499
GSM9418!	DMD	0.741721
GSM9418!	DMD	0.798436
GSM2934{	DMD	0.566997
GSM2934{	DMD	0.825459
GSM2934{	DMD	0.320324
GSM2934{	DMD	0.835164
GSM2934{	DMD	0.46546
GSM2934{	DMD	0.471162
GSM2934{	DMD	0.428978
GSM2934{	DMD	0.673182
GSM2934{	DMD	0.643238
GSM2934{	DMD	0.467467
GSM2934{	DMD	0.674143
GSM2934{	CONTROL	0.152683
GSM2934{	CONTROL	0.203417
GSM2934{	CONTROL	0.517685
GSM2934{	CONTROL	0.261004
GSM2934{	CONTROL	0.281859
GSM2934{	CONTROL	0.301151
GSM2934{	DMD	0.840669
GSM2934{	DMD	0.802246
GSM2934{	DMD	0.785484
GSM2934{	DMD	0.725437
GSM2934{	DMD	0.689472
GSM2934{	DMD	0.821622
GSM2934{	DMD	0.762964
GSM2934{	DMD	0.766196
GSM2934{	DMD	0.83087
GSM2934{	DMD	0.744476
GSM2934{	DMD	0.740382
GSM2934{	DMD	0.733203
GSM2934{	DMD	0.670579
GSM2934{	DMD	0.844144
GSM2934{	DMD	0.776476
GSM2934{	DMD	0.798408
GSM2934{	DMD	0.833683
GSM2934{	DMD	0.644988

GSM2934{	DMD	0.62419
GSM2934{	DMD	0.592492
GSM2934{	DMD	1
GSM2934{	DMD	0.49131
GSM2934{	DMD	0.758993
GSM2934{	DMD	0.504395
GSM2934{	DMD	0.816868
GSM2934{	DMD	0.537407
GSM2934{	DMD	0.076525
GSM2934{	DMD	0.563063
GSM2934{	DMD	0.208269
GSM2934{	DMD	0.469431
GSM2934{	DMD	0.474182
GSM2934{	DMD	0.230706
