# Supplementary material for: Transcriptomics-based analysis of muscle injury mechanism mediated by inflammatory imbalance in the Duchenne muscular dystrophy muscle microenvironment
Source: Front Neurol. 2026 Sep 15;17:1905030. doi: 10.3389/fneur.2026.1905030 (PMC13619489; doi:10.3389/fneur.2026.1905030)
Supplement: Supplementary file 3 [file Data_sheet_4.pdf]

Supplementary Table S4 6,087 DEGs in the DMD group compared with the Control group.

gene	logFC	AveExpr	t	P.Value	adj.P.Val	B	regulation
HOXB3	1.864658	7.293742	15.37338	1.4061157	3.0031820	45.43646	Up
LSS	-1.13157	8.433269	-14.4207	4.7753054	3.6764528	41.99282	Down
HOXB-AS	2.352305	6.220572	14.3999	5.1640409	3.6764528	41.91627	Up
KIAA0319I	-1.14041	8.45024	-14.0123	2.2421967	1.1972209	40.47939	Down
PNPLA6	-1.55396	6.888746	-13.714	7.0340012	2.3761275	39.35965	Down
FZD10	4.073014	6.092973	13.72338	7.8314978	2.3761275	39.2565	Up
RIPK2	1.783715	5.486516	13.68009	8.0159373	2.3761275	39.23161	Up
COL3A1	3.49249	12.86063	13.72272	8.9001874	2.3761275	39.133	Up
SIDT2	1.205624	8.55405	13.47221	1.7914990	4.2514262	38.44347	Up
MYL5	5.21509	9.32641	13.48791	2.1869910	4.6709755	38.25205	Up
CLCN5	2.335332	7.239132	13.3877	2.4881436	4.8310702	38.12144	Up
PKN1	-1.67632	6.502114	-13.335	3.0549572	5.0512923	37.92022	Down
MEGF10	2.809665	8.798574	13.35419	3.0745762	5.0512923	37.91528	Up
FRMD8	1.465414	6.306507	13.20852	5.0077745	7.1791051	37.43554	Up
PEG3	2.846514	6.848561	13.21893	5.1142144	7.1791051	37.41598	Up
MYH14	-2.03332	6.848494	-13.1903	5.3781104	7.1791051	37.36556	Down
C17orf58	1.133086	7.499198	13.12699	6.8934837	8.6606485	37.12206	Up
GPR137B	2.85785	5.928187	13.08813	8.2493317	9.7882903	36.9464	Up
GNG2	2.505495	7.624389	13.06196	9.3003383	1.0454559	36.82906	Up
GAS5	1.644769	9.515938	13.02747	1.0194233	1.0886421	36.73823	Up
MYH8	7.708618	10.51522	13.08516	1.1830099	1.2031774	36.59903	Up
PCIF1	-1.8425	6.231987	-12.9378	1.4520188	1.4096463	36.39114	Down
LRP11	1.587057	7.481969	12.89409	1.7254654	1.6022821	36.22181	Up
TOGARAM	-3.80953	5.112447	-12.9079	1.8240182	1.6232242	36.16939	Down
HINT3	-2.22209	9.894846	-12.8532	2.1314227	1.8209171	36.01538	Down
CELF2	1.832965	10.52541	12.78422	2.6661685	2.1901549	35.7947	Up
GLT8D2	2.467122	7.799525	12.71954	3.6180216	2.8619891	35.49597	Up
TBC1D16	1.584713	6.847242	12.68392	3.9715892	3.0294715	35.40346	Up
C1orf127	-2.55697	4.590225	-12.6726	4.1548261	3.0599578	35.35918	Down
RGL1	2.478393	7.293198	12.65122	4.6869223	3.3367762	35.24158	Up
DCAF15	-1.44755	5.301511	-12.5997	5.5563465	3.7613439	35.07377	Down
DOCK8	2.511321	5.689144	12.59611	5.6354999	3.7613439	35.05988	Up
CASP4	2.178031	6.810874	12.57812	6.0551399	3.9189599	34.98935	Up
IRX5	-4.12213	4.388978	-12.5935	6.4323504	4.0406512	34.93254	Down
ERBB3	5.029253	7.346732	12.56104	8.1358132	4.9647056	34.70407	Up
YWHAQ	1.412915	10.82346	12.46895	9.3696166	5.5587853	34.5606	Up
STRN4	-1.56237	6.078858	-12.4548	9.9150938	5.7234209	34.50502	Down
TENT5A	2.275532	8.228984	12.44847	1.0525643	5.9159653	34.44706	Up
SLC38A3	-2.45786	4.384619	-12.3543	1.4839847	8.1269091	34.10889	Down
TAFAZZIN	-1.20782	6.066775	-12.3239	1.6771284	8.9550274	33.98869	Down
PRICKLE3	-1.9051	4.417533	-12.3144	1.7427691	9.0785522	33.95097	Down
PLEKHA2	1.683642	8.109002	12.27938	2.0064610	1.0203332	33.81254	Up
PWWP2B	-1.80427	6.414972	-12.2556	2.2078067	1.0966124	33.71858	Down
SRPX	3.320864	8.881916	12.2517	2.5174931	1.2114847	33.59225	Up
INTS1	-1.53771	6.454129	-12.2197	2.5525243	1.2114847	33.57603	Down
CILP	2.316511	9.820722	12.21687	2.7636962	1.2831961	33.4995	Up
SLC9A9	2.037112	6.400116	12.16558	3.1756356	1.4199125	33.36139	Up
DNAJC12	-3.26428	5.868214	-12.1812	3.1911135	1.4199125	33.35819	Down
SPARC	2.376049	11.9028	12.16164	3.6150629	1.5757247	33.23669	Up
CHRNA1	3.481333	9.700935	12.1575	3.8729054	1.6543502	33.17021	Up
TCFL5	1.296813	7.157979	12.06842	4.7055675	1.9706178	32.97492	Up
SMARCB1	-1.18744	7.442325	-12.0573	4.9222859	2.0217342	32.93067	Down
MPPED2	-3.23209	5.312837	-12.0123	6.3114425	2.5433922	32.68792	Down
COL1A2	3.035138	12.22863	12.01007	7.3384161	2.9024794	32.5432	Up
FKBP8	-1.65971	6.461715	-11.9345	8.1042222	3.1470905	32.44055	Down
MCM6	0.811301	8.049312	11.91345	8.8303876	3.2969313	32.35618	Up

MED12L	3.250152	5.868168	11.92972	8.8541922	3.2969313	32.35525	Up
CTSK	2.933282	8.577925	11.93583	8.9939990	3.2969313	32.34085	Up
GAS1	2.023644	9.860388	11.91489	9.1075452	3.2969313	32.32672	Up
NNMT	3.971039	10.56818	11.95145	9.2924666	3.3078083	32.31114	Up
NPNT	3.768284	7.618292	11.92539	9.8423613	3.4438097	32.25343	Up
PLEC	-2.04132	9.029891	-11.8886	9.9970129	3.4438097	32.23476	Down
INPP4B	-1.84985	7.408425	-11.8755	1.0304969	3.4714883	32.20436	Down
SNAI2	1.818682	7.982178	11.76855	1.5946578	5.2398004	31.77507	Up
SLC26A9	-4.24495	4.107371	-11.7891	1.6313799	5.2792444	31.75543	Down
TP53I3	2.864956	6.575003	11.76834	1.7001801	5.3132762	31.7137	Up
ADAMTS2	4.471975	7.165454	11.80018	1.7122102	5.3132762	31.71029	Up
PRTFDC1	2.37493	6.179701	11.75054	1.7165280	5.3132762	31.70265	Up
DTX3L	1.376378	8.177089	11.73387	1.8376186	5.5292453	31.63562	Up
COL6A3	2.271936	11.46134	11.75976	1.8380766	5.5292453	31.63813	Up
MEST	3.08072	9.266716	11.74849	1.9243205	5.6899142	31.59304	Up
JAM3	1.789064	8.257025	11.72001	1.9448834	5.6899142	31.57983	Up
TMEM45A	2.75901	5.858481	11.72738	1.9714095	5.6899142	31.56765	Up
CCDC8	2.324273	6.901348	11.70852	2.0840463	5.9348081	31.51245	Up
BCL7B	-1.48536	7.627793	-11.6941	2.1623320	6.0767221	31.47561	Down
DMTN	-2.49087	5.454494	-11.6878	2.2186336	6.1539709	31.45033	Down
MEIS2	2.547924	5.299379	11.67855	2.3047164	6.3107864	31.4129	Up
LHFPL2	2.205536	6.516801	11.67425	2.3456869	6.3416686	31.39557	Up
NCOR2	-1.08809	10.06394	-11.6564	2.5236978	6.7376424	31.32363	Down
CC2D1A	-1.49971	6.283924	-11.6379	2.7225792	7.1788699	31.24903	Down
OR7E12P	-2.03151	4.87837	-11.624	2.8821165	7.5068591	31.19303	Down
ENPP2	2.169574	8.344024	11.62517	2.9868487	7.6859176	31.159	Up
FST	3.777253	6.939945	11.63935	3.0396054	7.7285586	31.1438	Up
WDTC1	-1.43078	7.138749	-11.5923	3.2823989	8.2477031	31.06512	Down
RPL28	0.872169	12.67266	11.56596	3.6581635	9.0781712	30.95851	Up
UBTD2	1.303935	8.736055	11.56333	3.6979160	9.0781712	30.94788	Up
CNKSR2	-3.10867	8.019908	-11.5831	3.7779710	9.1693075	30.9296	Down
PFKL	-1.02596	6.875434	-11.5537	3.8466593	9.2311180	30.90909	Down
KIF1C	-1.67679	8.904533	-11.5353	4.1494141	9.8470208	30.83457	Down
SERINC5	1.909559	7.386111	11.52026	4.4145385	1.0313191	30.77365	Up
TYRP1	-5.39595	4.210589	-11.5655	4.4424272	1.0313191	30.77269	Down
EVI5L	-1.35721	5.883467	-11.4956	4.8859119	1.1139903	30.67386	Down
RFNG	-1.4394	6.577756	-11.4948	4.9028511	1.1139903	30.67045	Down
HOXB2	1.644701	7.453045	11.45662	5.7371990	1.2898431	30.51586	Up
RPL3	1.627167	12.08018	11.46187	5.8532378	1.3022234	30.49732	Up
DACT1	3.012003	6.037246	11.45226	6.2007426	1.3596456	30.44109	Up
EPS15L1	-2.29661	5.660978	-11.4354	6.2626464	1.3596456	30.42966	Down
TPST1	2.246299	5.510526	11.43383	6.3023184	1.3596456	30.42344	Up
CFAP46	-2.61467	5.075984	-11.4323	6.4340059	1.3741749	30.4035	Down
COL1A1	3.950601	11.92931	11.46957	6.5696041	1.3892436	30.38784	Up
NOTCH2	1.888781	9.794721	11.40073	7.3963308	1.5487336	30.26664	Up
MS4A4A	3.520285	7.26532	11.41533	7.6040756	1.5767752	30.24186	Up
TSC2	-1.45961	7.531107	-11.379	7.9013909	1.6226721	30.20099	Down
NBPF12	1.730033	10.32571	11.3778	8.0157128	1.6304723	30.18712	Up
XYLT1	2.173318	5.729102	11.36796	8.2716336	1.6526955	30.15594	Up
MFHAS1	1.409615	6.331647	11.36772	8.2797278	1.6526955	30.15497	Up
RAP2B	2.048326	8.101912	11.34634	9.2344774	1.8262034	30.0482	Up
SYNE3	2.096657	8.800946	11.33974	9.6828371	1.8973030	30.00213	Up
NBPF11	1.791085	9.852083	11.31982	1.0190983	1.9787183	29.95091	Up
MAP2K2	-1.38846	9.231202	-11.3136	1.0357686	1.9823799	29.93467	Down
SLC41A1	-1.97609	9.363995	-11.3207	1.0395474	1.9823799	29.93204	Down
GUSB	1.139987	8.367394	11.3092	1.0546035	1.9886855	29.91694	Up
METTL25E	-1.32561	6.167753	-11.3059	1.0692729	1.9886855	29.90335	Down
HSPB6	-2.4898	11.27589	-11.3292	1.0707877	1.9886855	29.90478	Down

IQGAP2	2.545543	6.348772	11.30982	1.0955565	2.0148439	29.88063	Up
RGS3	-1.81342	8.020758	-11.2982	1.1037397	2.0148439	29.87213	Down
PGPEP1	-1.74051	6.69455	-11.2926	1.1297346	2.0448196	29.84923	Down
ETNPPL	-4.3011	4.007477	-11.311	1.1544680	2.0632807	29.83074	Down
FRMD6	2.103194	8.526472	11.29552	1.1592550	2.0632807	29.82495	Up
DCLK1	4.641486	9.284837	11.31889	1.2174036	2.1488682	29.78099	Up
FBXW5	-1.57835	7.639122	-11.2475	1.3614604	2.3834485	29.66565	Down
PALM2AK.	1.862789	9.737808	11.23859	1.4418360	2.5036370	29.60981	Up
SH2B2	-1.7646	8.249573	-11.2145	1.5609627	2.6886325	29.53109	Down
COL6A6	5.336513	6.932206	11.24717	1.6342268	2.7923052	29.49128	Up
CDC14B	2.140878	6.86711	11.20212	1.6550846	2.8054998	29.47369	Up
LAMC1	1.145692	10.23161	11.17784	1.8176313	3.0567693	29.38129	Up
COLEC12	2.711673	8.855374	11.19407	1.8698532	3.1200255	29.35626	Up
MCUB	2.627862	6.786655	11.1795	1.9105350	3.1631943	29.33393	Up
MYH3	7.227941	10.50717	11.22861	1.9404421	3.1741514	29.3251	Up
SBF1	-1.46619	6.88344	-11.1613	1.9468763	3.1741514	29.3137	Down
GALNT7	2.568554	6.640027	11.16262	2.0472380	3.3124931	29.2659	Up
ABCD1	-1.37022	6.184754	-11.1464	2.0715472	3.3266244	29.25262	Down
TMED3	2.937428	5.715803	11.13503	2.2969411	3.6610499	29.15267	Up
MYPOP	-1.77906	6.194094	-11.1054	2.4558964	3.8854101	29.08513	Down
C3	3.188381	10.73431	11.14392	2.4993409	3.9250679	29.07321	Up
CALM3	-1.67548	7.789349	-11.0946	2.5691426	3.9761378	29.04076	Down
RXRG	-2.59099	6.064844	-11.1035	2.5795378	3.9761378	29.03803	Down
COLQ	-2.165	6.33066	-11.0928	2.5877102	3.9761378	29.03367	Down
TRIP10	-1.63879	9.244318	-11.0625	2.9354982	4.4783122	28.90957	Down
THBS2	3.105099	7.562028	11.07465	3.0646168	4.6421338	28.87005	Up
OXNAD1	-1.12995	6.561021	-11.0494	3.1007222	4.6637483	28.85568	Down
NBPF14	1.685712	10.25435	11.03963	3.2556062	4.8624642	28.80795	Up
PPIA	1.119445	11.97749	11.02622	3.4146262	5.0645546	28.76077	Up
PIK3AP1	3.334954	5.711556	11.04443	3.4735238	5.1163808	28.74679	Up
OSTC	1.398644	9.062218	11.01617	3.5607002	5.2088655	28.71954	Up
ZCCHC9	-1.25569	7.463288	-11.0111	3.6367157	5.2838758	28.69875	Down
COL5A2	3.304709	9.743701	11.02749	3.9773362	5.7397261	28.61549	Up
HNMT	2.327116	8.636954	10.98485	4.2851515	6.1424339	28.53896	Up
NBPF1	1.658176	9.836597	10.96972	4.3217491	6.1535945	28.52889	Up
RFC3	1.492205	6.011664	10.96117	4.4786957	6.2931903	28.49378	Up
BEX4	1.881475	6.953744	10.96117	4.4787195	6.2931903	28.49377	Up
IGSF3	2.191883	6.167816	10.95891	4.5211476	6.3112856	28.48449	Up
CFHR1	2.490467	9.689718	10.97223	4.6872045	6.4282498	28.45185	Up
RPL15	0.574106	12.59654	10.94952	4.7017094	6.4282498	28.44595	Up
MCC	1.345149	7.709307	10.94916	4.7087798	6.4282498	28.44447	Up
PNMA8A	3.778418	6.734101	10.97996	4.7253264	6.4282498	28.44513	Up
NOVA1	2.069491	6.847135	10.92488	5.2158506	7.0506417	28.34383	Up
AMMECR1	1.926651	6.439082	10.91588	5.4108770	7.2682711	28.30767	Up
DBNDD2	-1.19753	10.56738	-10.9133	5.4704082	7.3023112	28.2969	Down
SLC2A10	2.481275	6.46317	10.9134	5.7135529	7.5795070	28.25548	Up
SFT2D2	1.328404	8.391574	10.89934	5.7981629	7.6442693	28.23962	Up
PLPPR4	2.879814	5.801178	10.89707	6.1761888	8.0927019	28.17915	Up
NCALD	1.28092	6.536951	10.87628	6.3847012	8.3149054	28.14477	Up
TRAPPC9	-1.38258	7.196921	-10.8725	6.4874400	8.3974997	28.12905	Down
RAD23A	-1.5885	11.31158	-10.8733	6.6210776	8.5188539	28.10974	Down
C5orf15	0.830105	9.249851	10.85317	7.0325955	8.9941422	28.04963	Up
PDGFRA	1.971246	10.35815	10.84963	7.5274163	9.5696761	27.9844	Up
ZNF787	-1.71236	5.866507	-10.8351	7.5858647	9.5809154	27.97508	Down
CFI	2.420011	7.033149	10.84651	7.6259744	9.5809154	27.97159	Up
TMEM176	2.545512	7.544702	10.84153	7.9062680	9.8088967	27.93655	Up
LGALS1	1.089438	12.16444	10.82464	7.9246778	9.8088967	27.93207	Up
PDLIM2	-1.74546	7.78662	-10.824	7.9452155	9.8088967	27.92952	Down

PTGFRN	2.84555	7.678474	10.83507	8.2936262	1.0180187	27.89014	Up
PROS1	2.180615	8.097681	10.80937	8.8464747	1.0796743	27.82523	Up
MS4A7	3.346779	7.971696	10.8203	9.1707339	1.1128894	27.79243	Up
TNFRSF10	1.315998	7.127114	10.78222	9.4653027	1.1421465	27.75718	Up
LRRC8D	0.935534	7.235007	10.7668	1.0097210	1.2115517	27.69356	Up
EPB41L4A	1.538778	8.129787	10.75916	1.0425841	1.2439951	27.66203	Up
ZNF512	0.959399	7.477151	10.75726	1.0509116	1.2469650	27.6542	Up
SCYL1	-1.1862	7.401324	-10.7529	1.0705061	1.2631972	27.63601	Down
THOP1	-1.22578	6.152677	-10.7478	1.0932412	1.2829366	27.61532	Down
EMILIN2	2.181095	5.910977	10.74365	1.1126677	1.2921312	27.59798	Up
COL5A1	2.665042	8.707128	10.7645	1.1131760	1.2921312	27.6004	Up
CCNG2	1.819233	9.210887	10.74645	1.1195733	1.2925322	27.59248	Up
DAPK1	1.946526	7.03605	10.72281	1.2142875	1.3943415	27.51194	Up
MAFB	1.327863	9.667039	10.71759	1.2411778	1.4175976	27.49038	Up
URGCP	-1.13125	7.144214	-10.7149	1.2553379	1.4261440	27.47921	Down
CLEC11A	2.955458	5.615682	10.71742	1.3080314	1.4761770	27.44044	Up
TTC28	0.905485	7.322192	10.70329	1.3179753	1.4761770	27.43127	Up
ADPGK	0.995755	6.972026	10.7029	1.3201134	1.4761770	27.42968	Up
SSR2	0.953996	9.318201	10.69548	1.3618722	1.5149410	27.39902	Up
IL10RA	2.438943	6.219965	10.69642	1.4063492	1.5563112	27.36857	Up
GNB4	2.057477	8.447806	10.69464	1.4255175	1.5693920	27.35545	Up
TMSB10	1.754892	11.93942	10.69315	1.4479842	1.5859511	27.34036	Up
CRYBG1	2.352343	5.962151	10.68255	1.4643210	1.5956616	27.32821	Up
NDN	1.760484	9.110972	10.66839	1.5390439	1.6637098	27.27889	Up
FAM171B	2.035732	6.206597	10.66486	1.5488217	1.6637098	27.27237	Up
MAN2A2	-1.56886	8.18651	-10.6647	1.5501369	1.6637098	27.27154	Down
TSHZ3	1.651847	6.262662	10.65831	1.5920269	1.7001255	27.24529	Up
GRAMD1A	-1.16995	6.841763	-10.6568	1.6023336	1.7026190	27.23893	Down
FNDC3B	1.217951	9.289541	10.65247	1.6315676	1.7251000	27.22113	Up
LRRC17	4.115487	8.190447	10.68282	1.6889259	1.7682391	27.19251	Up
KRT31	-3.1873	5.244609	-10.6608	1.7153346	1.7871276	27.17468	Down
CFH	2.519635	8.504761	10.65802	1.7371094	1.7954640	27.16229	Up
TLE2	-1.60583	7.725885	-10.6365	1.7449404	1.7954640	27.15499	Down
PLP1	3.556978	6.21739	10.65645	1.7485556	1.7954640	27.15583	Up
BCAT1	3.027555	7.254354	10.65444	1.7633375	1.8019790	27.14754	Up
PDIA3	1.214803	9.060135	10.62422	1.8373010	1.8686226	27.10421	Up
ZNF628	-1.74182	4.341252	-10.6072	1.9733772	1.9909068	27.03386	Down
CD47	1.091355	9.708091	10.60689	1.9761786	1.9909068	27.03247	Up
PAQR9	-4.30756	4.827604	-10.6306	2.0267745	2.0322934	27.01178	Down
SSR1	1.418957	10.05613	10.58296	2.1855820	2.1812925	26.9333	Up
RCC2	1.108752	6.977848	10.58184	2.1958536	2.1813507	26.92868	Up
DHCR24	-2.87387	7.018654	-10.5928	2.2825559	2.2569828	26.89343	Down
APBB1	-1.23391	6.126186	-10.5547	2.4614556	2.4226622	26.81626	Down
SIVA1	-1.50106	7.871389	-10.5529	2.4808780	2.4305776	26.80852	Down
VCAM1	3.55598	7.253943	10.5807	2.4966521	2.4348628	26.80648	Up
KIDINS22(	1.305099	8.190975	10.54473	2.5672376	2.4923209	26.77483	Up
DGAT1	-1.29968	7.189549	-10.5365	2.6575425	2.5683164	26.74079	Down
FBN1	2.011226	10.57444	10.54773	2.6799974	2.5783506	26.73441	Up
SHARPIN	-1.40455	6.215834	-10.5303	2.7285239	2.6132652	26.71483	Down
FAM111A	1.28828	5.448618	10.52794	2.7554241	2.6272477	26.70517	Up
FLII	-1.26268	9.883841	-10.5259	2.7792805	2.6382166	26.69669	Down
RPL18	0.712304	12.41645	10.51118	2.9571485	2.7934971	26.6356	Up
CDC23	0.673582	7.272365	10.5095	2.9781221	2.7934971	26.62865	Up
RNF126	-1.44858	7.018081	-10.5092	2.9821020	2.7934971	26.62733	Down
HSPBP1	-1.22437	6.482379	-10.5059	3.0239759	2.8203527	26.6136	Down
DEPP1	1.867064	8.026452	10.50718	3.0497369	2.8320122	26.60573	Up
TMUB1	-1.18108	5.744921	-10.4916	3.2118680	2.9696570	26.55425	Down
ABI3BP	2.554972	9.552968	10.50627	3.2812369	3.0207180	26.53608	Up

RNF125	2.196438	6.210389	10.48773	3.3014368	3.0262698	26.52755	Up
FYN	1.33059	8.409401	10.48329	3.3261708	3.0359126	26.51981	Up
ANKRD52	-1.22652	6.025493	-10.4733	3.4687680	3.1377465	26.47848	Down
MATN2	2.901254	7.457515	10.49254	3.4759682	3.1377465	26.47931	Up
HTRA1	2.145718	9.185016	10.48413	3.4818145	3.1377465	26.47649	Up
CLSTN2	2.216796	7.153376	10.47078	3.6502520	3.2757178	26.42966	Up
WIPF1	1.942037	7.983251	10.46375	3.6988609	3.3054507	26.41606	Up
MPG	-1.02126	7.488473	-10.4548	3.7509918	3.3380701	26.40146	Down
ROBO1	1.621596	8.041895	10.45125	3.8075308	3.3627254	26.38672	Up
FBXO3	-1.15366	9.930998	-10.4504	3.8208453	3.3627254	26.38329	Down
CASP1	1.685481	7.141152	10.44933	3.8386328	3.3627254	26.37871	Up
ATG4D	-1.7734	6.576751	-10.4491	3.8416752	3.3627254	26.37793	Down
BLNK	2.966857	4.936073	10.45767	3.8920028	3.3928733	26.36683	Up
SLC25A23	-1.62911	7.170716	-10.4364	4.0542517	3.5199474	26.3249	Down
NR1H2	-1.41952	7.354804	-10.4324	4.1237041	3.5657519	26.30817	Down
CCDC80	3.888867	9.65592	10.46533	4.1888258	3.6074573	26.29815	Up
GADD45A	2.893558	9.168154	10.44907	4.2130456	3.6137441	26.29028	Up
VEGFB	-1.35082	7.608814	-10.4171	4.3973914	3.7470495	26.2449	Down
TMEFF2	2.811737	5.89074	10.4276	4.4174404	3.7470495	26.24213	Up
LAMA4	2.163336	8.696217	10.4274	4.4210904	3.7470495	26.24131	Up
ZC3HAV1	1.398782	7.684709	10.41375	4.4609130	3.7658569	26.23077	Up
ZNF688	-1.17228	6.822131	-10.4019	4.6895417	3.9432768	26.18156	Down
TNNT2	5.077313	7.661422	10.43705	4.7154196	3.9494875	26.18154	Up
TSSC4	-1.20008	6.250963	-10.3974	4.7793281	3.9873785	26.16288	Down
OLFML1	2.153435	7.397205	10.40577	4.8003635	3.9893449	26.15995	Up
ADCY7	1.93247	7.228094	10.39349	4.9003664	4.0566677	26.13855	Up
ADGRB3-I	-3.52288	3.514552	-10.4059	4.9559182	4.0868147	26.12969	Down
RAB21	-0.94077	9.756821	-10.3868	4.9996039	4.1069823	26.11851	Down
EXTL3	-1.36132	5.20103	-10.382	5.1016358	4.1747409	26.09861	Down
ZWINT	2.556106	5.340156	10.3776	5.3752349	4.3818423	26.04837	Up
TMEM98	2.376209	6.470254	10.37407	5.4869060	4.4447081	26.02832	Up
SUCO	1.537418	6.469617	10.36446	5.4939739	4.4447081	26.02565	Up
MBD3	-1.30212	7.542735	-10.348	5.8901392	4.7472300	25.95709	Down
ADA2	2.389357	7.011498	10.35318	6.0450986	4.8538051	25.93323	Up
ARB2A	1.445961	7.022321	10.34066	6.0756329	4.8600512	25.92655	Up
EPB41L2	1.478006	8.989385	10.33663	6.1802050	4.9252544	25.90975	Up
PLAAT4	1.890092	7.115438	10.33164	6.3119964	5.0115843	25.88897	Up
CMTM3	1.910257	7.484119	10.32968	6.4551208	5.1062396	25.8674	Up
RPL12	0.564681	12.88757	10.3251	6.4890835	5.1141640	25.86172	Up
TENM1	2.301678	5.758537	10.32393	6.6317344	5.2073744	25.84091	Up
PEG10	2.770314	7.151132	10.33752	6.6720388	5.2198317	25.83722	Up
ZNF579	-1.62836	6.39549	-10.3163	6.7352687	5.2351229	25.82505	Down
TIMP2	1.817232	10.6406	10.32737	6.7406067	5.2351229	25.82598	Up
NFKBIB	-0.98452	6.265575	-10.3134	6.8173193	5.2521521	25.81313	Down
STARD10	-2.46083	6.682653	-10.3241	6.8347646	5.2521521	25.81232	Down
CD163	3.13069	7.965253	10.33571	6.8363063	5.2521521	25.81387	Up
LASP1	1.235419	9.185469	10.30263	7.1363541	5.4630198	25.76809	Up
CMTM7	2.05567	6.209286	10.30042	7.2034496	5.4946885	25.75887	Up
CD99	1.46083	11.08981	10.28937	7.6083291	5.7788181	25.70531	Up
OR7E47P	-3.36348	5.632013	-10.3055	7.6353957	5.7788181	25.7044	Down
CHRNA10	-1.70931	5.082324	-10.286	7.6571099	5.7788181	25.69873	Down
PSKH1	-1.22838	5.473232	-10.2684	8.2480354	6.2028711	25.62552	Down
FAT1	2.06949	7.694362	10.26805	8.5857777	6.4342119	25.58739	Up
DPP9	-1.53313	6.622798	-10.2511	8.8782881	6.6301565	25.553	Down
MS4A6A	2.833195	8.251452	10.26757	8.9602328	6.6680366	25.54683	Up
SPEG	-1.89795	7.41222	-10.2469	9.1387153	6.7772459	25.52494	Down
FBXW4	-1.19863	8.241509	-10.243	9.1889924	6.7909516	25.51913	Down
CITED4	-1.93762	6.244314	-10.2382	9.3766199	6.9057189	25.49922	Down

HCLS1	1.932103	7.160026	10.23874	9.4391632	6.9278917	25.493	Up
LPAR1	2.232396	7.730025	10.24308	9.6237141	7.0291458	25.47531	Up
STARD13	0.973023	6.917139	10.23157	9.6429428	7.0291458	25.47164	Up
HDDC3	-1.06447	6.915228	-10.2258	9.8821401	7.1790050	25.44751	Down
SLC1A4	-2.45309	7.926728	-10.2289	1.0550026	7.6382192	25.38598	Down
ACOT8	-1.4278	6.261544	-10.2074	1.0683014	7.7083723	25.37076	Down
TCF7L2	1.498157	9.274616	10.19969	1.1038953	7.9383825	25.33849	Up
SGCE	1.642084	8.517783	10.19405	1.1306221	8.1032980	25.31493	Up
RPL31	0.520932	13.5171	10.19156	1.1426169	8.1532717	25.30453	Up
ABTB2	-2.29746	5.90432	-10.1963	1.1452296	8.1532717	25.30311	Down
C1R	2.689425	9.431931	10.20178	1.1828622	8.3932133	25.27332	Up
TRAM2	1.644705	6.669265	10.18052	1.1974306	8.4684518	25.25839	Up
GNL3L	-1.32365	5.965129	-10.1633	1.2882314	9.0805433	25.1864	Down
CLIC1	1.709072	9.140763	10.16397	1.3021896	9.1487387	25.17629	Up
ORAI1	-1.83219	8.031514	-10.1607	1.3236293	9.2688771	25.16031	Down
MED9	-1.34823	5.834666	-10.1465	1.3832998	9.6550712	25.11628	Down
SLC2A4	-2.20044	5.892884	-10.1473	1.3953332	9.6784289	25.1082	Down
SMAD3	-1.03102	8.110456	-10.1444	1.3957093	9.6784289	25.10748	Down
VAC14	-1.17786	6.389046	-10.1434	1.4018304	9.6894160	25.10317	Down
HDGFL3	1.899367	7.645365	10.1461	1.4126105	9.7324313	25.09634	Up
CHCHD10	-1.41008	11.73653	-10.1408	1.4418470	9.8721867	25.07609	Down
FAM161A	1.948616	5.881275	10.13671	1.4421398	9.8721867	25.07525	Up
WDR75	0.860733	7.891555	10.13222	1.4699262	1.0030251	25.05646	Up
FKBP11	1.673217	6.890315	10.12151	1.5383349	1.0463616	25.01166	Up
PNPLA2	-1.30048	8.332247	-10.1132	1.5936696	1.0805586	24.97685	Down
LOX	3.944542	6.465232	10.14449	1.6119198	1.0894742	24.97103	Up
SELENON	-1.25542	6.838941	-10.1092	1.6209859	1.0921456	24.96011	Down
BTG3	1.574304	7.300914	10.10431	1.6549687	1.1115353	24.93968	Up
ASPSCR1	-1.29571	5.247943	-10.0947	1.7238968	1.1542002	24.89949	Down
NID1	1.905539	8.429352	10.10086	1.7334886	1.1569952	24.89522	Up
MRC2	2.019586	9.371975	10.09331	1.8144546	1.2072623	24.85077	Up
C1RL	1.878395	6.768593	10.07665	1.8613980	1.2346503	24.82391	Up
PRLH	-1.0129	4.93197	-10.0742	1.8808967	1.2437211	24.81365	Down
PLEKHM2	-1.04282	8.000502	-10.0707	1.9092860	1.2585966	24.79889	Down
ZER1	-1.48721	6.655391	-10.0691	1.9220399	1.2631055	24.79233	Down
MAFK	-1.1066	8.415229	-10.0625	1.9771546	1.2953395	24.76449	Down
GRAMD1E	1.124739	6.166021	10.06007	1.9974187	1.3046137	24.75445	Up
PAPSS1	1.435661	8.331071	10.05899	2.0065898	1.3066080	24.74994	Up
ANKRD40	-1.22095	9.25929	-10.0536	2.0527763	1.3326199	24.72752	Down
TIAM1	2.586912	5.657417	10.05671	2.1190966	1.3715050	24.69791	Up
MLST8	-1.17145	6.526083	-10.0447	2.1326480	1.3761056	24.68993	Down
TNFRSF21	1.772256	7.845305	10.03869	2.2039464	1.4178279	24.65782	Up
ABCB1	-2.03801	5.554881	-10.035	2.2225236	1.4223403	24.64927	Down
TNFSF13B	2.884728	7.005349	10.0525	2.2242798	1.4223403	24.65136	Up
CREB3L2	1.101163	8.766663	10.03004	2.2695881	1.4469810	24.62863	Up
MBNL3	2.200113	5.87861	10.02956	2.3094306	1.4658126	24.61208	Up
C1orf105	3.109103	6.494746	10.04328	2.3128516	1.4658126	24.6129	Up
TANC2	2.426942	6.333142	10.03513	2.3223105	1.4674529	24.60772	Up
RNPEPL1	-1.22617	5.888117	-10.0196	2.3722287	1.4945741	24.58507	Down
ZNF521	1.840114	7.04471	10.01481	2.4215377	1.5211530	24.56481	Up
ZFAS1	1.562749	9.889905	10.01327	2.4484040	1.5335194	24.55411	Up
SLC12A7	1.41483	7.350484	10.00725	2.5007166	1.5617048	24.53312	Up
MMP2	2.214368	9.585513	10.02006	2.5519790	1.5890719	24.516	Up
FMO1	3.887549	5.782891	10.02376	2.6003874	1.6145079	24.49881	Up
MAP1S	-1.37364	6.014053	-9.98845	2.7090885	1.6771221	24.45429	Down
DNM2	-1.29657	7.316984	-9.98008	2.8074229	1.7329751	24.41917	Down
IFIT1	1.439578	9.559855	9.978766	2.8231608	1.7376677	24.41367	Up
ZFPM1	-1.38299	6.396389	-9.97766	2.8364895	1.7408547	24.40903	Down

PCDH18	2.201103	7.974804	9.984204	2.8829681	1.7643104	24.39472	Up
LSM14B	-1.59274	6.09895	-9.97084	2.9201475	1.7819574	24.3804	Down
TBRG4	-1.17615	6.634749	-9.96866	2.9472995	1.7924942	24.37128	Down
PCOLCE	2.555521	8.99737	9.984728	2.9644524	1.7924942	24.36843	Up
MIF	1.424992	9.8975	9.966949	2.9688966	1.7924942	24.36409	Up
RPS6	0.522236	13.08671	9.966784	2.9709849	1.7924942	24.3634	Up
MYLK4	-3.22425	6.862571	-9.98159	3.0303996	1.8130848	24.3471	Down
HSD17B11	1.308965	8.096452	9.961867	3.0338630	1.8130848	24.34277	Up
LGMN	2.193495	9.042098	9.9778	3.0374176	1.8130848	24.34429	Up
SZRD1	-0.89339	8.087011	-9.96086	3.0469712	1.8130848	24.33852	Down
RAB8B	1.521839	7.849187	9.96081	3.0475580	1.8130848	24.33833	Up
PLEKHA7	1.779258	5.27746	9.955195	3.1213381	1.8518205	24.31477	Up
SAT1	1.966567	9.77651	9.96483	3.1303286	1.8520099	24.31364	Up
SOX9-AS1	-2.48884	6.499056	-9.9612	3.1790464	1.8756374	24.29843	Down
PPP3CC	-1.17696	8.075502	-9.94925	3.2014274	1.8836387	24.28982	Down
ASCC2	-1.30247	7.227325	-9.94728	3.2284675	1.8943299	24.28154	Down
DCN	1.401123	12.6522	9.954975	3.2382774	1.8948802	24.27994	Up
PRPF40B	-1.44672	5.352199	-9.9449	3.2612871	1.8981949	24.27157	Down
PARP3	-1.22595	5.282859	-9.94487	3.2617171	1.8981949	24.27144	Down
ZSWIM6	1.452582	7.032081	9.943917	3.2749942	1.9007425	24.26744	Up
CNOT3	-1.65059	5.492836	-9.9428	3.2906491	1.9028897	24.26275	Down
SMPD1	-0.9934	6.886268	-9.94238	3.2965128	1.9028897	24.26099	Down
OGN	3.974753	8.526158	9.973729	3.3139219	1.9077828	24.26118	Up
PITPNM2	-1.24598	5.403634	-9.93224	3.4420609	1.9762241	24.21844	Down
IQSEC2	-1.57771	6.010839	-9.92563	3.5405191	2.0236310	24.19066	Down
SEC11A	0.541221	10.96032	9.925424	3.5435809	2.0236310	24.18981	Up
SNTA1	-1.91232	8.8562	-9.92715	3.6451326	2.0760731	24.16337	Down
ANTXR2	1.548739	7.87977	9.912813	3.7392988	2.1240410	24.13686	Up
CDC25B	1.649376	6.859662	9.9097	3.7892610	2.1467118	24.12378	Up
PITX3	-1.91171	3.526148	-9.90869	3.8056801	2.1503099	24.11953	Down
RPL36A	0.578177	12.61737	9.907067	3.8320397	2.1594909	24.11273	Up
HENMT1	1.642111	6.305617	9.906273	3.8450394	2.1599651	24.10939	Up
NAV1	1.559166	8.221934	9.905238	3.8620503	2.1599651	24.10504	Up
FGL2	2.672454	9.690176	9.92435	3.8632206	2.1599651	24.10794	Up
UBALD1	-1.35858	5.041213	-9.90274	3.9034074	2.1767356	24.09455	Down
MRPL2	-1.27658	7.64799	-9.89492	4.0357203	2.2446592	24.06172	Down
PRCP	1.416819	8.520088	9.893616	4.0582902	2.2513496	24.05623	Up
PIK3CG	2.283132	4.775895	9.89328	4.0984110	2.2677166	24.04687	Up
GRK2	-1.44172	5.507944	-9.88765	4.1628498	2.2974198	24.03117	Down
COG7	-1.00989	6.200729	-9.88329	4.2411041	2.3345748	24.01283	Down
PRC1	1.791042	4.522441	9.87079	4.4733312	2.4560773	23.96032	Up
GLIPR2	1.594758	7.51588	9.853312	4.8197194	2.6394760	23.88686	Up
ZNF436	1.384321	8.141897	9.850909	4.8693961	2.6598609	23.87676	Up
PABPC1	1.233717	10.86849	9.84926	4.9037854	2.6718124	23.86983	Up
ESRRA	-1.52669	8.367231	-9.84527	4.9879271	2.7094183	23.85307	Down
GLT8D1	0.782843	8.697241	9.844793	4.9981778	2.7094183	23.85105	Up
FGF11	-1.65481	5.034909	-9.84388	5.0177877	2.7131622	23.84719	Down
DTX2	-1.51806	4.38125	-9.83836	5.1373832	2.7639868	23.82399	Down
ADCY3	1.21738	6.751088	9.833862	5.2369316	2.8103112	23.80509	Up
DESI1	-1.00026	6.507578	-9.82932	5.3394313	2.8581347	23.78599	Down
MPEG1	2.568325	7.972933	9.840467	5.4701331	2.9207775	23.76503	Up
CCND2	2.47161	8.26496	9.837716	5.5344995	2.9457779	23.75351	Up
PENK	3.77648	7.637219	9.852103	5.5445394	2.9457779	23.75423	Up
AHR	1.966422	7.940108	9.827998	5.5597952	2.9465535	23.74755	Up
MTSS1	1.263124	9.489773	9.818931	5.5816743	2.9508267	23.74229	Up
LAMP1	1.058204	11.1635	9.817613	5.6131607	2.9601453	23.73675	Up
PGAP6	-1.09426	6.751531	-9.81614	5.6485725	2.9714830	23.73056	Down
PAX7	1.681962	6.929129	9.80744	5.8624200	3.0764021	23.69395	Up

CD14	2.704345	8.070238	9.822911	5.8941373	3.0854653	23.6915	Up
PVALB	5.163781	6.782478	9.831224	6.0573458	3.1631489	23.6671	Up
MYD88	1.717409	7.686388	9.797732	6.1147258	3.1791158	23.65248	Up
KIAA2013	-0.92576	8.101511	-9.79746	6.1176917	3.1791158	23.65197	Down
IRF2BP1	-1.29345	5.836423	-9.79472	6.1897246	3.2087412	23.64044	Down
AP3S1	0.992593	9.906351	9.791161	6.2845671	3.2499710	23.62546	Up
TMT1A	1.168871	11.1061	9.790172	6.3111772	3.2499710	23.6213	Up
CDON	2.28014	6.154084	9.79812	6.3149077	3.2499710	23.62211	Up
RUNX2	2.082264	5.777596	9.782652	6.5449163	3.3602481	23.58565	Up
VCAN	2.504773	9.380498	9.783057	6.9832502	3.5766968	23.52448	Up
ANXA2	1.556839	11.68983	9.775051	7.0212006	3.5875311	23.51799	Up
RNF31	-1.03019	6.112534	-9.76007	7.1775837	3.6525793	23.49459	Down
HACD4	1.980462	5.697089	9.759901	7.1827106	3.6525793	23.49389	Up
CSRNP3	2.47448	4.729214	9.759175	7.4279495	3.7683170	23.46206	Up
S100A1	-2.05187	11.14871	-9.76746	7.4624899	3.7768687	23.4591	Down
B2M	0.901458	13.05119	9.749949	7.4948630	3.7828900	23.45199	Up
ARPC5	1.512402	9.374213	9.749483	7.5098107	3.7828900	23.45003	Up
ZNF853	-1.53905	6.786853	-9.7461	7.6192434	3.8289835	23.43578	Down
ABCB4	-2.37595	5.809316	-9.75313	7.6505436	3.8356880	23.43312	Down
LPAR6	1.444821	8.435353	9.740517	7.8032618	3.8946196	23.41227	Up
HSF4	-1.37495	5.602536	-9.74048	7.8045567	3.8946196	23.41211	Down
TMEM35B	1.102834	7.178204	9.733389	8.0447395	4.0051176	23.38225	Up
ZNF865	-1.14544	5.44694	-9.72917	8.1913544	4.0686267	23.36446	Down
ADAMTS9	1.707637	7.161584	9.72667	8.2792340	4.1021607	23.35395	Up
GPS2	-1.10091	8.630332	-9.72616	8.2972816	4.1021607	23.35181	Down
LY96	2.345129	7.183527	9.736016	8.3626165	4.1249137	23.34611	Up
CST3	1.246333	11.24668	9.720783	8.4903115	4.1782505	23.32915	Up
KCNJ11	-2.58091	5.828051	-9.72552	8.6727102	4.2582010	23.30991	Down
MARCKS	1.938381	10.18302	9.72177	8.8845656	4.3522145	23.28647	Up
TNIP1	-0.93983	9.598319	-9.70839	8.9525554	4.3754846	23.27694	Down
IRF2	-1.08384	7.515892	-9.70365	9.1358856	4.4517880	23.25697	Down
C1S	2.164053	10.29038	9.719125	9.1680990	4.4517880	23.25635	Up
PAM16	-1.47362	7.39538	-9.70275	9.1712085	4.4517880	23.25317	Down
RNF123	-1.52775	9.370712	-9.69957	9.2968471	4.5025410	23.23977	Down
PDGFD	2.248969	7.917338	9.713016	9.3545891	4.5202559	23.23627	Up
TOMM40	-1.0963	7.294262	-9.6934	9.5455902	4.5950796	23.21376	Down
GPM6B	2.634595	7.753872	9.709389	9.5563764	4.5950796	23.21549	Up
DNAJB6	-0.94772	10.30501	-9.6922	9.5947628	4.5950796	23.2087	Down
EEF1A1	0.968005	13.44574	9.692177	9.5954935	4.5950796	23.20862	Up
RBM42	-1.10829	7.325937	-9.68886	9.7326130	4.6503165	23.19465	Down
COL6A1	1.535426	11.05753	9.690591	9.9580121	4.7473933	23.17334	Up
LAIR1	2.23584	5.51711	9.685929	1.0037166	4.7744723	23.16505	Up
IPO13	-1.36133	8.473849	-9.67754	1.0215891	4.8486892	23.14691	Down
ZNF358	-1.28844	7.820733	-9.67673	1.0251155	4.8546381	23.14352	Down
BACE2	1.278477	7.69668	9.673518	1.0393087	4.9109638	23.12997	Up
CHSY1	1.353659	7.91738	9.668299	1.0627873	5.0108194	23.10797	Up
RGS2	2.443231	8.855829	9.673611	1.1130565	5.2270151	23.06529	Up
CHN1	2.007154	6.838986	9.662085	1.1175618	5.2270151	23.05945	Up
PGRMC1	1.238142	9.197446	9.656378	1.1184261	5.2270151	23.05771	Up
TRMT2A	-0.97794	5.516246	-9.65638	1.1184314	5.2270151	23.0577	Down
BAG6	-0.96873	9.926501	-9.65466	1.1266681	5.2540127	23.05048	Down
MRC1	2.396614	8.33366	9.668997	1.1351635	5.2820964	23.04592	Up
POLR3E	-1.06325	8.638553	-9.6517	1.1410287	5.2867694	23.038	Down
ATP6AP2	1.431759	9.908771	9.651686	1.1411184	5.2867694	23.03792	Up
STAMBPL1	1.63389	6.468742	9.645427	1.1721097	5.4185975	23.01153	Up
RPS21	0.511677	13.02562	9.642793	1.1854067	5.4682328	23.00042	Up
HMGN2	0.755904	11.80887	9.626511	1.2710068	5.8429015	22.93174	Up
PLAGL1	2.22815	9.05342	9.642289	1.2720990	5.8429015	22.93373	Up

HSPB7	-1.49415	11.67522	-9.63223	1.2824918	5.8779958	22.92427	Down
SPIN1	1.573658	8.808533	9.622648	1.2922089	5.9098498	22.91544	Up
RNASE6	2.814811	6.330618	9.636491	1.3039474	5.9507927	22.90938	Up
VIM	1.474794	12.47478	9.625814	1.3275102	6.0454082	22.89058	Up
ABCA1	1.408348	8.815604	9.611516	1.3553183	6.1589127	22.86848	Up
BOP1	-1.52375	6.281177	-9.60873	1.3715958	6.2196483	22.85672	Down
POLRMT	-1.46553	6.977113	-9.60819	1.3747804	6.2208815	22.85443	Down
LAPTM5	2.641022	9.04061	9.624566	1.3789460	6.2265392	22.8545	Up
TUBA1A	1.562494	11.97659	9.614077	1.3957887	6.2892945	22.84118	Up
S100A10	1.731374	11.28176	9.615119	1.4006582	6.2979494	22.83808	Up
SH2B1	-1.02268	6.588367	-9.59849	1.4331022	6.4302934	22.81351	Down
DPYSL2	1.248477	10.47101	9.594017	1.4608351	6.5273046	22.79463	Up
LSM11	1.191077	6.350092	9.592764	1.4686996	6.5487446	22.78934	Up
NCLN	-1.42191	4.862813	-9.58594	1.5123013	6.7291107	22.76052	Down
FXR2	-1.35596	8.101935	-9.58296	1.5317390	6.7950547	22.74794	Down
ERAL1	-1.0391	7.378421	-9.58269	1.5334845	6.7950547	22.74682	Down
LINC0114(	1.944039	7.158246	9.585468	1.5557223	6.8729921	22.73374	Up
RMND5B	-0.97225	6.939962	-9.57906	1.5575092	6.8729921	22.73151	Down
TGFBR2	1.602853	9.961741	9.58313	1.5633722	6.8846402	22.7287	Up
WWTR1	1.853725	10.06324	9.585109	1.5797800	6.9425806	22.71921	Up
FAS	1.358595	6.690295	9.571793	1.6068028	7.0468366	22.70082	Up
TLR5	1.398396	5.313718	9.56594	1.6476218	7.2006697	22.67611	Up
TBC1D2B	1.470831	7.106908	9.565799	1.6486223	7.2006697	22.67551	Up
ENOX1	1.46738	4.968963	9.564731	1.6561838	7.2189338	22.671	Up
PABPC3	1.287252	10.73096	9.561567	1.6788018	7.3026170	22.65764	Up
SCN1B	-1.6641	9.188805	-9.5625	1.7047216	7.4002938	22.64337	Down
S100PBP	0.913815	6.788842	9.54872	1.7738555	7.6847883	22.60339	Up
FRMD4B	2.128422	7.126634	9.55498	1.7970163	7.7693674	22.5923	Up
COL14A1	3.925714	7.662234	9.573672	1.8082594	7.8021830	22.58979	Up
SRBD1	0.963495	5.432943	9.538975	1.8495510	7.9642563	22.56223	Up
PARP4	1.153491	7.664901	9.535919	1.8739497	8.0530822	22.54932	Up
STK26	2.85807	5.773474	9.550993	1.8781990	8.0551354	22.54992	Up
PTPRC	2.598978	7.322167	9.549463	1.8905179	8.0917198	22.54348	Up
MORC4	0.949127	6.924508	9.531514	1.9096844	8.1574079	22.53072	Up
TRIM41	-1.06981	7.476688	-9.51759	2.0271684	8.6419686	22.47191	Down
MARVELD	1.854951	7.328208	9.517309	2.0687825	8.8018045	22.45271	Up
PSD3	1.905577	5.916358	9.502853	2.1594907	9.1694639	22.40963	Up
DRAP1	-0.87856	7.662299	-9.50137	2.1733231	9.2098879	22.40334	Down
CTF1	-1.53538	4.750146	-9.49816	2.2033914	9.3188185	22.3898	Down
MAPRE1	0.818519	8.913277	9.49633	2.2207838	9.3738145	22.38206	Up
DNMT1	1.071424	7.539017	9.491294	2.2692934	9.5596784	22.36078	Up
KCNE3	2.037964	5.959563	9.488545	2.3168312	9.7407246	22.34074	Up
MN1	-1.94964	9.224562	-9.49431	2.3297301	9.7548944	22.33656	Down
PDGFRL	2.05033	9.109948	9.497747	2.3302243	9.7548944	22.337	Up
ADD3	1.801619	9.834715	9.493889	2.3339034	9.7548944	22.3348	Up
ZBTB7A	-0.94509	8.094292	-9.48353	2.3461218	9.7868106	22.32798	Down
ERLIN1	0.884848	7.606599	9.481887	2.3627732	9.8370585	22.32101	Up
AAAS	-1.04407	5.963895	-9.47198	2.4653573	1.0244183	22.27915	Down
PLXNB2	1.554929	6.92038	9.470633	2.4796890	1.0283727	22.27344	Up
ITGB8	3.398728	5.455071	9.481669	2.5257133	1.0434510	22.25815	Up
TNFAIP2	1.372756	8.454551	9.46609	2.5285152	1.0434510	22.25424	Up
CENPB	-1.16009	7.193926	-9.46589	2.5307033	1.0434510	22.25338	Down
RRBP1	1.542744	8.03098	9.462465	2.5681616	1.0568554	22.23891	Up
CKAP5	-0.83934	8.758684	-9.46177	2.5758873	1.0579962	22.23595	Down
CRIM1-DT	-1.45051	7.516159	-9.45884	2.6084648	1.0681231	22.22357	Down
PCYT2	-1.39156	5.800143	-9.45865	2.6105454	1.0681231	22.22279	Down
ITM2B	1.041755	11.78591	9.453833	2.6651077	1.0883627	22.20241	Up
CHRDL1	2.759546	9.260807	9.475525	2.6725063	1.0893013	22.2038	Up

CABIN1	-0.85065	6.883546	-9.44966	2.7132886	1.1018739	22.18476	Down
BEND7	-1.30211	6.673395	-9.44963	2.7136703	1.1018739	22.18463	Down
SRP72	0.640739	9.2518	9.441307	2.8123570	1.1397784	22.14944	Up
PXYLP1	1.423054	6.479659	9.440099	2.8269849	1.1435368	22.14433	Up
AGTR1	1.679115	8.407447	9.437103	2.9031454	1.1721244	22.11874	Up
GRK3	2.428113	6.25737	9.440131	2.9380792	1.1839904	22.10804	Up
VPS18	-0.726	5.764583	-9.42031	3.0777388	1.2377081	22.06062	Down
KYAT1	-1.08256	6.100164	-9.41991	3.0829699	1.2377081	22.05895	Down
SPP1	6.482756	7.440573	9.458436	3.1518364	1.2629816	22.04529	Up
MMP16	3.068652	5.408862	9.425732	3.2082925	1.2830542	22.02252	Up
FKRP	-1.27014	6.723742	-9.41023	3.2139433	1.2830542	22.01797	Down
TBX1	-1.8503	8.291328	-9.41622	3.2322720	1.2879639	22.01374	Down
GOLM1	2.521669	6.049277	9.415741	3.2693082	1.2999520	22.00292	Up
CPQ	1.176655	7.635221	9.405879	3.2745302	1.2999520	21.99957	Up
COMTD1	-1.77238	5.587946	-9.40344	3.3090703	1.3112268	21.98924	Down
FAM89B	-1.10561	9.566398	-9.40176	3.3329824	1.3182562	21.98215	Down
SYNPO	-1.90021	10.02659	-9.41113	3.3519253	1.3232979	21.97856	Down
NMI	1.315861	7.637827	9.396422	3.4102964	1.3438581	21.95956	Up
PSMB8	1.896764	6.538441	9.394228	3.4682999	1.3641979	21.94327	Up
PPIH	0.763405	6.782036	9.389416	3.5145016	1.3798295	21.92991	Up
IFT52	1.200366	6.607075	9.387906	3.5373812	1.3862640	21.92352	Up
SHTN1	2.404736	5.608658	9.395207	3.5617735	1.3932666	21.91842	Up
CD1C	2.059641	5.183515	9.385631	3.5721260	1.3947617	21.91389	Up
EHBP1L1	-1.18372	8.771949	-9.37962	3.6656305	1.4286594	21.88844	Down
PERM1	-2.80076	6.722373	-9.38984	3.7408103	1.4553046	21.87126	Down
N4BP2L1	1.328032	7.796391	9.371403	3.7973319	1.4746075	21.85367	Up
HEXA	0.858925	7.902434	9.370564	3.8110394	1.4772446	21.85012	Up
OPLAH	-1.79149	6.251665	-9.3666	3.8765604	1.4999199	21.83333	Down
NID2	2.226969	8.209881	9.37718	3.9491907	1.5252588	21.81786	Up
LINC0284(	-1.01644	4.700858	-9.35766	4.0283894	1.5530386	21.79549	Down
PLA2G2A	5.516	8.928666	9.395431	4.1189178	1.5850783	21.78168	Up
VPS72	-1.09132	8.227843	-9.34653	4.2257471	1.6232645	21.74838	Down
MAMLD1	1.11569	5.817014	9.34433	4.2659330	1.6357594	21.73906	Up
CIPC	-1.0376	9.749444	-9.34383	4.2751971	1.6363738	21.73692	Down
FBXO2	-1.13683	5.681003	-9.34331	4.2846029	1.6370402	21.73476	Down
CXCL12	1.65896	11.09865	9.350063	4.3224451	1.6482095	21.72776	Up
CTDNEP1	-1.08709	9.798814	-9.3409	4.3292702	1.6482095	21.72454	Down
CFAP20	0.726137	8.558052	9.339912	4.3477223	1.6522892	21.72035	Up
NINJ2	-2.51365	6.079731	-9.34913	4.3721266	1.6586124	21.71683	Down
LOC64673	-3.14072	5.023819	-9.35161	4.4061201	1.6685445	21.71002	Down
ASB11	-3.08671	6.446965	-9.34626	4.5240957	1.7092834	21.68415	Down
ZFP36L2	1.22704	10.48725	9.318289	4.7713239	1.7972828	21.62878	Up
BCAR3	-1.34169	7.559274	-9.31072	4.9291105	1.8509584	21.59673	Down
RGS10	1.677272	8.165125	9.313718	4.9311516	1.8509584	21.59692	Up
RPS17	0.570335	12.73778	9.301498	5.1286034	1.9216967	21.55765	Up
MIR29B2C	-2.0778	7.895292	-9.30868	5.1621362	1.9308740	21.5529	Down
ELOVL1	0.860059	6.871364	9.293754	5.3022998	1.9790875	21.52485	Up
SLC25A17	0.627538	5.783516	9.293435	5.3095663	1.9790875	21.5235	Up
SLC48A1	-1.15848	6.745631	-9.29085	5.3690531	1.9977741	21.51252	Down
CXCR4	2.823563	6.608721	9.303721	5.4092506	2.0092308	21.50798	Up
PCDHB10	2.17961	5.206149	9.288994	5.5021786	2.0402001	21.48914	Up
SCUBE2	1.72597	6.962311	9.281697	5.5845554	2.0671565	21.47376	Up
ROR1	1.298529	5.390661	9.280922	5.6032045	2.0704713	21.47048	Up
SLC25A43	1.957991	5.924129	9.281081	5.6252890	2.0750418	21.46681	Up
TEAD3	-1.1976	6.570255	-9.2763	5.7157435	2.1045125	21.45089	Down
ACTL6A	0.859086	7.298564	9.275928	5.7248889	2.1045125	21.44932	Up
WASF2	1.204177	8.391856	9.270821	5.8520628	2.1475662	21.42768	Up
DEPDC7	1.556923	6.619327	9.263433	6.0410834	2.2112809	21.39637	Up

CNRIP1	1.428753	7.895386	9.263229	6.0463904	2.2112809	21.3955	Up
EPB41L3	2.723222	7.272555	9.277163	6.0612515	2.2129266	21.39588	Up
SNAI3	-2.12886	5.750747	-9.26542	6.1062129	2.2255374	21.38667	Down
CD44	2.081834	10.11986	9.273425	6.1591415	2.2410041	21.3801	Up
ACY1	-1.26204	7.377786	-9.2584	6.1734255	2.2423813	21.37502	Down
CRISPLD1	3.732539	6.259426	9.278394	6.3680302	2.3091407	21.3497	Up
ACHE	-1.58832	8.122776	-9.2504	6.4137115	2.3217635	21.33758	Down
ABCF3	-1.03998	7.030232	-9.2491	6.4254197	2.3220662	21.33562	Down
PTTG1	1.886363	5.50591	9.248264	6.4485615	2.3264928	21.33207	Up
PKP2	-2.10927	4.980352	-9.24761	6.5117432	2.3453256	21.32278	Down
KCNJ12	-2.03929	7.417039	-9.25131	6.6034532	2.3714283	21.31035	Down
FURIN	-1.09761	6.439118	-9.2425	6.6103874	2.3714283	21.30766	Down
SDC2	1.622402	8.962345	9.246013	6.6241793	2.3714283	21.30638	Up
PLAGL2	-1.21469	5.827323	-9.24186	6.6286297	2.3714283	21.30495	Down
LAMB1	2.220372	9.14161	9.25485	6.6695942	2.3791296	21.30167	Up
GIMAP5	0.860835	8.287706	9.240334	6.6724349	2.3791296	21.29846	Up
TMEM259	-0.96583	6.184875	-9.23667	6.7784741	2.4129108	21.28293	Down
MTMR10	-0.89834	8.02196	-9.23238	6.9049502	2.4538423	21.26472	Down
HPN	-2.10211	5.001349	-9.22438	7.1959388	2.5530043	21.22438	Down
MAPKAPK	-0.91991	6.904636	-9.21815	7.3408632	2.6001021	21.20443	Down
SLC66A3	1.338844	8.008946	9.215905	7.4122737	2.6176005	21.19489	Up
RBP7	1.872715	7.051442	9.220741	7.4147781	2.6176005	21.19552	Up
BOC	1.400085	8.447593	9.206364	7.7230963	2.7219453	21.15443	Up
IL17RD	2.54192	6.712162	9.219765	7.7523677	2.7236227	21.15349	Up
MEDAG	1.492682	7.128974	9.205455	7.7533599	2.7236227	21.15058	Up
THSD1	1.204568	7.144373	9.202462	7.8539244	2.7544190	21.13789	Up
DOP1B	-1.37748	5.863263	-9.19574	8.0844686	2.8299112	21.10939	Down
PTPRE	1.48947	6.52995	9.195421	8.0956819	2.8299112	21.10802	Up
LGALS3	1.424945	11.44463	9.196981	8.2839976	2.8862907	21.08673	Up
HPRT1	0.790031	9.005353	9.189684	8.2981522	2.8865136	21.08369	Up
ANXA2P2	1.569014	9.958139	9.194754	8.3145227	2.8875053	21.08284	Up
CKLF	1.198633	7.554274	9.188688	8.3338323	2.8895128	21.07947	Up
TMEM243	1.397993	7.15118	9.186134	8.4260030	2.9167353	21.06864	Up
IRF8	2.332586	6.62575	9.194861	8.4754784	2.9291143	21.06485	Up
MYMX	3.385145	5.201302	9.199075	8.5102767	2.9363892	21.06182	Up
CMTM6	1.573962	9.029848	9.183782	8.6212410	2.9698784	21.04666	Up
IRF7	1.434546	7.147796	9.179677	8.6635961	2.9796632	21.04125	Up
HGS	-0.96461	7.108141	-9.17771	8.7373891	3.0002115	21.03289	Down
CD200	2.139783	5.514111	9.181196	8.7592118	3.0028771	21.03124	Up
CEP85	-1.45908	8.604523	-9.17471	8.8510488	3.0294984	21.02016	Down
MXRA5	3.481784	8.946957	9.199868	8.9073993	3.0439077	21.01914	Up
TBKBP1	-1.73255	5.718026	-9.17199	8.9551639	3.0553417	21.00865	Down
PATJ	-1.492	7.699636	-9.16474	9.2393915	3.1446740	20.97787	Down
PLCE1	2.192558	6.688109	9.17294	9.2464428	3.1446740	20.97877	Up
IL1R1	1.468122	8.718421	9.161095	9.3854475	3.1868742	20.96242	Up
SNN	-1.10239	9.010588	-9.15792	9.5145759	3.2255922	20.94896	Down
SLC16A14	1.732637	5.558353	9.153873	9.6820448	3.2719796	20.93178	Up
PLEKHH3	-1.19153	6.184543	-9.15338	9.7026291	3.2737559	20.92969	Down
SAPCD1-/	-1.28256	5.198539	-9.15269	9.7315990	3.2783516	20.92675	Down
POU4F1	2.05658	5.337382	9.151608	9.8450113	3.3113346	20.91566	Up
SLC6A8	-1.61343	8.881213	-9.15165	9.9403049	3.3381294	20.90662	Down
FAM234A	-1.05719	7.7163	-9.14518	1.0051587	3.3702008	20.89489	Down
PON2	1.303248	8.240564	9.142538	1.0166551	3.4015040	20.88369	Up
KIF21A	-1.44861	7.057724	-9.1423	1.0176800	3.4015040	20.88269	Down
CEP164	1.594383	6.563577	9.139614	1.0295416	3.4331933	20.87128	Up
CLIC4	1.840801	10.02269	9.149387	1.0303759	3.4331933	20.87246	Up
WWP1	-1.14806	10.92262	-9.138	1.0367202	3.4451435	20.86444	Down
CERS5	1.02263	6.779902	9.137897	1.0371885	3.4451435	20.86399	Up

TNFAIP8	2.058554	6.734995	9.141654	1.0508825	3.4852095	20.85242	Up
HNRNPC	0.641758	10.40451	9.131041	1.0682843	3.5371026	20.8349	Up
KCNMB4	1.513555	6.467498	9.130702	1.0698418	3.5371026	20.83346	Up
PCGF1	-0.99789	7.626974	-9.12722	1.0859963	3.5849629	20.8187	Down
RALGAPA:	-1.12127	7.90995	-9.12666	1.0886461	3.5881642	20.8163	Down
OR7E37P	-1.31181	7.418277	-9.12101	1.1154738	3.6709230	20.79232	Down
NECAB3	-1.48223	7.645571	-9.11986	1.1210206	3.6835012	20.78744	Down
FAM111A	1.330319	7.187583	9.116785	1.1359649	3.7268723	20.77439	Up
FBXL7	1.160643	6.854006	9.10989	1.1702232	3.8333784	20.74513	Up
BTBD2	-1.3681	6.788775	-9.10938	1.1728213	3.8360057	20.74295	Down
DAB2	2.20718	9.622645	9.122838	1.1750569	3.8374413	20.74384	Up
AHNAK2	2.704647	6.409813	9.12218	1.1783823	3.8424259	20.74106	Up
PGAP1	1.26149	6.401069	9.103987	1.2003782	3.9072213	20.72007	Up
IGFBP3	3.126826	9.665934	9.129817	1.2019123	3.9072213	20.72402	Up
SASH1	1.198797	9.459256	9.101107	1.2153702	3.9449662	20.70785	Up
EVI2A	2.640474	6.383524	9.113318	1.2240811	3.9672117	20.70359	Up
SRGAP1	1.757709	5.732475	9.097268	1.2356483	3.9986329	20.69155	Up
HMBS	-1.16214	7.373002	-9.09606	1.2421187	4.0134905	20.68641	Down
CEP15	1.957355	5.519963	9.09192	1.2644619	4.0795133	20.66885	Up
SLC7A7	2.189707	6.674934	9.093749	1.2996607	4.1867501	20.64345	Up
ZNF671	-1.4615	4.768321	-9.08331	1.3122520	4.2209455	20.63231	Down
DUSP3	-0.97644	10.74588	-9.08291	1.3145612	4.2220147	20.63058	Down
DCAF16	1.279878	7.434869	9.078434	1.3401460	4.2977235	20.61159	Up
BMERB1	-1.07145	8.200402	-9.07477	1.3614725	4.3595697	20.59604	Down
LDHD	-1.85503	6.222328	-9.07511	1.3654263	4.3656851	20.59339	Down
FOXO4	-1.06098	7.222344	-9.07265	1.3740072	4.3865539	20.58702	Down
VSIG4	2.115135	7.282256	9.079314	1.3829078	4.4083798	20.5823	Up
TXNDC12	0.690755	8.356768	9.070584	1.3862734	4.4125227	20.57827	Up
BAP1	-0.90136	7.366328	-9.06437	1.4238961	4.5255317	20.55189	Down
PGP	-1.34965	9.031214	-9.06232	1.4365535	4.5530634	20.54318	Down
PAG1	2.10277	6.880881	9.070423	1.4368221	4.5530634	20.54463	Up
UBL7	-1.04111	7.738275	-9.05631	1.4742778	4.6648334	20.51765	Down
HMGN1	1.39764	9.594968	9.055814	1.4774226	4.6678687	20.51555	Up
TMEM39A	0.600934	6.467433	9.055118	1.4818612	4.6749767	20.51259	Up
CLIP1	-1.13255	10.76614	-9.04375	1.5563158	4.9026245	20.46431	Down
CASTOR2	-1.41277	10.01364	-9.04345	1.5673906	4.9302399	20.4576	Down
TMEM120	-1.82767	7.79746	-9.04793	1.5727659	4.9398726	20.4553	Down
RNF128	-2.99795	6.044094	-9.05296	1.5862784	4.9749978	20.44829	Down
CD63	0.922503	12.0724	9.036723	1.6041926	5.0238044	20.43447	Up
ARL6IP1	1.194422	8.693034	9.035602	1.6119718	5.0407751	20.42971	Up
EEFSEC	-1.06586	6.747502	-9.03267	1.6324731	5.0974212	20.41726	Down
ACKR1	2.004601	7.174181	9.039192	1.6434506	5.1242072	20.4123	Up
GPC4	1.327905	8.514003	9.027256	1.6710477	5.2026586	20.39426	Up
ANKRD44	2.005796	6.16624	9.029898	1.6848727	5.2380657	20.38707	Up
ALOX5	2.442952	5.891794	9.02481	1.7483737	5.4275822	20.35135	Up
GPR183	1.767535	5.170444	9.015519	1.7578183	5.4489816	20.3444	Up
PSMB8-A:	1.525773	6.068044	9.015126	1.7608020	5.4503202	20.34273	Up
TOM1	-1.55746	6.496371	-9.0057	1.8338967	5.6683598	20.30267	Down
PCDH7	3.340513	5.800882	9.020202	1.8425799	5.6869686	20.3012	Up
CLTB	-1.11473	8.711411	-8.99814	1.8946327	5.8391869	20.27059	Down
UNC13C	-2.431	4.969238	-9.00237	1.9256735	5.9263020	20.25622	Down
ADRM1	-0.94301	9.169052	-8.98844	1.9756460	6.0713450	20.22935	Down
BUB3	0.801651	8.7971	8.986883	1.9889408	6.1034193	20.22274	Up
GXYLT2	2.668757	7.956493	8.996265	2.0377313	6.2441700	20.20195	Up
CLEC10A	1.930565	5.703364	8.980566	2.0526149	6.2785856	20.19191	Up
KLHL11	1.11496	7.513031	8.979328	2.0548419	6.2785856	20.19064	Up
CENPU	2.126199	5.641379	8.982146	2.0761553	6.3346465	20.18155	Up
CREBL2	1.071639	8.548915	8.97306	2.1111715	6.4156095	20.16401	Up

DEGS1	1.022944	8.818363	8.972797	2.1135717	6.4156095	20.16289	Up
EBPL	1.171696	7.63917	8.972487	2.1163980	6.4156095	20.16157	Up
MPRIP	-1.00187	9.481569	-8.97237	2.1175038	6.4156095	20.16106	Down
RBPJ	1.045322	10.07302	8.972343	2.1177098	6.4156095	20.16096	Up
TSR3	-1.06826	7.466608	-8.9718	2.1226385	6.4214327	20.15867	Down
GPC3	2.523091	6.782256	8.982528	2.1470154	6.4859910	20.15018	Up
CD84	2.473462	6.756696	8.980933	2.1617849	6.5213846	20.14342	Up
GPBP1L1	-0.68702	8.483218	-8.96655	2.1713362	6.5409591	20.13633	Down
XAB2	-1.36093	5.114109	-8.96537	2.1823726	6.5649456	20.13134	Down
RBM43	1.776355	5.671116	8.962881	2.2059616	6.6265723	20.12075	Up
ATP13A2	-1.10923	4.661199	-8.96107	2.2232616	6.6691603	20.11306	Down
ACER3	1.585352	6.606165	8.960304	2.2306326	6.6818866	20.1098	Up
ZBTB47	-0.92932	8.747015	-8.959	2.2432243	6.7101939	20.10426	Down
PELI1	1.239325	7.990097	8.958439	2.2486547	6.7170305	20.10188	Up
RPL22L1	1.553191	8.081182	8.958333	2.2617111	6.7347352	20.09643	Up
C6orf136	-1.48031	8.1424	-8.95691	2.2635566	6.7347352	20.09537	Down
ZFP36L1	1.670295	9.702545	8.964659	2.2640415	6.7347352	20.09677	Up
KIF2A	1.862321	6.462036	8.956317	2.2967777	6.8226119	20.0816	Up
PREPL	1.612439	10.23515	8.960634	2.3041733	6.8350741	20.07948	Up
GPX8	2.041536	7.140985	8.959644	2.3145371	6.8562947	20.07507	Up
RSPRY1	0.698975	8.125474	8.950476	2.3272737	6.8844755	20.06803	Up
FPR3	2.714809	5.591267	8.962796	2.3370944	6.9039644	20.06663	Up
DSEL	2.429097	5.724688	8.955816	2.3530025	6.9413576	20.05883	Up
FBRS	-0.83584	6.387075	-8.94194	2.4146011	7.1132484	20.03175	Down
ANXA5	1.205197	9.918327	8.938793	2.4476266	7.2006074	20.01837	Up
SMTN	-1.23478	8.858479	-8.93137	2.5273091	7.4247962	19.98682	Down
RWDD1	-0.64251	10.26475	-8.9285	2.5587580	7.5068617	19.97465	Down
ATP11C	1.440531	6.701957	8.924797	2.6000380	7.6175051	19.95888	Up
COX10	-1.45231	8.337028	-8.91873	2.6690366	7.8089432	19.93309	Down
KCTD2	-0.87808	6.946067	-8.91395	2.7246436	7.9607303	19.91278	Down
RBBP8	1.450894	5.64766	8.913475	2.7302683	7.9662665	19.91075	Up
RAP1B	0.959264	9.78296	8.911877	2.7491650	8.0104594	19.90396	Up
PODN	2.057519	7.15605	8.918192	2.7668923	8.0511289	19.89926	Up
AHNAK	0.925733	10.86532	8.909169	2.7814947	8.0826073	19.89245	Up
HLA-DMB	1.906484	8.438359	8.916402	2.7883059	8.0913912	19.89167	Up
ASS1	1.783255	9.317512	8.91455	2.8106411	8.1451388	19.88381	Up
TP53INP1	1.104593	7.87563	8.905168	2.8299496	8.1899815	19.87544	Up
SOWAHC	1.071195	6.628919	8.89489	2.9583477	8.5499852	19.83174	Up
TM7SF2	-1.46255	5.878496	-8.88503	3.0870570	8.9003027	19.7898	Down
LRIG3	1.130145	5.570169	8.884964	3.0878941	8.9003027	19.78954	Up
ASB16	-1.31075	6.097408	-8.88447	3.0944887	8.9072899	19.78743	Down
ZNF22	0.711161	9.007002	8.882451	3.1215715	8.9731526	19.77885	Up
SRCAP	-0.8785	4.891634	-8.88133	3.1367408	9.0046385	19.77408	Down
TRERF1	1.870478	4.834778	8.88078	3.1441807	9.0138807	19.77175	Up
SAMHD1	1.581222	8.95402	8.884182	3.1573338	9.0394553	19.76855	Up
ABRACL	2.30607	5.528959	8.886393	3.1731056	9.0724485	19.76435	Up
ARRDC1-/	-1.06901	5.723642	-8.87593	3.2106947	9.1676496	19.75113	Down
SESN3	2.854948	7.947269	8.893886	3.2274544	9.2032006	19.75002	Up
AGPAT5	1.024431	9.21366	8.873467	3.2450449	9.2410227	19.74065	Up
TRPC1	1.651231	6.168417	8.868651	3.3132354	9.4226474	19.72017	Up
NOTCH2N	2.051884	9.449553	8.880283	3.3326871	9.4653634	19.71714	Up
LINC0046	0.953647	6.119068	8.865149	3.3637171	9.5408063	19.70528	Up
CPD	0.759485	7.782068	8.864801	3.3687803	9.5424946	19.7038	Up
ARMCX4	1.972596	6.879785	8.870652	3.3761592	9.5507295	19.70298	Up
TMPO	0.779917	8.060438	8.85869	3.4588718	9.7717705	19.67781	Up
RNF34	-1.27474	9.448892	-8.85825	3.4654777	9.7774998	19.67593	Down
ERBB4	-2.87726	4.615871	-8.86177	3.6089326	1.0168810	19.63871	Down
SCGB1D2	-4.41144	3.045126	-8.86265	3.6190913	1.0183999	19.63627	Down

ATP1B3	1.630077	9.381192	8.853985	3.6267048	1.0191995	19.63247	Up
APEH	-1.07844	8.22843	-8.84129	3.7288720	1.0465341	19.60379	Down
BSG	-1.86783	8.528749	-8.84496	3.7933845	1.0632428	19.58852	Down
PSMD5	0.829607	7.170199	8.834581	3.8384316	1.0744590	19.57527	Up
SUPT6H	-1.1188	7.438765	-8.83396	3.8488067	1.0759530	19.57262	Down
PRMT2	1.129579	7.601775	8.83243	3.8742619	1.0816534	19.56612	Up
PEX5	-0.9446	8.572206	-8.83053	3.9061009	1.0891188	19.55807	Down
FDX2	-0.72411	6.859054	-8.82895	3.9328829	1.0951566	19.55134	Down
SAMD4A	-0.96167	10.8374	-8.82742	3.9589826	1.1009889	19.54482	Down
VGLL3	2.712828	7.505163	8.838609	4.0132778	1.1122282	19.53445	Up
LUM	2.683508	10.8444	8.848392	4.0135147	1.1122282	19.53646	Up
TANC1	1.174137	7.555517	8.824168	4.0150200	1.1122282	19.53098	Up
PRKN	-1.33526	5.654177	-8.82285	4.0378657	1.1171079	19.52539	Down
RILP	-1.26378	8.115587	-8.81469	4.1828328	1.1557172	19.49066	Down
CASP7	1.322856	7.267023	8.814002	4.1952566	1.1576523	19.48774	Up
CSF2RB	2.297331	5.927607	8.820188	4.2207261	1.1631776	19.48339	Up
ARFIP1	0.909649	7.695536	8.805957	4.3436055	1.1954990	19.45352	Up
ELF4	1.384317	5.855796	8.805458	4.3529867	1.1965391	19.4514	Up
DMXL2	2.382222	6.818603	8.815598	4.4023215	1.2085447	19.44302	Up
NEURL4	-1.25927	5.296224	-8.79886	4.4788108	1.2279645	19.42334	Down
MACROD1	-1.33302	9.67539	-8.79562	4.5419227	1.2436716	19.40956	Down
MAPK12	-1.52511	8.805312	-8.79775	4.5523837	1.2449399	19.40786	Down
POP5	0.59222	7.787488	8.793617	4.5814504	1.2511906	19.40102	Up
GCDH	-1.23493	6.713248	-8.79334	4.5869571	1.2511906	19.39984	Down
KDELR3	2.048817	7.254903	8.798948	4.6254174	1.2600722	19.39323	Up
CDK2AP2	-0.98507	6.299396	-8.78978	4.6581136	1.2673629	19.38468	Down
COL15A1	1.860423	11.59677	8.801229	4.6831971	1.2725664	19.38211	Up
MAN1A1	1.890003	8.651214	8.793878	4.7276318	1.2830084	19.37171	Up
IGF1	2.385581	9.830654	8.798154	4.7762489	1.2920789	19.36305	Up
TTC39A	-1.53316	3.948971	-8.78389	4.7781036	1.2920789	19.35964	Down
GPR34	2.47588	6.765431	8.796515	4.7792037	1.2920789	19.36212	Up
SPRED1	1.379245	6.841526	8.782483	4.8072129	1.2980082	19.35366	Up
ANKRD50	1.390299	7.72656	8.781803	4.8213613	1.3001848	19.35076	Up
RBMS1	1.424259	9.585166	8.782278	4.8408958	1.3038064	19.34708	Up
TUFT1	1.18928	6.21523	8.780395	4.8507935	1.3048268	19.34477	Up
CEP41	1.04535	4.677657	8.779158	4.8767735	1.3101651	19.33951	Up
LINC0062	0.905967	8.044187	8.778121	4.8986883	1.3143993	19.33509	Up
DSC2	1.632945	5.590313	8.776915	4.9242845	1.3196094	19.32996	Up
ZNF41	-1.40514	5.20423	-8.77565	4.9512972	1.3251855	19.32458	Down
DVL1	-1.00855	9.534406	-8.77394	4.9880653	1.3333554	19.31729	Down
ARL15	1.384393	7.360315	8.760414	5.2881935	1.4118154	19.25976	Up
LRRN4CL	1.325106	7.88301	8.759388	5.3116971	1.4163199	19.25539	Up
PRDX4	0.94921	9.555557	8.75321	5.4553991	1.4528231	19.2291	Up
SPIN4	2.462933	5.05085	8.759324	5.4871082	1.4594477	19.225	Up
HPS3	1.09147	6.524559	8.749144	5.5520948	1.4748960	19.2118	Up
HYAL4	-2.64869	4.592995	-8.75905	5.5893376	1.4829450	19.20769	Down
FLAD1	-1.04923	6.985553	-8.74714	5.6003021	1.4834264	19.20329	Down
HMGA1	-1.7602	7.022606	-8.75055	5.6050434	1.4834264	19.20323	Down
CA2	-2.38904	7.570414	-8.75831	5.6336001	1.4891390	19.20016	Down
NECAP2	0.913571	6.595234	8.742165	5.7221121	1.5106658	19.1821	Up
PIM3	-0.98166	9.019296	-8.73611	5.8738625	1.5488142	19.15633	Down
LGALS8	1.009419	7.241504	8.732022	5.9784921	1.5744591	19.13894	Up
ECM2	2.200055	8.690436	8.739871	6.0991658	1.6022876	19.12197	Up
ST3GAL3	-1.6412	7.507879	-8.7289	6.1287135	1.6080720	19.11508	Down
SMAD7	-1.42935	6.822775	-8.72489	6.1656701	1.6157838	19.10859	Down
SAMD1	-1.10536	4.70149	-8.7208	6.2755696	1.6425688	19.09119	Down
MARCHF1	1.857342	5.858156	8.720662	6.3107345	1.6497511	19.08594	Up
TRIM52-A	-1.37772	8.319227	-8.71501	6.4346522	1.6800892	19.06654	Down

HLA-DPB1	2.309925	10.10031	8.72693	6.4895286	1.6905158	19.0612	Up
GNS	0.929378	9.367804	8.713013	6.4904157	1.6905158	19.05804	Up
TPPP3	2.193244	8.6156	8.718762	6.6795885	1.7376693	19.03245	Up
GIMAP2	2.137813	6.317559	8.713227	6.6938940	1.7392723	19.02925	Up
RAB3A	-1.67497	6.206158	-8.70403	6.7473632	1.7510350	19.01981	Down
ARF4	0.811747	10.31717	8.703363	6.7668690	1.7535606	19.01697	Up
ELAVL2	2.697566	5.43632	8.715519	6.7735161	1.7535606	19.0187	Up
ACTG1	1.038587	12.53778	8.699382	6.8842972	1.7800825	19.00003	Up
SERPING1	1.659995	9.655666	8.705476	6.9214588	1.7875274	18.99633	Up
GTF2IRD2	-1.13539	8.301331	-8.69759	6.9378804	1.7896044	18.9924	Down
PCBP4	-1.34332	7.555532	-8.69435	7.0357624	1.8126636	18.9786	Down
FUT8	1.111797	6.356951	8.684509	7.3414518	1.8891413	18.93673	Up
ANTXR1	1.730829	9.828936	8.690889	7.3709700	1.8944546	18.93437	Up
GGH	1.166911	5.292755	8.68019	7.4798166	1.9201192	18.91834	Up
ARL4C	2.927208	5.440654	8.687304	7.6487843	1.9611372	18.89904	Up
PELI3	-1.45641	5.955427	-8.67189	7.7528975	1.9854482	18.88303	Down
STK17B	2.047682	7.034062	8.67663	7.8385622	2.0049821	18.87381	Up
CPTP	-1.42946	7.899025	-8.66396	8.0235733	2.0498502	18.84924	Down
FAM156A	0.859546	7.858242	8.662855	8.0618644	2.0571720	18.84456	Up
KPNA3	-0.69846	11.24103	-8.66106	8.1248184	2.0707621	18.8369	Down
TYROBP	2.368099	8.299755	8.672942	8.1369295	2.0713771	18.83812	Up
GAS2	-3.01632	5.899797	-8.6715	8.1983325	2.0845236	18.83078	Down
SH3BGRL	1.757011	9.934996	8.662908	8.3679247	2.1251145	18.80978	Up
DAAM1	1.444142	8.322604	8.653045	8.4111438	2.1335535	18.8028	Up
DDR2	1.744552	9.223714	8.659262	8.4484336	2.1404702	18.80004	Up
BNC2	1.78773	7.217514	8.656519	8.4787590	2.1456082	18.79609	Up
SULT1C4	0.954273	8.812358	8.645386	8.6943265	2.1975553	18.77019	Up
RPAP1	-1.03108	6.354225	-8.64399	8.7470051	2.2071957	18.76424	Down
RPS9	0.58242	12.0124	8.643827	8.7531360	2.2071957	18.76355	Up
SVIP	-0.76176	6.072428	-8.64099	8.8611914	2.2297230	18.75147	Down
PRICKLE2	1.410554	6.266952	8.640805	8.8682752	2.2297230	18.75069	Up
DENND6A	0.859674	8.983941	8.640627	8.8750989	2.2297230	18.74993	Up
ECT2	1.562163	4.993605	8.640389	8.8842320	2.2297230	18.74892	Up
RABL6	-0.86783	7.626218	-8.64	8.8993204	2.2308883	18.74725	Down
TWF2	-1.54145	8.56029	-8.63746	9.1226507	2.2841919	18.72355	Down
FAM50B	-1.37321	6.696043	-8.63345	9.1546443	2.2895186	18.7194	Down
CDC42SE2	0.809352	8.681071	8.633167	9.1660259	2.2896839	18.71817	Up
MUC3A	-0.94945	4.992359	-8.63125	9.2424685	2.3060822	18.71	Down
EBF1	2.013707	9.3019	8.641936	9.2997507	2.3176671	18.70659	Up
NPPA	-1.42	5.283475	-8.6294	9.3165690	2.3191524	18.70213	Down
ALDH1L1	-2.07376	7.208825	-8.63651	9.3767281	2.3314104	18.6977	Down
DPT	1.616753	9.174499	8.631676	9.4626169	2.3500299	18.68812	Up
RNF135	1.186347	7.86708	8.618178	9.7797375	2.4259655	18.65436	Up
SP100	1.04977	7.922347	8.61191	1.0048433	2.4897267	18.62768	Up
AIF1	2.161275	7.54493	8.616945	1.0357032	2.5632154	18.60057	Up
CTR9	-0.56442	8.634427	-8.60276	1.0453990	2.5842168	18.58872	Down
PLAUR	1.673173	5.522665	8.597412	1.0698572	2.6416196	18.56595	Up
VASH2	3.814475	6.056956	8.619725	1.0711078	2.6416537	18.56983	Up
SLC20A2	-1.12374	7.194575	-8.59273	1.0917235	2.6893922	18.54603	Down
SEPTIN11	1.460931	8.046868	8.590207	1.1037195	2.7158112	18.53527	Up
SLC47A1	-3.37089	6.32047	-8.60991	1.1149623	2.7403183	18.53021	Down
VWA5A	1.752541	6.890861	8.589243	1.1249129	2.7615966	18.51731	Up
GASK1B	1.924519	9.497348	8.597073	1.1282951	2.7667197	18.51626	Up
CTSO	1.29268	7.468732	8.584557	1.1310177	2.7702153	18.51122	Up
FANCL	0.832429	7.849725	8.583251	1.1374252	2.7827181	18.50566	Up
PRRC2A	-0.99238	6.911821	-8.58252	1.1410344	2.7883540	18.50254	Down
F13A1	2.383642	8.306738	8.593012	1.1482155	2.8026956	18.49903	Up
JAZF1	1.408259	7.091212	8.577816	1.1644730	2.8391342	18.48252	Up

ANXA1	1.776021	10.19946	8.58677	1.1789679	2.8711969	18.47298	Up
MAN1C1	1.303943	7.820557	8.571459	1.1969332	2.9116286	18.45545	Up
AMIGO1	-1.80704	6.309827	-8.57158	1.2089399	2.9374903	18.44617	Down
PGAP4	-1.48563	5.684533	-8.56744	1.2179371	2.9559888	18.43832	Down
SNTB2	1.039131	8.8838	8.564974	1.2309804	2.9842542	18.42783	Up
ITGB2	2.047159	7.297333	8.572066	1.2400490	3.0028306	18.42258	Up
BTN3A3	1.268833	8.5922	8.562841	1.2423882	3.0050880	18.41875	Up
DHCR7	-1.07536	5.792482	-8.56202	1.2467850	3.0123117	18.41527	Down
ADAP2	1.933307	6.839336	8.567414	1.2554930	3.0299231	18.41	Up
GPNMB	1.83328	10.0213	8.569045	1.2731487	3.0690643	18.39734	Up
LONRF2	-2.56583	6.105471	-8.56814	1.2781507	3.0776485	18.39348	Down
RPUSD3	-1.38204	7.773665	-8.55569	1.2814186	3.0820426	18.3883	Down
LINC0196	-1.01164	6.538947	-8.55477	1.2865269	3.0908484	18.38438	Down
CISD3	-1.04433	7.389908	-8.55347	1.2937603	3.1047341	18.37886	Down
SEC23B	1.41126	6.216357	8.552954	1.2966623	3.1082059	18.37666	Up
AGPAT3	-1.1999	8.297293	-8.54827	1.3231735	3.1641333	18.35673	Down
FAP	1.75428	7.406364	8.553793	1.3244382	3.1641333	18.35708	Up
KCTD12	1.713016	10.71007	8.559594	1.3260771	3.1645089	18.35724	Up
TXNRD2	-1.05935	5.668779	-8.54538	1.3398555	3.1938208	18.3444	Down
COX6A2	-1.06468	12.18327	-8.54407	1.3474637	3.2083758	18.33882	Down
CDIP1	-1.12651	6.8168	-8.54141	1.3630746	3.2419319	18.32748	Down
PGM2	1.360337	6.196215	8.541124	1.3647397	3.2422815	18.32628	Up
H2BC12	1.011443	7.445216	8.540567	1.3680261	3.2464780	18.32391	Up
ITGAM	1.928041	6.627649	8.543662	1.3836621	3.2783416	18.31402	Up
PCBD2	-1.35477	7.578419	-8.53768	1.3852298	3.2783416	18.31161	Down
C11orf68	-0.71762	7.852492	-8.53754	1.3860579	3.2783416	18.31102	Down
IFI16	1.824872	9.338596	8.546938	1.3891140	3.2819189	18.31109	Up
LINC0221	-0.93568	4.861101	-8.53678	1.3906436	3.2819189	18.30777	Down
CD302	1.954446	8.400808	8.546802	1.3985261	3.2968786	18.30476	Up
DNAAF9	-1.13317	8.996175	-8.53395	1.4077698	3.3150108	18.29572	Down
SLC43A3	1.081794	7.313864	8.530102	1.4313717	3.3668764	18.27935	Up
SIGLEC1	1.293849	6.234401	8.528342	1.4423131	3.3884262	18.27185	Up
RND3	1.618721	8.779383	8.533824	1.4437062	3.3884262	18.27219	Up
FAT4	1.552607	6.718746	8.525109	1.4626252	3.4290614	18.25808	Up
MKNK2	-0.87334	12.25786	-8.52305	1.4757224	3.4559737	18.24931	Down
TCEAL9	1.713376	8.978633	8.529497	1.4790250	3.4599142	18.24869	Up
SGMS1	1.022836	7.488816	8.517547	1.5112581	3.5314499	18.22588	Up
TMSB4X	1.014781	12.6579	8.516688	1.5168815	3.5407165	18.22223	Up
CTHRC1	2.364774	8.354931	8.527345	1.5238214	3.5530325	18.22039	Up
TBC1D8	-1.42291	9.028807	-8.51604	1.5297271	3.5629129	18.21422	Down
SPTLC1	0.955577	8.663012	8.51412	1.5338218	3.5685584	18.21129	Up
SART1	-0.65147	7.460791	-8.51131	1.5525963	3.6083082	18.19932	Down
FBXO40	-1.67981	10.13201	-8.51739	1.5583480	3.6177388	18.19725	Down
NRK	3.903397	5.650098	8.530652	1.5704825	3.6419506	18.19303	Up
TP53TG1	-0.95083	7.253746	-8.50736	1.5793591	3.6567970	18.18249	Down
JAM2	1.402985	8.077524	8.507218	1.5803088	3.6567970	18.1819	Up
ABCA8	1.495266	10.55737	8.513428	1.5852576	3.6642783	18.1804	Up
YWHAE	-1.16049	10.41482	-8.50204	1.6161316	3.7316042	18.15983	Down
PRKX	1.414603	5.098102	8.501035	1.6231424	3.7437448	18.15557	Up
R3HDM1	-0.7812	8.52161	-8.49784	1.6457205	3.7917259	18.14197	Down
HIF1A	1.541382	9.646467	8.502999	1.6492480	3.7957586	18.14115	Up
CD86	1.475063	5.913214	8.496631	1.6543641	3.8034348	18.13681	Up
MID1	1.840444	6.523758	8.498644	1.6718927	3.8396005	18.12745	Up
FSTL1	1.635362	10.91394	8.50122	1.6996063	3.8990539	18.11272	Up
CISD1	-0.95855	10.03758	-8.48666	1.7273031	3.9583412	18.09434	Down
SRM	-0.94519	8.658267	-8.48361	1.7502358	4.0065955	18.08136	Down
CIAO1	-0.81362	7.868651	-8.48072	1.7722689	4.0526896	18.06904	Down
RELB	-0.87829	4.133115	-8.47644	1.8053576	4.1239387	18.05083	Down

SINHCAF	1.046106	6.331686	8.473631	1.8274304	4.1698994	18.03887	Up
CD109	1.892815	6.901049	8.4797	1.8309717	4.1735213	18.03845	Up
LSP1P5	2.147784	7.554036	8.483314	1.8423143	4.1948986	18.03353	Up
STK25	-1.06595	8.678453	-8.46753	1.8762772	4.2676815	18.0129	Down
SELENOP	1.295515	12.28309	8.468804	1.9220277	4.3670924	17.99075	Up
MYOF	2.291986	8.288185	8.468463	1.9641203	4.4579896	17.9705	Up
LIPA	1.728119	8.999419	8.461035	1.9875849	4.5064585	17.95774	Up
SSR4	0.754998	9.919984	8.451418	2.0117464	4.5564029	17.94427	Up
SERP1	1.047936	8.579197	8.4481	2.0408274	4.6173721	17.93014	Up
STIMATE	-2.049	6.188588	-8.45384	2.0499210	4.6330385	17.92733	Down
RASSF2	1.806072	6.339277	8.444213	2.1026963	4.7472927	17.90144	Up
ZNF592	-1.32243	7.404717	-8.43968	2.1165842	4.7736014	17.89426	Down
SAMD14	-1.33793	5.073207	-8.4335	2.1738549	4.8975943	17.86798	Down
LINC0235:	-1.47427	6.663014	-8.43099	2.1976723	4.9460364	17.85726	Down
PPP5C	-1.09365	6.488552	-8.42789	2.2272733	5.0073793	17.84409	Down
ZNF567	1.152825	5.771112	8.42724	2.2335692	5.0162535	17.84131	Up
KANK2	1.433549	8.993347	8.427787	2.2496735	5.0471142	17.83475	Up
THAP7	-0.81259	7.291292	-8.42095	2.2951895	5.1421465	17.81452	Down
PRKCA	-1.24391	7.305259	-8.42078	2.2968479	5.1421465	17.81381	Down
PYGL	1.916839	6.688418	8.416987	2.3951077	5.3565143	17.77394	Up
CYBRD1	1.927647	9.903382	8.42161	2.4037697	5.3702630	17.77165	Up
NUP133	0.500122	7.792173	8.409768	2.4089541	5.3762218	17.7669	Up
RILPL1	-1.06457	9.596606	-8.40916	2.4152704	5.3846916	17.76432	Down
BTG1	1.175598	10.14228	8.408207	2.4252733	5.4013543	17.76025	Up
TUBA1B	1.02748	11.92175	8.399979	2.5131629	5.5854459	17.72521	Up
MYO18A	-1.19343	10.15756	-8.39925	2.5210551	5.5971617	17.72212	Down
MTMR14	-0.79326	7.860231	-8.39884	2.5255420	5.6013008	17.72037	Down
ST6GALN/	-1.85709	6.839657	-8.40277	2.5426068	5.6332983	17.71502	Down
CPVL	1.555289	7.694454	8.399165	2.5481043	5.6396283	17.71217	Up
PYCARD	2.410032	5.561647	8.40423	2.5707661	5.6838948	17.70511	Up
SEPTIN10	0.866232	9.875193	8.392035	2.6010513	5.7449073	17.69138	Up
KPNA2	1.310997	8.294653	8.39053	2.6180356	5.7764468	17.68497	Up
HYMAI	2.102093	6.802165	8.395611	2.6513430	5.8438992	17.67439	Up
EIF4E2	0.815963	8.470409	8.385407	2.6767095	5.8937280	17.66315	Up
CEP44	1.23572	6.723892	8.384322	2.6893046	5.9153623	17.65853	Up
ZNF268	1.467093	5.659602	8.382662	2.7086938	5.9518808	17.65146	Up
HERC5	1.495366	6.119194	8.377148	2.7740897	6.0893123	17.62798	Up
MIDEAS-/	-1.47288	5.89791	-8.37297	2.8246309	6.1938877	17.61021	Down
FXYD1	-1.02505	10.34809	-8.37185	2.8384450	6.2177958	17.6054	Down
PLSCR1	1.483267	8.361421	8.368562	2.9083936	6.3644950	17.58199	Up
BEX3	1.179519	9.700261	8.36308	2.9481804	6.4449578	17.56807	Up
RITA1	-1.19547	7.288158	-8.35899	3.0008376	6.5533629	17.55064	Down
FCGR2B	2.274623	6.755669	8.369354	3.0112365	6.5693554	17.54986	Up
PSME2	0.916663	9.356775	8.357354	3.0221275	6.5863877	17.54368	Up
FMR1	1.473664	8.128123	8.357404	3.0431835	6.6255161	17.53724	Up
MAP4K3-I	-0.6126	4.801457	-8.35445	3.0602861	6.6559664	17.53133	Down
RNASE4	1.912237	7.949672	8.362309	3.0735632	6.6773029	17.52915	Up
HLA-E	1.098964	10.59608	8.353244	3.0763489	6.6773029	17.52618	Up
NTM	1.658884	5.685758	8.34847	3.1405622	6.8097591	17.50585	Up
TNNI3	-1.44676	5.706952	-8.34775	3.1503927	6.8241468	17.50277	Down
MIER2	-0.74397	5.885636	-8.34188	3.2313311	6.9923779	17.4778	Down
REXO1	-1.05915	4.6161	-8.33517	3.3265498	7.1841989	17.44922	Down
TRIM3	-1.10055	5.207329	-8.33516	3.3267032	7.1841989	17.44917	Down
EIF3J	-0.76024	10.26692	-8.33062	3.3926627	7.3192414	17.42985	Down
NCF4	1.569329	4.902097	8.328616	3.4222593	7.3756422	17.4213	Up
CSF1R	2.086967	7.898405	8.337954	3.4478194	7.4232387	17.41659	Up
BUD31	0.611934	8.21251	8.325228	3.4727936	7.4694790	17.40687	Up
TIMP1	2.36919	9.90155	8.337043	3.5384797	7.5963919	17.39225	Up

TXNIP	0.940746	12.48731	8.320869	3.5389127	7.5963919	17.38831	Up
CAPZA1	1.045361	9.530652	8.318504	3.5753072	7.6668083	17.37824	Up
LAPTM4A	0.749475	11.74662	8.311728	3.6816894	7.8870133	17.34938	Up
PPIC	0.807348	10.16491	8.311065	3.6922697	7.9017532	17.34656	Up
ZFPL1	-0.97998	6.213284	-8.30869	3.7304645	7.9724315	17.33643	Down
DCAF5	-1.04754	9.339444	-8.30854	3.7327612	7.9724315	17.33582	Down
DSE	1.116894	7.513502	8.306929	3.7589263	8.0202945	17.32895	Up
HYAL1	-1.9017	5.374019	-8.30674	3.7993576	8.0984711	17.31896	Down
SCMH1	-1.06417	7.465221	-8.30254	3.8309803	8.1531347	17.31026	Down
PTBP3	1.553334	8.11352	8.306207	3.8326375	8.1531347	17.31073	Up
SH3PXD2E	1.300432	6.760286	8.299811	3.8764986	8.2382345	17.29863	Up
RAMP3	1.124226	6.365893	8.299457	3.8824296	8.2426373	17.29713	Up
ZNF329	1.674249	5.340078	8.298811	3.8933077	8.2575240	17.29437	Up
RNF126P1	-0.87181	5.790929	-8.29465	3.9640366	8.3991958	17.27665	Down
ZNF76	-1.4128	6.676518	-8.29188	4.0118891	8.4921634	17.26484	Down
CASP4LP	2.820819	6.761734	8.305077	4.0302469	8.5225756	17.26374	Up
ST3GAL1	-1.39423	7.321207	-8.29052	4.0354391	8.5251147	17.25908	Down
NKAPL	-1.75239	4.311039	-8.28995	4.0455453	8.5380195	17.25662	Down
SYTL2	2.36652	8.214075	8.299557	4.0829191	8.6083896	17.25037	Up
FHOD1	-1.56794	8.178632	-8.29108	4.1003094	8.6317075	17.2444	Down
TRAM1L1	1.425932	5.135121	8.286739	4.1020615	8.6317075	17.24297	Up
CD4	1.861052	6.204932	8.287499	4.1643171	8.7540832	17.22916	Up
ZNF839	-1.22486	6.752062	-8.28262	4.1757859	8.7654000	17.22544	Down
PLP2	1.248551	9.039352	8.282504	4.1779086	8.7654000	17.22494	Up
MYORG	-1.7811	7.337158	-8.28219	4.3019895	9.0168687	17.19768	Down
TMEM129	-0.82909	7.381885	-8.27151	4.3815115	9.1745414	17.17811	Down
ZNF503	1.517539	7.101124	8.270472	4.4011638	9.2066658	17.1737	Up
AKR1B10	-2.16319	6.455782	-8.27674	4.4360679	9.2706007	17.16787	Down
DPY19L3	1.585577	6.576527	8.266293	4.4814735	9.3563354	17.15591	Up
PLEKHJ1	-0.85029	7.800341	-8.26541	4.4985270	9.3827676	17.15217	Down
STEAP4	1.304866	9.21116	8.264937	4.5078401	9.3930196	17.15013	Up
ZNF526	-1.08833	5.009394	-8.26447	4.5168790	9.4026805	17.14816	Down
ALKBH5	-0.75285	10.71742	-8.26385	4.5290284	9.4187914	17.14552	Down
UROS	-0.93076	8.872708	-8.2632	4.5417777	9.4361177	17.14275	Down
PPIL2	-0.87061	5.642667	-8.26278	4.5501286	9.4442806	17.14094	Down
KLHL34	-3.32921	7.942669	-8.28034	4.6059802	9.5509247	17.13381	Down
GTF2I	0.528575	10.97662	8.257961	4.6459691	9.6245013	17.12043	Up
CHMP5	0.833728	9.556499	8.254759	4.7107790	9.7493041	17.10679	Up
COL4A1	1.45762	10.66598	8.258405	4.7675041	9.8571493	17.09655	Up
DOCK9	0.916525	8.740703	8.250583	4.7966664	9.9078531	17.08901	Up
USP2	-1.35539	8.045397	-8.24915	4.8266004	9.9600513	17.08289	Down
ENO1	0.98067	9.748203	8.247662	4.8576629	1.0014475	17.07658	Up
CAPRIN2	1.116115	7.7808	8.246602	4.8800047	1.0047372	17.07206	Up
UBE3C	-0.84603	8.446384	-8.24646	4.8830284	1.0047372	17.07145	Down
S100A4	1.743655	11.3357	8.25626	4.9037741	1.0080347	17.06987	Up
VWA8	-1.44697	9.324295	-8.24872	4.9119568	1.0087459	17.06651	Down
CYBC1	1.207248	6.488815	8.243215	4.9520411	1.0160009	17.05764	Up
CTSC	1.458594	8.755302	8.245506	4.9637482	1.0174254	17.056	Up
GPD1L	-1.32845	10.7285	-8.24509	5.0017940	1.0242408	17.04881	Down
MARCHF9	-0.80052	6.126811	-8.2403	5.0147844	1.0259173	17.04525	Down
PSTPIP2	-2.16348	6.430832	-8.24773	5.0278624	1.0276084	17.04462	Down
PNPLA4	-1.4285	7.681195	-8.23648	5.0984447	1.0410380	17.02897	Down
RAVER2	-1.01956	6.757292	-8.23579	5.1137521	1.0431663	17.02602	Down
DNM3OS	2.563444	6.551468	8.245847	5.1289610	1.0452705	17.02568	Up
ARAF	-1.53582	7.999315	-8.23701	5.1622409	1.0510499	17.01755	Down
SLC2A13	1.600653	5.960638	8.23307	5.1742308	1.0518119	17.01444	Up
TMEM108	-1.86677	8.454118	-8.243	5.1758325	1.0518119	17.01655	Down
GPS1	-0.94894	7.336745	-8.23249	5.1873301	1.0531463	17.01196	Down

ELK3	1.641711	8.985098	8.238021	5.2061210	1.0559575	17.00994	Up
PPP2R1A	-0.87927	8.857291	-8.2311	5.2186129	1.0574870	17.00604	Down
POLL	-1.10163	5.579772	-8.23048	5.2325717	1.0593105	17.00341	Down
CDH11	2.372834	7.653281	8.239837	5.2636210	1.0645873	17.00018	Up
FBXO44	-1.1639	4.835549	-8.22851	5.2773740	1.0663590	16.99502	Down
MAF1	-1.09597	8.983455	-8.22727	5.3057157	1.0710725	16.98975	Down
TIMM17B	-1.15828	7.282106	-8.2262	5.3303721	1.0750338	16.98519	Down
SUOX	-1.10615	8.333063	-8.22529	5.3513277	1.0782420	16.98133	Down
RARRES2	1.964115	7.025839	8.228733	5.4191534	1.0908791	16.97047	Up
PKD1	-0.89007	6.939428	-8.2206	5.4611425	1.0982964	16.96133	Down
NDUFAF4	-0.92144	8.875979	-8.21769	5.5301978	1.1111379	16.94897	Down
TBXA2R	-1.10273	5.350145	-8.2125	5.6558080	1.1353077	16.92687	Down
TASP1	1.041773	6.186394	8.207532	5.7787051	1.1588881	16.90571	Up
HLA-DRB1	1.890558	10.77894	8.215378	5.8490721	1.1718994	16.89638	Up
GPR157	-2.30621	8.181067	-8.21413	5.8806480	1.1771216	16.89108	Down
TBC1D9	1.188533	7.555462	8.200568	5.9554470	1.1909778	16.87606	Up
SLC27A4	-1.13742	5.380295	-8.20031	5.9621013	1.1911932	16.87497	Down
GJA1	1.714425	9.584216	8.207952	5.9694356	1.1915439	16.87568	Up
ARHGEF1(	-0.88802	7.863737	-8.19566	6.0831763	1.2131137	16.85518	Down
LRRFIP2	-0.7991	9.037189	-8.19389	6.1299027	1.2212916	16.84765	Down
SPRYD7	-1.07204	6.577235	-8.18975	6.2409061	1.2422485	16.82999	Down
ZNF768	-1.01691	6.641875	-8.189	6.2609916	1.2450862	16.82683	Down
MYO1B	1.192295	8.260041	8.188183	6.2832399	1.2483482	16.82334	Up
ZNF219	-0.90474	5.623056	-8.18551	6.3563411	1.2616982	16.81196	Down
S100A16	1.343549	9.088511	8.183634	6.4301204	1.2751579	16.80079	Up
EIF4ENIF1	-0.68611	7.317759	-8.18234	6.4440032	1.2767256	16.79848	Down
SLC12A4	-0.77897	5.483203	-8.18137	6.4711320	1.2809123	16.79434	Down
ITIH5	1.197161	8.115765	8.171799	6.7447563	1.3338380	16.75359	Up
TAF4	-0.66274	7.472035	-8.17151	6.7533191	1.3342959	16.75234	Down
NHSL2	1.389088	6.34399	8.171176	6.7629634	1.3349664	16.75094	Up
TRAM1	0.944253	10.18695	8.168484	6.8421740	1.3493550	16.73948	Up
RALY	-0.82202	7.998641	-8.16611	6.9127310	1.3620120	16.72938	Down
RAB34	1.277415	7.395951	8.164104	6.9730441	1.3726292	16.72083	Up
FZD1	1.326361	6.910469	8.163044	7.0051155	1.3776727	16.71632	Up
WIZ	-0.86082	6.415891	-8.16168	7.0466859	1.3845733	16.7105	Down
NRG4	-3.37974	1.9927	-8.16529	7.0609803	1.3861067	16.7095	Down
PRKD1	1.054001	5.79507	8.160557	7.0808824	1.3887372	16.70573	Up
UCP2	1.631855	10.43121	8.169658	7.0986170	1.3909381	16.70564	Up
EIF4E3	-1.02521	8.826443	-8.15818	7.1539216	1.4002096	16.69564	Down
SDAD1	-0.87424	7.71994	-8.15802	7.1590456	1.4002096	16.69493	Down
SYNGR1	-1.04912	7.322434	-8.15511	7.2498461	1.4166716	16.68253	Down
NPC2	1.340088	9.750968	8.156119	7.2866357	1.4225591	16.67808	Up
PARVA	1.439365	7.397487	8.152031	7.3469220	1.4330188	16.66944	Up
MYL6	1.150028	11.71313	8.1542	7.3700980	1.4362276	16.66705	Up
TGFBI	1.384823	9.860941	8.153588	7.4094421	1.4425785	16.66197	Up
FBXO31	-1.03163	7.967549	-8.14821	7.4695164	1.4529502	16.65316	Down
PABIR1	-1.2396	7.893178	-8.14428	7.5975764	1.4752118	16.63643	Down
CCR3	-2.44628	3.17253	-8.14778	7.5977760	1.4752118	16.63727	Down
RXRB	-0.737	7.115422	-8.14315	7.6345929	1.4810139	16.63165	Down
DTNA	0.712026	9.920156	8.141641	7.6846628	1.4893741	16.62521	Up
SPIRE1	-0.91308	8.477761	-8.14056	7.7207063	1.4950031	16.62061	Down
PLK2	1.552485	7.597981	8.142415	7.7587991	1.5010184	16.6165	Up
FCER1A	2.517491	6.866211	8.145488	7.9060422	1.5281199	16.59983	Up
RGN	-1.41087	5.88643	-8.13314	7.9725393	1.5395795	16.58903	Down
RPUSD1	-0.8221	5.219161	-8.13114	8.0418489	1.5515610	16.58051	Down
TSHZ2	2.437614	5.932092	8.141078	8.0577725	1.5532302	16.58113	Up
REV3L	1.280223	8.335208	8.127566	8.1670481	1.5719372	16.56531	Up
NEK10	-2.42921	3.560879	-8.13366	8.1695402	1.5719372	16.56653	Down

RNASET2	1.468423	8.31121	8.126385	8.3064874	1.5968493	16.54933	Up
IFI30	2.394267	7.692126	8.13393	8.3376688	1.6014022	16.54772	Up
NAB1	1.04757	7.718894	8.120117	8.4344789	1.6185409	16.5336	Up
MFF	-0.66259	9.694681	-8.11924	8.4664030	1.6232085	16.52989	Down
KCNA5	-1.34915	4.985225	-8.11759	8.5273243	1.6334223	16.52283	Down
HLA-DRB5	1.754014	10.38838	8.127582	8.5405189	1.6344839	16.52387	Up
SQSTM1	-0.83868	10.8198	-8.11665	8.5619828	1.6371247	16.51884	Down
A2M	0.954686	11.47421	8.116208	8.5783026	1.6387780	16.51697	Up
PTPN23	-0.95527	6.560862	-8.11554	8.6031604	1.6420358	16.51412	Down
DCUN1D2	-1.49118	7.892961	-8.11777	8.6133456	1.6420358	16.51358	Down
LGI1	-4.47983	3.511207	-8.1346	8.6184203	1.6420358	16.51719	Down
TSPYL4	1.058688	7.784787	8.114201	8.6531032	1.6471745	16.50843	Up
BDH1	-1.63804	6.888505	-8.11525	8.7158348	1.6576384	16.50199	Down
PPP1R14A	-1.63522	5.838694	-8.11088	8.7783863	1.6680495	16.49428	Down
CD53	1.755941	7.202644	8.114777	8.8504860	1.6802549	16.48767	Up
SELENOF	0.839439	9.519488	8.108405	8.8727704	1.6829896	16.48376	Up
DCAF8	-1.15012	8.414232	-8.10529	8.9931704	1.7043135	16.4705	Down
TUBB6	2.585067	10.09262	8.122511	9.0779856	1.7188618	16.46606	Up
CAPN6	3.880809	8.315526	8.116002	9.3355786	1.7660698	16.43853	Up
RNF19B	-1.53466	5.826493	-8.09049	9.5876106	1.8121432	16.40752	Down
IL17RC	-0.89268	6.030427	-8.08914	9.6436597	1.8211254	16.40178	Down
MPND	-1.38629	5.986352	-8.08824	9.6812313	1.8266054	16.39796	Down
EFEMP1	2.065364	9.6207	8.096818	9.7517796	1.8382922	16.39337	Up
MTSS2	-1.68175	5.640032	-8.08372	9.8726545	1.8585629	16.37869	Down
EMP2	0.787558	8.337227	8.083622	9.8767158	1.8585629	16.37829	Up
FN1	1.610139	10.84789	8.088335	1.0115000	1.9007438	16.35738	Up
ALS2CL	-0.81156	6.359663	-8.07803	1.0118670	1.9007438	16.35448	Down
GZMA	1.952677	5.139317	8.081147	1.0135631	1.9022568	16.35371	Up
MYBBP1A	-1.07961	6.20047	-8.07743	1.0144950	1.9023340	16.35193	Down
PDXP	-1.11529	5.77626	-8.0771	1.0159406	1.9033736	16.35052	Down
SLC17A9	-0.65634	4.718566	-8.0758	1.0216347	1.9123640	16.34503	Down
CBX4	1.38996	7.399025	8.074926	1.0255236	1.9179627	16.34129	Up
GIMAP7	1.602024	7.340489	8.074067	1.0460876	1.9547103	16.32269	Up
SEMA6C	-1.44402	6.833231	-8.06571	1.0672058	1.9924285	16.30209	Down
LOC40049	-1.01887	4.743122	-8.06252	1.0820591	2.0183946	16.28849	Down
SPTBN4	-1.06305	5.348026	-8.06089	1.0896876	2.0307799	16.28158	Down
KIF13B	1.157141	6.734853	8.0607	1.0906005	2.0307799	16.28075	Up
MAP2K7	-1.0212	7.584354	-8.05802	1.1033064	2.0526497	16.26936	Down
RABEP1	-0.93534	9.464771	-8.05631	1.1114818	2.0660600	16.26209	Down
ZNF672	-1.09255	7.280912	-8.05513	1.1171765	2.0748397	16.25707	Down
GRK4	-0.96605	4.789137	-8.05397	1.1228053	2.0834818	16.25212	Down
CASP2	0.815266	7.095925	8.052215	1.1313634	2.0975400	16.24465	Up
USP21	-0.89384	7.219009	-8.04848	1.1497992	2.1288450	16.22875	Down
ANKRD13	-0.98154	5.302415	-8.04839	1.1502421	2.1288450	16.22837	Down
ABCD4	1.044846	6.721115	8.041475	1.1851449	2.1915433	16.19896	Up
RAB11B	-1.24552	6.340955	-8.03756	1.2054077	2.2270845	16.18228	Down
CAMK2B	-1.27566	8.020129	-8.03725	1.2070181	2.2281325	16.18097	Down
SLC35B4	0.867509	5.892156	8.036998	1.2083172	2.2284569	16.17991	Up
SMG5	-0.84937	6.783269	-8.03681	1.2092806	2.2284569	16.17913	Down
ARPIN	0.639337	7.2773	8.035017	1.2187110	2.2438991	16.17148	Up
DHX15	0.539556	9.772855	8.034698	1.2203919	2.2450587	16.17013	Up
CORO6	-0.81066	10.67269	-8.03382	1.2250133	2.2516209	16.16641	Down
APBB1IP	1.862677	6.488046	8.038096	1.2285469	2.2561459	16.16482	Up
IL33	1.930182	6.808245	8.038923	1.2296992	2.2561459	16.16416	Up
MYADML2	-1.6474	6.529337	-8.03392	1.2354298	2.2619774	16.1586	Down
NPM1	0.638491	11.88187	8.03177	1.2359433	2.2619774	16.15767	Up
SPATS2L	1.066454	10.37789	8.029795	1.2465432	2.2794239	16.14927	Up
POLR2G	0.589335	8.946379	8.029416	1.2485866	2.2812073	16.14766	Up

TXN	0.944768	9.838481	8.028327	1.2544813	2.2900182	16.14302	Up
SOCS2	1.523732	7.12148	8.028898	1.2576784	2.2938938	16.14081	Up
RPS7	0.536371	12.54394	8.02595	1.2674406	2.3061443	16.13291	Up
GLMP	1.472738	5.870171	8.025925	1.2675762	2.3061443	16.13281	Up
ST8SIA1	1.712833	4.252733	8.025915	1.2676343	2.3061443	16.13276	Up
GGTA1	1.706997	8.231791	8.027452	1.2921095	2.3484059	16.11545	Up
TRIM14	2.343979	4.783287	8.02833	1.2930636	2.3484059	16.11499	Up
OCIAD2	1.028601	5.871432	8.02017	1.2995199	2.3581263	16.10832	Up
MSR1	2.65438	5.181307	8.029093	1.3057683	2.3674533	16.10614	Up
TEP1	1.187219	7.189694	8.017075	1.3170274	2.3846478	16.09516	Up
HLA-DMA	1.712743	7.995434	8.022945	1.3174849	2.3846478	16.09632	Up
PABIR2	1.156419	6.828159	8.013681	1.3364951	2.4170078	16.08072	Up
METTL26	-1.07899	7.526491	-8.01155	1.3488528	2.4372927	16.07167	Down
MTFMT	-1.03589	6.973988	-8.01109	1.3515398	2.4391956	16.06971	Down
SLC25A39	-1.02629	6.959643	-8.01098	1.3521901	2.4391956	16.06924	Down
GOLGA2	-0.70005	8.059534	-8.01034	1.3559503	2.4439145	16.0665	Down
FHOD3	-1.42793	8.431692	-8.01051	1.3686927	2.4648010	16.05789	Down
KCNQ4	1.942504	7.172309	8.014215	1.3770735	2.4778043	16.05318	Up
PIGG	0.906859	7.416866	8.003714	1.3953526	2.5085810	16.03832	Up
RALGPS2	1.39524	6.257742	8.00309	1.3991211	2.5132405	16.03567	Up
PTP4A1	-1.31228	11.03523	-8.00367	1.4250913	2.5577395	16.01881	Down
KLK15	-1.0634	4.606801	-7.99825	1.4287052	2.5620727	16.01509	Down
SAMSN1	2.961287	5.119269	8.007169	1.4397574	2.5797264	16.01022	Up
SRPK2	0.907934	7.672273	7.993691	1.4571501	2.6087018	15.99569	Up
MRPS34	-0.97285	7.518811	-7.98628	1.5045669	2.6913351	15.96419	Down
UBA1	-0.73166	9.969111	-7.98522	1.5115205	2.7015109	15.95966	Down
LSM10	-1.04877	9.170994	-7.98497	1.5131636	2.7021864	15.95859	Down
HLA-DRB4	1.643559	10.71435	7.991111	1.5379982	2.7442411	15.94509	Up
ABCC1	-1.07484	8.098525	-7.98055	1.5423399	2.7496909	15.9398	Down
TMEM50A	0.670026	9.723399	7.980265	1.5442292	2.7507630	15.93859	Up
ZNF738	1.797233	5.830307	7.978121	1.5762639	2.8054870	15.91906	Up
MX2	1.642456	6.150124	7.974543	1.5878986	2.8238417	15.91135	Up
PLTP	1.695661	7.716072	7.977761	1.6011193	2.8449840	15.9045	Up
MAD2L1	1.186744	5.519932	7.965287	1.6475260	2.9250092	15.8749	Up
FNBP1L	-0.83516	7.614156	-7.96365	1.6592124	2.9433106	15.86795	Down
P3H1	1.203464	5.933486	7.961509	1.6746566	2.9682420	15.85883	Up
RPS8	0.646371	12.71542	7.96005	1.6852482	2.9845383	15.85263	Up
EIF4G3	-1.16971	8.297096	-7.95815	1.6991504	3.0066657	15.84455	Down
SMOC1	-1.81738	5.013894	-7.95768	1.7109101	3.0247750	15.83805	Down
PDCD4	1.325828	8.140206	7.956347	1.7124440	3.0247750	15.83688	Up
TMF1	0.670014	7.889727	7.956186	1.7136332	3.0247750	15.8362	Up
STIP1	-1.02147	8.215739	-7.95442	1.7267326	3.0453802	15.82871	Down
FAM20B	-0.76006	8.233061	-7.95202	1.7447456	3.0746103	15.8185	Down
SPR	-1.25306	6.625197	-7.94839	1.7723586	3.1206953	15.80306	Down
CDADC1	0.736916	6.938953	7.944931	1.7990620	3.1651044	15.78835	Up
DTL	1.468267	4.684506	7.942031	1.8217580	3.2023956	15.77601	Up
IFRD2	-1.05818	8.522939	-7.94098	1.8300834	3.2143851	15.77153	Down
ARMC6	-0.82186	5.918676	-7.93976	1.8397591	3.2287244	15.76634	Down
SLC2A8	-1.08855	7.205607	-7.93944	1.8423034	3.2305351	15.76498	Down
PRKACB	0.859952	8.763653	7.938698	1.8481938	3.2382053	15.76184	Up
FLCN	-1.00218	6.225668	-7.9347	1.8804084	3.2919478	15.74484	Down
PLPPR2	-1.02488	6.452413	-7.93163	1.9054869	3.3331196	15.73181	Down
WDFY2	1.138269	6.634086	7.930265	1.9168007	3.3501662	15.72599	Up
HSF1	-0.98999	8.906437	-7.92915	1.9260255	3.3635366	15.72127	Down
TMTC4	-1.06829	6.786835	-7.92858	1.9308410	3.3691913	15.71881	Down
TWSG1	1.124045	8.436097	7.927522	1.9396550	3.3818083	15.71433	Up
HLA-DRA	2.153677	10.68264	7.940902	1.9477534	3.3931581	15.71392	Up
RAI14	1.877116	5.934916	7.928798	1.9652296	3.4180272	15.70256	Up

EGLN1	-0.80081	10.99401	-7.92409	1.9686690	3.4212231	15.69973	Down
MAPKAPK	-1.23757	9.18511	-7.92378	1.9712692	3.4229568	15.69843	Down
CTSS	1.63528	7.401748	7.928299	1.9729883	3.4231588	15.69879	Up
PDZD7	-0.99592	6.642939	-7.92274	1.9801583	3.4314946	15.694	Down
CHCHD3	-0.70622	10.6633	-7.92264	1.9810061	3.4314946	15.69358	Down
RPL17	0.632968	12.79383	7.921952	1.9869196	3.4389489	15.69065	Up
GYS1	-1.08044	10.6621	-7.92082	1.9966842	3.4530512	15.68583	Down
MUL1	-1.05303	6.535626	-7.91499	2.0476123	3.5344131	15.66105	Down
RNF157	-2.29791	7.180709	-7.92461	2.0483234	3.5344131	15.66322	Down
WNT10B	-1.07049	4.524257	-7.91487	2.0486953	3.5344131	15.66053	Down
FAR2	1.206853	4.990806	7.909836	2.0937241	3.6091816	15.63915	Up
RIN2	0.943463	8.837104	7.905583	2.1325643	3.6702102	15.62107	Up
VDAC1	-0.86206	11.94841	-7.9045	2.1425465	3.6844210	15.61648	Down
CXorf38	1.07723	5.787436	7.902922	2.1572281	3.7066837	15.60976	Up
ENAM	-1.58027	5.547644	-7.90249	2.1612263	3.7105685	15.60794	Down
RGS9BP	-2.6512	4.091117	-7.91081	2.1737656	3.7277110	15.60475	Down
AQP7	-1.50355	6.282191	-7.90093	2.1758370	3.7277110	15.60131	Down
TMEM19	-0.7721	8.277658	-7.90071	2.1779583	3.7277110	15.60035	Down
TAP1	0.962631	7.101171	7.900684	2.1781924	3.7277110	15.60025	Up
BBOF1	0.992624	5.776859	7.900407	2.1807982	3.7291824	15.59907	Up
VPS37A	-0.72466	7.945706	-7.89903	2.1937702	3.7458139	15.59324	Down
CNOT11	-0.69824	7.809535	-7.89889	2.1951345	3.7458139	15.59263	Down
YIPF5	0.914472	8.890846	7.898822	2.1957857	3.7458139	15.59234	Up
IFFO1	1.282487	8.097481	7.897613	2.2072899	3.7624340	15.5872	Up
CARD6	1.21719	6.351985	7.895279	2.2296678	3.7975475	15.57727	Up
ADSS2	0.932908	6.659883	7.894891	2.2334069	3.7988794	15.57563	Up
RAB30	1.353965	7.775117	7.894829	2.2340072	3.7988794	15.57536	Up
WDR37	-0.91359	8.129135	-7.89342	2.2476907	3.8191072	15.56936	Down
COPB2	0.670151	8.989933	7.89192	2.2622591	3.8408052	15.563	Up
FKBP4	-1.19801	7.642727	-7.89035	2.2776381	3.8638439	15.55634	Down
TMEM175	-0.95884	6.475175	-7.88954	2.2856467	3.8743526	15.55289	Down
KAZN	1.862294	6.149699	7.89398	2.2878943	3.8750869	15.55313	Up
ISLR	1.66724	7.847194	7.89217	2.3161951	3.9199125	15.54131	Up
CYFIP1	0.976437	9.339296	7.884178	2.3392193	3.9557439	15.5301	Up
MIEF2	-1.13412	6.64713	-7.88374	2.3436317	3.9600701	15.52824	Down
ZWILCH	0.925624	6.700342	7.881502	2.3664249	3.9954233	15.51873	Up
TMEM150	-1.10147	6.565999	-7.88059	2.3758106	4.0081014	15.51483	Down
MLLT6	-0.95883	8.685611	-7.87944	2.3875852	4.0247865	15.50997	Down
ACTR1B	-0.79759	8.823482	-7.87918	2.3903164	4.0262127	15.50885	Down
ALG13	0.731965	7.914237	7.878028	2.4022075	4.0430534	15.50396	Up
THAP11	-0.84485	8.433955	-7.87434	2.4407953	4.1047642	15.48829	Down
GFER	-1.18037	5.664505	-7.86843	2.5039421	4.2076471	15.46317	Down
CEP192	0.983134	5.828737	7.867363	2.5154937	4.2237355	15.45864	Up
RACGAP1	0.959852	6.625054	7.867033	2.5190859	4.2264444	15.45724	Up
STAT2	0.971104	8.170864	7.866327	2.5267717	4.2360119	15.45424	Up
INPP5J	-1.94101	4.530649	-7.86895	2.5332057	4.2434673	15.45258	Down
TWIST1	2.215846	5.455845	7.860683	2.6590429	4.4507710	15.40568	Up
FAM3A	-1.09335	5.762723	-7.84293	2.7955534	4.6756014	15.35483	Down
HSD11B1L	-0.96373	5.071126	-7.84074	2.8220765	4.7162684	15.34554	Down
KYNU	2.78304	4.1705	7.849399	2.8318906	4.7289694	15.34461	Up
MOCS1	-1.20508	5.987738	-7.83974	2.8342866	4.7292730	15.34129	Down
OR7E156F	-1.07062	5.149096	-7.83914	2.8416617	4.7378776	15.33874	Down
LOC1005C	-1.19921	5.752655	-7.83775	2.8587435	4.7626400	15.33284	Down
SLC25A38	-0.94576	7.38868	-7.83572	2.8839988	4.8009701	15.32419	Down
LPXN	1.17485	6.041228	7.835178	2.8907170	4.8046642	15.3219	Up
SELENOS	-0.95617	9.394568	-7.83273	2.9214662	4.8519965	15.3115	Down
TCAF1	1.241353	9.628345	7.832229	2.9348988	4.8705181	15.30714	Up
CHST15	1.235528	7.584748	7.83079	2.9460282	4.8851918	15.30326	Up

L1CAM	2.530535	5.123885	7.838606	2.9666000	4.9154883	15.2989	Up
MITF	-0.9583	9.846907	-7.82875	2.9720536	4.9207070	15.29462	Down
ADAT1	0.921088	5.809388	7.827693	2.9857027	4.9394763	15.29011	Up
CRLF3	0.964343	7.023314	7.824756	3.0238118	4.9986512	15.27764	Up
SFTPC	-0.96751	4.53504	-7.82438	3.0287633	5.0029643	15.27603	Down
MID1IP1	-1.40985	9.248295	-7.82511	3.0700881	5.0673062	15.26372	Down
ALDH18A:	1.121958	6.609718	7.817759	3.1165817	5.1400735	15.24792	Up
PMF1	-0.86094	7.937645	-7.81545	3.1478477	5.1876337	15.2381	Down
KIRREL1	0.974845	5.815155	7.814904	3.1552513	5.1923275	15.23579	Up
CLMP	1.953951	6.444351	7.820305	3.1575356	5.1923275	15.23655	Up
PSME1	0.791513	9.687525	7.814703	3.1579892	5.1923275	15.23494	Up
GMPR	-1.34216	9.756673	-7.81669	3.1759790	5.2178892	15.23023	Down
SLC35E4	-1.56471	5.016023	-7.81238	3.1898765	5.2366936	15.22506	Down
WBP4	-1.1353	8.212996	-7.81083	3.2112896	5.2677975	15.21848	Down
FUT7	-1	6.322487	-7.80966	3.2275264	5.2903692	15.21352	Down
MGAT2	0.990764	8.067636	7.80794	3.2515850	5.3257173	15.20622	Up
LGALS3BP	1.705797	8.729541	7.814329	3.2591165	5.3323535	15.20577	Up
ASPN	2.411574	9.989323	7.824809	3.2619766	5.3323535	15.20773	Up
FOXP1	0.988421	9.191932	7.80712	3.2631267	5.3323535	15.20273	Up
ITGBL1	1.941737	7.697027	7.814639	3.2890580	5.3706194	15.19742	Up
PRR12	-1.13401	6.337475	-7.8027	3.3260031	5.4267971	15.18396	Down
SLC22A23	-1.34373	5.356883	-7.79937	3.3741342	5.5011267	15.16983	Down
SMIM1	-1.64742	4.272968	-7.7982	3.3912684	5.5248445	15.16485	Down
ACAP3	-0.79948	6.343676	-7.79717	3.4063547	5.5451923	15.16049	Down
ZEB2	1.46149	8.016169	7.800037	3.4103315	5.5474380	15.16017	Up
NCOA7	1.161511	8.271207	7.795912	3.4249408	5.5627289	15.15514	Up
ALCAM	1.611785	6.895407	7.796548	3.4630372	5.6203304	15.14511	Up
N4BP2L2	1.199424	9.917848	7.792566	3.4747888	5.6351207	15.14093	Up
MAP3K5	1.186742	7.98832	7.79127	3.4942791	5.6624290	15.13543	Up
DTD2	0.86658	5.840373	7.790762	3.5019490	5.6690585	15.13327	Up
TRMT5	0.733929	10.85828	7.790648	3.5036788	5.6690585	15.13279	Up
ANKRD54	-0.84914	5.429487	-7.78896	3.5293676	5.7063007	15.1256	Down
C1QA	2.128966	8.0519	7.7965	3.5561851	5.7453102	15.12063	Up
FAM13A-/	1.30757	5.650572	7.785789	3.5779698	5.7761360	15.11215	Up
FLVCR1	1.321386	6.14122	7.783405	3.6149812	5.8313779	15.10203	Up
ECSIT	-1.19563	7.682113	-7.78323	3.6176494	5.8313779	15.10131	Down
LYSET	-0.86875	8.159842	-7.78207	3.6358235	5.8562532	15.09638	Down
RDH11	0.811605	8.050152	7.781896	3.6386139	5.8563313	15.09563	Up
RAPH1	1.554819	9.413219	7.783697	3.6972193	5.9461755	15.08138	Up
KHDRBS1	0.53125	10.11248	7.777259	3.7121907	5.9657614	15.07594	Up
KSR1	-0.76215	8.213845	-7.77634	3.7270027	5.9850619	15.07203	Down
ZNF177	1.641996	5.215405	7.775021	3.7482446	6.0146513	15.06644	Up
TMEM60	-0.6746	8.397227	-7.77265	3.7867948	6.0719493	15.05637	Down
TEX2	-0.98785	8.366693	-7.76957	3.8374570	6.1485677	15.04331	Down
DGCR8	-0.90094	6.545562	-7.76784	3.8663427	6.1902061	15.03593	Down
GNAI3	0.907227	8.830169	7.766788	3.8838591	6.2135927	15.03149	Up
GGT7	-1.17371	5.20975	-7.76593	3.8983160	6.2320534	15.02783	Down
HDGFL2	-0.80252	7.459202	-7.76364	3.9369612	6.2844259	15.01813	Down
ARID1A	-0.98392	8.55045	-7.76326	3.9435363	6.2902202	15.01649	Down
RNLS	-1.0401	6.17509	-7.75581	4.0722974	6.4907558	14.9849	Down
CAPG	2.286941	5.367518	7.763093	4.0949307	6.5219635	14.98174	Up
NPEPPS	-0.85123	9.498268	-7.75325	4.1176485	6.5483795	14.97401	Down
TMEM200	2.46262	5.261727	7.762002	4.1253934	6.5558149	14.97462	Up
ZNF282	-1.09835	5.736854	-7.75166	4.1459282	6.5835490	14.96728	Down
HIF1AN	-0.81908	7.760105	-7.7508	4.1613369	6.6010415	14.96363	Down
DOCK11	2.095325	6.914155	7.759886	4.1631252	6.6010415	14.96567	Up
QRSL1	-0.95175	8.548606	-7.74694	4.2313220	6.7041970	14.94723	Down
F11R	1.003525	8.189698	7.745868	4.2509156	6.7302488	14.94269	Up

ANKRD39	-0.88206	12.48097	-7.7448	4.2705248	6.7562866	14.93817	Down
NDUFS8	-1.10257	10.74694	-7.74454	4.2754480	6.7590688	14.93703	Down
HSPA1L	-1.03577	6.573408	-7.7436	4.2927245	6.7813617	14.93307	Down
HEATR5B	0.543826	7.161379	7.743075	4.3024755	6.7917422	14.93084	Up
FAM240C	-2.03374	5.926681	-7.74649	4.3401129	6.8460954	14.92373	Down
ERI3	-1.03565	7.321137	-7.73799	4.3979339	6.9321825	14.90926	Down
C1QTNF2	1.579861	5.617971	7.737719	4.4031049	6.9352150	14.90811	Up
GYG1	-0.67039	12.45559	-7.73302	4.4932699	7.0720162	14.88817	Down
OSMR	1.70403	7.543117	7.737752	4.5066189	7.0878031	14.88671	Up
ZNF740	-0.84199	5.711729	-7.73085	4.5356524	7.1282166	14.87894	Down
C18orf54	1.598851	4.59851	7.730124	4.5498156	7.1399677	14.87588	Up
C10orf71	-1.25115	10.14238	-7.72963	4.6041195	7.2198815	14.86482	Down
CAMTA2	-0.77722	7.269512	-7.72709	4.6097632	7.2234280	14.86301	Down
PTGES2	-1.33381	7.059916	-7.72623	4.6269333	7.2450178	14.85935	Down
AKR1A1	0.73566	8.307738	7.726052	4.6304741	7.2452503	14.8586	Up
ZNF316	-1.122	5.464879	-7.72443	4.6630604	7.2908963	14.8517	Down
APOBEC3(	1.476451	6.048213	7.72366	4.6785285	7.3097302	14.84845	Up
EEF1AKM1	1.24205	4.936496	7.72184	4.7154116	7.3619708	14.84073	Up
HDAC5	-1.056	7.418645	-7.7177	4.8004918	7.4893283	14.82315	Down
COPB1	0.582838	10.31843	7.716467	4.8260327	7.5236794	14.81793	Up
BCHE	1.950209	6.451095	7.721098	4.8417973	7.5427504	14.81617	Up
SLCO2B1	1.449531	6.38033	7.714868	4.8594347	7.5605252	14.81115	Up
NT5E	2.199564	7.409095	7.723901	4.8602870	7.5605252	14.81342	Up
REEP2	-1.17636	6.223063	-7.71372	4.8835455	7.5911765	14.80628	Down
SLC25A6	0.819318	10.36846	7.711787	4.9244685	7.6485905	14.79808	Up
PARD3	-1.21205	7.524313	-7.71164	4.9276432	7.6485905	14.79744	Down
IQGAP1	1.072587	9.285244	7.711374	4.9332659	7.6517569	14.79632	Up
RNF220	-0.81676	7.641217	-7.71058	4.9502486	7.6724424	14.79294	Down
GMFG	1.423286	6.434635	7.710412	4.9537869	7.6724424	14.79224	Up
TMEM59	0.556259	10.40485	7.709394	4.9755811	7.7006131	14.78792	Up
RAB31	1.534907	8.163283	7.713515	4.9814675	7.7035179	14.78795	Up
POLDIP2	-0.98407	9.000183	-7.70897	4.9846717	7.7035179	14.78613	Down
UBL4A	-0.93799	7.784789	-7.70638	5.0406613	7.7844140	14.77515	Down
ARPC3	0.895925	9.482537	7.703179	5.1108264	7.8836537	14.76156	Up
IFI44	1.983655	6.81771	7.711529	5.1123047	7.8836537	14.76355	Up
SMYD5	-0.79246	5.870362	-7.70202	5.1363429	7.9150081	14.75666	Down
TRIM22	1.250037	9.032217	7.701832	5.1406041	7.9158632	14.75584	Up
EIF4G1	-0.77167	9.729777	-7.70111	5.1565976	7.9347703	14.75279	Down
RP9P	-0.74245	7.770716	-7.69903	5.2030697	8.0005157	14.74397	Down
GRIN1	-1.09679	4.679545	-7.69665	5.2567648	8.0772649	14.73388	Down
RCBTB2	1.111326	7.948424	7.69535	5.2864030	8.1111348	14.72835	Up
FBXO32	-1.08587	11.0195	-7.69571	5.2904182	8.1114683	14.72775	Down
KCTD3	1.207496	7.916781	7.694852	5.2977713	8.1169153	14.72624	Up
TDO2	3.959297	5.231679	7.710861	5.3193034	8.1440632	14.72683	Up
GNG12	1.094851	8.67483	7.693471	5.3294312	8.1537243	14.72038	Up
ZDHHC5	-0.59765	8.523584	-7.69282	5.3443854	8.1707504	14.71762	Down
TGOLN2	0.57837	10.01672	7.691548	5.3738404	8.2099058	14.71222	Up
SLC25A30	-1.4563	8.944284	-7.69408	5.4166055	8.2693252	14.70561	Down
MANBA	0.949109	6.079131	7.689364	5.4247056	8.2757759	14.70296	Up
CLIC6	-1.55202	4.775784	-7.68688	5.4830563	8.3555312	14.69244	Down
AGPAT1	-1.11105	7.860995	-7.68681	5.4848088	8.3555312	14.69213	Down
TRMT2B	-1.4136	5.534604	-7.68624	5.4984237	8.3703017	14.68969	Down
ODF2	1.071551	4.609014	7.685404	5.5181706	8.3943795	14.68616	Up
GDAP1	1.03226	6.969073	7.678259	5.6909093	8.6448393	14.65586	Up
TPRA1	-0.83962	5.823821	-7.6768	5.7267137	8.6930457	14.64969	Down
FAM117A	1.304685	7.859835	7.675992	5.7468360	8.7173951	14.64625	Up
TRIM73	1.6892	5.936048	7.676936	5.7701675	8.7465747	14.64277	Up
MAPRE3	-0.948	7.453455	-7.67406	5.7949316	8.7746482	14.63805	Down

ANKRD13.	0.715083	8.561821	7.67393	5.7981699	8.7746482	14.6375	Up
PRNP	1.094393	10.05873	7.670578	5.8826187	8.8917885	14.62329	Up
FCER1G	1.734061	7.327733	7.672379	5.9720382	9.0205653	14.6099	Up
RCCD1	-1.03861	6.25805	-7.66321	6.0726138	9.1659990	14.59204	Down
EML4	1.323654	6.653268	7.660119	6.1540437	9.2823492	14.57894	Up
MEF2D	-1.26645	7.63279	-7.65695	6.2386467	9.4033180	14.56552	Down
DCXR	-1.41605	8.615701	-7.65861	6.2873383	9.4700263	14.55882	Down
LAYN	1.606527	6.135486	7.655967	6.2920449	9.4704366	14.55741	Up
PGAM5	-1.46083	4.265012	-7.65116	6.3965457	9.6209453	14.54095	Down
HPCAL1	0.653832	7.413242	7.650377	6.4181273	9.6466125	14.53764	Up
GSK3A	-0.77139	7.131515	-7.64992	6.4306897	9.6586970	14.53572	Down
THAP4	-0.95973	8.36644	-7.64968	6.4374794	9.6621002	14.53468	Down
STK4	0.848685	7.009439	7.64889	6.4594189	9.6882212	14.53133	Up
KLHL25	-0.83923	4.950291	-7.64766	6.4937738	9.7329137	14.52612	Down
SH3GLB2	-0.90741	6.309438	-7.64521	6.5627154	9.8293461	14.51574	Down
MFSD1	1.213094	8.850784	7.64263	6.6361556	9.9323764	14.5048	Up
RPS19	0.528884	12.60373	7.642203	6.6483785	9.9437023	14.50299	Up
LACC1	1.851718	5.461125	7.645191	6.6640534	9.9546807	14.50164	Up
TP53INP2	-1.26228	9.211761	-7.64261	6.6650404	9.9546807	14.5008	Down
NAA10	-0.87689	7.789884	-7.64016	6.7071693	1.0010602	14.49433	Down
MYCN	0.837859	5.951449	7.639122	6.7372919	1.0048539	14.48993	Up
ZNF625	1.109635	5.03565	7.636862	6.8032715	1.0139865	14.48035	Up
IL32	2.592044	10.49461	7.650595	6.8880781	1.0259105	14.47272	Up
P4HA2	1.084519	8.206792	7.632267	6.9393934	1.0328332	14.46087	Up
FEZ2	-1.20954	10.99853	-7.63467	6.9770948	1.0377213	14.45655	Down
PPT1	0.879379	9.999738	7.630133	7.0035594	1.0409326	14.45183	Up
MAP1LC3.	-1.05791	6.888216	-7.62974	7.0155446	1.0419888	14.45015	Down
KRT8P12	-0.78687	5.950567	-7.62946	7.0239734	1.0425158	14.44897	Down
ATP5F1D	-1.18506	10.50305	-7.63017	7.0583919	1.0468967	14.44467	Down
VPS35	0.636004	9.397219	7.626816	7.1044304	1.0529939	14.43777	Up
DAAM2	-1.33089	7.519763	-7.62628	7.1209230	1.0547064	14.43549	Down
MREG	1.271285	4.848623	7.625686	7.1391162	1.0566683	14.43298	Up
EIF4A1	0.762764	10.50578	7.624021	7.1905486	1.0635438	14.42593	Up
CUEDC1	-1.65884	6.787705	-7.6279	7.2025368	1.0645798	14.42546	Down
TGIF1	0.960047	6.778005	7.623354	7.2112626	1.0651324	14.4231	Up
CMAHP	1.826114	5.894372	7.626573	7.2439159	1.0692160	14.41983	Up
PTMA	0.803249	11.47483	7.621536	7.2680080	1.0720311	14.41539	Up
C4orf3	0.906029	10.82625	7.620986	7.2852555	1.0738336	14.41306	Up
CKAP2	1.609734	6.82507	7.622861	7.3338107	1.0802450	14.40747	Up
C7orf50	-0.84409	7.339754	-7.61798	7.3804221	1.0863615	14.40031	Down
NDUFV3	-1.29891	10.34393	-7.62128	7.4101865	1.0899914	14.39752	Down
ZNF559	1.347718	6.891065	7.61683	7.4169660	1.0902378	14.39545	Up
PPID	-0.84776	9.204825	-7.6161	7.4403809	1.0929274	14.39235	Down
AJUBA	2.224131	6.237071	7.62296	7.5017644	1.1011868	14.38669	Up
HUS1B	-0.9366	5.636293	-7.61401	7.5077739	1.1013120	14.38349	Down
FOXK2	-1.07582	7.399275	-7.61317	7.5349896	1.1045457	14.37993	Down
CIZ1	-0.80294	7.389912	-7.6071	7.7346249	1.1330323	14.35423	Down
TCOF1	-0.81431	6.322108	-7.60584	7.7766982	1.1384148	14.3489	Down
CDK17	0.761814	7.974101	7.605338	7.7936152	1.1401098	14.34676	Up
SLC9B2	1.097242	4.422656	7.604514	7.8213597	1.1431036	14.34327	Up
NBPF3	1.058047	5.890036	7.604412	7.8247851	1.1431036	14.34284	Up
GIMAP8	1.032564	8.265148	7.60423	7.8309431	1.1432213	14.34206	Up
UGDH	1.293899	6.790764	7.603803	7.8453497	1.1445422	14.34026	Up
KMT2B	-0.90624	7.023699	-7.60096	7.9420715	1.1578618	14.32821	Down
NME6	-0.62862	6.703743	-7.60022	7.9674149	1.1607643	14.32508	Down
VPS4A	-0.92979	8.930441	-7.59927	8.0002397	1.1647520	14.32104	Down
PTPN21	0.717201	8.426695	7.598316	8.0331284	1.1687435	14.31701	Up
SLC25A19	-0.67701	5.963098	-7.59573	8.1231327	1.1810338	14.30606	Down

ATG9A	-1.00134	8.253654	-7.59431	8.1731651	1.1874997	14.30002	Down
DIS3L	-0.78626	8.34183	-7.59339	8.2054323	1.1913774	14.29615	Down
TFPT	-0.70117	5.690885	-7.59299	8.2196051	1.1921276	14.29445	Down
C16orf86	-0.93346	5.134765	-7.59293	8.2217623	1.1921276	14.29419	Down
SULF2	1.371756	8.108703	7.593429	8.2690635	1.1981727	14.28906	Up
CALHM2	1.261722	6.71479	7.59093	8.2929638	1.2008211	14.28572	Up
HOXA13	-1.28633	4.249206	-7.59	8.3263439	1.2048377	14.28177	Down
PTPN12	0.945359	9.111933	7.588201	8.3910552	1.2133795	14.27416	Up
SPATA6	0.869156	4.974414	7.586385	8.4569842	1.2220857	14.26647	Up
TSPAN13	1.045483	8.956173	7.584888	8.5117040	1.2291614	14.26013	Up
SPATA2	-1.15895	6.241366	-7.58242	8.6028243	1.2414805	14.24966	Down
COX5A	-0.57717	12.75142	-7.58165	8.6312925	1.2447477	14.24641	Down
ACIN1	-0.77441	7.493479	-7.578	8.7682721	1.2627967	14.23094	Down
FCGR2A	2.057277	6.305337	7.584346	8.8331494	1.2712830	14.22593	Up
CSTF3	0.590488	7.235627	7.575955	8.8457584	1.2722404	14.22229	Up
ZC3H8	-0.81229	6.581949	-7.57568	8.8564148	1.2729159	14.22111	Down
RPP25L	-1.19616	8.475928	-7.57526	8.8721539	1.2743205	14.21936	Down
CLEC4A	2.004243	5.200462	7.580231	8.8785273	1.2743789	14.22008	Up
MARK4	-0.74737	6.600521	-7.57348	8.9406117	1.2824283	14.21181	Down
ADRA2A	2.398825	5.245768	7.580355	9.0088564	1.2913500	14.20673	Up
RHNO1	0.839551	5.966437	7.571111	9.0323356	1.2938472	14.20177	Up
CPXM1	3.190961	5.295821	7.582432	9.1662567	1.3121508	14.1914	Up
HACD1	1.400113	10.13533	7.567005	9.3982102	1.3444539	14.16417	Up
MVP	1.077742	8.04408	7.56139	9.4186530	1.3464765	14.16061	Up
KLF15	-1.7473	8.184362	-7.56926	9.4335421	1.3477029	14.16135	Down
CCDC71	-0.74121	5.081578	-7.56012	9.4702499	1.3520427	14.15524	Down
ASB2	-1.83129	10.06707	-7.56591	9.5854609	1.3674405	14.14575	Down
NDST2	0.758104	6.154852	7.557183	9.5909068	1.3674405	14.1428	Up
PLSCR4	1.539107	8.334702	7.561659	9.6167748	1.3699855	14.14158	Up
TARS3	-0.94933	8.694901	-7.55644	9.6215861	1.3699855	14.13966	Down
CHASERR	0.570784	10.14841	7.555955	9.6417711	1.3719450	14.1376	Up
CMC2	-0.8625	9.297123	-7.55553	9.6594954	1.3735519	14.1358	Down
SEC24D	1.858178	6.482931	7.559276	9.7158479	1.3806459	14.1315	Up
TMEM47	0.732087	10.50412	7.553509	9.7439057	1.3837123	14.12724	Up
WRN	1.069172	4.874803	7.54908	9.9315891	1.4094277	14.10849	Up
STK40	-1.07121	8.155797	-7.54787	9.9835655	1.4150575	14.10336	Down
ARMH3	-1.19248	6.51964	-7.54785	9.9845101	1.4150575	14.10327	Down
METTL22	-0.97918	8.499568	-7.54744	1.0002021	1.4165993	14.10155	Down
FANCG	0.961278	5.226501	7.545112	1.0102812	1.4289792	14.09169	Up
MAPKAPK	-0.85857	9.318543	-7.54468	1.0121799	1.4307173	14.08985	Down
SPRING1	0.76817	6.634964	7.544126	1.0145789	1.4328947	14.08752	Up
LGR5	-4.38908	5.228975	-7.56012	1.0150621	1.4328947	14.09155	Down
GET4	-1.06652	5.724831	-7.54259	1.0213127	1.4407661	14.08102	Down
MMD	2.039814	7.355561	7.550493	1.0241706	1.4436325	14.08067	Up
MAMDC2	1.347388	8.315191	7.543622	1.0246965	1.4436325	14.07827	Up
NOX1	-1.07306	5.329663	-7.53976	1.0338587	1.4555804	14.06902	Down
LITATS1	-0.9464	4.694038	-7.53952	1.0348941	1.4560783	14.06804	Down
HIP1R	-0.805	5.670906	-7.53524	1.0541820	1.4818092	14.04989	Down
LINC0185	-3.4144	5.002822	-7.5512	1.0545697	1.4818092	14.05402	Down
VCP	-0.51178	9.996561	-7.53463	1.0569455	1.4841711	14.04732	Down
RAB32	1.126114	6.407313	7.532807	1.0652679	1.4948747	14.03961	Up
CKS1B	0.950693	7.518652	7.532638	1.0660421	1.4949789	14.03889	Up
ARMC5	-1.04941	5.556534	-7.53083	1.0743573	1.5047285	14.03126	Down
ENPP5	-1.95989	6.667622	-7.53868	1.0744035	1.5047285	14.03342	Down
NKAP	-0.58898	7.228144	-7.52999	1.0782651	1.5091472	14.02769	Down
ST6GAL1	-0.8245	7.85642	-7.52959	1.0801481	1.5107927	14.02598	Down
NRN1	1.094665	7.677878	7.525451	1.0995550	1.5369303	14.00848	Up
RFLNA	-1.22743	4.786258	-7.52484	1.1024585	1.5399809	14.00588	Down

ENSA	-0.95653	10.20328	-7.52465	1.1033731	1.5402512	14.00507	Down
DPYD	1.438621	7.567119	7.52673	1.1050656	1.5416063	14.00425	Up
SERPINE2	2.052812	8.378102	7.53206	1.1085326	1.5454334	14.00287	Up
PEMT	-1.02224	6.457401	-7.52285	1.1119598	1.5492001	13.99745	Down
OLFML2B	2.17612	8.127108	7.531508	1.1159499	1.5537456	13.9966	Up
MAL	1.69661	6.29578	7.524477	1.1210019	1.5597628	13.99048	Up
PARVB	-1.47561	8.347087	-7.52237	1.1341558	1.5760117	13.97919	Down
ELOF1	-0.66552	7.574524	-7.51614	1.1445360	1.5894018	13.96907	Down
CEP57	0.746474	7.806919	7.514273	1.1537750	1.6011908	13.96117	Up
FAM222B	-1.02648	7.232073	-7.51171	1.1665554	1.6170414	13.95035	Down
FRG1CP	0.709706	7.418201	7.511558	1.1673392	1.6170414	13.94969	Up
ILRUN	-1.10113	8.285933	-7.51153	1.1674678	1.6170414	13.94958	Down
EMB	1.925065	6.987128	7.519147	1.1713356	1.6213471	13.94869	Up
GAS7	1.234069	8.184368	7.508495	1.1828372	1.6362071	13.93673	Up
SLFN11	1.225122	6.257575	7.506741	1.1918020	1.6475410	13.92931	Up
PCDHB14	2.531578	5.095103	7.512464	1.2058200	1.6648545	13.92019	Up
LINC0059	1.736549	6.086855	7.507514	1.2058854	1.6648545	13.91875	Up
FABP4	1.632387	11.05301	7.510497	1.2160454	1.6777970	13.9119	Up
C3AR1	2.110523	6.90749	7.509768	1.2198573	1.6819698	13.90882	Up
NFYB	-0.87224	8.024316	-7.49867	1.2339601	1.7003174	13.89515	Down
JOSD2	-1.04727	5.422763	-7.49717	1.2419538	1.7102289	13.8888	Down
BASP1	1.68633	7.715368	7.501297	1.2465905	1.7155077	13.88655	Up
SLC39A9	0.548682	7.692554	7.491728	1.2713700	1.7484817	13.8658	Up
LINC0112	-1.55711	7.3024	-7.49327	1.2853285	1.7665409	13.85622	Down
LDLRAD3	1.115325	6.721228	7.485758	1.3044617	1.7916844	13.84055	Up
ARHGAP1	1.488639	6.063026	7.485074	1.3083091	1.7958140	13.83765	Up
SEL1L3	1.221174	6.232064	7.484721	1.3102970	1.7973875	13.83616	Up
SNX18	1.230199	9.507879	7.485757	1.3118813	1.7984057	13.83535	Up
FAM222A	-1.04936	4.249754	-7.48362	1.3165219	1.8036097	13.8315	Down
AP2A1	-1.48973	6.367021	-7.48156	1.3282539	1.8185158	13.82279	Down
PARP9	0.985381	7.442922	7.480954	1.3317130	1.8220837	13.82023	Up
BRD3OS	1.341566	5.322991	7.480162	1.3362606	1.8271354	13.81688	Up
FICD	-0.70622	5.776012	-7.47939	1.3407376	1.8313303	13.81359	Down
PPIB	1.364307	9.562294	7.483393	1.3410434	1.8313303	13.81452	Up
MPZL1	0.939961	7.432987	7.479004	1.3429418	1.8327509	13.81198	Up
TRIM25	0.821096	8.217333	7.477507	1.3516199	1.8434162	13.80565	Up
ZNF711	1.522092	5.475069	7.476454	1.3577554	1.8506024	13.8012	Up
SLC28A1	-1.06852	4.479704	-7.47504	1.3660668	1.8607433	13.7952	Down
INO80E	-0.73463	6.255249	-7.47419	1.3710303	1.8663139	13.79164	Down
COL4A2	1.196406	10.45936	7.472433	1.3957699	1.8975719	13.77475	Up
CRTAP	0.965462	9.320759	7.469701	1.3977964	1.8991180	13.77264	Up
IKBKB	-0.84539	8.42462	-7.46871	1.4037977	1.9060592	13.76843	Down
STK38	0.814156	8.271126	7.466911	1.4146749	1.9196078	13.76085	Up
C3orf18	-0.96322	7.387215	-7.465	1.4263368	1.9337386	13.75278	Down
ARHGAP3	4.953225	6.606162	7.48895	1.4268995	1.9337386	13.75921	Up
SLC40A1	0.827771	11.1484	7.46275	1.4402373	1.9505763	13.74325	Up
LFNG	1.653795	5.851939	7.461769	1.4571883	1.9722831	13.73225	Up
BLVRB	-0.77671	9.186982	-7.45542	1.4863810	2.0105210	13.71226	Down
NPLOC4	-0.74198	8.661037	-7.45366	1.4976476	2.0244783	13.70484	Down
ARPC1B	1.346949	8.183215	7.453027	1.5148988	2.0465028	13.69417	Up
PDIA5	1.173881	7.063638	7.450717	1.5167634	2.0477265	13.69238	Up
ARF3	1.015316	7.81941	7.449733	1.5231956	2.0551113	13.68822	Up
LEAP2	-0.99173	6.424223	-7.44832	1.5325080	2.0663703	13.68223	Down
ANP32B	0.71615	10.34561	7.44719	1.5399521	2.0750977	13.67747	Up
LRRC20	-1.27087	8.756706	-7.4453	1.5604885	2.1014448	13.6648	Down
ATP8B4	2.133118	5.223409	7.448737	1.5673452	2.1093483	13.66176	Up
SATB1	-1.01376	10.11123	-7.44242	1.5719042	2.1141518	13.65729	Down
DNAJC25-	1.018562	10.24195	7.442208	1.5733070	2.1147068	13.65642	Up

SPA17	0.797363	4.444165	7.441305	1.5794295	2.1216009	13.6526	Up
UGCG	1.695572	8.417754	7.448603	1.5820879	2.1238362	13.65315	Up
HLA-DQB	1.767602	7.908545	7.448878	1.5835570	2.1244730	13.65238	Up
H6PD	-1.08965	7.808089	-7.44007	1.5878474	2.1288917	13.64738	Down
ACSS1	-1.26413	7.218316	-7.4399	1.5889814	2.1290757	13.64668	Down
HOMER2	-1.60016	8.341994	-7.44442	1.5916119	2.1312631	13.64645	Down
VEGFA	-0.99672	10.273	-7.43914	1.5942292	2.1334303	13.64344	Down
DYNLL1	0.945143	10.60257	7.437846	1.6031075	2.1439681	13.63798	Up
PELP1	-0.84134	6.781588	-7.43676	1.6106383	2.1526917	13.63338	Down
IGSF10	3.294359	5.880453	7.451794	1.6138765	2.1556708	13.63583	Up
LINC0280	1.887307	7.337897	7.44058	1.6414550	2.1911373	13.61711	Up
ACKR3	1.085772	9.284595	7.432057	1.6435251	2.1925302	13.61352	Up
TAF11	0.774286	8.892311	7.428816	1.6665913	2.2219137	13.59982	Up
SLC25A44	-0.61977	7.8525	-7.42546	1.6908404	2.2528365	13.58563	Down
MDM1	1.226422	5.74485	7.424696	1.6963883	2.2588192	13.58241	Up
S100A2	3.489143	5.11002	7.439944	1.6977886	2.2592753	13.58603	Up
TRAPPC12	-0.84131	7.749331	-7.42334	1.7063182	2.2692120	13.57668	Down
TOM1L2	-0.85111	8.176235	-7.42198	1.7163383	2.2811172	13.57093	Down
G3BP1	-0.58848	10.00565	-7.4208	1.7250616	2.2912852	13.56595	Down
EFS	1.289795	6.113183	7.419439	1.7351780	2.3032897	13.5602	Up
PTPRK	0.886518	7.722052	7.416727	1.7555271	2.3288539	13.54875	Up
B3GALNT1	1.43962	5.118712	7.412676	1.7863740	2.3683040	13.53163	Up
HRH1	2.100082	4.761492	7.415304	1.8035021	2.3895284	13.52365	Up
ADAMTS1	1.758492	5.994801	7.411339	1.8275177	2.4198465	13.5104	Up
PTBP1	0.839548	8.650183	7.406461	1.8347501	2.4268943	13.50538	Up
TAF10	-0.8624	9.245854	-7.40642	1.8351129	2.4268943	13.50519	Down
TRAPPC1	-0.81082	8.172968	-7.406	1.8383706	2.4288169	13.50344	Down
ZNF524	-0.84284	4.823487	-7.40591	1.8391399	2.4288169	13.50303	Down
EVX1	-1.20939	4.160692	-7.4058	1.8399784	2.4288169	13.50259	Down
FKBP14	1.128146	6.867996	7.40456	1.8498095	2.4402860	13.49735	Up
ACAP2	1.229848	8.088138	7.403114	1.8613484	2.4539925	13.49124	Up
PSAP	0.620662	11.68683	7.401279	1.8760880	2.4718993	13.48349	Up
TANGO2	-0.80923	7.009688	-7.40065	1.8811753	2.4770741	13.48083	Down
HDAC10	-1.15771	4.376346	-7.40038	1.8833400	2.4783965	13.4797	Down
MLXIP	-0.9856	9.104339	-7.40024	1.8845234	2.4784268	13.47908	Down
ZBTB45	-0.85054	5.97858	-7.39974	1.8885246	2.4821606	13.477	Down
GMFB	1.110616	8.04546	7.397112	1.9099994	2.5088418	13.46589	Up
SPON1	3.014497	6.319064	7.408275	1.9440018	2.5519355	13.45296	Up
CASP8	1.518693	5.699151	7.390234	1.9673081	2.5809439	13.43685	Up
WNK1	-0.59359	10.09301	-7.38863	1.9808816	2.5971559	13.4301	Down
CLK3	-0.83599	7.126864	-7.38505	2.0116729	2.6359086	13.41494	Down
SRI	0.680911	8.935327	7.384284	2.0182694	2.6429306	13.41173	Up
TSPAN12	2.155612	6.669155	7.390007	2.0388578	2.6682552	13.4041	Up
PCCA-DT	-0.93797	5.543133	-7.38129	2.0443902	2.6738571	13.39909	Down
GSTM4	-1.33082	7.444081	-7.37922	2.0679360	2.7029974	13.38802	Down
SLC12A2-	-0.84539	6.396219	-7.37809	2.0727059	2.7063340	13.38558	Down
CHCHD4	-1.03706	7.923196	-7.37806	2.0730230	2.7063340	13.38543	Down
LINC0271	-1.87791	4.537421	-7.37799	2.1036283	2.7446117	13.372	Down
CRY2	-0.99867	7.798123	-7.37402	2.1093240	2.7503627	13.36838	Down
ST3GAL3-	-1.62921	7.696697	-7.37832	2.1137138	2.7544051	13.36772	Down
ZNF217	1.632222	6.845898	7.376946	2.1182350	2.7586136	13.36537	Up
PSME4	-0.85507	9.727224	-7.37265	2.1217495	2.7615068	13.36261	Down
MRPL4	-1.23809	7.890615	-7.37245	2.1235845	2.7622118	13.36176	Down
LOC10537	-1.38697	4.118845	-7.36995	2.1465377	2.7903684	13.3512	Down
DHRS1	0.855469	6.828388	7.369593	2.1497979	2.7929065	13.34971	Up
SCARB2	0.657058	10.92741	7.369269	2.1527955	2.7936894	13.34834	Up
SLC25A5	0.911538	9.582101	7.369245	2.1530165	2.7936894	13.34824	Up
TMX1	1.000063	8.765508	7.367204	2.1719799	2.8165846	13.33962	Up

LGI4	-1.17783	5.872557	-7.36566	2.1864496	2.8336281	13.3331	Down
TMEM161	-1.88003	6.829359	-7.37131	2.2029863	2.8533282	13.32785	Down
CARD16	1.495912	6.429391	7.362056	2.2291035	2.8845568	13.31438	Up
ADAM22	0.979471	5.117861	7.36109	2.2297983	2.8845568	13.31381	Up
DPH2	-0.99412	6.601117	-7.3603	2.2373935	2.8926302	13.31047	Down
PGAM1	0.854048	9.537355	7.359743	2.2427404	2.8977888	13.30813	Up
CARM1	-1.35081	9.255477	-7.36159	2.2625105	2.9215658	13.30064	Down
P2RY2	-1.41008	6.848334	-7.35776	2.2686714	2.9277513	13.29704	Down
LMO2	-0.63371	9.073173	-7.3536	2.3026748	2.9680464	13.28222	Down
PSME3	-0.89661	8.318282	-7.35324	2.3062415	2.9708508	13.2807	Down
CYSTM1	-0.66275	11.55749	-7.3521	2.3175886	2.9836683	13.27588	Down
LOC72839	1.011756	6.448666	7.350901	2.3295727	2.9972900	13.27082	Up
GNG5	-0.67198	11.48685	-7.34798	2.3589375	3.0332443	13.25851	Down
ANLN	2.78165	5.333369	7.35581	2.3701139	3.0457817	13.25647	Up
NT5C1A	-0.88412	4.370495	-7.34575	2.3816797	3.0588042	13.24909	Down
MRPS18A	-1.15781	7.727244	-7.3446	2.3935140	3.0721558	13.24422	Down
TAF1	0.817568	6.748355	7.34401	2.3995594	3.0780655	13.24174	Up
NACA	0.572224	12.36021	7.342402	2.4161828	3.0975290	13.23496	Up
SLC29A1	-1.33537	8.079054	-7.34345	2.4258392	3.1080429	13.23162	Down
TLCD3B	-1.2489	4.258643	-7.34129	2.4277429	3.1080806	13.23027	Down
BICD1	-1.74117	6.338434	-7.34554	2.4287792	3.1080806	13.23112	Down
FRMD4A	1.072079	6.734622	7.340089	2.4403065	3.1209620	13.2252	Up
MGP	1.878365	11.2313	7.351065	2.4481156	3.1290756	13.22548	Up
ZNF511	-0.801	7.976348	-7.33638	2.4794817	3.1672710	13.20956	Down
SPARCL1	0.67972	12.28241	7.335317	2.4908434	3.1798825	13.20507	Up
CHST6	1.490241	6.925734	7.336411	2.5035810	3.1942344	13.20072	Up
SP110	0.866382	7.675334	7.3311	2.5363559	3.2326354	13.18728	Up
MOB1B	1.197597	8.258553	7.331059	2.5368117	3.2326354	13.1871	Up
HS6ST2	2.166035	7.171882	7.338868	2.5382197	3.2326354	13.18888	Up
PICALM	0.718596	9.600963	7.330472	2.5432140	3.2370658	13.18463	Up
GPC6	2.257393	6.371108	7.336024	2.5693141	3.2683390	13.17692	Up
SLC38A7	-0.7759	6.6404	-7.32646	2.5874445	3.2894428	13.16769	Down
TBL3	-1.11025	6.22408	-7.326	2.5924750	3.2938775	13.16579	Down
PINK1	-0.81331	10.80384	-7.32526	2.6007169	3.3016562	13.16267	Down
PARP12	0.890116	6.837163	7.325178	2.6016890	3.3016562	13.1623	Up
HS6ST1	-0.74728	7.092677	-7.32386	2.6165001	3.3184803	13.15672	Down
NRDC	-0.67905	10.59653	-7.32232	2.6338606	3.3385161	13.15023	Down
C1QB	2.053413	7.038034	7.326928	2.6713557	3.3840341	13.13866	Up
TUSC3	1.823888	7.017801	7.325375	2.6776076	3.3899432	13.13607	Up
NAXE	-0.76693	8.720971	-7.31595	2.7068343	3.4249151	13.12339	Down
SKP2	1.018258	7.166367	7.315051	2.7173130	3.4361381	13.11959	Up
NECTIN1	-0.87795	5.823837	-7.31049	2.7710090	3.4998942	13.10037	Down
MIIP	-0.82652	6.995452	-7.30995	2.7774552	3.5059627	13.09809	Down
NABP2	-1.01801	6.708252	-7.30835	2.7966542	3.5260296	13.09132	Down
PPP1R10	-1.03812	5.545616	-7.30779	2.8033216	3.5323506	13.08899	Down
MPST	-0.92062	7.534862	-7.30544	2.8317692	3.5657951	13.07907	Down
ATXN2L	-1.09394	4.82246	-7.30532	2.8332027	3.5657951	13.07857	Down
UST	1.335582	5.400269	7.302117	2.8724535	3.6130661	13.06506	Up
FAM216A	1.271851	6.868389	7.301187	2.8839430	3.6253828	13.06114	Up
PPP1R1C	-1.771	5.5212	-7.3024	2.9093192	3.6551318	13.05349	Down
NEDD4	-1.36024	9.263569	-7.3001	2.9451581	3.6979828	13.04163	Down
ACAD10	-0.846	5.954348	-7.29496	2.9620198	3.7169694	13.0349	Down
EFHC1	1.428885	7.586792	7.296557	2.9805349	3.7380073	13.02968	Up
GALNT4	1.274345	4.549941	7.291534	3.0059274	3.7654310	13.02045	Up
ELN	2.505318	7.335988	7.303522	3.0159023	3.7740293	13.02096	Up
SLC16A2	1.328796	5.613508	7.29073	3.0163255	3.7740293	13.01706	Up
LOXL2	1.633392	7.410638	7.285773	3.1429808	3.9278984	12.97803	Up
HAGHL	-0.9684	6.006655	-7.27995	3.1590915	3.9457238	12.97164	Down

DES	-0.59302	12.63268	-7.27866	3.1766000	3.9650482	12.96621	Down
SLX9	-1.09622	5.864784	-7.27854	3.1782763	3.9650482	12.9657	Down
PLXNA1	0.880201	7.949779	7.273059	3.2539263	4.0570553	12.94259	Up
F8A1	-0.81319	8.621989	-7.27147	3.2761377	4.0823658	12.93591	Down
CYTH2	-0.54161	7.639561	-7.27081	3.2854199	4.0915451	12.93314	Down
MRPS9	-0.79103	9.001356	-7.27053	3.2893928	4.0941056	12.93195	Down
RPS3	0.684484	12.27736	7.270271	3.2930820	4.0963102	12.93085	Up
LILRB1	1.054895	5.311662	7.269127	3.3092763	4.1140584	12.92603	Up
SGTA	-0.87367	7.032737	-7.26788	3.3269790	4.1336601	12.92079	Down
LOC10537	-1.26949	6.471969	-7.26597	3.3544418	4.1653586	12.91272	Down
TLR1	1.715	4.679496	7.266929	3.3638620	4.1746290	12.91044	Up
MYG1	-0.71793	7.194379	-7.2649	3.3698818	4.1796710	12.90821	Down
KLC4	-0.61973	6.460965	-7.264	3.3828171	4.1932797	12.90445	Down
PPIF	-1.33643	8.658745	-7.26665	3.3880934	4.1973840	12.90381	Down
STIM1	-0.87754	7.143684	-7.26265	3.4025606	4.2128631	12.89873	Down
LOC10537	-0.75702	4.930987	-7.2622	3.4090316	4.2184297	12.89686	Down
MAGEH1	0.820957	7.878397	7.260314	3.4367726	4.2502946	12.88891	Up
PPM1G	-0.88136	7.076711	-7.25963	3.4468120	4.2602437	12.88604	Down
LONRF1	-1.27202	7.75927	-7.25931	3.4584304	4.2721316	12.88287	Down
DNAJC11	-0.58286	8.051448	-7.25845	3.4644304	4.2770697	12.88103	Down
STAM	0.891646	8.466402	7.257996	3.4711119	4.2828428	12.87914	Up
PTOV1	-0.91401	8.771566	-7.25699	3.4861121	4.2988673	12.87491	Down
MAOB	-1.71292	8.7884	-7.26458	3.4881717	4.2989251	12.87663	Down
PFKFB1	-2.17178	6.427395	-7.26323	3.5084579	4.3214328	12.87093	Down
GRINA	-1.25317	6.631346	-7.25451	3.5233951	4.3373299	12.86446	Down
HLA-DQA	2.497326	8.340309	7.2658	3.5724066	4.3951302	12.85521	Up
NXT2	0.901785	6.32183	7.25107	3.5757793	4.3967469	12.84997	Up
PPP1R15A	-0.77945	7.404663	-7.25072	3.5811840	4.4001850	12.84849	Down
AP1S2	-0.5778	11.08675	-7.2505	3.5844750	4.4001850	12.84758	Down
CMPK2	1.218021	7.32085	7.250485	3.5847560	4.4001850	12.84751	Up
ADGRA2	1.094358	6.816439	7.250115	3.5904470	4.4022569	12.84595	Up
EPS8	0.942235	9.214833	7.250095	3.5907627	4.4022569	12.84586	Up
PRR4	-0.92194	5.69837	-7.24997	3.5926274	4.4022569	12.84535	Down
CALCOCC	-0.68083	9.241169	-7.24863	3.6134494	4.4252324	12.83968	Down
PDXDC1	-0.65633	8.905478	-7.24811	3.6214293	4.4324635	12.83751	Down
ACADSB	-0.80678	9.205814	-7.24781	3.6261156	4.4356573	12.83624	Down
COL12A1	1.547556	9.211285	7.24987	3.6839158	4.5037822	12.82243	Up
CAP1	0.837357	9.292685	7.242514	3.7094132	4.5323597	12.81394	Up
IDH3A	-0.91105	9.498402	-7.24167	3.7227826	4.5460944	12.81041	Down
GAS8	-0.7799	5.875983	-7.24055	3.7408235	4.5655148	12.80566	Down
COTL1	2.132151	6.187282	7.247081	3.7592572	4.5853921	12.80313	Up
RAVER1	-0.79654	4.534783	-7.23845	3.7746361	4.6015228	12.79682	Down
BAIAP2L1	1.191023	4.750221	7.23741	3.7914752	4.6194140	12.79245	Up
CRYAB	-0.5848	13.17234	-7.23639	3.8080956	4.6370186	12.78816	Down
LARGE1	-1.10802	8.138753	-7.23581	3.8175434	4.6458741	12.78572	Down
XIRP2	-0.78531	13.12255	-7.23311	3.8619728	4.6972674	12.77436	Down
NAGK	0.807804	8.040376	7.232277	3.8758394	4.7100000	12.77084	Up
HDAC11	-1.27096	6.738349	-7.23222	3.8768517	4.7100000	12.77059	Down
PLEKHO2	1.055005	6.110388	7.229283	3.9259092	4.7663582	12.75824	Up
USF2	-0.93355	7.924468	-7.22918	3.9277041	4.7663582	12.75779	Down
RASA2	1.467815	7.378737	7.231101	3.9498609	4.7905241	12.75324	Up
TBC1D1	-0.96618	7.748306	-7.22588	3.9835995	4.8253105	12.74391	Down
LY6G5B	-0.96148	4.563287	-7.22578	3.9853206	4.8253105	12.74349	Down
KCNT2	1.963566	4.984464	7.224915	4.0784967	4.9353277	12.72216	Up
ZNF696	-1.32071	4.12753	-7.21593	4.1571993	5.0277159	12.70203	Down
DHRS4	-0.98756	7.588104	-7.21497	4.1743139	5.0455572	12.698	Down
FAM98A	-0.6143	9.377669	-7.2107	4.2514296	5.1358616	12.68002	Down
AKAP1	-0.6923	10.79968	-7.21039	4.2570946	5.1397980	12.67872	Down

CARMIL3	-0.96282	5.880907	-7.20984	4.2670607	5.1456325	12.67642	Down
ACTB	0.907822	12.66233	7.211366	4.2675830	5.1456325	12.67677	Up
GORAB	1.082115	6.744407	7.209728	4.2691548	5.1456325	12.67594	Up
DUT	0.684386	10.45427	7.209123	4.2802252	5.1560659	12.6734	Up
VPS51	-0.65789	9.205477	-7.20815	4.2981372	5.1747246	12.6693	Down
USP24	-0.93558	9.375391	-7.20739	4.3121141	5.1886272	12.66611	Down
FUCA1	0.678601	8.695664	7.207202	4.3156218	5.1899241	12.66531	Up
TNK2	-1.04208	6.197027	-7.20679	4.3232808	5.1962088	12.66357	Down
NEURL1	-0.96676	6.265814	-7.20642	4.3300563	5.2004178	12.66203	Down
PLAU	1.951857	7.045841	7.213924	4.3316524	5.2004178	12.66395	Up
TMEM63B	-1.11731	5.98155	-7.20179	4.4169571	5.2956455	12.64252	Down
MRPS26	-0.68905	7.944116	-7.20177	4.4172608	5.2956455	12.64245	Down
BNIP3L	1.368709	9.083968	7.205385	4.4184101	5.2956455	12.6433	Up
MIB2	-1.01912	7.553736	-7.20094	4.4329993	5.3101514	12.63896	Down
SERPINF1	0.524857	12.23617	7.200196	4.4471388	5.3241026	12.63583	Up
NLRX1	-0.88989	7.423588	-7.19902	4.4696344	5.3469767	12.63088	Down
OLFML2A	1.401203	8.238237	7.202606	4.4712522	5.3469767	12.63163	Up
USP39	-0.81852	7.305031	-7.19798	4.4896521	5.3659760	12.62649	Down
NUDT22	-0.74624	7.618056	-7.1973	4.5026561	5.3785083	12.62365	Down
KDM5B	0.761432	6.727493	7.193106	4.5842836	5.4729530	12.60601	Up
LAMA2	1.034157	8.888985	7.192602	4.5942025	5.4817306	12.60389	Up
CLTC	0.610919	10.9422	7.191663	4.6127276	5.5007613	12.59994	Up
SGSM2	0.585432	8.00684	7.189148	4.6626942	5.5572446	12.58936	Up
ARHGAP1	0.791	8.324306	7.18828	4.6800642	5.5748361	12.58571	Up
C1orf54	1.131319	8.2194	7.186641	4.7130269	5.6060336	12.57882	Up
PICK1	-0.83944	4.702585	-7.18662	4.7134861	5.6060336	12.57873	Down
RCAN2	-1.61822	9.180796	-7.19412	4.7141288	5.6060336	12.58087	Down
CDK11A	-0.85436	6.868001	-7.18629	4.7200938	5.6075626	12.57735	Down
NASP	0.639974	7.668005	7.186263	4.7206655	5.6075626	12.57723	Up
TTC8	1.293464	5.984717	7.18564	4.7332878	5.6194309	12.57461	Up
SLC22A5	-1.22661	6.507096	-7.18543	4.7376264	5.6214569	12.57371	Down
GOT1	-1.27237	10.73463	-7.18673	4.8022366	5.6949566	12.56176	Down
CDIN1	-1.20446	6.730369	-7.18157	4.8164979	5.7079680	12.5575	Down
TXN2	-0.81788	8.734551	-7.18147	4.8185533	5.7079680	12.55708	Down
CYTH1	-0.6656	7.149507	-7.17977	4.8537752	5.7444289	12.54993	Down
ADAMTSL	2.746958	4.53761	7.188127	4.8547121	5.7444289	12.55228	Up
ARL4A	1.617663	5.461325	7.180935	4.8598250	5.7472947	12.54915	Up
SRGN	1.313751	10.10747	7.18359	4.8671134	5.7527288	12.54859	Up
TMEM184	-1.12998	5.100892	-7.17733	4.9048597	5.7941368	12.53965	Down
MRPL38	-1.26841	8.438536	-7.17681	4.9488149	5.8428296	12.53137	Down
SLC38A6	1.327355	5.982046	7.173797	4.9795626	5.8758839	12.52481	Up
HIP1	1.298292	6.106354	7.171715	5.0241511	5.9252247	12.51606	Up
SLC44A1	1.122322	7.388255	7.170936	5.0409405	5.9417443	12.51278	Up
MTOR	-0.84082	7.128221	-7.17024	5.0559042	5.9560950	12.50987	Down
TMEM222	-0.73575	7.283033	-7.16682	5.1305638	6.0407156	12.49548	Down
FBXO5	1.21038	4.117635	7.164714	5.1770504	6.0920905	12.48663	Up
ENDOG	-1.18025	9.236805	-7.1651	5.1869552	6.1003849	12.485	Down
DTX4	0.929881	7.134722	7.161787	5.2423364	6.1621255	12.47433	Up
TUT1	-0.93805	4.946612	-7.15981	5.2868612	6.2110441	12.46602	Down
TET2	1.035008	7.762164	7.159232	5.3000057	6.2230633	12.46359	Up
TYMS	2.260212	5.800306	7.16411	5.3587096	6.2885340	12.45504	Up
LINC0156:	1.133432	5.22695	7.156235	5.3684348	6.2964871	12.45099	Up
POPDC3	-0.77984	10.83007	-7.15581	5.3781303	6.3043966	12.44922	Down
FHL3	-1.58412	9.820944	-7.16181	5.4116990	6.3402669	12.44538	Down
TRMT112	0.533415	10.1408	7.15214	5.4633730	6.3972983	12.43378	Up
PTPRU	-0.944	6.078209	-7.15201	5.4665382	6.3974972	12.43321	Down
MAML2	1.260971	6.564837	7.14943	5.5271299	6.4648653	12.42239	Up
SLC3A2	-0.81934	6.898743	-7.14746	5.5738738	6.5159714	12.41413	Down

YAF2	-1.2045	6.216895	-7.14491	5.6350848	6.5839246	12.4034	Down
ATP6V0A1	-0.72873	7.271889	-7.14366	5.6652623	6.6155644	12.39816	Down
NTN4	1.207746	7.883815	7.142216	5.7004518	6.6530191	12.39208	Up
KPNA4	-0.58151	11.22584	-7.14206	5.7041456	6.6536943	12.39145	Down
FOLR2	1.6723	7.104022	7.145871	5.7186209	6.6669381	12.3903	Up
PPP1R3C	-1.1142	12.18871	-7.14484	5.7438945	6.6927496	12.38597	Down
TLE5	-0.94632	9.564424	-7.14014	5.7513733	6.6978097	12.38335	Down
DUX4L1	-1.46874	5.487991	-7.13958	5.7652158	6.7089396	12.38099	Down
POLR2J3	-1.48388	4.64346	-7.13938	5.7700006	6.7089396	12.38018	Down
SOX18	-1.1655	4.573651	-7.13937	5.7703540	6.7089396	12.38012	Down
EEIG2	1.960175	6.63541	7.144627	5.8234061	6.7669373	12.37339	Up
DNAJC21	-0.73896	9.261398	-7.13708	5.8270758	6.7675196	12.37052	Down
ADAMTS1	1.001858	5.441522	7.136467	5.8424538	6.7816918	12.36793	Up
MAZ	-1.01567	8.132793	-7.13533	5.8710517	6.8111853	12.36314	Down
MTCH1	0.517063	10.18764	7.135174	5.8748567	6.8118995	12.3625	Up
ASB10	-1.33379	7.02446	-7.13372	5.9225690	6.8634959	12.35469	Down
MAF	0.617486	10.56582	7.131089	5.9784470	6.9235955	12.34534	Up
PIP4P1	-0.69448	7.076959	-7.13088	5.9836946	6.9235955	12.34448	Down
CLEC7A	2.212198	5.807106	7.138247	5.9841546	6.9235955	12.34666	Up
LDB1	-1.15875	5.54838	-7.12985	6.0103537	6.9501427	12.34012	Down
SLC8A3	-1.16824	7.551598	-7.12785	6.0619781	7.0060459	12.33172	Down
PAK1	1.020493	8.9462	7.124605	6.1466314	7.1000407	12.31811	Up
MLXIPL	-1.28628	5.782953	-7.1236	6.1731067	7.1267682	12.31389	Down
BCL7C	-0.79121	6.788695	-7.12285	6.1930653	7.1459476	12.31072	Down
THSD7A	1.748047	5.133039	7.124426	6.2270926	7.1813307	12.30622	Up
NKIRAS1	-0.78261	8.232144	-7.12092	6.2441826	7.1971533	12.30265	Down
DNAJC1	0.805022	8.363003	7.120505	6.2554094	7.2042803	12.30089	Up
SLC30A7	1.489901	7.425003	7.123782	6.2631793	7.2042803	12.30076	Up
NFIB	0.993769	11.01942	7.120114	6.2658810	7.2042803	12.29925	Up
PXDN	1.314447	8.116748	7.122215	6.2661414	7.2042803	12.29985	Up
MFSD14A	0.576016	8.307502	7.120063	6.2672313	7.2042803	12.29904	Up
HSDL1	-0.88565	6.433464	-7.11615	6.3730643	7.3206487	12.2826	Down
SAMM50	-0.71631	10.12066	-7.11607	6.3753191	7.3206487	12.28225	Down
ATP2B1-A	1.14769	6.762147	7.114124	6.4285010	7.3777498	12.2741	Up
EML6	1.104163	5.438158	7.113969	6.4327722	7.3786868	12.27345	Up
UBAC1	-0.95498	10.06958	-7.11334	6.4500164	7.3944955	12.27082	Down
LATS1	0.807836	7.599152	7.113154	6.4552149	7.3964850	12.27003	Up
ATG4A	-0.85571	7.438996	-7.11247	6.4740695	7.4141113	12.26716	Down
GSTZ1	-1.03659	7.175899	-7.11175	6.4941931	7.4331713	12.26412	Down
SRXN1	-0.99585	6.983837	-7.11023	6.5364416	7.4775211	12.25775	Down
MAP3K1	0.969601	7.553693	7.10974	6.5501879	7.4892352	12.25569	Up
TMEM184	-0.58894	7.746925	-7.10691	6.6299907	7.5764228	12.2438	Down
IGLV4-3	-1.11016	5.623635	-7.10277	6.7484331	7.7076489	12.22642	Down
KCNA7	-1.3351	7.831435	-7.10358	6.7901173	7.7511130	12.22107	Down
CKMT2	-0.87156	12.34062	-7.09736	6.9062747	7.8794987	12.20373	Down
ANKLE2	-0.66638	6.813959	-7.09487	6.9802471	7.9596432	12.19328	Down
FBXO6	-1.37155	6.759548	-7.094	7.0224499	7.9992259	12.18752	Down
APPL1	-0.8899	8.726207	-7.09303	7.0353515	8.0055931	12.18556	Down
CIART	-2.74926	6.514711	-7.10671	7.0355362	8.0055931	12.18974	Down
HLA-DPA1	2.109994	10.92478	7.104871	7.0909298	8.0643280	12.18205	Up
MCCC2	-0.81491	8.121165	-7.08997	7.1280467	8.1022257	12.17271	Down
PITPNM1	-0.88526	5.822247	-7.08501	7.2808916	8.2715576	12.15189	Down
ACLY	0.84155	8.232691	7.083872	7.3162927	8.3073567	12.14713	Up
SNHG32	0.625563	9.292751	7.082717	7.3524920	8.3440236	12.14228	Up
GBP2	0.987666	8.462595	7.081906	7.3780304	8.3685594	12.13888	Up
RHOD	-0.71087	6.002803	-7.08109	7.4037062	8.3932249	12.13547	Down
LY86	1.388583	5.868838	7.080556	7.4207340	8.4080656	12.13322	Up
PARP6	-0.50025	8.037533	-7.07872	7.4790665	8.4696661	12.12553	Down

CLCN1	-1.38417	6.15584	-7.06922	7.7890393	8.8160202	12.08568	Down
SOX4	1.423285	8.390618	7.072981	7.8064231	8.8310162	12.08481	Up
ZC3HAV1l	1.599473	7.140705	7.071932	7.8414528	8.8659476	12.08042	Up
CCDC90B	0.579746	8.737315	7.063475	7.9826867	9.0208583	12.06157	Up
MIPEPP3	-0.71837	6.489498	-7.06029	8.0920375	9.1395948	12.04822	Down
ERRFI1	1.698092	8.703107	7.067306	8.0980219	9.1415196	12.04973	Up
JMJD6	-0.7632	6.809848	-7.05676	8.2149913	9.2686627	12.03342	Down
SLC52A2	-0.56042	5.96443	-7.05322	8.3402652	9.4000731	12.01857	Down
TSTD1	1.131029	8.391515	7.051986	8.3842928	9.4447113	12.0134	Up
PARD6A	-1.21266	5.066167	-7.05055	8.4359529	9.4978957	12.00738	Down
DPP4	1.7711	5.918458	7.053878	8.4691713	9.5302720	12.00484	Up
NHERF2	-2.1196	3.339036	-7.05104	8.5440161	9.6094311	11.99597	Down
DDAH2	0.903433	7.030783	7.047344	8.5521890	9.6135607	11.99395	Up
VMP1	0.820891	8.729217	7.046808	8.5718264	9.6304604	11.9917	Up
PACC1	0.996004	5.311683	7.046687	8.5762410	9.6304604	11.99119	Up
EEF1A2	-0.98558	12.28739	-7.04247	8.8430518	9.9244714	11.96205	Down
IL12RB2	-2.47291	5.230005	-7.04653	8.8473610	9.9244714	11.96287	Down
PPP4R1	0.526879	8.315743	7.03673	8.9487929	1.0032982	11.94946	Up
TMX3	0.909355	8.234286	7.034589	9.0310047	1.0119842	11.94049	Up
AFAP1L1	0.901835	8.73134	7.034055	9.0516005	1.0137602	11.93825	Up
SNORD11	2.488772	6.733946	7.04153	9.1654280	1.0259707	11.92924	Up
NDUFB11	-0.71714	11.05118	-7.03077	9.1795149	1.0270093	11.92448	Down
WBP2	-1.18484	7.298332	-7.03064	9.1847046	1.0270519	11.92393	Down
MAP2K4	-0.54179	8.232842	-7.02984	9.2161523	1.0300292	11.92057	Down
LINC0111(	1.377876	5.419845	7.028413	9.2723279	1.0357655	11.91461	Up
DLL1	1.233153	6.640919	7.027104	9.3242920	1.0410257	11.90913	Up
ZBTB2	-0.65458	7.069117	-7.02685	9.3342865	1.0415971	11.90808	Down
ATXN7L3-	0.98952	4.454398	7.025741	9.3787145	1.0460082	11.90342	Up
GINS1	1.731381	4.049633	7.025514	9.4478189	1.0531655	11.89668	Up
TBC1D17	-1.22923	7.44758	-7.0235	9.4690640	1.0549831	11.89401	Down
THY1	2.361262	6.921867	7.032561	9.5076766	1.0587328	11.89315	Up
RUSC2	-0.8916	7.069727	-7.02036	9.5967820	1.0680983	11.88086	Down
SGF29	-0.97648	6.959423	-7.01896	9.6542067	1.0735496	11.87501	Down
E2F1	-0.86544	4.779498	-7.01892	9.6558143	1.0735496	11.87485	Down
NECTIN3	1.12087	5.403569	7.016417	9.7595298	1.0845163	11.86436	Up
VTI1B	-0.64493	9.272436	-7.01504	9.8172780	1.0903662	11.85857	Down
TMEM119	2.277113	5.35808	7.020653	9.8780746	1.0965484	11.85473	Up
CRLF1	-2.14199	5.002339	-7.01868	9.9588394	1.1049397	11.84672	Down
GIMAP4	0.739955	8.000071	7.01111	9.9831289	1.1070595	11.84213	Up
USP5	-0.91255	6.615483	-7.00908	1.0070213	1.1161370	11.83361	Down
PPAN	-0.94198	5.703927	-7.00772	1.0128748	1.1220426	11.82793	Down
IFITM1	1.168757	10.31756	7.010059	1.0146020	1.1233732	11.82711	Up
PROCR	1.601423	7.025857	7.010648	1.0182955	1.1268785	11.824	Up
ALKBH4	-1.06647	5.216444	-7.00596	1.0204893	1.1287214	11.82058	Down
FLI1	1.110115	7.115566	7.001888	1.0383871	1.1479230	11.80352	Up
HCN2	-0.72487	5.41024	-6.99992	1.0471412	1.1570017	11.79528	Down
KLF10	1.046877	10.07999	6.99952	1.0489309	1.1583798	11.79361	Up
ZFHX2	-0.6507	4.939503	-6.99907	1.0509253	1.1599826	11.79174	Down
EEPD1	-0.78446	8.283828	-6.99836	1.0541268	1.1629153	11.78876	Down
PBX2	-0.68924	6.903219	-6.9979	1.0562036	1.1646049	11.78683	Down
ETHE1	-0.76652	6.987202	-6.99716	1.0595362	1.1676767	11.78374	Down
GDNF	-3.26526	4.26064	-7.00979	1.0621263	1.1693244	11.78549	Down
FAM162B	0.729869	7.075986	6.99642	1.0628969	1.1695699	11.78063	Up
GFRA1	1.282166	6.553588	6.995523	1.0669737	1.1734513	11.77687	Up
NIPSNAP3	1.776343	8.25983	6.999418	1.0812373	1.1881560	11.76605	Up
PLD1	0.796496	5.738209	6.992318	1.0816618	1.1881560	11.76346	Up
CHRNB1	-1.00552	7.899169	-6.99224	1.0820130	1.1881560	11.76314	Down
HSPA2	-1.51846	10.28527	-6.99824	1.0866503	1.1926350	11.76115	Down

GPT	-0.98287	6.232727	-6.98863	1.0988075	1.2052131	11.74803	Down
CEBPB	-0.88664	11.35948	-6.98854	1.0992392	1.2052131	11.74764	Down
ATF4	-0.57509	11.56303	-6.98791	1.1022078	1.2078478	11.745	Down
GALC	0.853861	7.927576	6.986508	1.1088076	1.2144571	11.73914	Up
STIM2	0.861988	6.775237	6.986096	1.1107539	1.2159653	11.73742	Up
TMED8	0.713922	6.324644	6.98582	1.1120656	1.2167775	11.73626	Up
DNPH1	-0.96278	7.22661	-6.98567	1.1127901	1.2169468	11.73562	Down
NAV3	1.500558	6.206135	6.985235	1.1218551	1.2256052	11.72812	Up
TCF12	0.571827	8.826107	6.980421	1.1379714	1.2423813	11.71367	Up
SNHG1	0.720934	7.890743	6.980338	1.1383745	1.2423813	11.71332	Up
SNX30	0.935353	5.349838	6.979088	1.1444566	1.2478186	11.70809	Up
KTN1	-0.76331	11.77496	-6.97907	1.1445250	1.2478186	11.70803	Down
WDR62	-1.66784	7.451499	-6.98498	1.1454014	1.2481369	11.7092	Down
NCAPH2	-0.70986	6.404184	-6.97768	1.1513668	1.2539976	11.70219	Down
VAPB	-0.88552	9.262408	-6.97737	1.1528817	1.2550075	11.7009	Down
TFIP11	-1.0522	7.336647	-6.9758	1.1606390	1.2621654	11.69432	Down
LCP2	1.774596	5.913472	6.979143	1.1645323	1.2657547	11.69234	Up
LYZ	2.674178	9.149342	6.987351	1.1682696	1.2691711	11.69202	Up
TSPAN17	-0.87841	7.006391	-6.9723	1.1780776	1.2791755	11.67969	Down
DUBR	1.43553	5.066983	6.970119	1.1890806	1.2899227	11.67056	Up
C15orf61	-0.74582	10.82895	-6.9701	1.1891834	1.2899227	11.67048	Down
ARRDC1	-0.85509	5.914298	-6.96833	1.1982014	1.2990449	11.66307	Down
LETM1	-0.80325	5.766985	-6.96821	1.1988098	1.2990451	11.66257	Down
FBL	0.595775	9.59202	6.967712	1.2013454	1.3011326	11.6605	Up
CYS1	1.765631	5.460815	6.970154	1.2060958	1.3056155	11.65769	Up
PHETA1	-0.79894	5.371183	-6.9652	1.2142925	1.3138227	11.64998	Down
TRIM44	0.648144	9.017782	6.964294	1.2189791	1.3182256	11.6462	Up
PLPP3	1.125562	10.22037	6.965822	1.2205285	1.3192331	11.64554	Up
SQLE	1.268347	5.340097	6.963265	1.2243390	1.3226825	11.6419	Up
RAB27A	1.068038	6.659084	6.960491	1.2389046	1.3377414	11.6303	Up
ANKRD23	-1.42372	10.51602	-6.96507	1.2513366	1.3504824	11.6227	Down
DHX30	-0.58883	7.778442	-6.95791	1.2525936	1.3511563	11.61952	Down
ZNF121	0.579072	8.388755	6.955943	1.2631578	1.3618640	11.61128	Up
MCRIP2	-1.22708	8.723737	-6.95712	1.2663558	1.3646230	11.60936	Down
CARD19	-0.59503	7.681841	-6.95415	1.2728363	1.3709147	11.60379	Down
ELL3	1.538346	6.122944	6.955134	1.2782783	1.3760820	11.60023	Up
ZNF584	-0.90173	5.566024	-6.95303	1.2789361	1.3760965	11.5991	Down
DNAJB5	-1.32376	8.462693	-6.9544	1.2881410	1.3853029	11.59303	Down
CCR2	1.485743	4.535156	6.949863	1.2963186	1.3933957	11.58586	Up
OPA1	-0.69541	8.576465	-6.9495	1.2983220	1.3948471	11.58434	Down
TOMM40L	-1.04973	7.260414	-6.94651	1.3150012	1.4113465	11.57182	Down
SAMD9	1.43755	5.74618	6.945914	1.3183182	1.4141959	11.56935	Up
C4orf46	1.0656	5.406896	6.943506	1.3319195	1.4280691	11.55928	Up
USP38	-0.87654	9.094644	-6.94262	1.3369328	1.4327251	11.55559	Down
RNF187	-0.79157	8.498634	-6.94144	1.3436807	1.4392343	11.55065	Down
TMEM14A	1.094381	6.690986	6.941213	1.3449918	1.4399166	11.5497	Up
MAX	0.54514	9.185033	6.940789	1.3474257	1.4417996	11.54792	Up
HOXA10	-0.76328	9.511923	-6.93967	1.3538938	1.4472705	11.54323	Down
KEAP1	-0.91101	9.242916	-6.93945	1.3551492	1.4478877	11.54232	Down
CTBP1	-0.7025	10.03715	-6.93756	1.3660706	1.4588267	11.53444	Down
NINJ2-AS	-0.97946	6.771157	-6.93038	1.4085354	1.5034232	11.50442	Down
PDCD11	-0.63692	6.491911	-6.92812	1.4221473	1.5164364	11.49498	Down
SEMA7A	-1.04794	5.337458	-6.92767	1.4248774	1.5185894	11.4931	Down
ADAM10	0.932017	8.822096	6.927381	1.4266325	1.5197016	11.49189	Up
LRRC2	-1.14162	10.75676	-6.92922	1.4356690	1.5285653	11.48676	Down
PLXDC2	1.141266	7.949628	6.923895	1.4479730	1.5408973	11.47733	Up
AQP3	1.712978	6.358172	6.925596	1.4625272	1.5556103	11.46881	Up
ERCC1	-0.81704	8.142137	-6.92128	1.4641797	1.5565928	11.46641	Down

LRRC41	-0.65654	7.64364	-6.92106	1.4655621	1.5572873	11.46548	Down
PRELID1	0.885043	8.249603	6.92045	1.4693774	1.5605650	11.46293	Up
PIEZO1	1.679598	6.404968	6.92035	1.4955184	1.5875389	11.44693	Up
CDO1	1.519417	7.552796	6.920084	1.4972103	1.5885453	11.44582	Up
STARD9	1.20654	5.559282	6.915558	1.5003089	1.5910426	11.4425	Up
TMCC1	-0.84975	8.764547	-6.91319	1.5155101	1.6063655	11.43261	Down
RRP7BP	-1.25052	4.656403	-6.91224	1.5216457	1.6120689	11.42865	Down
SLC37A1	-1.00718	5.946365	-6.9105	1.5329414	1.6231485	11.4214	Down
SLC39A10	1.249766	6.899123	6.9104	1.5336238	1.6231485	11.42096	Up
MYO18B	-1.11826	10.23186	-6.91204	1.5345699	1.6233454	11.42093	Down
FAF1	-0.79169	9.396529	-6.90943	1.5399539	1.6282345	11.41692	Down
TIMM23	-0.50199	10.78084	-6.90856	1.5456955	1.6334965	11.41327	Down
INO80B	-0.76612	5.301516	-6.90633	1.5604486	1.6482721	11.40395	Down
BLOC1S1	-0.66988	8.862577	-6.90317	1.5815424	1.6697272	11.39078	Down
VIPR2	-0.63459	9.480529	-6.9026	1.5853980	1.6729709	11.38839	Down
HILPDA	1.026566	6.347404	6.901607	1.5921281	1.6792431	11.38424	Up
ZDHHC20	0.852359	8.300113	6.901325	1.5940360	1.6795965	11.38306	Up
OGDH	-1.27541	9.030068	-6.90288	1.6038710	1.6891260	11.37799	Down
GLCE	0.978991	5.837055	6.899407	1.6071077	1.6917006	11.37505	Up
PAK4	-0.56463	6.579443	-6.89866	1.6122064	1.6962317	11.37195	Down
IL17B	3.97983	4.605436	6.909856	1.6226577	1.7063872	11.36969	Up
MAP1LC3	2.424725	5.461961	6.9018	1.6371992	1.7208317	11.35903	Up
ANKFY1	0.652429	7.731888	6.89487	1.6384414	1.7212903	11.35612	Up
ZSWIM1	-0.71582	6.504096	-6.89465	1.6399466	1.7220245	11.35522	Down
ELMO1	0.842377	5.640678	6.894474	1.6412086	1.7225029	11.35446	Up
ANTKMT	-0.78054	6.149161	-6.89278	1.6530951	1.7341260	11.34738	Down
FXYD6	0.929051	8.606207	6.892369	1.6559743	1.7362935	11.34568	Up
FOXP2	-1.70122	6.014869	-6.89523	1.6638289	1.7436731	11.34232	Down
NMT1	-0.51525	8.231424	-6.8909	1.6663386	1.7454468	11.33956	Down
DCHS1	0.817575	7.279066	6.885881	1.7023341	1.7822771	11.3186	Up
NPTN	0.558807	10.56076	6.881679	1.7330418	1.8127436	11.30106	Up
SNX6	0.585917	9.778009	6.881667	1.7331316	1.8127436	11.30101	Up
PER1	-1.76261	8.161532	-6.8871	1.7425752	1.8217289	11.29785	Down
MRPL48	-0.96869	7.958212	-6.87495	1.7833930	1.8623397	11.27298	Down
DEDD	-0.52948	8.841244	-6.87491	1.7836417	1.8623397	11.27284	Down
RAF1	-0.68773	9.738267	-6.87486	1.7840374	1.8623397	11.27262	Down
FYB1	2.142476	5.882047	6.879461	1.8000124	1.8780979	11.26604	Up
IQSEC3	-0.73609	5.099479	-6.87198	1.8060333	1.8834599	11.2606	Down
CLPP	-0.83498	8.460918	-6.86976	1.8231550	1.9003877	11.25135	Down
ZNF382	1.350078	4.516962	6.868339	1.8342377	1.9110072	11.24541	Up
KLHL2	1.425991	7.424731	6.869122	1.8535106	1.9300552	11.23621	Up
ALG3	-0.70024	6.522093	-6.86578	1.8543278	1.9300552	11.23473	Down
SORBS1	-0.956	10.67978	-6.86355	1.8720078	1.9475082	11.22542	Down
RNF115	-1.00827	10.43724	-6.86222	1.8825680	1.9565881	11.2199	Down
WDR43	-0.91084	6.95325	-6.86175	1.8863622	1.9595781	11.21793	Down
SDCBP	0.845802	11.10197	6.860737	1.8945069	1.9670821	11.2137	Up
RPL18A	0.608522	12.48289	6.860509	1.8963422	1.9680310	11.21275	Up
MSC-AS1	1.512269	5.18144	6.85402	1.9499150	2.0226462	11.18545	Up
LRRC23	-1.1463	4.93261	-6.85244	1.9625372	2.0347509	11.17911	Down
TMEM273	0.996004	7.305663	6.851767	1.9681598	2.0395903	11.1763	Up
FLVCR2	1.527395	5.288469	6.85218	1.9701836	2.0406974	11.17551	Up
DDX51	-0.54372	6.216093	-6.8497	1.9855088	2.0555743	11.1677	Down
THOC3	1.067269	6.55237	6.849423	1.9878706	2.0570223	11.16653	Up
GFPT2	1.026713	6.271111	6.848518	1.9955320	2.0639503	11.16276	Up
ZNF260	0.907865	6.71947	6.848137	1.9987686	2.0662972	11.16117	Up
ANG	1.246118	5.46353	6.847142	2.0072423	2.0740532	11.15702	Up
PHETA2	1.159374	5.051573	6.845115	2.0246102	2.0909877	11.14857	Up
FHIP2B	-0.62996	6.796941	-6.84353	2.0383124	2.1032480	11.14196	Down

CD79B	-0.87195	5.075836	-6.84351	2.0384509	2.1032480	11.14189	Down
EHD4	1.062034	7.369083	6.841981	2.0517572	2.1159551	11.13551	Up
FAM220A	-1.02824	10.80614	-6.84313	2.0535057	2.1167362	11.13512	Down
NEDD1	-1.11027	8.988199	-6.84051	2.0645985	2.1271440	11.12939	Down
HCCS	-0.66077	8.982414	-6.83982	2.0706850	2.1323862	11.12651	Down
ABCC6	-1.43449	5.878204	-6.8382	2.0855673	2.1466769	11.1195	Down
ABCF1	-0.69449	8.733236	-6.83792	2.0874517	2.1475816	11.1186	Down
RFXAP	1.063865	6.652812	6.837588	2.0904280	2.1496081	11.1172	Up
CENPT	-1.0769	3.948672	-6.83485	2.1149188	2.1727001	11.10578	Down
DHX16	-0.77077	7.846001	-6.8336	2.1261860	2.1832251	11.10057	Down
MFSD3	-1.06747	5.013123	-6.83175	2.1429396	2.1993707	11.09287	Down
STMN3	0.811001	6.558945	6.830581	2.1536077	2.2081975	11.08801	Up
CCDC107	-1.0257	6.826332	-6.83024	2.1567690	2.2103777	11.08657	Down
SMARCA1	0.646918	7.87803	6.829379	2.1646307	2.2173709	11.083	Up
FBRSL1	-0.9169	7.132147	-6.82769	2.1801793	2.2322277	11.07598	Down
EMD	-1.06986	7.464743	-6.8267	2.1893802	2.2405741	11.07185	Down
COL11A2	-0.75479	5.189388	-6.82638	2.1924141	2.2426045	11.07049	Down
KATNA1	-0.5914	7.427368	-6.82317	2.2225160	2.2723072	11.05712	Down
DOHH	-0.96918	5.526293	-6.82248	2.2290025	2.2778486	11.05427	Down
COL21A1	1.891011	8.429403	6.828757	2.2317452	2.2795607	11.05521	Up
NSFL1C	-0.65965	9.224887	-6.8206	2.2468787	2.2938445	11.04643	Down
SERPINB1	0.971399	8.045546	6.820497	2.2478774	2.2938445	11.046	Up
POGLUT2	1.461534	5.344331	6.81921	2.2601958	2.3050653	11.04064	Up
H4C8	-1.19191	4.874985	-6.81912	2.2610318	2.3050653	11.04028	Down
MANSC1	0.882399	7.108597	6.81794	2.2724277	2.3155778	11.03535	Up
ANKRA2	0.708629	7.373385	6.817715	2.2745959	2.3166819	11.03441	Up
TMEM250	-1.28876	6.052606	-6.81572	2.2939663	2.3352971	11.0261	Down
APOBEC3I	1.426377	4.100749	6.814621	2.3046834	2.3450895	11.02153	Up
MEG3	2.186176	8.263864	6.824515	2.3122949	2.3517140	11.02179	Up
IFT25	0.723695	8.162054	6.813225	2.3183980	2.3567989	11.01571	Up
CEP70	-0.75793	6.807141	-6.81048	2.3455447	2.3832609	11.0043	Down
PCYT1A	-0.58808	8.844769	-6.80964	2.3539638	2.3906780	11.00079	Down
FOXP4	-1.1827	4.927424	-6.80879	2.3624862	2.3981929	10.99724	Down
BHMT2	-1.65383	6.640206	-6.81113	2.3779325	2.4123995	10.99212	Down
RIN1	-1.01304	5.789622	-6.80717	2.3787402	2.4123995	10.99052	Down
PAN3	0.918934	8.269587	6.806487	2.3856955	2.4183049	10.98766	Up
BTN3A2	1.366459	7.355771	6.808862	2.3873391	2.4188230	10.98781	Up
TNXB	1.084568	9.227586	6.806056	2.3900598	2.4204313	10.98587	Up
FBLN1	1.699779	8.87549	6.812151	2.3944031	2.4236806	10.98622	Up
HARS2	-0.77746	7.691877	-6.80449	2.4059624	2.4342276	10.97936	Down
TXNDC5	0.784813	9.266799	6.804064	2.4103595	2.4375217	10.97757	Up
EVI2B	2.083283	6.402225	6.808646	2.4302100	2.4564328	10.97167	Up
GNA14	1.118445	5.110461	6.799851	2.4538714	2.4791762	10.96003	Up
UBAP1	-0.62576	7.851994	-6.79882	2.4646407	2.4888793	10.95574	Down
GLS	1.06152	8.108712	6.797696	2.4764170	2.4995895	10.95107	Up
LZTFL1	1.077236	6.314603	6.797245	2.4811643	2.5031983	10.94919	Up
RAB1B	-0.83352	8.71538	-6.79677	2.4861551	2.5070491	10.94722	Down
FAM91A1	1.100933	7.08984	6.790745	2.5505803	2.5698071	10.92214	Up
BICC1	1.188351	7.096415	6.790691	2.5511648	2.5698071	10.92191	Up
P2RX2	-0.83254	4.976441	-6.79061	2.5519996	2.5698071	10.92159	Down
KDM5C	-1.08892	6.566696	-6.78711	2.5902313	2.6070764	10.90701	Down
ARHGDIB	0.849745	9.691763	6.786204	2.6002175	2.6158947	10.90324	Up
WLS	0.812036	7.473485	6.785179	2.6115452	2.6260538	10.89898	Up
MRPL34	-1.00075	9.84454	-6.7827	2.6391878	2.6526010	10.88866	Down
JOSD1	0.545471	7.424305	6.781668	2.6507474	2.6629663	10.88437	Up
TAGLN2	1.028987	8.632632	6.78112	2.6569208	2.6679132	10.88209	Up
NUDCD2	0.543106	8.299596	6.77892	2.6818457	2.6916758	10.87294	Up
LIMA1	1.241055	9.437376	6.780557	2.6988970	2.7075172	10.86776	Up

MT1M	2.06401	5.252776	6.783302	2.7054626	2.7128296	10.86647	Up
PCGF3	0.543402	7.740666	6.770721	2.7767759	2.7830305	10.83884	Up
DENND4B	-0.78175	8.775646	-6.76929	2.7936710	2.7986503	10.83289	Down
TRMT1L	-0.79678	8.081275	-6.76858	2.8021024	2.8057807	10.82994	Down
TMEM167	0.528218	8.498961	6.767073	2.8200768	2.8224555	10.82367	Up
ALKBH7	-1.03766	8.844547	-6.76401	2.8568989	2.8579694	10.81095	Down
PPP2R5B	-0.78289	6.504573	-6.76275	2.8722365	2.8719676	10.8057	Down
NFKB2	-0.67317	5.772475	-6.76249	2.8754579	2.8738432	10.80461	Down
GIMAP6	0.63328	8.727939	6.760852	2.8954705	2.8924910	10.79781	Up
ALPK3	-1.00251	9.31644	-6.76071	2.8972237	2.8928893	10.79721	Down
SLC25A4	-0.58653	12.61646	-6.76014	2.9042476	2.8985477	10.79484	Down
RBM20	-1.3422	7.681188	-6.76213	2.9142326	2.9071546	10.7924	Down
CD300A	1.872388	4.861209	6.762375	2.9235739	2.9151117	10.7896	Up
PHF11	0.831403	8.244603	6.758371	2.9260983	2.9162673	10.78749	Up
ZFAT	-0.88757	5.938922	-6.75816	2.9287444	2.9175431	10.78661	Down
LHFPL6	1.430514	9.6451	6.762264	2.9511168	2.9384593	10.7811	Up
PPP1R13B	-0.74803	5.988787	-6.75498	2.9684397	2.9543307	10.77341	Down
IFITM2	1.005527	10.86016	6.756069	2.9710534	2.9555547	10.77298	Up
NNT	-0.84697	10.60681	-6.75287	2.9951919	2.9781801	10.76462	Down
CFD	1.324228	11.42054	6.757758	3.0142208	2.9957062	10.76053	Up
SYK	1.293779	5.442156	6.749044	3.0441399	3.0240344	10.74873	Up
KPNA1	-0.95013	9.6126	-6.74859	3.0499813	3.0284287	10.74685	Down
CA14	-2.46979	4.810688	-6.75376	3.0656063	3.0425287	10.74396	Down
CLEC1A	0.952015	5.223701	6.74679	3.0733640	3.0488114	10.73936	Up
TRAF3IP1	-0.91408	7.267568	-6.74572	3.0873225	3.0612365	10.73492	Down
TECR	-0.86088	9.374847	-6.7452	3.0941007	3.0665338	10.73277	Down
MSANTD4	-0.65661	7.886855	-6.74506	3.0959349	3.0669285	10.73219	Down
LNX1	0.844521	6.797677	6.744647	3.1014119	3.0709298	10.73046	Up
PSMD10	0.580488	8.637832	6.744336	3.1054981	3.0735509	10.72917	Up
PDE7A	-0.79693	9.000108	-6.74333	3.1187142	3.0852014	10.725	Down
MYO5A	1.164723	7.634538	6.742565	3.1289079	3.0938525	10.72181	Up
PRDM1	1.402465	4.853784	6.741821	3.1387926	3.1021903	10.71871	Up
STING1	1.272281	6.695068	6.739152	3.1745009	3.1360310	10.70763	Up
TET3	1.125496	5.614891	6.738607	3.1818363	3.1418243	10.70536	Up
PRPS2	1.331431	5.665711	6.737158	3.2014358	3.1597166	10.69934	Up
NKX3-1	-1.2701	5.685509	-6.7369	3.2049007	3.1616752	10.69828	Down
LRRC28	-0.98144	6.681493	-6.73486	3.2328056	3.1877314	10.68979	Down
UBE2D4	-0.78608	8.783507	-6.73357	3.2505482	3.2037475	10.68442	Down
TMEM115	-0.51265	6.412716	-6.73221	3.2692099	3.2206543	10.67881	Down
RBM38	-1.21649	8.627414	-6.73148	3.3074025	3.2567774	10.6681	Down
LOC10537	1.182031	6.321057	6.727359	3.3371723	3.2830643	10.65865	Up
PDAP1	-1.00422	8.118343	-6.72552	3.3632987	3.3071449	10.651	Down
LYN	1.579018	6.313784	6.728484	3.3647466	3.3071449	10.6516	Up
BAG1	-0.95678	9.70633	-6.72417	3.3825658	3.3231297	10.6454	Down
VAT1	0.862494	8.523736	6.722906	3.4007271	3.3376576	10.64016	Up
AKT3	1.107664	7.29336	6.722815	3.4020417	3.3376576	10.63978	Up
CHMP6	-0.9018	6.629151	-6.71952	3.4499226	3.3830783	10.62608	Down
SIGIRR	-0.58207	5.89041	-6.71734	3.4818910	3.4128604	10.61704	Down
IFITM3	0.938893	11.31385	6.717889	3.4841172	3.4134760	10.61665	Up
NRAS	0.807744	7.899971	6.716992	3.4869948	3.4147288	10.6156	Up
SPTBN1	0.926016	11.22911	6.716674	3.4925718	3.4186228	10.61406	Up
MED15	-0.76916	8.275263	-6.71622	3.4984446	3.4228026	10.61239	Down
UBXN1	-0.6432	8.544338	-6.71384	3.5339352	3.4559427	10.6025	Down
BACE1	0.623847	7.060267	6.711248	3.5728623	3.4924116	10.59176	Up
PSMD12	-0.57859	9.790158	-6.7094	3.6008910	3.5165903	10.5841	Down
RAB5IF	-0.65481	10.86188	-6.70901	3.6068862	3.5208352	10.58247	Down
NME4	-0.78772	7.277075	-6.7061	3.6515812	3.5620774	10.5704	Down
FGF2	1.647482	5.371716	6.708539	3.6541397	3.5620774	10.57059	Up

NFIC	-0.69871	10.99672	-6.70548	3.6612299	3.5673608	10.56782	Down
CCDC69	-1.28931	9.310185	-6.70701	3.6952482	3.5988651	10.56	Down
SOX11	3.240669	4.730748	6.714358	3.7064878	3.6081662	10.55973	Up
ZNF622	-0.63536	8.096006	-6.7002	3.7439545	3.6429786	10.54592	Down
CNKSR1	-1.25046	8.107409	-6.70024	3.7768129	3.6732773	10.53806	Down
RELT	-1.19764	5.162538	-6.69582	3.8140781	3.7078325	10.52774	Down
SLC35A5	0.65701	6.881872	6.695254	3.8231898	3.7137606	10.5254	Up
PHF12	-0.8755	6.03932	-6.69523	3.8236537	3.7137606	10.52528	Down
DYNC2I2	1.001514	5.996827	6.694565	3.8343548	3.7224614	10.52254	Up
TECTA	-1.32	4.30614	-6.69143	3.8856496	3.7705454	10.50952	Down
CLEC3B	1.467385	9.7853	6.695663	3.9185691	3.7995100	10.50335	Up
SMG7	-0.93467	7.141938	-6.6894	3.9190564	3.7995100	10.50113	Down
SEMA4C	0.6817	7.385868	6.687521	3.9503976	3.8281576	10.49332	Up
NPEPL1	-0.62719	7.76142	-6.68521	3.9892310	3.8622845	10.48374	Down
TTC39B	0.953011	4.477229	6.68279	4.0302705	3.9002500	10.47371	Up
SF3A2	-0.97292	5.438565	-6.67686	4.1326699	3.9975346	10.44912	Down
TBX2	-0.75865	5.352581	-6.67595	4.1486595	4.0111847	10.44534	Down
RBM5	0.621055	9.296455	6.675076	4.1639910	4.0241864	10.44172	Up
EBF3	0.743163	7.466151	6.674821	4.1684695	4.0266925	10.44067	Up
CSDE1	-0.52578	12.30408	-6.67425	4.1786293	4.0346819	10.43828	Down
ATN1	-1.00035	6.1544	-6.67338	4.1939154	4.0476116	10.43471	Down
FBXW4P1	-0.74214	3.977493	-6.6724	4.2113314	4.0623685	10.43065	Down
CCNA2	1.513254	4.221761	6.672309	4.2130097	4.0623685	10.43026	Up
FABP7	-4.13	4.412006	-6.68302	4.2295300	4.0751832	10.43035	Down
TUBG1	-0.97987	8.341164	-6.67135	4.2301157	4.0751832	10.42629	Down
ADAMTSL	-2.00907	3.388775	-6.66976	4.3129922	4.1531509	10.40829	Down
MRRF	-0.57852	6.334587	-6.66642	4.3191792	4.1572343	10.40587	Down
TULP3	0.838593	7.465749	6.664583	4.3529451	4.1878470	10.39824	Up
GNB2	-0.65997	8.278714	-6.66254	4.3907983	4.2204622	10.38975	Down
CYTOR	1.558245	7.453419	6.666494	4.3986054	4.2260645	10.3895	Up
TASOR	0.969572	7.909539	6.662003	4.4006980	4.2261740	10.38755	Up
CSNK1G1	0.582504	6.446466	6.661583	4.4085285	4.2317911	10.38581	Up
SYS1	-0.90192	6.670525	-6.66119	4.4158586	4.2369230	10.38418	Down
SUB1	0.703055	11.06002	6.661025	4.4189398	4.2379755	10.3835	Up
TRIM34	1.23762	4.775605	6.65886	4.4595857	4.2750374	10.37452	Up
PRR5	1.586166	5.089826	6.658675	4.4965323	4.3085212	10.36704	Up
NT5DC3	-1.05423	5.859776	-6.65641	4.5060924	4.3157391	10.36436	Down
BZW1	0.64025	9.848584	6.655231	4.5285403	4.3333585	10.35949	Up
SNX25	-0.60024	6.609168	-6.65507	4.5316994	4.3344396	10.35881	Down
GSTO1	-0.80091	10.2948	-6.65254	4.5804305	4.3790884	10.34833	Down
SNHG8	0.844792	8.733408	6.650648	4.6171289	4.4121986	10.34051	Up
MYLIP	0.673682	9.737695	6.649885	4.6320540	4.4244817	10.33735	Up
VEZF1	-0.66391	9.58407	-6.64939	4.6416667	4.4316816	10.33531	Down
GGA2	-0.62372	7.345112	-6.64656	4.6976538	4.4831318	10.32357	Down
CNIH1	0.594857	9.81247	6.64152	4.7987333	4.5775501	10.30271	Up
SLC9A7	0.993239	5.474426	6.64018	4.8259913	4.6014965	10.29716	Up
ETS1	0.861312	9.254001	6.63987	4.8323141	4.6054691	10.29588	Up
CNNM4	-1.04574	7.487749	-6.63915	4.8469755	4.6173819	10.29291	Down
POMT2	-0.6927	5.587127	-6.63662	4.8991788	4.6650317	10.28241	Down
DYNLT2B	1.466233	4.937522	6.635552	4.9212925	4.6840002	10.278	Up
SEPTIN7	0.521511	10.81502	6.634791	4.9371463	4.6969964	10.27485	Up
PSMB9	0.993279	6.785737	6.633673	4.9605242	4.7171361	10.27022	Up
RMC1	-0.57075	6.564555	-6.63167	5.0025946	4.7550252	10.26195	Down
IFI44L	2.196285	7.665613	6.641069	5.0138097	4.7633462	10.26313	Up
NISCH	0.648402	8.477573	6.63105	5.0158094	4.7633462	10.25937	Up
C2CD3	0.950471	5.898005	6.629102	5.0572536	4.8005699	10.2513	Up
TENM4	1.08286	6.228324	6.62719	5.0982584	4.8373436	10.24339	Up
MCM5	0.785408	6.240001	6.626993	5.1025082	4.8392260	10.24258	Up

WEE1	0.816284	7.277448	6.625266	5.1398686	4.8703333	10.23543	Up
MIS18BP1	1.476451	5.341711	6.625473	5.1497834	4.8775642	10.23377	Up
MFAP4	1.512223	8.348574	6.629745	5.1654160	4.8902019	10.2325	Up
PIGB	1.173509	5.594569	6.623688	5.1742378	4.8963833	10.2289	Up
ULK1	-0.84237	6.446291	-6.62125	5.2278690	4.9449436	10.2188	Down
PELO	-0.69918	7.861179	-6.62092	5.2351571	4.9496452	10.21743	Down
TUBB2A	1.293412	10.06246	6.623236	5.2634631	4.9742055	10.21338	Up
SLC25A25	-1.81775	6.348805	-6.62281	5.3267340	5.0317729	10.20249	Down
LMOD2	-0.56019	12.66804	-6.61575	5.3505999	5.0520828	10.19607	Down
MSMO1	0.592224	7.535785	6.614502	5.3789029	5.0765625	10.1909	Up
C1QTNF3	1.810315	6.720485	6.620341	5.3835947	5.0787462	10.19211	Up
RAB8A	0.669683	7.935015	6.61169	5.4431346	5.1326476	10.17927	Up
PXMP2	-1.24942	7.456217	-6.61217	5.4612636	5.1474699	10.17645	Down
NOMO1	-0.77561	8.994236	-6.61004	5.4812539	5.1640327	10.17243	Down
CEROX1	1.958374	7.140951	6.615676	5.4904237	5.1697486	10.17286	Up
PPARGC1/	-1.2699	7.840677	-6.61191	5.4921620	5.1697486	10.17129	Down
AKT1S1	-1.18051	7.408267	-6.60788	5.5313472	5.2043398	10.16352	Down
DTNBP1	-0.72541	6.962869	-6.60708	5.5502014	5.2197799	10.16019	Down
PIGZ	-0.9297	6.775011	-6.60689	5.5545720	5.2215910	10.15942	Down
HDDC2	-0.60052	10.37145	-6.60555	5.5859737	5.2482856	10.15389	Down
SSBP2	0.562654	8.230958	6.605474	5.5878835	5.2482856	10.15356	Up
ERI1	0.669951	6.224666	6.602992	5.6467142	5.2988805	10.1433	Up
SMOX	-0.92417	6.264221	-6.60251	5.6583350	5.3074536	10.14129	Down
FAM218A	1.25622	4.208715	6.602093	5.6681861	5.3143599	10.13958	Up
RUNX1	1.274017	9.53056	6.605151	5.6804579	5.3235288	10.13869	Up
NCF2	1.728041	5.94019	6.605027	5.6834279	5.3239761	10.13818	Up
FEM1A	-1.04624	10.78984	-6.60345	5.6906916	5.3284433	10.13649	Down
CD2BP2	-0.6048	6.536155	-6.60078	5.6995944	5.3344407	10.13417	Down
PPP3R1	-0.87794	7.190024	-6.59988	5.7212931	5.3524037	10.13045	Down
THBS3	0.875468	6.078311	6.599728	5.7250542	5.3535774	10.1298	Up
PPP6R1	-0.62567	6.653904	-6.59933	5.7345875	5.3601452	10.12817	Down
LIMS2	-0.71357	6.729769	-6.59844	5.7563330	5.3781172	10.12447	Down
MYCL	2.184043	5.290156	6.604146	5.7635611	5.3825158	10.1253	Up
SGPP1	1.006696	6.938232	6.59778	5.7723167	5.3883366	10.12175	Up
DYRK1B	-1.47629	7.191529	-6.60087	5.7839405	5.3928483	10.121	Down
PBX3	0.71654	7.087514	6.597283	5.7844243	5.3928483	10.1197	Up
MOB1A	0.795294	8.357007	6.597271	5.7847249	5.3928483	10.11965	Up
MAGED4E	2.219409	4.940361	6.601803	5.8206897	5.4216437	10.11564	Up
PPP1R3F	-0.8576	6.320184	-6.59188	5.9178590	5.5097486	10.09736	Down
MAP2K6	-1.59856	7.754221	-6.59522	5.9843471	5.5678103	10.08847	Down
IL13RA1	0.537209	9.071061	6.589188	5.9854352	5.5678103	10.08624	Up
KRT17	-0.98572	6.364053	-6.58773	6.0222784	5.5996440	10.08023	Down
POLR3D	-1.09422	5.993822	-6.5868	6.0461034	5.6193506	10.07636	Down
ING5	0.778021	5.453427	6.583659	6.1266666	5.6917505	10.06339	Up
NR2F2	0.895574	10.04598	6.583222	6.1379876	5.6997886	10.06159	Up
AGBL5	-0.76926	5.518132	-6.58305	6.1425404	5.7015375	10.06086	Down
TESK1	-0.83832	7.956652	-6.58287	6.1470945	5.7032860	10.06013	Down
FTL	0.812302	12.5898	6.582805	6.1660285	5.7183689	10.05735	Up
UTP11	-0.7547	10.64412	-6.57894	6.2498871	5.7911101	10.04389	Down
ZBTB16	-1.55856	9.568144	-6.58261	6.3103912	5.8446373	10.03651	Down
VPS13D	-0.77439	7.422892	-6.57492	6.3565900	5.8842184	10.02731	Down
LOC10099	1.319937	6.994945	6.57653	6.3586367	5.8842184	10.02758	Up
ARIH1	-0.77007	9.021936	-6.57388	6.3845851	5.9056721	10.02301	Down
IFT80	1.21337	7.572495	6.57439	6.4033163	5.9204341	10.02055	Up
GDF2	-1.22598	4.697329	-6.57256	6.4201541	5.9334336	10.01756	Down
UBE2R2	-0.72696	9.82403	-6.57235	6.4258556	5.9361342	10.0167	Down
PRKAB2	-0.70254	10.33536	-6.57202	6.4350143	5.9420249	10.0153	Down
HOOK2	-0.83984	7.540476	-6.56991	6.4924998	5.9925156	10.00659	Down

TMEM266	-1.6445	5.403955	-6.57196	6.5151978	6.0108680	10.00417	Down
ARMC8	-0.50868	8.692083	-6.56877	6.5236365	6.0146700	10.0019	Down
EMP1	1.412333	9.810395	6.573074	6.5686262	6.0497076	9.997222	Up
TNFSF10	1.160223	9.41412	6.567855	6.6128916	6.0878508	9.989392	Up
SOAT1	0.919192	6.674417	6.56494	6.6299004	6.1008795	9.986079	Up
REV1	-0.52536	8.560742	-6.56355	6.6687358	6.1339733	9.980359	Down
GPRC5C	-0.67974	8.024363	-6.56271	6.6926088	6.1532819	9.976859	Down
ASMTL	0.623723	8.256906	6.56073	6.7486061	6.2020968	9.968699	Up
WDFY3-A	-1.01376	5.155952	-6.5582	6.8208707	6.2658131	9.958268	Down
BLTP2	-0.64164	8.036354	-6.55763	6.8375083	6.2783964	9.955882	Down
GABARAP	-0.93919	8.006327	-6.55684	6.8603097	6.2966263	9.952621	Down
UBE2M	-1.13483	8.654961	-6.55691	6.8912724	6.3210981	9.948608	Down
OSER1	-0.9472	8.099842	-6.55562	6.8956771	6.3210981	9.947585	Down
TMEM123	1.404227	8.685989	6.559055	6.8975469	6.3210981	9.948534	Up
LRSAM1	-0.85505	4.700346	-6.55542	6.9013117	6.3210981	9.946786	Down
OPTN	-1.09482	11.2363	-6.55891	6.9017702	6.3210981	9.947935	Down
ENTREP3	-0.70025	7.065656	-6.55405	6.9412096	6.3544944	9.94114	Down
CCNQ	-0.79868	7.191124	-6.55365	6.9531529	6.3627009	9.939457	Down
ZNF253	-0.93136	6.332924	-6.55071	7.0396145	6.4390615	9.927354	Down
TMEM176	1.724306	7.185776	6.55516	7.0824817	6.4727276	9.923451	Up
AMZ1	-0.68995	4.914307	-6.54917	7.0855553	6.4727669	9.920984	Down
B4GALT3	-0.65832	6.571617	-6.54885	7.0951374	6.4787492	9.91966	Down
TCEAL4	0.538916	9.616357	6.547754	7.1279302	6.5059117	9.915144	Up
RNF217	1.025093	7.154258	6.547182	7.1451237	6.5188189	9.912785	Up
GOT2	-0.99959	11.03228	-6.5468	7.2164250	6.5810591	9.903745	Down
RPS27A	0.586811	12.72281	6.544047	7.2401354	6.5998639	9.899849	Up
CHST12	0.526014	6.819018	6.5422	7.2966604	6.6485526	9.892233	Up
ATAD3B	-0.84191	5.728183	-6.54186	7.3072477	6.6553601	9.890813	Down
ARHGEF6	-0.80423	9.909536	-6.53983	7.3697905	6.7094623	9.882467	Down
CD164	0.607247	10.65715	6.53929	7.3866787	6.7219720	9.880226	Up
HNRNPA3	0.636314	9.391645	6.538755	7.4033441	6.7342685	9.878019	Up
CD24	2.799831	7.267773	6.548941	7.4295267	6.7530232	9.878391	Up
SLC25A11	-1.18798	9.715058	-6.54074	7.4302859	6.7530232	9.875451	Down
CCR5	1.875368	5.328069	6.539575	7.5081894	6.8209234	9.865695	Up
SGMS2	1.424853	6.902468	6.537959	7.5176365	6.8266020	9.864006	Up
CTIF	-0.70347	5.57336	-6.53436	7.5416200	6.8454705	9.859898	Down
VAMP8	1.276477	8.043366	6.535517	7.5952433	6.8912153	9.853948	Up
FAM171A1	0.749604	7.716654	6.532208	7.6102969	6.9019415	9.851021	Up
CLN6	-0.82413	5.128641	-6.52873	7.7224180	6.9992134	9.836699	Down
ZKSCAN2	-0.92846	5.4063	-6.52867	7.7246513	6.9992134	9.836416	Down
MNAT1	-0.79716	7.873108	-6.52856	7.7281516	6.9992134	9.835972	Down
BRAP	-0.84029	7.080939	-6.52848	7.7306604	6.9992134	9.835655	Down
OGFOD1	-0.79285	9.13721	-6.5282	7.7399721	7.0046748	9.834476	Down
FAM53B	-0.72965	6.909474	-6.52802	7.7457827	7.0069643	9.833741	Down
PEX11A	0.82133	6.177497	6.526196	7.8054176	7.0579217	9.826231	Up
SEMA3F	0.711733	7.259643	6.525046	7.8433090	7.0891830	9.821489	Up
TGFBR3	1.044747	8.978328	6.523959	7.8792625	7.1156570	9.817011	Up
CDH23	-0.85388	3.808462	-6.52301	7.9108177	7.1411346	9.813097	Down
ATL1	0.991286	5.296281	6.521815	7.9506959	7.1741006	9.808173	Up
ARRDC2	-1.65228	7.582981	-6.52746	7.9566482	7.1764397	9.809472	Down
IPW	-0.81673	8.121897	-6.52114	7.9732029	7.1883355	9.805405	Down
PCLAF	1.593557	7.586016	6.524456	8.0576578	7.2614116	9.797118	Up
ETNK1	1.285255	7.948263	6.519922	8.1100395	7.3024546	9.789729	Up
TIMM17A	-0.56263	10.60668	-6.51519	8.1756261	7.3584080	9.780857	Down
ZNF385B	-1.41255	7.749094	-6.51681	8.2387126	7.4120650	9.774535	Down
MLX	-0.68918	9.176292	-6.51322	8.2434578	7.4132115	9.772766	Down
PIGM	0.715039	6.047224	6.511507	8.3032349	7.4638254	9.765692	Up
NEURL1B	0.815272	8.70162	6.508505	8.4088098	7.5555473	9.753321	Up

GTPBP6	-0.69175	7.983974	-6.50613	8.4934126	7.6283560	9.743519	Down
CEACAM3	-0.8373	4.407261	-6.50497	8.5348366	7.6623388	9.738755	Down
COPZ2	-0.63906	8.63631	-6.50478	8.5416009	7.6651895	9.73798	Down
ZDHHC4	-0.73915	8.183738	-6.50449	8.5521877	7.6714668	9.736767	Down
GALNT16	1.544585	5.312281	6.506124	8.5592314	7.6745619	9.736603	Up
NOMO2	-0.60528	10.19024	-6.50342	8.5906554	7.6962759	9.732373	Down
TMEM143	-1.12654	8.74971	-6.5044	8.5978385	7.6972891	9.731969	Down
TOP1	0.584169	8.242463	6.502621	8.6195458	7.7124532	9.729086	Up
MAD2L1B	-0.78337	7.18609	-6.50233	8.6302276	7.7187773	9.727873	Down
DLGAP1-/	1.482862	5.515777	6.501946	8.6883347	7.7642448	9.721729	Up
NOS1	-2.38909	7.717365	-6.51125	8.7005810	7.7719368	9.723727	Down
LMCD1	-0.90053	10.94156	-6.49885	8.7574832	7.8194952	9.713542	Down
MDFIC	0.66883	9.656176	6.495772	8.8714989	7.9179888	9.700877	Up
SGCA	-1.0699	9.455369	-6.49452	8.9514153	7.9826442	9.692406	Down
SLC29A2	-1.32702	7.750757	-6.49385	9.0490461	8.0663408	9.682458	Down
LOC15476	1.12602	4.407302	6.487409	9.1890363	8.1877112	9.666447	Up
PLXDC1	1.235091	7.700569	6.487227	9.2677978	8.2488111	9.658742	Up
SH3BP4	0.929651	7.56042	6.485329	9.2697363	8.2488111	9.657887	Up
TRIQK	1.180552	5.874019	6.485166	9.2760978	8.2488111	9.657215	Up
HMCN2	-0.94888	6.131354	-6.48515	9.2769193	8.2488111	9.657129	Down
ARHGEF1(	0.905248	7.156487	6.484704	9.2941235	8.2606696	9.655315	Up
ARPC5L	-0.63606	9.475661	-6.48295	9.3629880	8.3184151	9.648088	Down
MFGE8	-0.93426	7.443692	-6.48227	9.3896290	8.3386152	9.645306	Down
PTPN3	-1.60441	8.809405	-6.48734	9.4156546	8.3582524	9.644612	Down
MASP1	-2.3816	6.457371	-6.48872	9.4680280	8.4012523	9.640131	Down
MRPL11	-0.90939	7.93869	-6.47859	9.5359178	8.4579789	9.630171	Down
CD58	0.671561	7.971124	6.478062	9.5573121	8.4734360	9.627977	Up
DHTKD1	-0.81437	7.985522	-6.47604	9.6389045	8.5422292	9.619655	Down
TSPAN4	0.872701	7.91128	6.475847	9.6466916	8.5455844	9.618865	Up
CACNG8	-0.80349	4.31821	-6.47525	9.6709480	8.5635202	9.616406	Down
MIR1244-	0.758353	9.820123	6.474785	9.6898367	8.5738944	9.614496	Up
STAG2	0.644533	9.728412	6.474764	9.6906925	8.5738944	9.61441	Up
ZFP90	0.776437	7.703996	6.472633	9.7778554	8.6474301	9.605644	Up
E2F6	-0.6708	7.020026	-6.47222	9.7947696	8.6588034	9.603952	Down
KIAA0232	-0.71781	9.643381	-6.47133	9.8314562	8.6876392	9.600292	Down
UBE2L6	0.847223	7.833949	6.470204	9.8782085	8.7253423	9.595648	Up
BAZ1B	-0.59851	7.918017	-6.47003	9.8854748	8.7281509	9.594928	Down
DUSP15	-1.05421	4.545551	-6.46941	9.9113755	8.7474032	9.592367	Down
CTNNA1	0.549095	10.34138	6.468727	9.9396982	8.7687763	9.589573	Up
ACSL4	0.995982	6.748496	6.468108	9.9655637	8.7879648	9.587029	Up
CAMTA1	-0.5379	10.60731	-6.46796	9.9719130	8.7881505	9.586406	Down
CCDC13	-1.68243	7.671522	-6.4736	9.9740036	8.7881505	9.588211	Down
CHMP1A	-0.69087	7.09632	-6.46666	1.0026524	8.8251647	9.581059	Down
ZNF700	0.903591	5.710867	6.466612	1.0028408	8.8251647	9.580875	Up
ITM2A	1.359579	9.860984	6.471358	1.0051785	8.8420934	9.580468	Up
IFNLR1	-1.73698	5.235037	-6.46828	1.0100674	8.8814411	9.57504	Down
KLF5	1.619869	7.878455	6.470452	1.0106553	8.8829532	9.575286	Up
ZNF266	0.731697	7.792825	6.464521	1.0116897	8.8883876	9.572275	Up
CFAP97	0.921454	8.284351	6.463586	1.0156725	8.9197101	9.568429	Up
CCDC85C	-0.7736	7.904202	-6.46342	1.0163725	8.9221884	9.567755	Down
PPM1B	-0.62657	10.09188	-6.46316	1.0174739	8.9281876	9.566695	Down
SMIM14	0.801318	8.619431	6.46165	1.0239667	8.9814709	9.560468	Up
SYAP1	-0.59929	7.715535	-6.46121	1.0258806	8.9945335	9.55864	Down
NF2	-0.59518	7.649575	-6.46111	1.0262982	8.9945335	9.558242	Down
PDE4A	-0.76663	8.135893	-6.45987	1.0316643	9.0378536	9.553137	Down
TMEM11	-0.77747	8.706043	-6.45948	1.0333447	9.0484716	9.551544	Down
ZNF593	-0.91303	7.440216	-6.45932	1.0340180	9.0484716	9.550906	Down
EPHX1	-1.02631	8.521523	-6.45913	1.0348801	9.0484716	9.55009	Down

IFIT5	0.910457	6.847796	6.459121	1.0349031	9.0484716	9.550068	Up
SERTAD2	0.561272	8.573563	6.459045	1.0352323	9.0484716	9.549757	Up
GALNT10	0.985904	6.148104	6.458909	1.0358237	9.0484716	9.549198	Up
SPDYE2	-0.97595	6.20291	-6.4584	1.0380238	9.0615041	9.547121	Down
TBC1D12	0.554936	6.747683	6.458323	1.0383760	9.0615041	9.546789	Up
APOO	-0.92614	9.288835	-6.45827	1.0386067	9.0615041	9.546572	Down
TNRC6C	-0.71299	7.276346	-6.45635	1.0469971	9.1272514	9.538696	Down
ABCB6	-0.83741	6.828881	-6.45546	1.0509293	9.1554635	9.535026	Down
PRKCQ	-0.99034	10.28285	-6.456	1.0510907	9.1554635	9.53508	Down
L3MBTL1	-0.93127	5.617262	-6.45529	1.0516822	9.1568815	9.534325	Down
DYNC1I1	-1.34743	6.115049	-6.4525	1.0665714	9.2827352	9.520762	Down
RRM2	2.075125	4.317073	6.45687	1.0698445	9.3074294	9.519569	Up
LTB	-1.03797	6.033525	-6.4507	1.0721674	9.3220767	9.515442	Down
EML2	-0.74059	5.405565	-6.45065	1.0724010	9.3220767	9.515229	Down
WASF1	1.476675	5.069579	6.450969	1.0746024	9.3374119	9.513509	Up
NR4A1	-1.79329	7.101778	-6.45423	1.0817597	9.3957810	9.508727	Down
CTBP1-DT	-0.82092	5.867953	-6.44837	1.0827175	9.4002767	9.505858	Down
CNDP2	0.753373	7.649291	6.447031	1.0888042	9.4492815	9.50037	Up
RHOJ	0.940865	7.610275	6.446692	1.0903518	9.4588691	9.49898	Up
SOX8	2.514406	5.821749	6.453501	1.0969778	9.5124855	9.495977	Up
ARHGEF4(	0.634551	7.618262	6.444902	1.0985793	9.5225069	9.491622	Up
ALG8	0.50779	6.787929	6.444188	1.1018764	9.5472119	9.488689	Up
PDE5A	1.104719	8.55137	6.44381	1.1065399	9.5833511	9.484778	Up
PMP22	1.067139	10.53841	6.445434	1.1069448	9.5833511	9.485028	Up
HLA-C	1.129697	11.27514	6.445791	1.1099631	9.6055888	9.48272	Up
HAUS4	0.60159	7.23635	6.442115	1.1115077	9.6150600	9.480171	Up
LRRC37A7	-2.02766	4.308189	-6.44732	1.1133991	9.6275215	9.4805	Down
MXRA8	1.015126	9.235036	6.441238	1.1156059	9.6427000	9.476568	Up
NBL1	1.141467	7.849575	6.441518	1.1172316	9.6528454	9.475366	Up
MTHFD2	0.853478	9.274056	6.438663	1.1277323	9.7389473	9.465987	Up
ARHGAP2	1.513698	4.615576	6.439721	1.1281091	9.7389473	9.466064	Up
PLEKHB1	-1.34725	4.00956	-6.43572	1.1417587	9.8528016	9.453888	Down
CES2	0.714288	8.983335	6.434397	1.1481060	9.9035743	9.448462	Up
MED24	-0.67259	7.862906	-6.43276	1.1560162	9.9677814	9.441742	Down
RNF10	-0.69565	9.976204	-6.43252	1.1571772	9.9737659	9.440759	Down
THAP9	-0.95229	6.884743	-6.43242	1.1576517	9.9738304	9.440358	Down
HACD3	0.650935	8.289417	6.429466	1.1721075	1.0092465	9.428212	Up
SLC2A11	-0.79404	4.660498	-6.42941	1.1723666	1.0092465	9.427996	Down
DCDC2B	-0.85787	5.078829	-6.42903	1.1742360	1.0104485	9.426436	Down
LURAP1L	0.856119	5.439861	6.428933	1.1747339	1.0104699	9.426022	Up
RPS6KA4	-0.94598	6.469977	-6.42789	1.1798901	1.0144965	9.421735	Down
EPN1	-0.75567	7.136398	-6.42713	1.1836546	1.0173237	9.418617	Down
SOHLH2	1.69207	4.793948	6.428585	1.1900616	1.0224189	9.414312	Up
UBE2E2	0.70152	7.973187	6.425675	1.1908996	1.0227275	9.412645	Up
UHRF1	1.654662	5.219515	6.42807	1.1925026	1.0236925	9.412297	Up
FGD2	1.493484	5.47962	6.426513	1.1944574	1.0249587	9.410278	Up
TSEN15	-0.95529	8.246367	-6.42395	1.1995737	1.0289355	9.405542	Down
ZNF580	-0.76293	6.090903	-6.42314	1.2036426	1.0320112	9.402228	Down
SLC33A1	0.726674	6.172203	6.422203	1.2083735	1.0356517	9.398389	Up
HECTD3	-0.6547	7.779262	-6.42124	1.2132711	1.0394322	9.39443	Down
KLHL40	-1.14936	10.14453	-6.42155	1.2256578	1.0496230	9.385466	Down
ALDH2	-1.38194	10.15745	-6.424	1.2276841	1.0509369	9.384864	Down
MAP7D1	-0.70453	9.809446	-6.41761	1.2319005	1.0541238	9.379517	Down
ARMC7	-0.92388	7.797691	-6.41719	1.2340411	1.0555326	9.377818	Down
ZNF451	1.017656	8.107433	6.416904	1.2355333	1.0563859	9.376636	Up
TPP1	0.719905	9.381577	6.416712	1.2365281	1.0568134	9.375848	Up
AXIN1	-0.65304	5.14344	-6.41549	1.2429044	1.0618381	9.370814	Down
SMIM29	-0.78871	5.637609	-6.41349	1.2533365	1.0703223	9.362635	Down

LMO1	-1.66696	6.469708	-6.41705	1.2554392	1.0716894	9.362408	Down
MAGI3	0.751971	5.778468	6.410692	1.2681405	1.0816671	9.351143	Up
ADCY9	-0.81552	9.045903	-6.40885	1.2779601	1.0896076	9.343594	Down
ANPEP	2.2952	4.61878	6.412965	1.2857053	1.0957739	9.339668	Up
CD300LG	1.060259	7.741079	6.405825	1.2942851	1.1026462	9.331173	Up
DHX40	0.534922	8.287225	6.405233	1.2975014	1.1049455	9.328744	Up
PPP1R14B	-0.71926	8.73162	-6.40488	1.2994356	1.1061516	9.327286	Down
TRPC4AP	-0.51567	7.636455	-6.40288	1.3103795	1.1150233	9.319079	Down
OTUD7B	-0.71967	7.422431	-6.4023	1.3135357	1.1172639	9.316724	Down
IDE	-0.85545	8.79499	-6.39924	1.3305018	1.1312443	9.304166	Down
WHAMM	-0.71624	7.190589	-6.39866	1.3337619	1.1335649	9.301771	Down
DMAC2L	-0.65868	8.82023	-6.39789	1.3380356	1.1367448	9.29864	Down
C16orf54	1.656089	4.465596	6.40018	1.3402686	1.1381891	9.297972	Up
DNMT3A	-0.65967	7.433319	-6.39687	1.3438015	1.1407358	9.294433	Down
FAM20A	1.302176	4.635456	6.394989	1.3544220	1.1492946	9.286729	Up
MTARC2	-0.78167	7.552454	-6.39469	1.3561202	1.1502786	9.285503	Down
SNHG12	1.382886	7.371972	6.397776	1.3572199	1.1507544	9.285887	Up
PTS	-0.72966	9.391254	-6.39364	1.3620786	1.1541482	9.281213	Down
MICALL1	-0.61074	5.994699	-6.3936	1.3623034	1.1541482	9.281051	Down
SRGAP2	1.239031	6.171804	6.393332	1.3638632	1.1550115	9.279932	Up
ZNF300	0.926862	4.619912	6.39169	1.3732747	1.1625209	9.273202	Up
EN1	1.266789	6.809438	6.392339	1.3756913	1.1641052	9.271866	Up
UBE3B	-0.71549	8.171884	-6.38863	1.3909883	1.1765833	9.260661	Down
PHKB	-0.70505	9.756482	-6.38659	1.4029134	1.1862005	9.252308	Down
SNHG19	1.046857	8.661543	6.385288	1.4106072	1.1918511	9.246957	Up
RSPO3	-1.89071	8.073651	-6.39142	1.4107125	1.1918511	9.249095	Down
SMC1A	0.733639	7.62461	6.384745	1.4138219	1.1940058	9.244729	Up
PRCC	-0.68748	7.078582	-6.38422	1.4169257	1.1961541	9.242583	Down
ADA	0.747938	6.584809	6.384113	1.4175715	1.1962264	9.242138	Up
NSUN4	-0.60831	6.75516	-6.38361	1.4205557	1.1982713	9.24008	Down
MYADM	1.047044	9.651935	6.382187	1.4354354	1.2103446	9.230267	Up
METTL8	0.530266	7.369655	6.379294	1.4464744	1.2191713	9.222388	Up
RNF213	0.945752	7.200894	6.375874	1.4673411	1.2359886	9.208374	Up
LMF1	2.063162	6.012881	6.381315	1.4675846	1.2359886	9.210186	Up
NRG1	-1.34635	5.773043	-6.37541	1.4702009	1.2377040	9.206469	Down
SVEP1	1.201904	7.296218	6.376065	1.4726950	1.2393152	9.205193	Up
TMEM135	-0.85543	8.541263	-6.37431	1.4769633	1.2424176	9.201978	Down
GYPC	-0.82506	9.333411	-6.36995	1.5041826	1.2648162	9.184111	Down
PCYOX1L	0.941282	5.226724	6.367397	1.5203521	1.2779095	9.173649	Up
FLRT3	-1.31906	6.129534	-6.3668	1.5272235	1.2831802	9.16941	Down
PPP1R12C	-0.85558	5.789758	-6.36561	1.5317458	1.2861823	9.166344	Down
RUNX1-IT	3.326611	5.601422	6.375838	1.5320011	1.2861823	9.169885	Up
ZSCAN16-	-0.80747	6.162755	-6.36455	1.5385661	1.2911864	9.161998	Down
FAM200C	0.84007	6.295337	6.363784	1.5435255	1.2948397	9.158849	Up
ZNF736	0.866605	5.199159	6.363124	1.5477960	1.2979123	9.156146	Up
NOXA1	-0.90237	5.363229	-6.3628	1.5499002	1.2991668	9.154817	Down
HUS1	-0.76335	7.049685	-6.36157	1.5578697	1.3053347	9.149799	Down
LINC0239	1.850013	4.253485	6.361875	1.5770799	1.3209126	9.138977	Up
UPF2	-0.70955	8.133357	-6.35822	1.5798750	1.3227350	9.136076	Down
RUNX1T1	0.972848	8.157078	6.357031	1.5877701	1.3288242	9.131199	Up
FHIP1A	-1.56643	6.803072	-6.35983	1.5906311	1.3306972	9.130606	Down
ELL	-0.8928	5.020321	-6.35636	1.5922393	1.3315210	9.128449	Down
CBX6	0.795751	7.863989	6.353849	1.6090524	1.3450544	9.118172	Up
COL5A3	1.206504	6.968574	6.353859	1.6123778	1.3473069	9.116335	Up
POLD4	-0.96861	7.063714	-6.35311	1.6140448	1.3481724	9.115142	Down
UBOX5	-0.6645	5.685659	-6.35263	1.6172790	1.3503458	9.113183	Down
PPP2R2A	-0.57337	9.324339	-6.35209	1.6209435	1.3528766	9.110969	Down
FZR1	-0.75058	5.553719	-6.35113	1.6274660	1.3577898	9.10704	Down

RASL12	-0.79638	6.807661	-6.34793	1.6493644	1.3753862	9.093964	Down
FUT11	0.542707	7.359374	6.347865	1.6498453	1.3753862	9.093679	Up
DPP7	-0.7777	6.901704	-6.34619	1.6614716	1.3845381	9.086809	Down
FBXL17	-0.75685	8.506432	-6.34546	1.6665274	1.3882095	9.083837	Down
PTGR1	0.959027	8.003631	6.345254	1.6679571	1.3888588	9.082998	Up
PPP5D1P	0.879916	5.086144	6.344173	1.6755184	1.3946112	9.078573	Up
AGBL1	-1.38445	7.096112	-6.34579	1.6838402	1.4009918	9.074745	Down
FAM13A	0.891311	9.627543	6.342477	1.6874465	1.4034456	9.071633	Up
LINC0084	2.366153	8.561001	6.349889	1.7068978	1.4190706	9.064106	Up
FAM78A	-1.08381	8.084923	-6.33954	1.7086530	1.4199771	9.059434	Down
MYO1F	1.20569	6.128496	6.339232	1.7105003	1.4209594	9.058359	Up
ADCK1	-0.85904	5.672242	-6.33863	1.7148047	1.4239813	9.0559	Down
STX6	0.557495	6.771602	6.337972	1.7195392	1.4273579	9.053203	Up
CYB5D1	-0.68333	6.66551	-6.33734	1.7241067	1.4305932	9.050608	Down
UBR2	-0.53523	9.355087	-6.33619	1.7323968	1.4369138	9.045916	Down
RBBP9	0.595318	6.597025	6.336018	1.7336420	1.4373885	9.045213	Up
SNX7	1.686668	8.232258	6.340906	1.7372705	1.4398379	9.045126	Up
SOCS3	2.902181	6.933597	6.344235	1.7475560	1.4472392	9.041073	Up
NAALAD2	1.60274	4.90923	6.331606	1.7835670	1.4759173	9.018307	Up
GBF1	-1.07646	7.077352	-6.32811	1.7919509	1.4822807	9.012853	Down
ARK2N	-0.54493	8.499105	-6.32783	1.7940441	1.4834376	9.011711	Down
PPP6C	-0.5531	9.897113	-6.32596	1.8080940	1.4944764	9.00408	Down
CLIC2	0.97209	6.759925	6.323973	1.8231722	1.5063564	8.995956	Up
ALDH1L2	1.926503	4.242514	6.327212	1.8311424	1.5123565	8.993251	Up
TCEAL8	1.033229	7.750369	6.322232	1.8364853	1.5161829	8.98884	Up
HIBADH	-0.71797	10.07862	-6.32182	1.8396574	1.5182149	8.987152	Down
PDK2	-1.23833	8.827955	-6.32366	1.8497909	1.5259881	8.98293	Down
CALML4	0.952784	5.536995	6.319332	1.8588719	1.5328875	8.976988	Up
CSGALNA	1.140367	7.943835	6.32019	1.8607016	1.5338041	8.976425	Up
AIFM1	-0.82077	8.00393	-6.31893	1.8620011	1.5342188	8.975343	Down
DDX52	0.728612	6.537835	6.318847	1.8626413	1.5342188	8.975007	Up
PDGFC	0.952014	8.835749	6.318631	1.8643287	1.5350166	8.974121	Up
TLCD3A	0.930617	4.64156	6.317163	1.8757959	1.5438631	8.968123	Up
USP18	0.921989	5.424319	6.313045	1.9083511	1.5700525	8.951293	Up
DKK2	1.74282	6.140985	6.31483	1.9338604	1.5904270	8.94012	Up
IER5	-0.79057	9.388856	-6.30948	1.9369494	1.5923543	8.936744	Down
PPHLN1	0.712252	8.757645	6.307854	1.9501805	1.6026147	8.930085	Up
HLA-A	0.911663	11.53112	6.308214	1.9597174	1.6098325	8.925873	Up
TNXA	0.979077	9.229971	6.305042	1.9732150	1.6202970	8.9186	Up
B4GALT2	-0.56506	6.849159	-6.30449	1.9777925	1.6234317	8.916334	Down
MYOZ3	-0.98886	10.24916	-6.30494	1.9826001	1.6267527	8.914337	Down
ADAR	0.610843	9.105649	6.302907	1.9908883	1.6329260	8.909879	Up
SLC30A1	1.012877	7.924706	6.302457	1.9946341	1.6353702	8.908041	Up
OSBPL9	-0.57996	9.540559	-6.30146	2.0029213	1.6415347	8.903986	Down
LYSMD2	-1.04073	8.44158	-6.29986	2.0163496	1.6519062	8.89745	Down
GOLGA4	-0.93055	10.60558	-6.2995	2.0227285	1.6564968	8.894504	Down
DGLUCY	-0.70468	9.552946	-6.299	2.0236665	1.6566297	8.893908	Down
ASAP2	0.959367	7.632312	6.297432	2.0369232	1.6668431	8.887522	Up
SEZ6L2	-0.89825	5.98563	-6.29728	2.0381871	1.6672386	8.886915	Down
PHLDB2	1.34579	8.543654	6.299041	2.0500290	1.6761813	8.882403	Up
SPRY1	0.593987	8.890047	6.295819	2.0506890	1.6761813	8.880934	Up
TNKS2	-0.57885	9.844589	-6.29547	2.0536908	1.6779926	8.879504	Down
KIAA1656	-0.98222	4.695849	-6.29367	2.0691215	1.6899540	8.872183	Down
AP1G1	-0.54828	8.425162	-6.29235	2.0805752	1.6986592	8.866784	Down
P2RX7	1.905856	4.928803	6.296956	2.0846399	1.7013274	8.866737	Up
TMEM37	1.527866	5.543578	6.293982	2.0854908	1.7013718	8.865282	Up
RAB23	0.991989	8.470628	6.291208	2.0905362	1.7048367	8.862113	Up
UBXN6	-0.67001	9.183435	-6.29049	2.0968183	1.7093071	8.859178	Down

FKBP5	-2.20757	9.249627	-6.29852	2.1135380	1.7222794	8.855057	Down
CDC42BPI	-1.00445	6.032397	-6.28767	2.1216006	1.7281901	8.847687	Down
DENND1A	-0.60045	6.196319	-6.2867	2.1302789	1.7345977	8.843695	Down
FGFR2	-2.11023	4.545596	-6.28874	2.1591174	1.7567402	8.832483	Down
DPY19L1	1.180811	5.804016	6.282894	2.1643401	1.7603189	8.828182	Up
CXCL14	1.432844	11.19171	6.287209	2.1729566	1.7666542	8.826234	Up
CSTA	2.135591	5.763048	6.285376	2.1896023	1.7795101	8.81877	Up
BANF1	-0.83705	10.52755	-6.27967	2.1936193	1.7820967	8.815041	Down
GRWD1	-0.66323	5.913657	-6.27785	2.2103352	1.7949939	8.807617	Down
ST3GAL5	-0.63774	7.608092	-6.27772	2.2116023	1.7953402	8.807057	Down
LPIN1	-0.98262	9.44447	-6.2768	2.2200613	1.8015224	8.803324	Down
SMC4	0.702874	8.226056	6.276238	2.2252873	1.8050774	8.801024	Up
PLAC9	1.192524	7.926608	6.276451	2.2419079	1.8178689	8.794484	Up
POLR3K	-1.06241	7.416164	-6.27265	2.2587987	1.8308699	8.786407	Down
ZMIZ1	0.538054	8.953392	6.270285	2.2812309	1.8483509	8.776744	Up
MME	0.7765	8.173986	6.269069	2.2928233	1.8567224	8.771787	Up
PPP1R12B	-0.60897	9.953765	-6.26902	2.2933016	1.8567224	8.771583	Down
GRPEL1	-0.55587	8.698746	-6.26853	2.2979371	1.8597703	8.769609	Down
MAGED1	1.013886	9.804909	6.269298	2.2993134	1.8601794	8.769358	Up
TSPAN6	0.658931	7.21237	6.266989	2.3127951	1.8696699	8.763306	Up
VEGFC	0.731108	6.35868	6.265778	2.3245101	1.8784293	8.758366	Up
DNAJB2	-0.65906	7.230624	-6.26494	2.3326554	1.8842986	8.754945	Down
NT5DC1	-0.50618	8.062465	-6.26446	2.3373590	1.8873843	8.752975	Down
LRWD1	-0.7844	6.260101	-6.26399	2.3419312	1.8890680	8.751065	Down
NRP2	1.105117	5.884464	6.263975	2.3420462	1.8890680	8.751017	Up
GOLGA1	-0.57514	6.708559	-6.26397	2.3420976	1.8890680	8.750995	Down
TMTC2	1.098282	5.670877	6.26382	2.3435594	1.8895335	8.750385	Up
DTWD1	0.982778	7.245716	6.261295	2.3683554	1.9088051	8.740093	Up
ATL3	1.008434	8.842805	6.258273	2.3983843	1.9322781	8.727773	Up
LINC0223	-1.14703	4.552475	-6.25443	2.4370908	1.9608802	8.712119	Down
HLA-F	0.946992	9.910006	6.254386	2.4375583	1.9608802	8.711931	Up
ASAP1	0.964589	8.315901	6.252957	2.4521156	1.9715067	8.706109	Up
ZFP69	0.914058	3.808276	6.252908	2.4526141	1.9715067	8.70591	Up
PFKFB2	-1.50987	6.028278	-6.25337	2.4737912	1.9877815	8.698439	Down
MAP7	1.554771	4.770408	6.251194	2.4910718	2.0009143	8.691447	Up
GEMIN5	-0.58163	7.761532	-6.24626	2.5215504	2.0246344	8.678807	Down
VAMP2	-0.98944	6.891105	-6.24577	2.5266582	2.0279732	8.676829	Down
THOC6	-0.98947	4.96037	-6.24421	2.5431358	2.0396655	8.670473	Down
SH2D3C	-0.86646	6.512784	-6.24337	2.5520478	2.0441465	8.667053	Down
AHI1	1.025435	6.984723	6.243348	2.5522800	2.0441465	8.666964	Up
WDR74	-0.7067	6.61616	-6.24332	2.5525512	2.0441465	8.66686	Down
CCDC106	-0.87534	5.660976	-6.2427	2.5591773	2.0486847	8.664325	Down
EFNA5	1.351909	4.650419	6.242318	2.5632530	2.0507093	8.662769	Up
CFB	1.899385	5.482743	6.247461	2.5636267	2.0507093	8.664549	Up
MYH11	-1.81453	9.823994	-6.24747	2.5896468	2.0707479	8.655579	Down
NPRL3	-0.6051	6.807016	-6.23899	2.5990533	2.0774918	8.649208	Down
IRF2BPL	0.615264	9.949049	6.238249	2.6070688	2.0824150	8.646198	Up
SEPTIN2	0.571582	10.70912	6.23824	2.6071625	2.0824150	8.646163	Up
YJU2	-0.66169	5.848206	-6.23751	2.6151117	2.0876791	8.643186	Down
NDUFAF5	-0.95843	8.460977	-6.23745	2.6157081	2.0876791	8.642963	Down
ACOX2	-0.87127	6.961981	-6.23643	2.6268415	2.0957818	8.638811	Down
BOLL	-1.16841	4.569889	-6.23592	2.6325276	2.0995341	8.636697	Down
USP3	0.600781	7.246444	6.234642	2.6465290	2.1099129	8.63151	Up
NRM	0.812816	5.935746	6.233454	2.6596493	2.1195817	8.626676	Up
NUDT5	0.780017	6.400429	6.233108	2.6634781	2.1210502	8.625269	Up
ODF2L	1.288436	4.80912	6.232952	2.6652128	2.1216405	8.624633	Up
BOK	1.34825	5.676755	6.233177	2.6680204	2.1230842	8.623781	Up
CACNA1S	-0.89241	9.961416	-6.23079	2.6892971	2.1392181	8.615838	Down

EPHA3	1.904647	5.633686	6.234757	2.7025874	2.1489897	8.612935	Up
TBC1D13	-0.50295	7.014492	-6.22852	2.7149059	2.1563764	8.606572	Down
NOP53	0.518346	11.81128	6.227598	2.7252880	2.1638179	8.60284	Up
TUSC2	-0.81807	7.286377	-6.22586	2.7450432	2.1786931	8.595779	Down
SEPTIN8	0.903986	6.815185	6.224197	2.7641487	2.1922186	8.588999	Up
LRRC66	-1.02315	5.097919	-6.22417	2.7644144	2.1922186	8.588905	Down
LINC0109:	1.984689	7.817002	6.231623	2.7651639	2.1922186	8.591439	Up
DOCK10	1.835905	4.751674	6.227641	2.7665229	2.1924823	8.58952	Up
PITX1	-2.78483	4.812534	-6.23273	2.7771705	2.2001041	8.588001	Down
GNPTAB	-0.58197	8.209991	-6.22153	2.7950182	2.2134223	8.578141	Down
USP19	-0.75507	7.948131	-6.22137	2.7968742	2.2140711	8.577492	Down
PLXND1	0.99616	7.961507	6.219328	2.8207292	2.2321280	8.56919	Up
MTMR2	0.555409	7.691567	6.219109	2.8233080	2.2333412	8.568296	Up
AFG3L2	-0.53266	11.12608	-6.21889	2.8259242	2.2345830	8.567391	Down
ARHGAP1	0.591591	7.845836	6.21764	2.8406159	2.2453691	8.562322	Up
CADM2	1.122548	6.463484	6.217026	2.8478784	2.2502770	8.559825	Up
PACS2	-0.65873	8.483376	-6.21646	2.8545658	2.2547269	8.557533	Down
OGFR	-0.77287	5.866149	-6.2159	2.8613073	2.2592163	8.555227	Down
TBC1D8B	1.098995	4.887552	6.21571	2.8635244	2.2601314	8.554469	Up
LINC0303:	-0.82552	3.926787	-6.21383	2.8860596	2.2770765	8.546806	Down
ALG5	0.560809	8.585526	6.212793	2.8984927	2.2860416	8.542604	Up
AKAP12	1.266925	8.883436	6.211177	2.9550399	2.3297801	8.52485	Up
ZNF784	-1.14195	6.615959	-6.20788	2.9583692	2.3315443	8.522616	Down
RAC2	1.27236	6.524478	6.208228	2.9667596	2.3372944	8.520234	Up
RBM3	1.411512	9.849375	6.210891	2.9841131	2.3500991	8.516053	Up
SCRN1	1.374196	6.244405	6.207375	2.9857543	2.3505249	8.51425	Up
HAGH	-0.73264	8.578821	-6.20333	3.0147928	2.3725108	8.504148	Down
PECAM1	1.094405	9.296384	6.204514	3.0225010	2.3777008	8.502324	Up
TEX264	-0.54324	6.300372	-6.20189	3.0329778	2.3850640	8.498269	Down
JSRP1	-1.13697	6.617161	-6.2014	3.0390992	2.3889982	8.496298	Down
UBE2D3-A	-0.89381	6.28774	-6.19989	3.0582764	2.4031887	8.49015	Down
NRBP2	0.743159	7.22781	6.19881	3.0720479	2.4131224	8.485758	Up
ANXA3	-1.29307	7.03241	-6.20052	3.0770927	2.4161965	8.484945	Down
SCAF1	-1.19375	4.320805	-6.19824	3.0793361	2.4170695	8.483442	Down
NAA30	-0.69731	9.023883	-6.1968	3.0978385	2.4306993	8.477586	Down
ARL6IP4	-0.59567	8.86823	-6.19599	3.1082976	2.4380102	8.474292	Down
FGD5	0.726844	6.592538	6.195226	3.1181716	2.4448572	8.471191	Up
COX4I2	-0.71465	6.068336	-6.19395	3.1347450	2.4562301	8.46601	Down
FRAT1	-0.86355	6.020195	-6.19393	3.1349767	2.4562301	8.465938	Down
METRNL	0.954128	6.648493	6.192461	3.1542234	2.4702099	8.459955	Up
STMN2	3.6515	4.392078	6.201923	3.1551328	2.4702099	8.463248	Up
ATP1B4	1.092221	10.61436	6.194397	3.1682943	2.4796053	8.456734	Up
FXN	-0.7501	7.348131	-6.18935	3.1952388	2.4997769	8.447327	Down
CMKLR1	1.302021	5.149049	6.188542	3.2060246	2.5072967	8.444034	Up
CNST	-0.99237	7.565314	-6.18794	3.2141108	2.5127005	8.441571	Down
ARRDC4	0.82481	8.140739	6.187341	3.2220580	2.5179917	8.439158	Up
NUDCD3	-0.54298	8.01688	-6.18669	3.2307237	2.5238404	8.436532	Down
CASK	0.539235	7.065663	6.186203	3.2373369	2.5280819	8.434534	Up
CCDC86	-0.65053	7.031404	-6.18605	3.2393636	2.5287400	8.433922	Down
LCP1	1.528886	6.801652	6.18764	3.2608620	2.5445922	8.428653	Up
GNB1	0.751424	9.522746	6.183072	3.2797279	2.5583794	8.421819	Up
CDKN2AIF	-1.17809	6.752433	-6.18176	3.2976466	2.5714179	8.416493	Down
MAST3	1.418408	5.844823	6.182956	3.3045032	2.5758241	8.415101	Up
MAP3K13	0.809461	7.813593	6.17997	3.3222741	2.5887315	8.409221	Up
SPRED3	-1.05028	7.136519	-6.17972	3.3257938	2.5905290	8.408186	Down
ACTR1A	-0.81438	8.443455	-6.17928	3.3318421	2.5942940	8.40641	Down
DDX5	0.62939	11.49498	6.176987	3.3637009	2.6181459	8.397109	Up
AGAP2	-0.94328	4.721745	-6.17561	3.3830295	2.6322311	8.391509	Down

SELP	1.055055	4.796468	6.17532	3.3870692	2.6344145	8.390343	Up
CACHD1	0.907541	7.10901	6.174016	3.4054631	2.6477568	8.38505	Up
EFNA1	0.787295	6.830821	6.173807	3.4084207	2.6490921	8.384201	Up
THEMIS2	1.259951	5.492663	6.173267	3.4160838	2.6540821	8.382007	Up
LYSMD1	-1.0596	4.703651	-6.1725	3.4270244	2.6616141	8.378882	Down
LRP12	-0.78928	7.074771	-6.17238	3.4287004	2.6619478	8.378404	Down
MAP3K8	0.990326	6.020645	6.171845	3.4363199	2.6668939	8.376234	Up
PTPMT1	-0.56955	7.42926	-6.16991	3.4640691	2.6872956	8.368374	Down
FIS1	-0.52074	10.53817	-6.16983	3.4651242	2.6872956	8.368076	Down
VKORC1	0.78788	8.111685	6.168388	3.4860021	2.7025057	8.362206	Up
GKAP1	-0.71567	9.518482	-6.16646	3.5139528	2.7231859	8.354401	Down
CDK16	-0.9815	9.175646	-6.16597	3.5212150	2.7278240	8.352383	Down
PTGER4	1.055419	8.445581	6.166358	3.5229477	2.7281768	8.352095	Up
CD70	-0.66851	4.371958	-6.16514	3.5333019	2.7352034	8.349034	Down
LRCH1	0.746761	6.485671	6.163369	3.5594033	2.7544107	8.341842	Up
SPATA21	-0.78785	6.158507	-6.16243	3.5733636	2.7642122	8.338016	Down
PITX2	-0.99296	9.587081	-6.16188	3.5890174	2.7753162	8.333937	Down
VPS26B	-0.51507	6.739036	-6.1611	3.5930688	2.7774435	8.332642	Down
DAB2IP	-0.64634	7.989998	-6.16026	3.6055774	2.7861043	8.329246	Down
C1QC	1.925186	7.667527	6.166696	3.6083721	2.7872554	8.330989	Up
MTNAP1	-0.57718	7.929486	-6.15715	3.6525212	2.8203379	8.316604	Down
LSM12	-0.88533	8.554354	-6.15564	3.6754131	2.8369885	8.310499	Down
MYH7B	-1.6024	8.200711	-6.15923	3.6968033	2.8524684	8.306715	Down
TBC1D5	0.520675	7.602779	6.153561	3.7072980	2.8595330	8.302058	Up
C22orf39	-0.52816	8.769199	-6.15262	3.7218318	2.8686714	8.298234	Down
AP4E1	-0.52236	7.218947	-6.1522	3.7282275	2.8725643	8.296557	Down
GAK	0.542367	7.511268	6.151196	3.7438584	2.8825280	8.292468	Up
SNX19	-0.71024	7.190261	-6.15025	3.7586430	2.8928683	8.288617	Down
HEXIM2	-1.08426	6.740581	-6.14889	3.7798694	2.9071102	8.283114	Down
PHACTR2	1.075427	9.087573	6.149399	3.7942908	2.9171513	8.279934	Up
ZBTB7B	-0.85605	5.931922	-6.14723	3.8059583	2.9240164	8.276392	Down
TBL2	0.577614	9.085522	6.146703	3.8143000	2.9293714	8.274253	Up
ZDHHC14	-1.079	4.815284	-6.14627	3.8211052	2.9335430	8.272511	Down
ZNF83	1.527863	6.80907	6.148727	3.8381613	2.9455784	8.269497	Up
PTPRD	1.333612	6.17143	6.144499	3.8690470	2.9682150	8.260795	Up
SIRT5	-1.12165	7.640535	-6.14219	3.9004920	2.9912642	8.252749	Down
MIR1-1HG	-1.27467	10.04635	-6.14438	3.9256521	3.0094787	8.247887	Down
IKZF4	-0.74514	6.411657	-6.1384	3.9480345	3.0255515	8.24058	Down
VGLL2	-1.38392	9.542321	-6.14068	3.9917675	3.0579688	8.231695	Down
STAT1	0.867783	9.521762	6.135201	4.0006693	3.0636893	8.227639	Up
ZNF677	1.007942	6.095074	6.13468	4.0093285	3.0681203	8.225527	Up
NAPA	-0.74691	8.598998	-6.13416	4.0180599	3.0737007	8.223401	Down
NUTF2	-0.89154	9.193707	-6.13347	4.0295524	3.0802856	8.220611	Down
FZD4-DT	-0.86874	4.872528	-6.13248	4.0460421	3.0917842	8.21662	Down
THG1L	0.934419	4.948544	6.131821	4.0571347	3.0979838	8.213945	Up
NUDT21	0.601228	9.007648	6.131728	4.0587021	3.0979838	8.213568	Up
BOD1	-0.52262	9.151223	-6.13165	4.0599572	3.0979838	8.213266	Down
NIPAL2	0.920264	5.860359	6.130467	4.0799734	3.1121454	8.20846	Up
ZNF546	-0.65419	5.587455	-6.1296	4.0947040	3.1222666	8.204939	Down
RFESD	-0.89126	5.637151	-6.12948	4.0966224	3.1226146	8.204482	Down
CAV1	0.808863	11.45858	6.129373	4.0985223	3.1229482	8.204028	Up
ARHGAP2	0.727388	6.448258	6.128701	4.1099457	3.1305356	8.201309	Up
ARGLU1	0.706766	9.908652	6.128364	4.1156945	3.1337969	8.199943	Up
ENTREP1	-0.80679	8.796192	-6.12801	4.1217954	3.1373238	8.198496	Down
CCNB1	1.374999	4.605348	6.12741	4.1320067	3.1439757	8.196078	Up
MAMDC4	-1.09649	3.9428	-6.12602	4.1558169	3.1598411	8.190465	Down
ARNT2	-1.61947	4.502911	-6.12674	4.1847016	3.1806711	8.184607	Down
POMGNT2	-0.64991	7.072864	-6.12214	4.2232489	3.2065464	8.174739	Down

JPH2	-0.95936	9.822705	-6.12115	4.2456925	3.2224414	8.169672	Down
DMPK	-1.33466	8.302904	-6.1231	4.2576008	3.2303317	8.167936	Down
TCTN3	0.535463	6.596186	6.119195	4.2750925	3.2413002	8.162818	Up
NSUN5P2	0.574254	8.305743	6.109837	4.4440703	3.3663963	8.124946	Up
COMMD2	0.631141	7.688191	6.109797	4.4448158	3.3663963	8.124782	Up
DST	-0.51464	11.23316	-6.10769	4.4838104	3.3947261	8.116248	Down
NAT10	-0.51691	6.850177	-6.1065	4.5059700	3.4102943	8.111432	Down
CALHM6	1.56635	5.516259	6.109003	4.5125389	3.4140562	8.111103	Up
NBAT1	-0.94926	4.864062	-6.10456	4.5422267	3.4340841	8.103603	Down
PARGP1	-0.79199	6.405444	-6.10414	4.5501981	3.4388935	8.10189	Down
HBA1	-1.47817	12.59032	-6.11258	4.5638822	3.4480154	8.102464	Down
APP	1.006825	10.12299	6.103365	4.5913043	3.4675062	8.093641	Up
NES	1.526695	10.15103	6.105984	4.6075133	3.4785178	8.091527	Up
NCAPG2	0.664088	5.685609	6.100943	4.6108052	3.4797731	8.088964	Up
OGG1	0.576413	6.896281	6.100778	4.6139403	3.4809091	8.0883	Up
PDE2A	0.833103	7.112029	6.100623	4.6169016	3.4819133	8.087673	Up
ACSS3	-1.05223	5.586915	-6.10047	4.6199244	3.4829631	8.087034	Down
UBE2O	-1.20241	7.499814	-6.10219	4.6232411	3.4842337	8.087058	Down
BHLHE41	-1.24435	9.501374	-6.10071	4.6707598	3.5185838	8.07745	Down
FAM204A	-0.51003	8.044126	-6.09775	4.6721152	3.5185838	8.07606	Down
VRK1	0.602857	6.124558	6.096695	4.6926021	3.5327669	8.071786	Up
GLB1	0.677704	7.016077	6.095367	4.7184556	3.5497279	8.066419	Up
LPIN2	0.897605	6.071029	6.093302	4.7589558	3.5789358	8.05807	Up
ZNF286A	1.258555	6.291573	6.092755	4.7804294	3.5938194	8.05388	Up
KRBA1	-0.5151	5.938732	-6.09199	4.7848885	3.5948107	8.052761	Down
LBX1-AS1	-1.04897	5.371472	-6.09189	4.7867973	3.5948107	8.052372	Down
GNA11	-0.52462	8.906292	-6.0915	4.7944923	3.5993239	8.050803	Down
LOC10192	-0.73307	3.684266	-6.09131	4.7982941	3.6009123	8.050028	Down
TRIM5	0.951019	5.751611	6.085291	4.9193479	3.6904613	8.025691	Up
CRYZL1	0.683191	7.756617	6.085049	4.9242762	3.6928613	8.024713	Up
ATP8B2	1.098862	6.671365	6.084743	4.9305177	3.6962441	8.023475	Up
TTC13	0.553168	7.298922	6.083782	4.9501516	3.7096609	8.019593	Up
TIGD5	-0.98302	5.996713	-6.08312	4.9637068	3.7185145	8.016922	Down
TLN1	-1.10979	7.085802	-6.08276	4.9711276	3.7227680	8.015463	Down
PEBP1	-0.56819	11.32939	-6.08234	4.9798262	3.7279750	8.013755	Down
CCL2	2.247073	7.380075	6.090297	5.0029363	3.7439633	8.012723	Up
DUSP26	-0.9075	10.28703	-6.07995	5.0293378	3.7624026	8.004092	Down
TSC1	-0.52971	8.7792	-6.0796	5.0365061	3.7664460	8.002701	Down
H19	1.303213	12.45172	6.082302	5.0960897	3.8083374	7.993348	Up
CTDSPL2	0.630055	7.116059	6.076495	5.1016269	3.8111419	7.990152	Up
DCLRE1B	0.799243	5.025485	6.075239	5.1281872	3.8296441	7.985081	Up
CENPW	1.06869	5.17304	6.073895	5.1567816	3.8496519	7.979649	Up
DOT1L	-0.89592	4.941768	-6.07348	5.1656087	3.8542701	7.977979	Down
CYRIA	0.708241	6.893533	6.073436	5.1665770	3.8542701	7.977796	Up
MRPL37	-0.75219	9.495486	-6.069	5.2622906	3.9243018	7.959868	Down
RDH13	-0.79399	6.322805	-6.06881	5.2663496	3.9259579	7.959114	Down
ATP10D	1.132573	6.209636	6.067917	5.2858121	3.9390919	7.955512	Up
SSC4D	-1.17299	5.522654	-6.067	5.3058958	3.9526795	7.951808	Down
USP47	-0.62269	10.08024	-6.06619	5.3236462	3.9645201	7.948546	Down
MED22	-0.63827	5.7588	-6.06468	5.3570283	3.9879892	7.942441	Down
IL6ST	0.770902	11.17023	6.062978	5.3948183	4.0147223	7.935575	Up
CAPS	-0.60781	5.802872	-6.0608	5.4436302	4.0482261	7.926778	Down
MON1A	-0.77136	6.140345	-6.05787	5.5099104	4.0960900	7.914959	Down
ADH1B	1.642203	9.726825	6.062561	5.5246802	4.1056409	7.914409	Up
HTT	-0.75085	5.503895	-6.05566	5.5603335	4.1306992	7.906062	Down
ACYP2	-0.64661	10.83218	-6.05521	5.5707174	4.1369743	7.90424	Down
RASGRP4	-0.74591	5.435994	-6.05386	5.6019909	4.1587529	7.898773	Down
GRIK1	-0.69152	4.593072	-6.05124	5.6628089	4.2024417	7.888228	Down

NRIP2	-0.87116	4.600883	-6.05046	5.6812847	4.2146884	7.885047	Down
CDKN1A	2.252803	8.079253	6.058093	5.7121768	4.2346640	7.883216	Up
GNL2	0.552478	7.650112	6.047882	5.7419855	4.2538095	7.874668	Up
SLX1B	-0.63628	6.521848	-6.0468	5.7676479	4.2713392	7.870313	Down
CPA3	2.078667	7.131936	6.051818	5.8148579	4.3048089	7.865063	Up
CD79A	-0.83551	5.742973	-6.04408	5.8327650	4.3165695	7.85935	Down
SERGEF	-0.59907	6.078735	-6.04398	5.8353185	4.3169634	7.858922	Down
USP7	-0.54459	9.209393	-6.04286	5.8622033	4.3341225	7.854434	Down
PALS1	-0.60164	8.638958	-6.04285	5.8625713	4.3341225	7.854372	Down
VAV3	1.222561	5.111917	6.042462	5.8719422	4.3395481	7.852813	Up
MED12	-0.6148	7.076561	-6.04185	5.8867039	4.3489527	7.850361	Down
GNPNAT1	-0.52261	7.321523	-6.03951	5.9440674	4.3898130	7.840891	Down
INTS15	-0.59883	6.545177	-6.03759	5.9913732	4.4216914	7.83315	Down
ANAPC15	-0.73508	7.875167	-6.0359	6.0331189	4.4509621	7.82637	Down
SLC16A1	-1.09574	9.586287	-6.03808	6.0360582	4.4515929	7.826787	Down
S100A11	1.235484	9.221725	6.036531	6.0875746	4.4880365	7.818687	Up
MDH2	-0.52537	12.25014	-6.0335	6.0932941	4.4907031	7.816679	Down
SFRP4	2.131731	8.492772	6.041714	6.1101600	4.5015798	7.817433	Up
VCY	-0.90699	4.478757	-6.0313	6.1488823	4.5285458	7.807811	Down
CNOT6L	0.673899	8.408451	6.031087	6.1542325	4.5309237	7.806962	Up
XG	2.000045	6.000794	6.035449	6.1633122	4.5360448	7.807359	Up
ELP5	-0.71579	7.991994	-6.02948	6.1951502	4.5579062	7.800491	Down
RABGAP1I	1.353511	7.240956	6.032092	6.1999862	4.5598934	7.80082	Up
HRC	-0.64199	10.04307	-6.02631	6.2766823	4.6147119	7.787725	Down
ZNF746	-0.52821	6.266705	-6.02512	6.3076039	4.6358501	7.782926	Down
SHC1	0.645263	9.361581	6.024887	6.3136839	4.6387224	7.781986	Up
MRPL52	-0.62781	8.902964	-6.0247	6.3186518	4.6407760	7.781218	Down
NHSL1	1.26373	5.844881	6.023866	6.3403541	4.6551146	7.77787	Up
ADAMTS5	1.287987	7.920799	6.026047	6.3563519	4.6652565	7.776498	Up
SURF4	0.529464	8.891087	6.022388	6.3791096	4.6803511	7.77192	Up
PLEKHN1	-1.29524	4.386485	-6.02189	6.3922741	4.6883994	7.769907	Down
WNT7B	-0.94622	5.253873	-6.02049	6.4291366	4.7138173	7.764292	Down
ZMAT3	0.916992	7.585714	6.019498	6.4556194	4.7316102	7.760279	Up
XRCC4	2.106095	5.636616	6.023083	6.4851799	4.7516457	7.757649	Up
PTPRA	0.787679	6.312041	6.015571	6.5610152	4.8039137	7.744467	Up
TPBG	0.890586	7.392655	6.013137	6.6271842	4.8506991	7.73467	Up
SLC13A2	-0.86696	4.199179	-6.01282	6.6358012	4.8553423	7.733401	Down
DUSP13B	-0.8398	10.6714	-6.0126	6.6418888	4.8581322	7.732506	Down
MTHFD1L	2.252692	4.463893	6.016856	6.6534983	4.8649578	7.73263	Up
TACC1	0.697639	9.961238	6.01193	6.6602452	4.8682244	7.729811	Up
HS2ST1	0.824123	6.784719	6.010258	6.7063068	4.9002155	7.723082	Up
IGDCC3	-0.77686	5.070481	-6.00958	6.7251824	4.9123271	7.720338	Down
MTMR12	0.571707	8.218832	6.009352	6.7314112	4.9151959	7.719434	Up
FBXO4	0.636466	5.91477	6.008278	6.7612685	4.9353101	7.715114	Up
HBA2	-1.83663	11.84403	-6.01697	6.7640795	4.9356751	7.718142	Down
CLEC16A	-0.60255	5.918071	-6.00547	6.8398644	4.9875665	7.70383	Down
GUCD1	-0.83318	9.28128	-6.00533	6.8439262	4.9888251	7.703251	Down
UQCRC1	-0.68245	11.80726	-6.0047	6.8618558	4.9984828	7.700696	Down
ZNF318	-0.52151	6.165363	-6.00289	6.9129979	5.0323043	7.693447	Down
RSU1	0.61593	8.372128	6.002405	6.9269315	5.0407292	7.691481	Up
NOVA2	-0.90597	4.141846	-5.99573	7.1200388	5.1794887	7.664638	Down
TAX1BP3	0.729555	9.516397	5.995529	7.1259992	5.1820596	7.663821	Up
RAPSN	-0.63394	7.068677	-5.99416	7.1663141	5.2096030	7.658314	Down
SYNJ2	-0.93966	5.672405	-5.99247	7.2164713	5.2442802	7.651505	Down
PLN	-0.79319	12.44313	-5.99268	7.2498313	5.2667311	7.647526	Down
SCAP	-0.55788	7.230023	-5.99097	7.2610661	5.2730993	7.645491	Down
MCF2L	-0.71651	6.837614	-5.98865	7.3306330	5.3218103	7.636183	Down
HAX1	-0.6177	10.35972	-5.98851	7.3348785	5.3230830	7.635618	Down

CGRRF1	-0.71986	7.75093	-5.98733	7.3707519	5.3473003	7.630855	Down
FBLN5	1.101572	8.727189	5.988879	7.3783669	5.3510071	7.630555	Up
TUBA8	-0.83939	5.36476	-5.98644	7.3978314	5.3633022	7.627276	Down
EBF2	1.154814	7.396543	5.986362	7.4401293	5.3917215	7.622225	Up
IDI2	-2.35881	8.324613	-5.99369	7.4420802	5.3917215	7.624871	Down
ANKRD2	-2.04311	10.04343	-5.99232	7.4838550	5.4201484	7.619405	Down
MAN2B1	0.857327	6.687667	5.982788	7.5098679	5.4364423	7.612603	Up
LIMK2	0.957039	6.282032	5.981138	7.5610482	5.4704901	7.605973	Up
ATF7IP2	1.127912	4.987372	5.97821	7.6527348	5.5349512	7.594208	Up
SLC19A2	-1.37864	9.438559	-5.98138	7.6980487	5.5658403	7.590259	Down
IGFBP7	1.084157	11.74385	5.980909	7.7130194	5.5747773	7.588363	Up
AMN	-0.88471	4.227431	-5.97537	7.7426419	5.5926390	7.582807	Down
SEC31B	-0.91072	5.92078	-5.97495	7.7562091	5.6003081	7.581098	Down
GNAQ	0.739506	9.21817	5.972034	7.8497597	5.6659401	7.569396	Up
LINC0048:	-1.43747	4.529491	-5.97269	7.8667338	5.6762737	7.567752	Down
CYP4B1	-3.44506	3.896251	-5.97998	7.8722507	5.6783360	7.570009	Down
CPE	1.027213	9.126568	5.969867	7.9553262	5.7363220	7.556783	Up
PTPN20	-2.12219	4.322207	-5.97124	8.0255431	5.7850000	7.54959	Down
CDC34	-0.69185	8.331962	-5.96604	8.0458938	5.7977125	7.545308	Down
ZNF137P	0.726357	4.463748	5.96547	8.0646152	5.8092429	7.54304	Up
LINC0298	1.799284	4.670373	5.969534	8.0686012	5.8101545	7.54421	Up
LRRN3	2.288579	5.989431	5.971958	8.0717469	5.8104608	7.544823	Up
ACSS2	-0.75857	8.736858	-5.96185	8.1855456	5.8903936	7.528513	Down
FAAP20	-0.57733	6.169921	-5.96152	8.1967991	5.8965050	7.527172	Down
UMAD1	-0.59625	7.247797	-5.96143	8.1997388	5.8966337	7.526822	Down
CLIP3	1.094159	5.221069	5.957297	8.3402867	5.9956864	7.510235	Up
SELENOO	-0.64431	6.76818	-5.95597	8.3859207	6.0264635	7.504909	Down
MTFR1L	-0.83782	9.306248	-5.95359	8.4682609	6.0835895	7.495373	Down
NOL6	-0.59947	6.373272	-5.95338	8.4756650	6.0868612	7.494521	Down
CALCR	1.161531	4.604915	5.95314	8.4840766	6.0908541	7.493552	Up
FPGS	-0.75651	6.511538	-5.95203	8.5229591	6.1167124	7.48909	Down
KLHL21	-0.89307	8.650165	-5.95082	8.5654710	6.1450753	7.484234	Down
IL17C	-0.81623	6.676098	-5.95074	8.5682341	6.1450753	7.483919	Down
PCDHB4	0.961024	5.071571	5.949864	8.5990964	6.1651393	7.48041	Up
EPB41	-1.06875	7.392171	-5.94958	8.6106314	6.1713378	7.479119	Down
PSAT1	1.49438	4.747002	5.951271	8.6158333	6.1729945	7.479258	Up
RAB1A	0.550118	11.14109	5.946013	8.7362723	6.2560449	7.464965	Up
STX2	0.711906	6.860641	5.945976	8.7376074	6.2560449	7.464816	Up
TUBA1C	0.818982	11.68541	5.946158	8.7644210	6.2731402	7.462194	Up
LSP1	1.042301	7.220288	5.943448	8.8288512	6.3171391	7.454678	Up
SURF6	-0.77586	6.622136	-5.94256	8.8612009	6.3381624	7.451109	Down
NR1D1	-1.94573	6.983056	-5.94757	8.8686811	6.3413890	7.452357	Down
ELP6	-0.51295	7.097792	-5.94168	8.8933152	6.3568750	7.447579	Down
UBE2D1	-0.73316	10.97385	-5.94064	8.9311390	6.3817754	7.443437	Down
SCAI	0.836004	6.194289	5.938608	9.0061615	6.4328912	7.435274	Up
ST8SIA4	1.41324	5.684	5.9403	9.0086982	6.4328912	7.435699	Up
PPP1R11	-0.60529	9.02097	-5.93774	9.0383094	6.4497230	7.431797	Down
SAMD10	-0.8721	5.043497	-5.93743	9.0497334	6.4557183	7.430564	Down
TACO1	-0.74794	7.810406	-5.9373	9.0546793	6.4570898	7.430031	Down
LRRC25	-0.90195	4.809372	-5.93688	9.0701860	6.4659891	7.428362	Down
SMCO1	-1.71056	8.854437	-5.94082	9.1174060	6.4974827	7.425363	Down
POU2F2	-0.67483	4.968783	-5.93472	9.1509531	6.5186721	7.419711	Down
EIF2B5	-0.57929	7.700355	-5.93466	9.1532436	6.5186721	7.419466	Down
DUSP12	0.505039	7.063957	5.933362	9.2023093	6.5514308	7.41425	Up
SPAG7	-0.59424	10.31168	-5.93265	9.2292722	6.5684370	7.411395	Down
VPS26C	-0.54231	7.926665	-5.92999	9.3305200	6.6338630	7.400748	Down
RANBP10	-0.55369	6.587202	-5.92965	9.3436235	6.6405265	7.399379	Down
VN1R1	-0.72205	4.912926	-5.92959	9.3461104	6.6405265	7.399119	Down

EPM2A	-0.80115	9.040919	-5.92933	9.3558581	6.6434755	7.398102	Down
CCDC34	0.824096	6.053258	5.929316	9.3564821	6.6434755	7.398037	Up
HPGDS	1.323402	5.597582	5.928743	9.4121751	6.6807988	7.392594	Up
JUP	-1.03404	7.615443	-5.92699	9.4461396	6.7026794	7.388731	Down
PDCD2L	-0.65266	7.51001	-5.9219	9.6459193	6.8421636	7.36831	Down
PLCD4	-0.97326	9.170628	-5.92181	9.6668254	6.8547163	7.366374	Down
MLF2	-0.61912	8.923459	-5.92114	9.6760322	6.8589677	7.365269	Down
GOLM2	0.778391	9.546439	5.920666	9.6946935	6.8699158	7.363389	Up
SNX20	-1.56812	6.439803	-5.92415	9.7098054	6.8783424	7.363409	Down
RASD2	-1.15452	5.149516	-5.91991	9.7248481	6.8867144	7.360358	Down
ZNF791	-0.67311	7.714861	-5.91978	9.7298579	6.8879783	7.359856	Down
MAP4K4	0.566009	8.881305	5.917105	9.8374285	6.9618223	7.349128	Up
MYF5	1.485815	7.223245	5.920959	9.8613723	6.9741453	7.348531	Up
TXNDC16	0.67761	6.684945	5.915074	9.9197285	7.0130937	7.340999	Up
DET1	-0.82829	8.034533	-5.91437	9.9483492	7.0310007	7.338188	Down
RPS6KA2	-0.578	8.073372	-5.91377	9.9729990	7.0460904	7.335774	Down
L3MBTL2	-0.52024	7.064498	-5.91188	1.0050468	7.0984756	7.328224	Down
MFAP2	2.63942	5.910349	5.91866	1.0117862	7.1437128	7.325062	Up
CIAO2B	-0.55955	10.41662	-5.90916	1.0163205	7.1733558	7.317341	Down
YWHAZ	0.694318	9.901961	5.907598	1.0228669	7.2171758	7.311077	Up
RB1	0.783541	8.901704	5.907214	1.0244797	7.2261684	7.30954	Up
TTC23	0.744025	4.846965	5.904666	1.0352426	7.2972648	7.299344	Up
RNF138	0.639816	7.944095	5.904423	1.0362751	7.3021331	7.298371	Up
STK39	1.123754	6.103714	5.904289	1.0368450	7.3037391	7.297835	Up
PHF6	0.667627	7.707448	5.902395	1.0449267	7.3582411	7.29026	Up
HLA-B	0.962386	11.49343	5.904354	1.0478638	7.3764915	7.28858	Up
IDO1	-1.6815	4.881491	-5.90383	1.0500999	7.3897975	7.286501	Down
ID2-AS1	-0.57397	4.742118	-5.90008	1.0549031	7.4211532	7.28099	Down
EMP3	1.06034	8.047086	5.900673	1.0562599	7.4282515	7.2801	Up
INSIG1	0.955319	7.699588	5.89733	1.0668538	7.4953503	7.27	Up
INPP5A	-0.50908	9.832246	-5.89449	1.0793503	7.5806526	7.258639	Down
ANKH	-0.70041	10.19187	-5.89418	1.0807248	7.5878112	7.257398	Down
TMEM160	-0.82867	5.820088	-5.89409	1.0811362	7.5882052	7.257026	Down
LINC0127ɛ	-0.72971	5.723522	-5.89239	1.0886644	7.6360246	7.250257	Down
ARHGEF3	0.674363	7.775248	5.890942	1.0951551	7.6765091	7.244458	Up
RHBDD3	-0.68343	5.979775	-5.8899	1.0998208	7.7066844	7.240311	Down
NDUFAF6	-0.66715	8.535445	-5.88954	1.1014579	7.7156241	7.23886	Down
UQCC1	-0.67123	9.330316	-5.88933	1.1024292	7.7198960	7.238	Down
SP3	0.681011	8.866221	5.888979	1.1039980	7.7283478	7.236613	Up
TPRKB	-0.58955	9.330887	-5.88803	1.1082944	7.7539687	7.232824	Down
KCND2	1.019284	4.748647	5.888012	1.1083840	7.7539687	7.232745	Up
RASL10B	-1.48161	5.930339	-5.88909	1.1154269	7.8006836	7.227622	Down
LYPLA2	-0.6174	6.954278	-5.88613	1.1169790	7.8064263	7.22521	Down
FAM3C	0.971991	9.308454	5.886464	1.1177488	7.8092511	7.224741	Up
TMEM94	-0.55646	6.99203	-5.88586	1.1181918	7.8097910	7.224151	Down
FBXO21	-0.81684	9.013454	-5.88577	1.1185905	7.8100221	7.223803	Down
SBF2	0.573939	7.295892	5.88503	1.1220081	7.8300488	7.220828	Up
ABRAXAS:	-0.67671	8.056375	-5.88499	1.1221921	7.8300488	7.220668	Down
CYB5A	0.738702	8.661968	5.884328	1.1252404	7.8487543	7.218022	Up
ZNF503-A	-0.70408	4.296994	-5.88389	1.1272626	7.8602923	7.21627	Down
DUS1L	-0.61383	8.24855	-5.8834	1.1295014	7.8733330	7.214335	Down
ACAD8	-0.50976	8.135253	-5.88265	1.1330103	7.8952155	7.211309	Down
ABLIM2	-1.07549	8.667667	-5.88362	1.1361104	7.9142360	7.209305	Down
RPL3L	-1.29628	10.37264	-5.88535	1.1409764	7.9455413	7.206249	Down
LINC0238:	0.934299	6.27954	5.879578	1.1473469	7.9872998	7.199044	Up
TGM2	-1.09939	6.818871	-5.87931	1.1486150	7.9923464	7.197966	Down
RCC1L	-0.61412	8.310147	-5.87926	1.1488202	7.9923464	7.197792	Down
LOC38983	1.443806	7.46309	5.882279	1.1551851	8.0340099	7.19416	Up

PLPP7	-0.92907	9.442602	-5.87756	1.1568780	8.0431642	7.190974	Down
B4GALT5	-0.67123	9.20769	-5.87723	1.1584125	8.0512123	7.189681	Down
ECI2	-0.62531	10.44831	-5.87457	1.1711028	8.1341187	7.179054	Down
ELOVL5	1.279402	9.050436	5.878252	1.1718561	8.1367048	7.179973	Up
TRAF5	1.425366	6.340901	5.876476	1.1744935	8.1523670	7.177286	Up
CACNA2D	1.353474	4.5852	5.87352	1.1785191	8.1776518	7.173093	Up
MAGED2	1.093758	9.278312	5.873115	1.1884346	8.2411000	7.165584	Up
USB1	-0.77483	5.518167	-5.87066	1.1900247	8.2494476	7.16342	Down
BHLHE22	1.773822	5.779096	5.871805	1.2058890	8.3539986	7.152272	Up
MCRS1	-0.71687	7.504695	-5.86461	1.2198271	8.4478174	7.139294	Down
ILF3	-0.56309	8.176774	-5.8641	1.2224118	8.4629730	7.13723	Down
FKBP7	0.748879	6.728291	5.863947	1.2231668	8.4642324	7.136628	Up
VRK3	-0.6957	6.889729	-5.8639	1.2233863	8.4642324	7.136453	Down
NDUFA3	-0.61348	11.02579	-5.86347	1.2255412	8.4763959	7.134736	Down
FIZ1	-0.77602	7.110846	-5.86272	1.2292995	8.4996370	7.13175	Down
PCBP1	-0.56778	10.92827	-5.86248	1.2305168	8.5053004	7.130785	Down
SFMBT1	-0.68156	6.262852	-5.86194	1.2332334	8.5213202	7.128634	Down
LAMB3	-0.77296	4.427641	-5.86114	1.2372965	8.5466298	7.125426	Down
FKBP1B	0.897714	4.850858	5.858868	1.2488529	8.6236664	7.116359	Up
HINT2	-0.67695	9.20953	-5.85721	1.2573375	8.6710412	7.109755	Down
VPS4B	0.65314	7.870442	5.855434	1.2665198	8.7287291	7.102659	Up
SSR3	0.554729	9.164953	5.854266	1.2725833	8.7676889	7.098001	Up
THAP1	-0.53282	6.985211	-5.85168	1.2861196	8.8580918	7.087682	Down
GSTK1	-0.50811	9.891735	-5.85011	1.2943730	8.9120631	7.081443	Down
MPP7	-1.46502	6.627244	-5.85218	1.2971078	8.9280144	7.080431	Down
SOCS1	-0.9718	5.427278	-5.84706	1.3106527	9.0183377	7.069254	Down
TRUB1	0.727271	7.862607	5.8452	1.3206428	9.0841514	7.061849	Up
MRPL49	-0.51054	8.345347	-5.84492	1.3221306	9.0914569	7.060751	Down
DBNDD1	-1.18359	5.306864	-5.84452	1.3242953	9.1034116	7.059156	Down
DCP2	0.725177	8.202986	5.843974	1.3272797	9.1209911	7.056961	Up
ARPP19	-0.69406	10.81054	-5.84333	1.3307750	9.1420690	7.054396	Down
SFXN4	-0.7371	8.343757	-5.84182	1.3390314	9.1958305	7.048365	Down
CUL5	-0.55302	10.53604	-5.839	1.3545537	9.2994403	7.037125	Down
PHLDA1	1.974696	6.43591	5.842083	1.3642521	9.3600055	7.032132	Up
TRIM63	-1.20864	10.53875	-5.84122	1.3662360	9.3706068	7.030508	Down
STARD8	-0.72493	6.641058	-5.83667	1.3675052	9.3752569	7.027846	Down
LINC0095	-0.80973	7.336082	-5.83662	1.3677919	9.3752569	7.027641	Down
TRIM54	-0.81629	9.123552	-5.83548	1.3741594	9.4140026	7.023112	Down
CPNE9	-0.96193	4.440595	-5.83539	1.3746442	9.4140026	7.022768	Down
PRODH	-1.05856	5.711229	-5.83206	1.3934970	9.5392021	7.009486	Down
WASHC1	1.250925	7.813166	5.833942	1.3973561	9.5625542	7.007831	Up
SLC35A4	-0.68029	8.729596	-5.83079	1.4007485	9.5826993	7.004425	Down
SMIM10L2	0.763056	4.486619	5.830378	1.4031100	9.5951543	7.002782	Up
LINC0086	0.871615	4.698921	5.830315	1.4034676	9.5951543	7.002534	Up
IFT27	-0.52522	6.597729	-5.82991	1.4057898	9.6079550	7.000922	Down
IQSEC1	-0.57338	7.845582	-5.82931	1.4092320	9.6284000	6.998537	Down
ATOSB	-0.5529	6.599134	-5.82368	1.4420470	9.8431576	6.976093	Down
POLI	0.887501	6.605377	5.82208	1.4514747	9.9043444	6.969739	Up
MAN2B2	0.878124	6.265001	5.821126	1.4571349	9.9397921	6.965944	Up
SERINC2	-1.05542	6.507446	-5.8204	1.4614746	9.9662120	6.963044	Down
MRNIP-DT	0.91507	5.263914	5.819913	1.4643736	9.9827938	6.961112	Up
ZNF431	1.251201	6.504834	5.8208	1.4676844	1.0002171	6.959499	Up
SLC41A2	1.156119	4.714654	5.817564	1.4784834	1.0072551	6.951763	Up
TLR2	1.138905	5.672639	5.817381	1.4795848	1.0076840	6.951037	Up
SNX14	0.583685	7.643613	5.816221	1.4866067	1.0121436	6.94642	Up
ITPKB-IT1	-0.83513	4.671325	-5.81362	1.5024452	1.0226011	6.936088	Down
P2RX4	1.139471	5.166074	5.813465	1.5034284	1.0229443	6.93545	Up
RPL23	0.624657	12.40689	5.812331	1.5103977	1.0270319	6.930941	Up

GAS2L1	0.631903	6.729847	5.808191	1.5361285	1.0438636	6.914472	Up
FUT5	-1.00965	4.686676	-5.80801	1.5372812	1.0443146	6.913741	Down
WDR19	0.959035	6.181196	5.806134	1.5490793	1.0519948	6.906287	Up
RUNX3	1.24309	4.526582	5.805413	1.5536368	1.0547545	6.903423	Up
SIM1	-1.22655	7.469939	-5.80736	1.5564556	1.0562974	6.902634	Down
PDE1A	0.789197	6.171472	5.804899	1.5568987	1.0562974	6.901378	Up
LY75	1.468554	4.149109	5.805552	1.5620391	1.0593911	6.898763	Up
TGFBR1	0.732795	8.548106	5.804027	1.5624506	1.0593911	6.897908	Up
SAMD9L	0.870925	7.329887	5.803561	1.5654189	1.0607302	6.896058	Up
LINC0031;	-1.03452	8.36753	-5.80145	1.5847254	1.0731361	6.884474	Down
C17orf75	-0.61489	6.049402	-5.79994	1.5887404	1.0755093	6.881641	Down
COG1	-0.58211	7.814889	-5.79947	1.5917528	1.0772071	6.879794	Down
ABHD4	-0.53636	7.450144	-5.79874	1.5965313	1.0797872	6.876872	Down
BDH2	0.589028	8.830518	5.798729	1.5965765	1.0797872	6.876845	Up
RASSF4	1.315966	8.741542	5.802133	1.6018010	1.0829777	6.875384	Up
DNAJC4	-0.62259	7.775259	-5.79647	1.6113250	1.0887276	6.867881	Down
ACO2	-0.80472	11.27263	-5.79566	1.6192716	1.0937509	6.863246	Down
SNX27	-1.06311	7.738476	-5.79573	1.6220468	1.0952790	6.861778	Down
RBCK1	0.592172	7.410003	5.793014	1.6342160	1.1031474	6.854129	Up
LOC10192	-0.61101	5.40259	-5.79252	1.6375138	1.1050243	6.852164	Down
MYPN	-0.75539	9.932503	-5.79075	1.6493784	1.1126792	6.845127	Down
UBE2QL1	-1.26647	9.340053	-5.79469	1.6510522	1.1134566	6.845859	Down
NPHP3	1.072228	7.895188	5.791057	1.6548008	1.1156324	6.842385	Up
FZD7	-0.71437	8.970701	-5.7896	1.6570968	1.1168278	6.840576	Down
ADGRD2	-1.79152	4.633255	-5.7929	1.6630980	1.1205188	6.838773	Down
ZNF529	0.549863	7.785661	5.787999	1.6679667	1.1230905	6.834202	Up
UGGT2	0.87241	5.849194	5.785627	1.6841679	1.1336419	6.82478	Up
PTPRF	1.798923	5.988902	5.789216	1.6884890	1.1361924	6.824017	Up
RFX1	-0.60456	6.519398	-5.78375	1.6971072	1.1416320	6.81732	Down
NTMT1	-0.7881	8.332231	-5.78235	1.7068056	1.1477945	6.811765	Down
PAIP2B	-1.33386	8.440104	-5.78623	1.7088793	1.1488273	6.8123	Down
WASH2P	-0.63366	8.024848	-5.77873	1.7321782	1.1637578	6.797382	Down
TKFC	-0.75326	5.593553	-5.77577	1.7531889	1.1775034	6.78563	Down
CEP126	1.232522	6.363553	5.776697	1.7544547	1.1779831	6.785383	Up
CEBPZOS	-0.94297	8.236977	-5.77357	1.7689813	1.1873633	6.77689	Down
C2CD2	0.998577	6.310822	5.772633	1.7757143	1.1915082	6.773187	Up
COA7	-0.5849	7.035816	-5.77178	1.7818650	1.1952598	6.769817	Down
PLXNC1	1.003367	7.028052	5.769993	1.7948997	1.2036254	6.762713	Up
MYO6	0.855891	6.084946	5.769885	1.7956900	1.2037774	6.762284	Up
TNFRSF11	1.111004	4.639291	5.769598	1.7977913	1.2048078	6.761144	Up
RBP1	1.195288	7.310603	5.771436	1.7992141	1.2053832	6.761212	Up
REEP1	0.682274	10.32053	5.768242	1.8077442	1.2107181	6.755763	Up
MBNL1-A	-1.27083	10.62344	-5.772	1.8108465	1.2124156	6.75582	Down
DLAT	-0.7189	9.882366	-5.76727	1.8148761	1.2147328	6.751926	Down
IRAG2	1.226901	4.55397	5.767165	1.8156856	1.2148939	6.751491	Up
KIR3DX1	-0.77318	4.332112	-5.7668	1.8183735	1.2163113	6.750049	Down
GLIPR1	1.648716	7.455346	5.770163	1.8244450	1.2199905	6.748528	Up
DBP	-1.28528	7.395367	-5.76759	1.8309314	1.2239447	6.744367	Down
TOP6BL	1.616408	4.372197	5.766915	1.8359745	1.2265481	6.741686	Up
HACD2	1.232658	7.616707	5.765078	1.8497380	1.2349704	6.734407	Up
CDNF	-0.80585	6.469389	-5.76137	1.8590147	1.2407761	6.728507	Down
CNTLN	1.204092	4.077495	5.760447	1.8660185	1.2450616	6.724842	Up
YES1	0.989657	8.613432	5.760813	1.8676470	1.2457590	6.724232	Up
MASTL	0.93587	4.716036	5.759353	1.8743435	1.2498354	6.720504	Up
RCN1	0.628216	8.531597	5.759162	1.8757953	1.2504131	6.719749	Up
CUTC	-0.53103	11.17822	-5.758	1.8846610	1.2559310	6.715154	Down
CHMP7	-0.63793	7.086273	-5.7578	1.8861976	1.2565629	6.714359	Down
UPF3A	0.69893	9.342483	5.756691	1.8947555	1.2618705	6.709948	Up

SYNGAP1	-1.19309	5.828056	-5.75511	1.9070086	1.2696349	6.703666	Down
H1-2	-1.25292	8.177104	-5.75743	1.9081468	1.2699969	6.704107	Down
SCD5	1.495761	6.859843	5.758394	1.9134725	1.2727482	6.702075	Up
ADORA1	-0.64725	5.118118	-5.75158	1.9345751	1.2859836	6.68968	Down
GLIS2	1.140921	6.676971	5.750443	1.9472863	1.2940305	6.683495	Up
HSPA6	1.41942	6.021083	5.751448	1.9550778	1.2988041	6.680428	Up
PHACTR1	0.883438	4.876755	5.747817	1.9643909	1.3045852	6.674775	Up
MRPS30	-0.53767	8.589534	-5.74703	1.9706897	1.3083615	6.671656	Down
MUC3B	-0.75014	5.188438	-5.74627	1.9767943	1.3120066	6.668642	Down
CCM2	-0.55107	7.799865	-5.74562	1.9819799	1.3148412	6.666089	Down
ZFP3	-0.55498	6.967647	-5.74559	1.9822964	1.3148412	6.665933	Down
ZNF101	0.536634	5.508234	5.745347	1.9842139	1.3157044	6.664991	Up
SPX	1.184914	8.95753	5.746184	1.9973182	1.3239826	6.659597	Up
CRTC2	-0.55551	6.433696	-5.74357	1.9986419	1.3244491	6.657931	Down
NOL3	-0.79582	8.313362	-5.74339	2.0000683	1.3249832	6.657236	Down
CLUH	-0.74116	8.22057	-5.74191	2.0121293	1.3325599	6.651377	Down
ZRANB1	-0.54383	9.358056	-5.74058	2.0230612	1.3393278	6.646098	Down
ARHGAP4	1.278382	5.26138	5.740898	2.0236027	1.3393278	6.645997	Up
TIFA	0.707409	5.935831	5.739527	2.0317211	1.3438680	6.641935	Up
SUCLG1	-0.58603	10.93739	-5.73817	2.0429902	1.3509035	6.636546	Down
SLA	1.326148	5.790222	5.738235	2.0539591	1.3577363	6.631905	Up
MCM3AP	-0.56853	7.879003	-5.73626	2.0588409	1.3605422	6.629015	Down
SLC7A2	-1.14695	9.743132	-5.73531	2.0874316	1.3790090	6.616594	Down
MAPK3	-0.55319	7.003414	-5.73195	2.0952268	1.3837308	6.611945	Down
OSBPL5	-0.59032	5.554331	-5.73173	2.0971559	1.3842385	6.611048	Down
NOC2L	-0.784	6.444569	-5.73171	2.0972918	1.3842385	6.610985	Down
ARHGEF2	0.830161	8.114214	5.731319	2.1006324	1.3860151	6.609434	Up
PCAT6	-0.7264	4.670972	-5.73057	2.1070055	1.3897907	6.606483	Down
MAPDA	-0.67581	6.379761	-5.73017	2.1104892	1.3916588	6.604873	Down
PDGFRB	0.773496	9.011634	5.728478	2.1250145	1.4008043	6.59819	Up
KAT7	-0.58311	8.19616	-5.72751	2.1334166	1.4054753	6.594346	Down
PSPH	0.948619	5.159497	5.727313	2.1350903	1.4061442	6.593582	Up
NTRK2	1.826592	7.823075	5.730888	2.1561965	1.4190044	6.586497	Up
SERTAD4	1.260889	5.087114	5.724844	2.1566103	1.4190044	6.58381	Up
LRRC59	0.655718	7.794718	5.724132	2.1628549	1.4226749	6.580993	Up
PRKG1	-0.76976	9.697738	-5.72288	2.1738616	1.4294746	6.576048	Down
NFATC2	0.993254	5.901531	5.722301	2.1789931	1.4316901	6.573751	Up
CDK3	0.849796	5.05031	5.722273	2.1792418	1.4316901	6.573639	Up
CUL1	-0.53204	9.316676	-5.7196	2.2030253	1.4468700	6.563064	Down
PLIN4	-1.56375	8.125773	-5.72359	2.2038967	1.4469974	6.564386	Down
HGF	0.95821	5.702619	5.719183	2.2067553	1.4484290	6.561416	Up
UBXN8	0.554208	6.046906	5.718282	2.2148376	1.4532873	6.557854	Up
EGFL7	-0.96366	6.229702	-5.7182	2.2156058	1.4533448	6.557516	Down
MAPK8IP2	-0.82567	6.00318	-5.71812	2.2162929	1.4533492	6.557214	Down
CNPY4	0.75838	6.33299	5.715451	2.2404396	1.4678316	6.546657	Up
RAX2	-0.58106	5.74486	-5.71514	2.2432952	1.4692517	6.545416	Down
RASAL3	-0.79126	5.760547	-5.71483	2.2460533	1.4706072	6.544219	Down
TMEM25	-0.67739	4.937646	-5.71407	2.2530357	1.4747268	6.541195	Down
DNAAF5	-0.52102	8.602763	-5.71342	2.2589908	1.4781717	6.538624	Down
CARMIL2	-0.92969	5.745039	-5.7127	2.2656059	1.4820463	6.535775	Down
ABHD11	-0.60105	6.246271	-5.71243	2.2680972	1.4832211	6.534704	Down
ABCA6	0.919458	6.872306	5.712352	2.2687908	1.4832211	6.534406	Up
CD81	0.936307	10.67336	5.713646	2.2724567	1.4851631	6.533541	Up
ABCA2	-0.54149	5.367118	-5.71142	2.2773800	1.4874757	6.530725	Down
CXCL16	1.460114	4.928701	5.713398	2.2773882	1.4874757	6.531549	Up
WRAP73	-0.53097	6.411174	-5.71074	2.2836829	1.4911311	6.528033	Down
B3GALNT2	-0.52895	9.720218	-5.71014	2.2892843	1.4943317	6.525646	Down
PRADC1	-0.77839	8.741092	-5.70997	2.2908058	1.4948680	6.524999	Down

ABHD17A	-0.60908	9.333491	-5.70977	2.2926837	1.4956365	6.524201	Down
EPS8L1	-0.88179	5.459451	-5.70571	2.3307341	1.5198490	6.508166	Down
CTDP1	-0.78157	5.808057	-5.70566	2.3312227	1.5198490	6.507961	Down
POSTN	2.483833	8.654479	5.71002	2.3623643	1.5396819	6.49823	Up
CBLB	0.838831	7.511754	5.702132	2.3648367	1.5408231	6.494015	Up
ACTR3B	-0.7346	6.350677	-5.70096	2.3761139	1.5476987	6.489381	Down
CLASP1	-0.53288	8.38285	-5.69759	2.4088119	1.5678750	6.476068	Down
TPMT	-0.72898	6.869833	-5.69669	2.4175836	1.5727916	6.472527	Down
HOXD8	1.002951	5.780325	5.696079	2.4236014	1.5762265	6.470105	Up
RFC5	-1.10493	9.565019	-5.69791	2.4292034	1.5793889	6.468864	Down
FEZ1	1.006008	7.321612	5.694737	2.4368276	1.5838637	6.464804	Up
GM2A	0.782548	7.865948	5.693709	2.4469984	1.5899906	6.460747	Up
FBXL13	1.984058	5.165906	5.694876	2.4756066	1.6080902	6.451121	Up
ZNF236	0.522789	5.549202	5.689006	2.4940907	1.6196044	6.44218	Up
CDCA4	0.603894	5.309861	5.687947	2.5048229	1.6260792	6.437997	Up
RORC	-1.46649	7.841947	-5.6917	2.5076051	1.6273908	6.43861	Down
ZMAT5	-0.80505	6.338187	-5.68622	2.5223616	1.6364702	6.431201	Down
ZBTB18	-0.50555	10.72434	-5.68611	2.5235013	1.6367124	6.430761	Down
GABARAP	-0.85416	9.470437	-5.68334	2.5520616	1.6547338	6.419799	Down
LINC0017:	1.067667	4.793206	5.682748	2.5581477	1.6581766	6.41748	Up
SMAD1	1.008279	9.133822	5.681226	2.5883714	1.6770285	6.406621	Up
A2M-AS1	-0.77506	6.484431	-5.67981	2.5888019	1.6770285	6.405878	Down
MEX3C	0.51137	8.077929	5.676747	2.6210837	1.6974259	6.393808	Up
DDX60L	1.280121	6.240901	5.677365	2.6310837	1.7028693	6.390757	Up
CES3	-1.15591	5.761069	-5.67492	2.6405201	1.7084589	6.386613	Down
PTX3	2.041281	3.578776	5.677283	2.6581173	1.7193237	6.381832	Up
PIP5K1C	-0.71276	6.616612	-5.67229	2.6688718	1.7257573	6.376211	Down
MTERF3	-0.55764	8.697866	-5.67185	2.6735713	1.7282729	6.374498	Down
ANGPTL1	1.107607	9.686678	5.673009	2.6867460	1.7357386	6.370713	Up
RTN4IP1	-0.86252	7.34483	-5.67011	2.6924559	1.7389015	6.367643	Down
ATP9A	0.83843	8.858043	5.669721	2.6967239	1.7411315	6.366101	Up
CD163L1	1.098298	4.999475	5.669469	2.6994766	1.7423820	6.365107	Up
ABTB1	-0.69599	6.753763	-5.66909	2.7035796	1.7445031	6.363628	Down
SESN1	-0.84422	9.700296	-5.66867	2.7081638	1.7469333	6.361978	Down
ANOS1	-1.107	8.141896	-5.67051	2.7092348	1.7470965	6.362412	Down
GAMT	-1.1717	10.40334	-5.67217	2.7136040	1.7493858	6.36171	Down
MEGF9	-0.51371	8.125215	-5.66727	2.7236093	1.7553062	6.35644	Down
MOB3C	-0.76701	6.492329	-5.66539	2.7443579	1.7681446	6.349049	Down
RECQL	0.92049	8.334983	5.66504	2.7482981	1.7701493	6.347652	Up
DPH1	-0.55631	7.183599	-5.66373	2.7629052	1.7790211	6.34249	Down
KLC2	-0.79669	6.148838	-5.66349	2.7656476	1.7802502	6.341523	Down
KIAA1549	0.717063	4.944067	5.662512	2.7765595	1.7861975	6.337689	Up
IFT20	0.736363	7.616951	5.662416	2.7776333	1.7863502	6.337312	Up
MIEN1	-0.51647	7.994858	-5.66133	2.7898270	1.7936522	6.333047	Down
CC2D2A	0.569876	5.922419	5.660477	2.7995107	1.7993364	6.329672	Up
KIT	1.110753	6.239689	5.658687	2.8198647	1.8113284	6.322618	Up
PITPNA-A	-0.70084	7.549689	-5.65807	2.8268513	1.8152703	6.320208	Down
IRAK3	1.203918	5.471549	5.656419	2.8458505	1.8269214	6.313685	Up
B4GALT4	-0.56256	5.872262	-5.65576	2.8534489	1.8312488	6.311089	Down
PDZRN3	-0.75097	9.682184	-5.65558	2.8555511	1.8320475	6.310372	Down
SCAMP3	-0.6025	8.504887	-5.65544	2.8571986	1.8325540	6.30981	Down
MAP6D1	-1.03582	7.364986	-5.65351	2.8840882	1.8492452	6.300855	Down
MAPKAP1	-0.68042	8.4687	-5.65285	2.8872811	1.8507368	6.299611	Down
FAAH	-0.91376	4.712	-5.65084	2.9107850	1.8646834	6.291717	Down
TOB1-AS1	-0.73263	4.817473	-5.65056	2.9140519	1.8662165	6.290624	Down
RAPGEF6	0.643394	7.680052	5.649735	2.9238193	1.8719104	6.287366	Up
PNMA3	-0.85327	4.834656	-5.64903	2.9322250	1.8767294	6.284571	Down
CD2	1.365916	4.565917	5.649219	2.9422983	1.8826125	6.281674	Up

TMEM201	-1.15188	7.787068	-5.64769	2.9710457	1.9004371	6.272578	Down
MLEC	-0.60447	8.507334	-5.64534	2.9762152	1.9031738	6.270072	Down
ATP1B1	-0.6311	12.59106	-5.64418	2.9902476	1.9104310	6.265492	Down
SP140L	0.87775	5.494815	5.643862	2.9940752	1.9123043	6.264246	Up
FGF18	1.659373	6.269758	5.647458	2.9982920	1.9144251	6.264552	Up
SH3D19	-0.69804	9.295165	-5.64307	3.0036708	1.9172863	6.261131	Down
ANKS1A	0.523605	8.166866	5.642982	3.0047388	1.9173950	6.260785	Up
HGSNAT	0.518657	8.067092	5.641154	3.0270150	1.9310330	6.253593	Up
NAP1L3	1.598479	5.353133	5.643521	3.0284037	1.9313421	6.254201	Up
ZNF416	-0.69356	5.095259	-5.63752	3.0717748	1.9578325	6.239301	Down
E4F1	-0.62719	6.237137	-5.63693	3.0791333	1.9619370	6.236972	Down
TWF1	0.91132	7.320062	5.636234	3.0877923	1.9668675	6.234238	Up
NOTUM	-0.56547	4.617324	-5.63516	3.1012392	1.9742553	6.230007	Down
COPS7B	-0.70796	5.965	-5.63423	3.1128462	1.9810539	6.22637	Down
TTC3	0.672349	10.46695	5.633158	3.1264028	1.9890888	6.222139	Up
SLC25A24	0.836627	8.485804	5.632862	3.1301497	1.9908796	6.220973	Up
IFIT2	1.307707	6.554755	5.633044	3.1569807	2.0073472	6.213642	Up
SENP3	-0.55783	6.868537	-5.62981	3.1689553	2.0143615	6.208977	Down
POLR2F	-0.52668	8.26591	-5.62909	3.1782028	2.0196386	6.206141	Down
CSNK1G3	0.608753	7.29691	5.629015	3.1791603	2.0196462	6.205847	Up
WWOX	1.00807	6.075715	5.627131	3.2034333	2.0338564	6.198442	Up
MPRIP-AS	-0.78305	5.440044	-5.62562	3.2230325	2.0452082	6.192504	Down
EMC9	-0.95199	7.15856	-5.62561	3.2232282	2.0452082	6.192445	Down
FUCA2	0.571387	6.998994	5.623413	3.2518770	2.0621612	6.183831	Up
GATAD2B	-0.70741	6.973165	-5.62097	3.2840514	2.0819462	6.174246	Down
ZFP37	0.837416	4.293088	5.620316	3.2927906	2.0868671	6.171659	Up
PRKAR1B-	-0.5753	4.742228	-5.61977	3.3000059	2.0908195	6.169528	Down
COPG1	-0.80952	7.51804	-5.61861	3.3155172	2.1000242	6.164963	Down
ADCY2	-0.85924	9.554219	-5.6178	3.3263571	2.1062654	6.161786	Down
ELAVL3	-1.31272	4.330875	-5.61801	3.3294244	2.1075828	6.161074	Down
CD38	-1.92281	5.843294	-5.62071	3.3396288	2.1126088	6.159574	Down
FCGR2C	1.336977	6.072584	5.618662	3.3402221	2.1126088	6.158545	Up
CX3CL1	-0.88051	5.639302	-5.61676	3.3403315	2.1126088	6.157705	Down
HSP90B1	0.672306	10.29429	5.616377	3.3455533	2.1152850	6.156184	Up
WDR11	0.95564	7.671474	5.616201	3.3479242	2.1161576	6.155495	Up
ATF3	-1.80165	6.958591	-5.61994	3.3561473	2.1205201	6.154967	Down
TP73	-0.62207	4.285828	-5.61554	3.3568118	2.1205201	6.152914	Down
SCN4A	-0.82596	8.714731	-5.61529	3.3602533	2.1220665	6.151916	Down
UQCC6	-0.58314	8.751304	-5.6147	3.3682002	2.1264564	6.149617	Down
ZC3H3	-0.65735	6.236999	-5.61346	3.3851307	2.1365136	6.144736	Down
OBSCN	-0.64467	10.40295	-5.61305	3.3906959	2.1393939	6.143137	Down
ARL14EP	-0.53749	7.508322	-5.61182	3.4075998	2.1487900	6.138296	Down
NEGR1	1.297788	7.57894	5.613762	3.4188808	2.1552673	6.13626	Up
HCK	1.13387	5.93246	5.610821	3.4213872	2.1562109	6.134366	Up
ANAPC2	-0.59638	6.010745	-5.60962	3.4379977	2.1654012	6.129652	Down
MIR503HC	1.327081	4.619395	5.607887	3.4734576	2.1870904	6.120011	Up
CYSLTR1	1.694055	5.750059	5.610538	3.4792482	2.1893456	6.119702	Up
ZFYVE1	-0.52304	7.967776	-5.6066	3.4801145	2.1893456	6.1178	Down
SH3PXD2A	0.640457	8.823469	5.606442	3.4823519	2.1901081	6.117174	Up
PTTG1IP	0.530877	10.18925	5.606123	3.4868367	2.1922831	6.115921	Up
SNHG15	0.601042	5.685957	5.605549	3.4949139	2.1967149	6.113669	Up
CCDC92	-0.51619	9.228152	-5.60489	3.5042647	2.2019442	6.111068	Down
TOX4	-0.57587	8.296981	-5.60477	3.5059713	2.2023687	6.110594	Down
SERPINA3	2.892213	6.353175	5.609914	3.5361440	2.2206693	6.105371	Up
DNMBP	-0.54933	7.531323	-5.60222	3.5421105	2.2237624	6.100613	Down
CD320	-0.75855	6.130761	-5.60105	3.5589465	2.2336756	6.095997	Down
NTAN1	0.57828	8.960996	5.600211	3.5709442	2.2405472	6.092722	Up
FARP2	0.587948	6.097075	5.598182	3.6002677	2.2576194	6.084762	Up

HSPB1	-0.52186	12.70225	-5.59798	3.6032285	2.2588128	6.083962	Down
MRPS17	-0.57976	8.37487	-5.59708	3.6162518	2.2663118	6.08045	Down
CLPTM1	-0.72232	7.350723	-5.597	3.6174713	2.2664110	6.080122	Down
NDUFA4L2	-0.669	6.34113	-5.59609	3.6308207	2.2741076	6.076537	Down
ABHD8	-0.83779	4.435856	-5.59405	3.6607440	2.2918674	6.068549	Down
SLC2A12	-1.06639	5.344078	-5.59401	3.6613221	2.2918674	6.068395	Down
ZSWIM9	-1.30265	4.947793	-5.59464	3.6636419	2.2926476	6.068117	Down
NOCT	-0.69444	4.541919	-5.59115	3.7037037	2.3163603	6.057194	Down
UQCC5	-0.98908	6.915373	-5.58976	3.7245756	2.3287320	6.051725	Down
ELMOD1	-1.52096	4.488294	-5.59168	3.7299172	2.3313893	6.051311	Down
BCL3	1.197512	7.282431	5.591176	3.7332242	2.3327736	6.050325	Up
ATAD2B	0.80659	5.884576	5.587022	3.7658605	2.3518482	6.040996	Up
DNAJB11	0.561607	7.769285	5.587016	3.7659524	2.3518482	6.040973	Up
PKP4	0.530722	7.347449	5.582588	3.8337086	2.3934623	6.023619	Up
LMNTD2	-0.77364	3.983625	-5.58207	3.8416472	2.3977177	6.021606	Down
KLF7	0.679902	6.938431	5.580221	3.8704173	2.4148438	6.014345	Up
DOCK2	1.513617	4.972088	5.582432	3.8713480	2.4148438	6.01509	Up
IQCH-AS1	-0.86282	6.41372	-5.57984	3.8762963	2.4172244	6.012868	Down
DHX37	-0.74598	5.142153	-5.57959	3.8803066	2.4190189	6.011862	Down
DKK3	1.212168	8.191225	5.577384	3.9507388	2.4614901	5.995335	Up
RUFY3	1.003586	8.806121	5.574789	3.9764440	2.4767830	5.988599	Up
DEPDC5	-0.81201	5.008793	-5.57255	3.9917319	2.4855805	5.984312	Down
MRPL23	-0.64022	8.988618	-5.57163	4.0065448	2.4940770	5.980707	Down
MRTO4	-0.58886	6.874928	-5.57079	4.0202336	2.5018691	5.977388	Down
STAT6	0.723247	7.765661	5.56887	4.0513410	2.5204934	5.969888	Up
LINC02591	-1.34056	5.147253	-5.56777	4.0903283	2.5432673	5.961122	Down
PCDH19	1.299253	5.662904	5.567474	4.0951354	2.5455152	5.959979	Up
H1-0	-0.87927	9.399716	-5.56612	4.0964566	2.5455956	5.959112	Down
DNAJC7	-0.98457	7.893624	-5.56637	4.0991928	2.5465550	5.958646	Down
ACOT7	1.236356	5.205083	5.562784	4.1564170	2.5806033	5.945094	Up
PRRG2	-0.82354	4.789259	-5.56197	4.1653362	2.5853894	5.942888	Down
LIMCH1	0.668778	10.88979	5.561829	4.1677208	2.5861179	5.942331	Up
PCDHB5	0.848233	4.523306	5.56164	4.1708912	2.5873335	5.941591	Up
NDUFB10	-0.68176	12.12116	-5.56137	4.1754257	2.5893943	5.940534	Down
C5orf24	0.543358	9.62441	5.561093	4.1800640	2.5907663	5.939453	Up
MIF4GD	-0.52013	8.280123	-5.5602	4.1951217	2.5993446	5.935955	Down
ARPC4	-0.63146	7.377267	-5.55759	4.2394110	2.6252636	5.925736	Down
CDK15	0.77871	5.299639	5.557268	4.2448629	2.6278777	5.924486	Up
DIPK2A	0.683829	7.82946	5.556235	4.2625219	2.6380453	5.920447	Up
SIRT3	-0.61415	7.237145	-5.55488	4.2858091	2.6509213	5.915146	Down
ZHX1	0.739806	7.563535	5.55443	4.2935629	2.6549483	5.913387	Up
CCL18	2.602307	5.299736	5.561037	4.3021347	2.6591468	5.914525	Up
METAP1D	-0.52198	6.164243	-5.55389	4.3028428	2.6591468	5.911287	Down
FKSG49	-1.15478	8.348317	-5.55535	4.3164667	2.6667948	5.909183	Down
SHD	2.376116	5.154468	5.559426	4.3186726	2.6673860	5.910509	Up
METTL21A	-0.65336	7.132104	-5.55136	4.3469043	2.6840469	5.901375	Down
CPSF1	-0.71404	8.368441	-5.54967	4.3765646	2.7015799	5.894759	Down
CCDC136	-0.81801	4.557087	-5.54858	4.3957238	2.7126226	5.890509	Down
DEAF1	-0.6153	6.218378	-5.54785	4.4086729	2.7191048	5.887647	Down
MEG9	1.376338	4.140727	5.548888	4.4087742	2.7191048	5.88808	Up
TRDC	1.317233	4.279009	5.546776	4.4394762	2.7364598	5.881161	Up
JAG1	0.705009	9.102125	5.545377	4.4526396	2.7437818	5.877993	Up
KALRN	-0.72326	8.401107	-5.54412	4.4752275	2.7569053	5.87307	Down
PRRX1	0.750742	10.80291	5.542435	4.5055738	2.7747994	5.866496	Up
SERPINB9P	2.190815	4.782253	5.546437	4.5179251	2.7816040	5.86587	Up
ITPK1	-0.65562	7.749341	-5.54127	4.5266561	2.7861764	5.861955	Down
ITGB7	-1.30664	6.560367	-5.54334	4.5295114	2.7871306	5.862311	Down
FOLR1	-1.10753	10.10349	-5.54311	4.5417178	2.7938367	5.859883	Down

CAMK2A	-1.18223	7.870903	-5.54077	4.5765563	2.8136468	5.852258	Down
MRPS2	-0.69906	7.803654	-5.53769	4.5923151	2.8225228	5.847945	Down
SULF1	1.096296	7.560915	5.538232	4.6105812	2.8328005	5.844752	Up
VEGFD	-1.33398	5.736978	-5.5383	4.6116899	2.8328005	5.844572	Down
KAT14	-0.63704	7.549547	-5.53506	4.6410276	2.8500019	5.837681	Down
MARS2	-0.90942	5.027979	-5.53498	4.6424096	2.8500312	5.837391	Down
PRKD3	0.752175	7.866681	5.534212	4.6568284	2.8568500	5.834375	Up
SLC22A4	1.010559	6.222502	5.534174	4.6575296	2.8568500	5.834228	Up
GEM	1.040088	6.596088	5.533946	4.6617910	2.8586429	5.833339	Up
JMJD8	-0.63633	6.472183	-5.53265	4.6861750	2.8721671	5.828264	Down
C19orf12	-0.51627	9.235786	-5.53263	4.6865355	2.8721671	5.828189	Down
CLCF1	-0.67329	4.230396	-5.53145	4.7088136	2.8849925	5.823576	Down
PLA2G4F	-0.84493	4.249585	-5.53048	4.7271140	2.8953742	5.819803	Down
TXNDC11	-0.63791	6.720563	-5.52984	4.7392367	2.9019672	5.817311	Down
C21orf91	1.017083	7.633851	5.528144	4.7862537	2.9290775	5.808042	Up
NDUFV2	-0.54667	11.57222	-5.52608	4.8112231	2.9435148	5.802648	Down
FUBP3	0.88988	7.462041	5.525512	4.8222997	2.9494466	5.800411	Up
FAM117B	0.596658	5.902266	5.523928	4.8530438	2.9674008	5.794229	Up
GBP3	1.522692	7.059418	5.527496	4.8560315	2.9683778	5.795256	Up
TECPR2	-0.67653	6.627166	-5.52319	4.8674516	2.9745073	5.791346	Down
PYGO2-A!	-0.72653	4.174448	-5.52308	4.8696490	2.9749989	5.790907	Down
FBXL6	-0.64253	4.82416	-5.5223	4.8847722	2.9825318	5.787891	Down
CELF3	-1.07462	3.942841	-5.5219	4.8927758	2.9857115	5.786298	Down
COL4A5	-1.35055	5.406658	-5.52269	4.9073896	2.9937739	5.784072	Down
RAB22A	0.512385	7.34731	5.52023	4.9255573	3.0039992	5.779803	Up
MRPL14	-0.64314	10.2025	-5.51852	4.9593957	3.0229102	5.773144	Down
KIAA1217	-0.56046	8.568131	-5.5167	4.9958615	3.0442684	5.766019	Down
PHIP	0.755851	8.558003	5.515439	5.0211251	3.0587903	5.761113	Up
ADSS1	-0.90204	12.10394	-5.51732	5.0275774	3.0618477	5.760827	Down
UBL3	-0.61263	9.773911	-5.51347	5.0609878	3.0813163	5.753422	Down
GATD3	-0.56886	10.63914	-5.51285	5.0735083	3.0880590	5.751019	Down
FNDC1	2.01587	7.497936	5.518938	5.0912287	3.0979619	5.750673	Up
CD63-AS1	-0.874	4.897753	-5.51096	5.1120015	3.1097159	5.743668	Down
OAS2	0.990222	5.771963	5.510742	5.1165641	3.1107195	5.7428	Up
SPICE1	0.769169	4.37549	5.509802	5.1358794	3.1206860	5.739136	Up
ZNF562	0.648431	6.81628	5.50903	5.1517863	3.1294611	5.736128	Up
SLC14A1	-1.27257	5.72179	-5.50958	5.1645938	3.1358825	5.734227	Down
CARINH	0.51804	7.683497	5.508377	5.1652939	3.1358825	5.733581	Up
FLOT2	-0.62391	6.87484	-5.50813	5.1704885	3.1381441	5.732604	Down
CARS2	-0.66883	7.869162	-5.50754	5.1826913	3.1446568	5.730311	Down
TBX15	-0.53206	9.730661	-5.50561	5.2228422	3.1681188	5.722806	Down
LOC10272	-1.07318	8.300228	-5.50707	5.2298329	3.1714586	5.722291	Down
CYP2W1	-0.80698	4.554893	-5.50457	5.2446762	3.1791787	5.718749	Down
CFLAR	-0.58905	9.559695	-5.50453	5.2455407	3.1791787	5.718589	Down
ATP8B1-A	0.86544	5.236477	5.50212	5.2964395	3.2091164	5.709198	Up
COA1	-0.66321	8.538386	-5.50176	5.3040331	3.2121701	5.707805	Down
CD48	1.28899	5.656613	5.502167	5.3223175	3.2220538	5.705013	Up
ANKRD46	-0.65431	9.223184	-5.49998	5.3419396	3.2321268	5.700879	Down
TRDV2	1.180463	4.563865	5.499982	5.3419832	3.2321268	5.700871	Up
MED16	-0.65944	6.971317	-5.49983	5.3453157	3.2332272	5.700265	Down
PRMT6	0.849691	5.157877	5.497979	5.3850269	3.2563252	5.693067	Up
BCAS4	-0.76931	4.5093	-5.49764	5.3922884	3.2597932	5.691757	Down
CCL8	2.359886	4.912401	5.503493	5.4014083	3.2643825	5.692868	Up
DNAJC22	-0.99102	6.070408	-5.49462	5.4580434	3.2967862	5.67997	Down
H2BC21	-0.62709	9.065293	-5.49461	5.4581122	3.2967862	5.679958	Down
NR2F6	-0.67413	8.590784	-5.49453	5.4599581	3.2969636	5.679629	Down
RSAD1	-0.62091	7.906126	-5.49446	5.4614933	3.2969636	5.679356	Down
COQ9	-0.66761	10.4782	-5.49386	5.4745091	3.3038871	5.677041	Down

P4HA1	-0.65084	9.412825	-5.49336	5.4856578	3.3096802	5.675063	Down
FANCM	0.786347	5.584487	5.49292	5.4952329	3.3135851	5.673367	Up
GRTP1	-0.79191	5.38014	-5.49212	5.5128836	3.3232901	5.670249	Down
UPK2	-0.6369	4.389758	-5.49173	5.5214185	3.3274959	5.668744	Down
TPP2	-0.61436	8.132165	-5.49116	5.5340929	3.3341934	5.666515	Down
GALT	-0.65396	6.918485	-5.49093	5.5392114	3.3363361	5.665616	Down
ALDH1L1-	-1.58924	3.643996	-5.49188	5.5579750	3.3466938	5.663117	Down
RNF167	-0.60839	8.058942	-5.48954	5.5700677	3.3530300	5.660214	Down
RALGPS1	-0.54612	5.842419	-5.48879	5.5868988	3.3622143	5.65728	Down
SEPTIN6	0.94573	6.67993	5.487956	5.6055290	3.3724757	5.654043	Up
CTAGE9	-0.71633	6.363443	-5.48718	5.6230592	3.3820697	5.651007	Down
ARRB1	0.5844	6.626401	5.48561	5.6584040	3.4023703	5.644914	Up
ZC2HC1A	0.960915	6.149185	5.484691	5.6792370	3.4139359	5.641341	Up
MAP4K5	0.659468	7.138341	5.484001	5.6949543	3.4224208	5.638654	Up
HOTAIRM	0.994517	6.539781	5.482658	5.7256275	3.4398861	5.633431	Up
ASIC4	-0.87651	4.744928	-5.48221	5.7360117	3.4451104	5.631669	Down
PLCL1	-1.12381	7.234909	-5.48365	5.7375492	3.4451104	5.632076	Down
CFHR2	1.24762	6.331912	5.483405	5.7455206	3.4489271	5.630771	Up
RYR1	-0.81734	11.45379	-5.48309	5.7483006	3.4496264	5.630214	Down
ANGPTL2	0.827191	9.690087	5.48114	5.7605109	3.4559829	5.627525	Up
CD9	0.86945	9.814739	5.480764	5.7774955	3.4651994	5.624821	Up
P3H4	1.337256	5.224148	5.481509	5.7846963	3.4685442	5.624077	Up
C10orf71-	-1.14921	8.587395	-5.48217	5.7866697	3.4687536	5.624074	Down
SPATA4	-0.77751	4.436341	-5.47747	5.8456128	3.5031032	5.613266	Down
SYBU	0.71395	5.737482	5.477263	5.8505485	3.5050775	5.612446	Up
AP2B1	-0.67843	8.461473	-5.47522	5.8985696	3.5318657	5.604498	Down
ZNF574	-0.76835	6.200778	-5.47468	5.9112683	3.5384772	5.602407	Down
ABHD14A	-0.68731	7.769465	-5.47322	5.9458749	3.5581954	5.596732	Down
CD74	1.277822	11.37274	5.476553	5.9631187	3.5675151	5.595709	Up
PBX1	-0.54014	9.365226	-5.47092	6.0008154	3.5890623	5.58779	Down
PYGM	-0.80065	13.01683	-5.47288	6.0053644	3.5907775	5.588005	Down
PFDN6	-0.90258	6.806752	-5.46844	6.0605536	3.6209082	5.578159	Down
GGTLC1	-0.66402	4.521801	-5.46843	6.0608422	3.6209082	5.578113	Down
EMILIN3	0.780871	5.407629	5.466751	6.1017030	3.6443001	5.571581	Up
LINC-PIN	-1.09475	7.135784	-5.46666	6.1335098	3.6614555	5.567063	Down
SLC35D2	0.84269	6.623774	5.465436	6.1338551	3.6614555	5.566472	Up
CENPV	-0.9966	8.112158	-5.4662	6.1384108	3.6631511	5.566172	Down
OGFOD2	-0.61589	6.043868	-5.46369	6.1767418	3.6849958	5.559698	Down
MUC8	-1.09536	5.208005	-5.46326	6.1874346	3.6903442	5.558017	Down
PTEN	0.725016	9.457982	5.462333	6.2103859	3.7029989	5.554417	Up
ART1	-1.00639	4.191327	-5.46204	6.2176606	3.7063018	5.553279	Down
COX6A1	0.587826	9.877935	5.460719	6.2505663	3.7238380	5.548148	Up
FAM13B	0.760716	7.610659	5.459848	6.2723434	3.7347285	5.544767	Up
TIMM50	-0.80412	8.508266	-5.45948	6.2815303	3.7391561	5.543344	Down
STX17-DT	0.884432	7.54971	5.456659	6.3527618	3.7805039	5.532383	Up
TUG1	0.50978	10.16107	5.455782	6.3750569	3.7927149	5.528977	Up
DHRS11	-0.77463	6.065125	-5.45546	6.3832987	3.7965606	5.527722	Down
SURF1	-0.71057	9.438287	-5.45477	6.4009212	3.8059820	5.525042	Down
TMEM242	-0.68469	6.788353	-5.45435	6.4115261	3.8112266	5.523432	Down
NEURL2	-0.73702	7.280774	-5.45417	6.4163244	3.8130177	5.522705	Down
PARP8	0.84956	5.465776	5.453383	6.4364313	3.8239026	5.519664	Up
DDX17	0.51647	11.54588	5.450771	6.5039195	3.8629230	5.509525	Up
PIN1P1	-0.63778	4.299588	-5.45067	6.5065999	3.8634407	5.509124	Down
ZNF518A	0.820591	7.166138	5.450313	6.5158169	3.8678381	5.507748	Up
SERAC1	-0.50363	6.116682	-5.45022	6.5182628	3.8682150	5.507383	Down
GSN	0.821551	11.7077	5.451121	6.5401174	3.8801063	5.504904	Up
XKR6	1.359207	4.444897	5.4478	6.6160417	3.9240605	5.493494	Up
CHAF1A	-0.5846	6.292328	-5.44633	6.6202460	3.9254640	5.492293	Down

ARHGEF4	-0.73701	5.53248	-5.44528	6.6480729	3.9408698	5.488216	Down
ZNF277	0.714654	7.082393	5.444549	6.6674589	3.9512649	5.485386	Up
GOLGA2P	-0.99058	5.657636	-5.44212	6.7323565	3.9886178	5.475971	Down
MKKS	-0.53566	8.556443	-5.44115	6.7584050	4.0029399	5.472218	Down
PLCB3	-0.61001	5.004097	-5.44103	6.7618227	4.0038538	5.471727	Down
PALS2	0.730792	5.395821	5.440941	6.7641625	4.0041292	5.47139	Up
LETMD1	0.571086	7.485866	5.436878	6.8746983	4.0673076	5.455636	Up
LMOD1	-1.51641	7.803813	-5.44006	6.8852380	4.0724152	5.455735	Down
GPR160	0.819146	4.93968	5.435018	6.9258813	4.0941869	5.448427	Up
ACOT11	-1.12513	5.633312	-5.43072	7.0455466	4.1626220	5.431778	Down
PCDHB16	1.303048	5.11246	5.431601	7.0525217	4.1655906	5.43132	Up
LOC10272	1.607911	6.701167	5.433885	7.0565186	4.1667991	5.431852	Up
VPS39	-0.58623	7.051313	-5.42874	7.1013879	4.1909765	5.424106	Down
MEOX2	0.935341	8.360172	5.428896	7.1082235	4.1938518	5.423348	Up
NDOR1	-0.93552	4.488734	-5.42723	7.1444743	4.2129122	5.418228	Down
KCND3	-1.06726	5.469166	-5.42646	7.1662446	4.2245833	5.415271	Down
MID2	-0.69135	6.514193	-5.42619	7.1739501	4.2279588	5.414227	Down
SERPINB9	1.255167	6.053662	5.427522	7.1780064	4.2291824	5.414335	Up
PRSS22	-0.83568	4.98596	-5.42532	7.1989011	4.2403234	5.410853	Down
CCL5	1.041481	6.102792	5.424633	7.2186813	4.2508021	5.408186	Up
FSCN1	1.12233	7.60863	5.426066	7.2269557	4.2545016	5.407842	Up
PRKCE	-1.41312	5.133795	-5.42601	7.2319726	4.2562819	5.40722	Down
HSPA13	0.756599	7.255007	5.422602	7.2773564	4.2818121	5.400319	Up
GAS2L3	1.589981	5.381116	5.42422	7.3251595	4.3087512	5.395415	Up
GK5	0.701991	5.988082	5.420276	7.3451228	4.3193043	5.391312	Up
ACTR2	0.635453	10.22526	5.417545	7.4254604	4.3653450	5.380741	Up
IDNK	0.931472	5.660877	5.416081	7.4689083	4.3896792	5.375072	Up
SUMF1	0.929185	7.232374	5.415471	7.4870600	4.3991369	5.372713	Up
USP15	-0.78014	9.239942	-5.41532	7.4915096	4.4005407	5.372136	Down
VRK2	-0.52768	7.363705	-5.41459	7.5134242	4.4122000	5.369298	Down
ENDOV	-1.03131	5.870448	-5.41419	7.5254113	4.4180246	5.367749	Down
SIRPA	1.155225	6.928947	5.415656	7.5281973	4.4184456	5.368088	Up
CORO1C	0.722365	8.307291	5.411474	7.6072024	4.4599129	5.357245	Up
STOX2	0.844263	6.447642	5.409994	7.6521803	4.4850512	5.351516	Up
RABGAP1	-0.53376	8.97685	-5.40959	7.6645999	4.4910980	5.349941	Down
ZSWIM7	-0.92585	8.256014	-5.40895	7.6920901	4.5059698	5.346578	Down
MYL4	1.471798	10.33663	5.408957	7.8422589	4.5926779	5.329978	Up
OR7E104F	-0.6887	6.232969	-5.40316	7.8630718	4.6036044	5.325101	Down
EPSTI1	1.214256	6.082642	5.404219	7.8669273	4.6045994	5.325154	Up
MBD2	0.560873	9.025052	5.401196	7.9249077	4.6372652	5.31749	Up
XPO7	-0.50475	8.023612	-5.40099	7.9312483	4.6397042	5.316713	Down
ROGDI	-0.80256	8.026132	-5.40012	7.9589710	4.6546468	5.313323	Down
SLC7A1	1.08785	7.121416	5.398218	8.0569368	4.7080726	5.301966	Up
HHATL	-0.60558	10.62676	-5.39643	8.0764762	4.7181996	5.299084	Down
MRPS25	-0.69967	8.027858	-5.3949	8.1258149	4.7457248	5.293167	Down
CMSS1	0.542286	7.69814	5.392278	8.2110931	4.7942188	5.283025	Up
BRICD5	-0.73416	4.948074	-5.39136	8.2411724	4.8104663	5.279472	Down
PDSS1	-0.62895	6.743656	-5.3909	8.2561667	4.8174147	5.277706	Down
PPP1R16A	-1.18445	8.216624	-5.39293	8.2575875	4.8174147	5.278471	Down
CC2D1B	-0.60987	5.812268	-5.39021	8.2787918	4.8284663	5.275048	Down
NPTXR	-0.67704	4.615642	-5.38892	8.3214163	4.8520013	5.270059	Down
XPR1	-0.66795	6.999433	-5.3885	8.3355006	4.8588870	5.268416	Down
DDX54	-0.9861	6.520283	-5.38837	8.3396350	4.8599706	5.267934	Down
TRPS1	0.695427	7.592631	5.388082	8.3492589	4.8624193	5.266814	Up
PTMS	-1.07556	5.68657	-5.38797	8.3530843	4.8624193	5.266369	Down
TRIP6	0.793158	7.279206	5.387902	8.3552200	4.8624193	5.26612	Up
PLS3	0.778585	10.11053	5.387249	8.3769504	4.8725019	5.263597	Up
DNAJC28	-0.67869	5.036216	-5.38724	8.3771078	4.8725019	5.263579	Down

MTFP1	-0.89804	6.586404	-5.38622	8.4112723	4.8910415	5.259625	Down
ZNF704	0.962813	8.070451	5.385775	8.4499709	4.9108701	5.255486	Up
REM1	-0.75884	6.71395	-5.3843	8.4758187	4.9245521	5.252199	Down
DYNC1H1	-0.52865	9.503674	-5.38417	8.4802135	4.9257655	5.251695	Down
CHROMR	1.376034	6.065793	5.38604	8.4865094	4.9280823	5.251904	Up
HGH1	-0.59331	6.74618	-5.38198	8.5541356	4.9646529	5.243264	Down
OGFRL1	0.949039	8.634962	5.382486	8.5626743	4.9682586	5.242636	Up
XPA	-0.66692	7.351943	-5.38144	8.5725233	4.9726223	5.241179	Down
OAS1	1.370102	5.566568	5.381958	8.6195151	4.9985230	5.236723	Up
GPRC5B	-0.52821	8.541534	-5.3769	8.7287670	5.0591318	5.223634	Down
SIRT4	-0.86242	5.143249	-5.37675	8.7339429	5.0607583	5.223058	Down
MUC5AC	-1.21034	5.129944	-5.37485	8.8121958	5.1047160	5.214553	Down
FNDC5	-1.18498	10.4284	-5.37788	8.8149192	5.1049090	5.215655	Down
PDCL	0.614743	7.096214	5.373539	8.8459603	5.1214969	5.210679	Up
SEC63	0.575928	8.35741	5.372726	8.8745657	5.1366659	5.207543	Up
ZBTB17	-0.56511	6.562828	-5.37001	8.9707007	5.1894969	5.197078	Down
TMOD3	0.974619	7.128134	5.369511	8.9885884	5.1984368	5.195143	Up
KCNJ2	-0.9413	8.691446	-5.37	8.9969735	5.2018776	5.194566	Down
HSPA1A	-0.50375	11.77373	-5.36893	9.0092460	5.2075636	5.192914	Down
SPRR1A	-0.52381	4.574336	-5.36849	9.0251078	5.2139100	5.191205	Down
PROX1	-1.00566	7.578682	-5.36909	9.0299510	5.2152973	5.191015	Down
RHBDF2	-0.74366	6.13969	-5.36807	9.0403037	5.2198650	5.189571	Down
SECISBP2	-0.84055	8.544806	-5.36718	9.0722987	5.2355081	5.18614	Down
KANSL2	-0.61068	8.858033	-5.36537	9.1377523	5.2695374	5.179157	Down
KDM1A	-0.59669	7.863162	-5.36485	9.1563383	5.2783016	5.177184	Down
HIGD1B	-1.20471	5.693375	-5.36491	9.1805731	5.2908440	5.174943	Down
FRMD3	-0.7737	8.29243	-5.36306	9.2216582	5.3130881	5.17028	Down
CLN3	-0.71215	7.053732	-5.36085	9.3030068	5.3585118	5.16175	Down
SNAPC3	-0.67384	7.743088	-5.36072	9.3079035	5.3598868	5.161239	Down
MRPL58	-0.71194	8.731738	-5.36007	9.3317024	5.3721428	5.158759	Down
PLIN5	-1.08317	8.356933	-5.3597	9.4211801	5.4192718	5.150414	Down
SNRNP25	-0.71371	9.631476	-5.35751	9.4272458	5.4213009	5.148866	Down
RNASE1	0.978869	10.1582	5.358485	9.4666987	5.4425236	5.145733	Up
NUMBL	0.76152	10.76745	5.355264	9.5114494	5.4637521	5.14023	Up
DDB2	0.608014	5.589951	5.353712	9.5701804	5.4946213	5.134252	Up
CYBB	1.400381	7.042674	5.356041	9.6115420	5.5168856	5.131616	Up
HEG1	0.567152	8.995267	5.350818	9.6806827	5.5550785	5.123103	Up
GRN	0.72086	9.001203	5.349647	9.7257080	5.5794163	5.118597	Up
USP25	-0.67316	9.788861	-5.3478	9.7971811	5.6174011	5.111487	Down
TCTA	0.642104	7.093061	5.347177	9.8214109	5.6297825	5.109088	Up
MRPS15	-0.50186	11.68709	-5.34701	9.8277491	5.6319041	5.108462	Down
LDB2	0.915955	8.233744	5.346693	9.8500774	5.6431854	5.106372	Up
PNISR-AS	1.186698	5.784814	5.346895	9.8595916	5.6456074	5.105637	Up
F2R	1.28678	6.151855	5.345821	9.9409379	5.6906607	5.098114	Up
C19orf33	-1.29191	3.970729	-5.3439	9.9667593	5.7039133	5.095019	Down
STAC	-0.95267	5.610637	-5.34254	1.0003811	5.7235842	5.091219	Down
PPP2R5D	-0.51975	7.343991	-5.34149	1.0045474	5.7443441	5.087184	Down
PPT2-EGF	0.669729	6.51167	5.340887	1.0069346	5.7536455	5.084879	Up
RUBCNL	1.633339	4.726599	5.344231	1.0069822	5.7536455	5.086362	Up
SMARCD3	-0.69428	9.025069	-5.34048	1.0085424	5.7610188	5.08333	Down
BCAR1	-0.58583	6.538407	-5.34	1.0104901	5.7706010	5.081456	Down
AARSD1	-0.65721	7.587459	-5.33895	1.0146778	5.7914187	5.077441	Down
C1QTNF9	0.726835	5.46051	5.335675	1.0279437	5.8640018	5.064828	Up
ZNRF3	0.616626	6.496212	5.335362	1.0292211	5.8697212	5.063623	Up
NANS	0.547257	7.166771	5.333799	1.0356111	5.9014364	5.057613	Up
CPXM2	1.276093	6.337859	5.334694	1.0401826	5.9227459	5.054253	Up
SMAP2	0.879368	6.33209	5.332251	1.0419782	5.9313888	5.051662	Up
ACADS	-0.95613	7.546497	-5.33081	1.0491777	5.9707800	5.045112	Down

ZNF48	-0.56454	5.87898	-5.32952	1.0533002	5.9926445	5.041169	Down
ZNF658	-0.74683	5.346767	-5.32908	1.0551311	6.0014622	5.039483	Down
RP9	-0.69589	8.107934	-5.32865	1.0569291	6.0100888	5.03783	Down
ARHGAP3	0.764826	5.525161	5.328082	1.0593234	6.0204974	5.035633	Up
CCDC22	-0.5021	6.196435	-5.3277	1.0609101	6.0279112	5.03418	Down
CFAP410	-0.50008	5.838642	-5.32739	1.0622271	6.0335146	5.032975	Down
TBC1D10E	-0.62771	7.095734	-5.32538	1.0707017	6.0786942	5.02526	Down
CHDH	-1.33998	5.773359	-5.32684	1.0729279	6.0897144	5.024152	Down
OTUD3	-0.56211	5.613448	-5.32374	1.0777016	6.1151839	5.018934	Down
PPP1R18	0.824285	7.694807	5.323332	1.0794298	6.1233633	5.017378	Up
CLEC2L	-0.86337	3.224121	-5.32192	1.0854887	6.1560992	5.011944	Down
UTP20	-0.54242	5.857393	-5.31723	1.1057903	6.2645810	4.993955	Down
SEMA4A	-0.56676	4.786478	-5.31329	1.1231777	6.3613977	4.978809	Down
VARS1	-0.75905	6.380374	-5.31315	1.1237869	6.3623994	4.978283	Down
ZNF683	-1.0314	4.601687	-5.31312	1.1239504	6.3623994	4.978142	Down
MINPP1	0.614342	7.193797	5.311271	1.1321814	6.4055976	4.971059	Up
OSGIN2	1.018507	7.68765	5.310446	1.1409719	6.4536225	4.964071	Up
FABP5	0.781887	10.65921	5.306254	1.1562710	6.5384267	4.950764	Up
RAB11FIP3	1.0491	7.528001	5.307171	1.1565968	6.5385374	4.950945	Up
FHL2	-1.19952	8.932041	-5.30807	1.1617915	6.5661665	4.94753	Down
REX1BD	-0.56523	8.344215	-5.30306	1.1695506	6.6082707	4.939537	Down
UCHL1	-1.47882	7.869376	-5.30552	1.1735699	6.6292271	4.937738	Down
LRRC70	1.543164	4.068419	5.304052	1.1740880	6.6303999	4.936688	Up
GET3	-0.55181	7.689561	-5.30197	1.1745793	6.6314212	4.935373	Down
HDAC9	-0.72485	7.571439	-5.30103	1.1789712	6.6544575	4.93175	Down
GBA2	-0.79408	7.111611	-5.29981	1.1846680	6.6848457	4.927071	Down
NUCB2	0.761273	8.3539	5.298808	1.1893480	6.7059385	4.923245	Up
PINX1	-0.54402	6.445694	-5.29843	1.1911012	6.7140512	4.921815	Down
ENPEP	0.801383	6.953528	5.298008	1.1931128	6.7233494	4.920177	Up
IGF2	1.588007	11.40715	5.3036	1.1961934	6.7374210	4.920548	Up
SCAMP4	-0.59112	4.774271	-5.297	1.1978749	6.7451131	4.916311	Down
NOD1	0.604852	6.227426	5.296827	1.1986921	6.7474992	4.915649	Up
TOLLIP	-0.59845	6.433316	-5.29678	1.1989306	6.7474992	4.915456	Down
NUTM1	-0.68855	5.237478	-5.29639	1.2007807	6.7561315	4.913959	Down
PDE7B	0.584153	7.657948	5.296172	1.2017971	6.7600695	4.913138	Up
HOXB7	0.792284	5.796023	5.295929	1.2029481	6.7647621	4.912209	Up
MRPL41	-0.66095	10.78804	-5.29454	1.2095883	6.8003124	4.906866	Down
FBLN7	1.107873	5.255822	5.29352	1.2144498	6.8222565	4.902973	Up
PIP5K1A	-0.66134	7.335624	-5.29342	1.2149202	6.8231045	4.902597	Down
OSGIN1	-1.0046	4.103624	-5.29235	1.2200869	6.8503200	4.898479	Down
NPR1	-0.69819	6.660304	-5.29136	1.2248718	6.8742094	4.89468	Down
MEIS3P1	0.716166	7.273711	5.290872	1.2272170	6.8831146	4.892824	Up
CHUK	-0.51305	6.764483	-5.29054	1.2288189	6.8902902	4.891558	Down
XIRP1	-1.1599	11.20159	-5.29255	1.2351334	6.9220627	4.888109	Down
PRX	-1.03074	5.68294	-5.28913	1.2356991	6.9234160	4.886139	Down
BRF1	-0.60929	4.865869	-5.28842	1.2391468	6.9390926	4.883435	Down
EPO	-0.92051	4.246972	-5.28729	1.2446729	6.9682110	4.879116	Down
SNAPC5	-0.52256	9.096527	-5.28529	1.2545757	7.0218105	4.871426	Down
SNX21	0.590276	8.108094	5.283291	1.2644954	7.0754764	4.863783	Up
CNN3-DT	1.314868	4.823242	5.283579	1.2694591	7.0995310	4.860573	Up
SETD5	0.554903	9.124995	5.280905	1.2764565	7.1367956	4.854646	Up
LZTS3	-0.67471	5.949805	-5.28075	1.2772365	7.1392878	4.854053	Down
ALYREF	-0.84962	7.412443	-5.27938	1.2841461	7.1760317	4.848818	Down
SFXN3	0.766649	6.993642	5.278506	1.2885951	7.1990097	4.845462	Up
GSTP1	-0.55644	9.060461	-5.27788	1.2917985	7.2150193	4.843052	Down
S1PR4	-1.03039	5.677837	-5.27697	1.2964099	7.2388819	4.839594	Down
LVRN	1.458361	3.79598	5.277548	1.3016292	7.2661258	4.836433	Up
CARHSP1	1.032062	6.534962	5.274378	1.3097441	7.3095154	4.829664	Up

PPP6R2	-0.55522	7.137851	-5.27373	1.3131224	7.3264548	4.827165	Down
VSNL1	-0.56265	4.437627	-5.27289	1.3174371	7.3486087	4.823982	Down
ZNF487	0.628943	5.368899	5.272535	1.3192991	7.3570735	4.822611	Up
CDC37	-0.59079	9.16605	-5.272	1.3220881	7.3707019	4.820562	Down
TUBA4B	-0.85936	5.959459	-5.27119	1.3263391	7.3924717	4.817447	Down
LRTM1	1.880912	4.38502	5.274136	1.3280387	7.4000134	4.817722	Up
TUBB2B	0.947101	7.654349	5.269994	1.3344991	7.4301962	4.811665	Up
FMN2	-1.29408	4.979343	-5.26983	1.3401746	7.4598512	4.807968	Down
CPA5	-0.62702	4.947772	-5.26847	1.3406146	7.4603564	4.807059	Down
TAPT1	-0.60199	9.432332	-5.26812	1.3424865	7.4668821	4.805705	Down
HEXB	0.618323	9.127137	5.267831	1.3439922	7.4724207	4.804618	Up
GPT2	-1.1251	8.376321	-5.26936	1.3461601	7.4814695	4.803954	Down
SPPL2B	-0.82541	5.956398	-5.26593	1.3541211	7.5237566	4.797333	Down
LOC1005C	-2.45237	6.816447	-5.2714	1.3576898	7.5416230	4.797627	Down
TMEM68	0.787425	4.979732	5.264635	1.3610277	7.5581980	4.792396	Up
SAE1	0.513171	8.065998	5.264557	1.3614498	7.5585772	4.792095	Up
HIRIP3	-0.59249	7.171659	-5.26447	1.3619384	7.5593247	4.791747	Down
DYRK4	-0.5012	7.000675	-5.26369	1.3660818	7.5803523	4.7888	Down
SPOCK2	-0.74708	5.532218	-5.26241	1.3730413	7.6169914	4.78387	Down
TMEM43	0.599045	8.859222	5.262001	1.3752327	7.6271669	4.782322	Up
NET1	0.609502	8.501075	5.257746	1.3984746	7.7500312	4.766062	Up
COL16A1	1.032771	6.991747	5.257893	1.4014176	7.7643264	4.764339	Up
H4C3	0.610298	9.233109	5.256102	1.4075587	7.7935451	4.759781	Up
HSPG2	0.814008	9.728035	5.25607	1.4077391	7.7935451	4.759656	Up
HMGCLL1	1.935126	4.915923	5.253231	1.4418405	7.9779355	4.737944	Up
MCTP1	1.902972	6.493626	5.251626	1.4595215	8.0736756	4.726815	Up
KCNF1	-1.02446	3.744499	-5.24565	1.4666845	8.1086473	4.719862	Down
DDA1	-0.64006	7.607979	-5.24513	1.4696673	8.1213856	4.717891	Down
MXD1	-0.71029	6.418109	-5.2449	1.4710118	8.1267128	4.717004	Down
TBRG1	0.727116	7.869787	5.244248	1.4747991	8.1455287	4.71451	Up
IL16	0.576743	6.14824	5.242109	1.4872619	8.2122389	4.706347	Up
GREM1	-2.05306	3.239911	-5.24416	1.4941396	8.2459522	4.703376	Down
MIX23	-1.1813	6.150648	-5.24144	1.4984401	8.2675494	4.699659	Down
BIRC3	0.99062	6.016352	5.239382	1.5033049	8.2922486	4.69594	Up
ANKRD9	-0.79573	10.49789	-5.23878	1.5090776	8.3161071	4.692394	Down
GIMAP1	0.77593	7.567322	5.238389	1.5091877	8.3161071	4.692151	Up
NKAPD1	-0.73718	7.041796	-5.23686	1.5182939	8.3641275	4.686316	Down
DGCR6	-0.76671	9.294013	-5.23554	1.5262189	8.4034505	4.681266	Down
CASP3	0.774758	7.232279	5.235259	1.5278827	8.4104434	4.680209	Up
PARPBP	1.037358	3.799943	5.234691	1.5312979	8.4248997	4.678044	Up
ADAM28	2.074123	3.855492	5.237622	1.5334863	8.4347672	4.678198	Up
YKT6	-0.65114	6.215429	-5.23327	1.5398866	8.4677907	4.672619	Down
B3GALT6	0.540643	6.484641	5.233141	1.5406549	8.4698345	4.672135	Up
DAB1	-0.75873	8.268716	-5.23234	1.5454974	8.4920847	4.669091	Down
NPL	1.523097	6.071272	5.235375	1.5465845	8.4958726	4.66991	Up
CS	-0.52838	11.58888	-5.23184	1.5485755	8.5046224	4.667161	Down
CYP1B1	1.569029	7.112763	5.234218	1.5536182	8.5301232	4.665508	Up
MRPL35	-0.66573	8.479387	-5.23049	1.5568152	8.5454792	4.662014	Down
DENND4A	0.655375	5.952634	5.230399	1.5573519	8.5462288	4.66168	Up
ACTR3	0.535513	10.31107	5.230019	1.5596789	8.5567999	4.660231	Up
C14orf18C	-1.15547	5.409729	-5.22948	1.5649557	8.5813415	4.657104	Down
CILK1	0.691215	6.406967	5.228849	1.5668686	8.5896252	4.655771	Up
TLR4	0.740694	5.804507	5.22847	1.5692035	8.5980115	4.654326	Up
BEGAIN	-0.72352	3.987182	-5.22757	1.5747504	8.6261915	4.650904	Down
DNLZ	-0.82448	5.476454	-5.22664	1.5805617	8.6558043	4.647331	Down
CENPC	0.808547	5.504399	5.223906	1.5976040	8.7446507	4.63693	Up
EIF2D	-0.56812	8.730451	-5.22256	1.6060950	8.7888747	4.631789	Down
BBS5	-0.71354	3.998085	-5.22124	1.6144393	8.8322735	4.626763	Down

DLEU2	0.902978	8.425908	5.220254	1.6230185	8.8746620	4.621795	Up
CCDC9B	-0.69834	8.306565	-5.21775	1.6367263	8.9473256	4.613467	Down
C19orf48F	0.936948	4.517465	5.216966	1.6417520	8.9725026	4.610494	Up
CEBPG	-0.63502	8.772451	-5.21561	1.6505183	9.0181046	4.605329	Down
MITD1	0.62786	7.320957	5.21533	1.6523300	9.0256944	4.604265	Up
PPIP5K1	-0.68029	5.561033	-5.21402	1.6608467	9.0675780	4.59928	Down
BCL10	0.619807	7.461589	5.213817	1.6621768	9.0725206	4.598504	Up
OLFML3	1.127737	8.327914	5.214741	1.6684696	9.1045415	4.595725	Up
ACTC1	1.922302	12.53866	5.219003	1.6798517	9.1643099	4.591946	Up
ANKRD6	0.570985	6.024534	5.209569	1.6901242	9.2156431	4.582334	Up
GON7	-0.50046	7.652669	-5.20431	1.7253351	9.4028345	4.56234	Down
FATE1	-0.57843	4.34794	-5.20424	1.7258239	9.4030987	4.562066	Down
TMEM109	-0.53839	8.596309	-5.20164	1.7434978	9.4969721	4.552186	Down
MRPL12	-0.92476	8.73375	-5.20185	1.7480760	9.5194821	4.550057	Down
CA11	-0.56624	5.486916	-5.20021	1.7533669	9.5458608	4.546713	Down
TMC8	0.774134	4.532093	5.199566	1.7577647	9.5649270	4.544284	Up
MMP11	-0.73095	4.497487	-5.19935	1.7592840	9.5707561	4.543447	Down
CPNE7	-0.65205	6.300037	-5.19899	1.7617287	9.5816149	4.5421	Down
FZD10-AS	0.744218	4.774714	5.196552	1.7786672	9.6712768	4.532823	Up
HLA-DRB6	1.145079	8.326866	5.198513	1.7806008	9.6793262	4.532825	Up
SDHB	-0.5533	10.95134	-5.196	1.7825242	9.6873165	4.530722	Down
SNX1	0.78329	7.921436	5.194134	1.7956055	9.7559256	4.523633	Up
SKI	0.811797	8.368682	5.193635	1.7991180	9.7725236	4.521738	Up
CYP26B1	2.011092	5.19105	5.196969	1.8022754	9.7871849	4.521822	Up
ARIH2	-0.72948	9.553023	-5.19272	1.8055975	9.8027331	4.518253	Down
TRPT1	-0.79729	8.853055	-5.19236	1.8081251	9.8139611	4.516897	Down
TLR3	0.957944	5.243643	5.191993	1.8107344	9.8256264	4.515499	Up
DDX60	0.924247	7.002546	5.191924	1.8112267	9.8258018	4.515235	Up
TSPOAP1	-0.91228	3.97667	-5.1916	1.8135388	9.8333491	4.513998	Down
ARHGEF37	-0.94659	7.608753	-5.18955	1.8315019	9.9282283	4.504664	Down
CCDC124	-0.57329	6.826046	-5.18824	1.8375870	9.9586869	4.501227	Down
TAF1A	0.967338	3.902879	5.188021	1.8391450	9.9646017	4.500405	Up
TRIM16	-0.96036	7.32485	-5.18789	1.8425888	9.9807284	4.498757	Down
SECTM1	1.384296	5.490479	5.187202	1.8585462	1.0062961	4.491111	Up
LYST	0.955916	6.734186	5.18267	1.8780978	1.0164953	4.480087	Up
ZNG1A	0.67092	9.579023	5.182615	1.8785032	1.0164953	4.479877	Up
RBPMS2	-1.02417	5.208734	-5.17975	1.8997041	1.0271868	4.468998	Down
MORN2	-0.53588	7.093521	-5.17956	1.9011387	1.0277023	4.468266	Down
GRB10	-0.80963	9.043132	-5.17865	1.9078750	1.0310828	4.464837	Down
IPO11	-0.53761	6.6423	-5.17724	1.9184691	1.0365459	4.45947	Down
TNFRSF18	-0.60196	4.393036	-5.17652	1.9238626	1.0391972	4.456748	Down
SAP130	-0.57815	6.820027	-5.17357	1.9462510	1.0510247	4.445533	Down
PKDCC	-1.40131	8.195211	-5.17615	1.9502440	1.0529148	4.445022	Down
AVPR1A	1.03166	6.25363	5.172544	1.9540497	1.0545523	4.441656	Up
CASTOR3I	-0.93769	8.060942	-5.17318	1.9542645	1.0545523	4.441863	Down
C2CD5	0.768333	8.39749	5.171782	1.9598878	1.0573196	4.438765	Up
DNAJA2	-0.64607	9.02625	-5.16967	1.9761213	1.0658080	4.430769	Down
BNIP1	-0.6919	6.137322	-5.16889	1.9821860	1.0688091	4.427799	Down
FNDC8	-0.87436	3.719925	-5.16811	1.9882281	1.0717965	4.424849	Down
EDNRB	0.790311	6.655257	5.167302	1.9945388	1.0746559	4.421777	Up
KCNA6	-0.59544	5.516713	-5.16707	1.9963329	1.0753097	4.420906	Down
APOL1	1.164014	5.484127	5.167619	1.9967593	1.0753097	4.420984	Up
BCKDK	-0.72313	8.20258	-5.16674	1.9989559	1.0762213	4.419633	Down
CCDC18-/	1.105204	7.586997	5.167902	2.0042488	1.0787990	4.417945	Up
MARCKSL	1.073569	7.381482	5.167249	2.0067336	1.0798643	4.416584	Up
WDFY4	1.088816	4.904112	5.165125	2.0115973	1.0822089	4.413523	Up
TRIM56	0.582102	7.249165	5.164989	2.0126650	1.0825107	4.413009	Up
MPV17L2	-0.60167	5.535442	-5.16466	2.0152256	1.0833423	4.411776	Down

PPP2R2D	-0.72713	6.96072	-5.16405	2.0200761	1.0856765	4.409446	Down
RNF41	-0.71972	7.206136	-5.16333	2.0258066	1.0884621	4.406701	Down
DDAH1	0.964395	9.662626	5.165079	2.0262784	1.0884621	4.407337	Up
PANK4	-0.6534	7.769557	-5.1628	2.0300081	1.0901914	4.404693	Down
PLA2G4A	1.103191	4.736941	5.161822	2.0377495	1.0940737	4.401004	Up
DDIT4L	1.055719	11.10603	5.164771	2.0389019	1.0944173	4.401927	Up
IFNL1	-0.84862	4.131621	-5.16142	2.0409409	1.0952365	4.399487	Down
SAMTOR	0.567934	6.568266	5.160392	2.0491765	1.0991037	4.395584	Up
PLCD3	-1.01459	7.300275	-5.15958	2.0635711	1.1065466	4.389266	Down
CDK14	0.700475	7.476887	5.15746	2.0727951	1.1112138	4.384477	Up
COA6	-0.54077	9.387583	-5.15734	2.0737316	1.1114368	4.38404	Down
AKAP8L	-0.60075	6.594627	-5.15551	2.0886814	1.1191685	4.377078	Down
UCKL1	-0.68973	8.698012	-5.15467	2.0955089	1.1223276	4.373915	Down
DYNC2I1	-0.61001	6.257344	-5.15466	2.0956281	1.1223276	4.37386	Down
LILRB2	1.824003	5.321901	5.156531	2.1055325	1.1270667	4.370758	Up
PATL1	-0.61798	7.737336	-5.15324	2.1072842	1.1277218	4.368485	Down
ADAM15	-0.63735	5.949202	-5.1524	2.1141649	1.1305542	4.365326	Down
GPX7	0.710974	6.839314	5.151855	2.1187058	1.1324154	4.363246	Up
KCTD21	-0.65082	5.55844	-5.15147	2.1218578	1.1338163	4.361806	Down
HCFC1R1	-0.83079	10.11026	-5.15105	2.1307228	1.1382686	4.358074	Down
NUTM2F	-1.11837	3.586484	-5.15012	2.1331367	1.1392731	4.356668	Down
NAP1L5	0.703542	6.946595	5.149922	2.1347627	1.1398565	4.35593	Up
FRZB	2.227849	9.758662	5.154511	2.1447031	1.1448739	4.354182	Up
ECPAS	-0.57167	10.11304	-5.14867	2.1452315	1.1448739	4.351189	Down
H2AC25	1.097541	5.618397	5.148076	2.1502131	1.1472458	4.348941	Up
SEMA6A	1.096023	6.783679	5.148578	2.1560330	1.1500637	4.346892	Up
RMI2	0.893467	4.897821	5.14683	2.1607063	1.1522687	4.344224	Up
WDR54	0.704472	4.667751	5.146466	2.1637813	1.1536206	4.342846	Up
ACP5	2.426157	4.504817	5.151737	2.1679974	1.1555799	4.343711	Up
RRM2B	-0.62584	8.292055	-5.14585	2.1690209	1.1558370	4.340502	Down
PMS2P6	-0.61401	4.323949	-5.14328	2.1908664	1.1668959	4.330791	Down
GBE1	-0.5687	10.97699	-5.14289	2.1941824	1.1683706	4.329326	Down
GRM8	-0.88189	3.842557	-5.14071	2.2129843	1.1780887	4.321058	Down
TTI2	-0.56526	6.041487	-5.13948	2.2235845	1.1831419	4.316428	Down
ZNF2	0.530709	4.863475	5.138391	2.2330777	1.1878972	4.3123	Up
FAM210A	-0.77552	8.212709	-5.1353	2.2601585	1.2017044	4.30062	Down
SHISA3	-0.6034	4.266693	-5.1347	2.2654835	1.2042358	4.29834	Down
CAMK2N1	0.563407	7.486734	5.134227	2.2696612	1.2061563	4.296555	Up
ZBTB44	-0.58284	10.24988	-5.13167	2.2924519	1.2179649	4.286874	Down
SIK1	-0.9311	4.184058	-5.12883	2.3179095	1.2311840	4.276174	Down
KIFC2	-0.56176	5.105732	-5.12858	2.3202087	1.2319548	4.275214	Down
CASQ2	0.698735	11.97645	5.129299	2.3205142	1.2319548	4.275446	Up
LRR1	0.602634	5.616519	5.128262	2.3230911	1.2327400	4.274011	Up
MAPRE2	-0.61957	8.513323	-5.12826	2.3231477	1.2327400	4.273987	Down
SFMBT2	0.822893	5.712087	5.128048	2.3250358	1.2334355	4.2732	Up
RBL1	-0.55894	5.827685	-5.12733	2.3315298	1.2365735	4.270498	Down
LBP	-1.33663	4.811164	-5.12847	2.3346087	1.2376557	4.269926	Down
CHN2	1.144623	5.119019	5.127205	2.3347293	1.2376557	4.269277	Up
PLAA	-0.50638	7.773999	-5.12514	2.3515129	1.2462434	4.26223	Down
PARS2	-0.83334	5.870139	-5.12208	2.3797260	1.2608829	4.250675	Down
YIF1B	-0.55602	6.764994	-5.11979	2.4010836	1.2718835	4.24202	Down
TESC	-0.74697	4.842006	-5.11603	2.4365247	1.2900172	4.227825	Down
ARL8B	-0.50713	9.848451	-5.11437	2.4523292	1.2977415	4.221562	Down
IFIH1	0.976531	6.511248	5.112561	2.4696967	1.3062848	4.214726	Up
SLC30A4	0.867486	6.486571	5.11071	2.4875614	1.3154082	4.207745	Up
GPR65	1.43017	5.104162	5.112307	2.4894116	1.3160607	4.207885	Up
RMND5A	-0.54746	9.322349	-5.11021	2.4923754	1.3169756	4.205872	Down
FASTKD5	-0.66765	7.440699	-5.11008	2.4936528	1.3173247	4.205375	Down

FHIT	-0.77046	4.427934	-5.10819	2.5120736	1.3263482	4.198246	Down
PWRN1	-0.7152	4.351444	-5.10814	2.5125972	1.3263482	4.198045	Down
NEK6	0.969225	5.344967	5.107909	2.5148427	1.3268777	4.197179	Up
GBA1	0.588434	5.223624	5.106755	2.5261674	1.3325236	4.192827	Up
POU6F1	-0.58262	6.09398	-5.10254	2.5679225	1.3542145	4.176948	Down
KLF6	0.784746	9.682009	5.10033	2.5901423	1.3652581	4.168604	Up
DIAPH1	-0.75952	8.1555	-5.09719	2.6219294	1.3811321	4.15679	Down
MED29	0.55345	6.484447	5.097168	2.6221982	1.3811321	4.156691	Up
PATZ1	-0.62513	6.553317	-5.09684	2.6255967	1.3825812	4.155436	Down
PHF14	0.627339	7.54457	5.096489	2.6291308	1.3834189	4.154134	Up
AVEN	-0.52681	7.624847	-5.09492	2.6451941	1.3911858	4.148235	Down
GZMK	1.221061	4.711695	5.094933	2.6531942	1.3950498	4.14569	Up
IQCJ-SCH	-0.7895	4.853125	-5.09182	2.6773267	1.4070458	4.136541	Down
C10orf143	-0.68834	5.716907	-5.09088	2.6871368	1.4118540	4.132999	Down
GLIDR	0.589619	6.328893	5.090119	2.6950809	1.4153316	4.13014	Up
PRKAR1A	0.776796	11.43724	5.090779	2.7017989	1.4185108	4.128358	Up
SHE	0.545053	7.557265	5.089358	2.7030604	1.4185718	4.127277	Up
APAF1	0.883412	5.059964	5.089341	2.7032435	1.4185718	4.127212	Up
UMPS	0.527205	6.182386	5.08696	2.7283774	1.4314095	4.118249	Up
TMEM255	1.396018	4.306051	5.088619	2.7295039	1.4316489	4.118704	Up
MYF6	0.540857	11.56617	5.084952	2.7497545	1.4415938	4.110692	Up
FREM1	1.207865	5.064073	5.085729	2.7498142	1.4415938	4.11105	Up
MATK	-0.89164	4.540237	-5.08449	2.7546978	1.4437996	4.108952	Down
WNK2	-1.18914	6.760482	-5.08533	2.7646285	1.4486490	4.106321	Down
TRIM6	0.878671	4.410591	5.080884	2.7935718	1.4630973	4.095383	Up
SEC24A	0.733021	7.486265	5.079688	2.8065832	1.4695514	4.090883	Up
THEM4	-0.51611	7.588222	-5.07825	2.8222765	1.4770444	4.085484	Down
KDM6A	-0.73756	6.590378	-5.07643	2.8423567	1.4864606	4.078619	Down
DYNC2H1	1.151062	5.011673	5.076313	2.8472272	1.4886433	4.07712	Up
NEK3	0.833675	5.993632	5.075298	2.8548402	1.4922584	4.074376	Up
FUZ	-1.02299	6.053056	-5.07497	2.8584609	1.4937854	4.073149	Down
PRR34-AS	-0.54304	9.830565	-5.07353	2.8744912	1.5017951	4.067734	Down
TPST2	-0.82837	7.03394	-5.07296	2.8808411	1.5047445	4.065597	Down
POLR2I	-0.57843	10.29004	-5.07164	2.8956459	1.5121077	4.060634	Down
PGM5	-0.81854	8.782501	-5.07067	2.9065740	1.5174433	4.056987	Down
MARCHF5	-0.68006	7.270567	-5.07042	2.9093821	1.5185382	4.056052	Down
SCAF4	-0.63569	6.408664	-5.07017	2.9122747	1.5196766	4.05509	Down
TMEM38A	-0.74503	10.60905	-5.06782	2.9409971	1.5339149	4.045674	Down
FAH	-0.85818	7.291858	-5.06663	2.9525969	1.5388376	4.041777	Down
DNMT3B	0.72752	4.084167	5.066081	2.9588430	1.5417167	4.039731	Up
BAZ1A	0.87177	7.430828	5.064805	2.9735222	1.5489875	4.03494	Up
RRAS2	-0.58738	9.482945	-5.06468	2.9749975	1.5493781	4.03446	Down
MOSPD2	0.629711	5.618219	5.062016	3.0058682	1.5645557	4.024466	Up
SIX4	-0.63066	8.675866	-5.06198	3.0063380	1.5645557	4.024314	Down
CYRIB	0.75365	8.379051	5.060972	3.0180712	1.5698968	4.020543	Up
ZKSCAN1	0.522172	8.720129	5.059747	3.0324404	1.5769871	4.015945	Up
NUDT19	-0.50486	9.200458	-5.05905	3.0407067	1.5801317	4.01331	Down
HEXD	-0.58091	6.056193	-5.0576	3.0578340	1.5878730	4.007872	Down
FAM201B	-0.64417	3.515144	-5.05732	3.0611027	1.5891840	4.006838	Down
HLA-DOA	1.103495	6.020779	5.057231	3.0694581	1.5931345	4.004498	Up
AIRIM	0.533375	7.539708	5.056298	3.0732694	1.5947251	4.002998	Up
ADAM12	2.338061	4.47386	5.060354	3.0904599	1.6032558	4.000277	Up
COQ8A	-0.71053	11.52762	-5.05505	3.0954827	1.6050818	3.996325	Down
DISC1	0.833075	5.245083	5.053894	3.1020506	1.6080970	3.993975	Up
MISP	-0.54615	5.583382	-5.05312	3.1113689	1.6117539	3.991071	Down
BGN	1.498777	7.735715	5.055385	3.1234102	1.6175993	3.988924	Up
CECR2	-0.64428	7.619408	-5.05157	3.1301551	1.6206994	3.985244	Down
HBB	-1.52411	12.40655	-5.05677	3.1335572	1.6220677	3.986868	Down

AMOTL2	0.922497	8.048621	5.051462	3.1407706	1.6254077	3.982342	Up
FAM99B	-0.70206	5.463329	-5.05042	3.1440881	1.6267304	3.980945	Down
LRP3	-0.63616	6.384156	-5.05	3.1491680	1.6289641	3.979382	Down
DPPA2	-0.57042	4.17794	-5.04974	3.1524347	1.6302591	3.978379	Down
LINC0089!	1.107658	4.765015	5.049479	3.1555946	1.6314981	3.977409	Up
TCF15	-1.00517	6.131893	-5.04936	3.1569965	1.6315343	3.976979	Down
APOC1	1.070497	8.055254	5.050945	3.1588155	1.6319783	3.977266	Up
PCNT	-0.63122	9.76778	-5.04607	3.1975059	1.6507694	3.964638	Down
ADGRL4	0.892112	8.252033	5.045898	3.2069741	1.6552574	3.962063	Up
SUGCT	-0.90808	5.459872	-5.04441	3.2182201	1.6606606	3.958387	Down
PLPP6	0.674292	5.369543	5.043388	3.2309367	1.6660151	3.95457	Up
ERG	0.689301	6.223883	5.042542	3.2415437	1.6702748	3.951398	Up
FZD4	0.665281	8.788374	5.042463	3.2425332	1.6703816	3.951103	Up
ASAP3	-0.6911	7.167581	-5.04213	3.2467225	1.6720290	3.949853	Down
OSBPL3	0.905642	5.6576	5.042084	3.2472967	1.6720290	3.949682	Up
NCK1-DT	-0.56128	5.899941	-5.04092	3.2620259	1.6792082	3.945302	Down
HK2	-1.01712	8.153004	-5.04237	3.2642562	1.6799514	3.94544	Down
S100A6	1.085266	11.40297	5.042356	3.2804751	1.6870789	3.941261	Up
RABGGTA	-0.60473	5.804866	-5.03787	3.3007382	1.6967699	3.933883	Down
DLG5	-0.65576	8.190706	-5.03786	3.3009078	1.6967699	3.933833	Down
PACS1	-0.71562	6.661812	-5.0375	3.3054716	1.6987070	3.932496	Down
LOC25489	-0.85888	4.405378	-5.03631	3.3207604	1.7061535	3.92803	Down
LINC0269:	-0.60825	6.502184	-5.03619	3.3222917	1.7065297	3.927584	Down
PDGFA	-0.90621	6.718745	-5.03537	3.3328143	1.7107005	3.924523	Down
BTN3A1	0.685121	6.84357	5.033118	3.3619892	1.7244324	3.916088	Up
MANF	0.581054	7.260646	5.033032	3.3631119	1.7244577	3.915765	Up
PDIK1L	-0.57933	6.44413	-5.03299	3.3636534	1.7244577	3.91561	Down
KIF26A	0.672807	6.31123	5.032268	3.3730721	1.7288714	3.912903	Up
RHO	-0.77134	4.846242	-5.03199	3.3767588	1.7303458	3.911846	Down
IMPA2	-1.12587	9.397182	-5.03472	3.3787169	1.7309339	3.9127	Down
PPM1F	0.517167	7.623544	5.031135	3.3878887	1.7352165	3.908662	Up
SLC17A5	0.647544	6.828048	5.030897	3.3910136	1.7364006	3.907769	Up
PCTP	-0.58093	7.136918	-5.03041	3.3973942	1.7392508	3.90595	Down
SUPV3L1	-0.64261	7.579774	-5.02798	3.4294560	1.7548232	3.89686	Down
AXL	0.774428	8.166789	5.026845	3.4445814	1.7621405	3.892602	Up
AFF4	0.503127	8.997438	5.025964	3.4563328	1.7673056	3.889306	Up
PHTF2	-0.83082	10.48249	-5.027	3.4577884	1.7676267	3.889463	Down
ZMYM6	0.783631	6.234057	5.024619	3.4743590	1.7756726	3.884272	Up
LACTB	-0.60234	8.665941	-5.02386	3.4845666	1.7804634	3.881433	Down
ATXN7L1	0.71512	5.104303	5.021728	3.5134260	1.7943508	3.873452	Up
SLC66A1	-0.68347	4.614036	-5.02134	3.5186897	1.7966095	3.872004	Down
C19orf47	-0.64234	5.331342	-5.02065	3.5281549	1.8010117	3.869404	Down
HECTD1	-0.51125	10.82937	-5.02018	3.5345745	1.8038576	3.867645	Down
ZNF655	0.54462	8.721212	5.017954	3.5650585	1.8189804	3.859336	Up
LDAH	0.89882	4.806038	5.016765	3.5814768	1.8269210	3.85489	Up
TBCEL	0.647507	7.81759	5.016569	3.5841921	1.8278695	3.854157	Up
ZBTB43	-0.84827	7.891715	-5.01461	3.6114500	1.8408914	3.846826	Down
LZTS1	1.054771	4.19024	5.013125	3.6321945	1.8501409	3.841284	Up
ATF5	0.862624	6.708179	5.012876	3.6357033	1.8514867	3.84035	Up
CHODL	1.908076	6.054459	5.01665	3.6413504	1.8539204	3.840905	Up
CCDC174	-0.5467	6.059918	-5.01022	3.6732308	1.8688152	3.830414	Down
IRX3	-1.35291	7.27695	-5.01264	3.6793428	1.8714790	3.830211	Down
ITM2C	0.598462	6.740778	5.006539	3.7257796	1.8946476	3.816672	Up
RSAD2	1.124968	6.756321	5.007153	3.7368521	1.8993738	3.814483	Up
MMACHC	-0.66639	5.778904	-5.00558	3.7395731	1.9003046	3.813096	Down
SLC25A12	-0.75038	10.63072	-5.00492	3.7538090	1.9066314	3.809581	Down
MSL2	-0.64656	6.877834	-5.00402	3.7622553	1.9104671	3.807246	Down
WDR24	-0.57185	4.443022	-5.00259	3.7829800	1.9205345	3.801932	Down

KRTAP10-	-0.66307	5.537498	-5.00018	3.8184030	1.9377039	3.792915	Down
ADGRE5	0.614971	7.269766	5.000105	3.8194712	1.9377039	3.792645	Up
REPIN1	-0.51964	9.691509	-5.0001	3.8195213	1.9377039	3.792632	Down
MDN1	-0.53926	7.681821	-4.99922	3.8325979	1.9438761	3.789326	Down
PPIP5K2	1.5757	6.357717	5.001299	3.8437087	1.9490486	3.787926	Up
LILRB5	1.230679	4.883649	4.999226	3.8485168	1.9510235	3.785852	Up
KDM8	-0.67323	5.237783	-4.99764	3.8560512	1.9543792	3.783424	Down
COQ3	-0.60263	8.639368	-4.99612	3.8786623	1.9653729	3.777768	Down
HLA-F-AS	0.762703	8.063865	4.992249	3.9370033	1.9932533	3.763326	Up
PLAAT1	-0.83875	8.593208	-4.99243	3.9374172	1.9932533	3.763328	Down
CAPN10	-0.58953	4.519282	-4.99204	3.9401852	1.9941819	3.762545	Down
RAB13	0.610778	9.405869	4.990605	3.9620412	2.0047684	3.757194	Up
DNAJA3	-0.62118	9.556058	-4.98869	3.9914193	2.0186771	3.750048	Down
USP10	-0.54756	7.726709	-4.98699	4.0176602	2.0305060	3.74371	Down
JPT1	0.839324	9.36658	4.986794	4.0298264	2.0361730	3.741078	Up
BAZ2B-AS	-0.7123	6.686273	-4.98479	4.0518931	2.0468385	3.735504	Down
TUT4	-0.62307	7.616566	-4.98433	4.0590994	2.0499939	3.733785	Down
PWAR5	-1.2775	5.168873	-4.98532	4.0648942	2.0524352	3.733082	Down
MRPL43	-0.61801	8.412362	-4.98221	4.0922966	2.0657828	3.725907	Down
NREP	0.863702	11.15729	4.982205	4.1187885	2.0781735	3.720493	Up
CSRNP1	-0.87882	5.945292	-4.9802	4.1241912	2.0804080	3.718398	Down
TFPI	0.68418	9.14982	4.980045	4.1266318	2.0811476	3.717826	Up
MAPK8IP1	-0.94588	5.544976	-4.97913	4.1412113	2.0880073	3.714415	Down
EFCAB13	-0.94457	3.866106	-4.97717	4.1725247	2.1028028	3.70713	Down
ANO5	-0.70013	9.870104	-4.97681	4.1783105	2.1052219	3.70579	Down
ADAMTS1	0.795951	4.660389	4.975127	4.2055415	2.1184423	3.699507	Up
RNA45SN	-0.92046	5.602438	-4.9744	4.2172737	2.1234679	3.696813	Down
AIG1	0.736241	8.433152	4.97439	4.2175068	2.1234679	3.69676	Up
SLC7A5	-0.87362	5.860784	-4.97425	4.2197905	2.1239912	3.696236	Down
GADD45G	-0.52633	8.216315	-4.9742	4.2205351	2.1239912	3.696066	Down
PAQR4	-0.72557	6.02895	-4.97344	4.2330344	2.1292781	3.693206	Down
SLC18B1	0.903593	6.817922	4.972836	4.2428188	2.1336973	3.690973	Up
TSLP	-0.96412	4.418238	-4.97155	4.2638624	2.1437752	3.686188	Down
PTCD3	-0.60727	8.866001	-4.97129	4.2681091	2.1454054	3.685226	Down
WDR47	-0.55806	8.258754	-4.97039	4.2828980	2.1523326	3.681881	Down
PHKA1	-1.26843	8.957657	-4.9716	4.3090615	2.1649714	3.677379	Down
LINC0301	1.143318	5.011401	4.968668	4.3191616	2.1695356	3.673959	Up
MICAL3	-0.69892	6.043426	-4.96749	4.3310145	2.1745020	3.671077	Down
APOBEC3I	-0.73961	4.712736	-4.966	4.3558705	2.1864320	3.665543	Down
OLIG1	-1.60272	4.40573	-4.96778	4.3727348	2.1943813	3.663193	Down
ARHGAP2	-1.02224	4.278809	-4.96233	4.4178624	2.2165070	3.651879	Down
LIME1	-0.78678	4.691182	-4.9596	4.4645293	2.2393945	3.641718	Down
PRKAR2B	1.824259	7.225858	4.964535	4.4693245	2.2412733	3.643281	Up
ADAT2	1.111823	4.368433	4.959222	4.4709931	2.2415838	3.640319	Up
GATM	0.915362	9.907467	4.960443	4.4781265	2.2446333	3.639594	Up
NAT16	-0.69796	6.738448	-4.95848	4.4837629	2.2469312	3.637562	Down
RRP9	-0.83124	4.641702	-4.9583	4.4869156	2.2479836	3.636882	Down
BIRC7	-0.99359	4.128627	-4.95737	4.5029625	2.2554942	3.63343	Down
NUDT13	-0.70009	5.823146	-4.95721	4.5058007	2.2563867	3.632821	Down
SLFN12	0.971853	4.37112	4.956999	4.5093807	2.2576501	3.632053	Up
TFEB	-0.70648	7.712153	-4.95542	4.5369073	2.2703670	3.626169	Down
LRIF1	-0.52158	7.39149	-4.95526	4.5396336	2.2711992	3.625588	Down
AFAP1	0.781996	4.886911	4.950692	4.6200460	2.3103475	3.608612	Up
AKNA	0.902371	4.798812	4.949183	4.6469022	2.3226898	3.603008	Up
GPR27	-0.63781	5.295098	-4.94823	4.6639025	2.3300966	3.599478	Down
MRPS7	-0.71646	9.278187	-4.94761	4.6751241	2.3346107	3.597155	Down
PTPRO	-0.5974	13.12425	-4.94762	4.6853441	2.3386207	3.595332	Down
TECRL	-1.52162	7.336963	-4.94896	4.7061770	2.3479217	3.592295	Down

YPEL1	0.880797	6.485819	4.945663	4.7101777	2.3493689	3.589933	Up
PROM1	1.179319	5.651285	4.945703	4.7288509	2.3581321	3.586644	Up
IBA57	-0.93436	6.462608	-4.94455	4.7303086	2.3583084	3.58581	Down
IP6K3	-0.91474	8.816357	-4.94548	4.7342764	2.3594526	3.585571	Down
PLPP1	0.541666	10.28472	4.944305	4.7348132	2.3594526	3.58489	Up
COPS3	-0.54129	9.824481	-4.9427	4.7639966	2.3734415	3.57895	Down
ARHGAP2	0.649769	8.217271	4.942395	4.7696660	2.3757119	3.5778	Up
MIS18A	0.582935	5.639133	4.94219	4.7734259	2.3770303	3.577038	Up
PLCG2	0.579786	5.975314	4.939679	4.8196701	2.3989400	3.567718	Up
SLC4A4	-0.88293	6.530375	-4.93825	4.8462486	2.4110453	3.562402	Down
PYGO2	-0.61278	5.288119	-4.93788	4.8530185	2.4138511	3.561053	Down
MRS2	-0.60582	8.425974	-4.93668	4.8754658	2.4244516	3.556592	Down
SLC35E3	0.515722	7.85786	4.935656	4.8946672	2.4334335	3.552792	Up
EIF2AK2	0.576436	8.4393	4.934391	4.9184903	2.4447083	3.548099	Up
GALNS	0.505932	6.160595	4.93346	4.9360859	2.4528832	3.544647	Up
LRRC58	0.654135	7.883147	4.930868	4.9854186	2.4762458	3.535035	Up
LRFN3	-0.64265	5.012437	-4.93078	4.9871267	2.4765183	3.534704	Down
INO80C	-0.5396	8.239136	-4.93023	4.9976796	2.4811818	3.532661	Down
CR1	1.122195	4.468851	4.929452	5.0157029	2.4889726	3.529263	Up
SNRNP35	-0.71225	8.049109	-4.92857	5.0296365	2.4953072	3.5265	Down
ELAPOR1	-0.96308	6.159081	-4.92763	5.0477148	2.5036947	3.523032	Down
SLC36A1	0.995747	6.24044	4.927293	5.0542663	2.5063622	3.521779	Up
CCDC88A	0.776646	7.909241	4.926879	5.0622872	2.5097570	3.520246	Up
ADIRF	1.312391	9.928702	4.92975	5.0656563	2.5108444	3.521134	Up
PRXL2C	0.623675	7.425478	4.925621	5.0867744	2.5201421	3.515582	Up
FIBIN	1.149171	7.601312	4.927235	5.0948904	2.5235776	3.515063	Up
IDH2	-0.67832	11.95087	-4.92582	5.0968964	2.5239859	3.514022	Down
LOC10537	0.726588	8.478026	4.924902	5.1008071	2.5253370	3.512919	Up
PARP14	0.756949	7.15179	4.924422	5.1102166	2.5294091	3.511138	Up
NABP1	1.484943	6.083034	4.926741	5.1180692	2.5321223	3.511023	Up
CCDC85B	-0.5559	7.758706	-4.92364	5.1255158	2.5352192	3.508249	Down
IDI1	-0.69814	10.06654	-4.92211	5.1556447	2.5495313	3.502585	Down
ETFBKMT	-0.64868	5.226333	-4.92164	5.1650636	2.5535979	3.500821	Down
IGSF6	1.450216	3.732374	4.921352	5.2027207	2.5716201	3.494612	Up
ISCA2	-0.59544	8.748019	-4.91842	5.2292254	2.5841230	3.488889	Down
PERP	1.303135	7.781623	4.920253	5.2467347	2.5915763	3.487025	Up
ARG2	1.343839	7.110172	4.917675	5.2987362	2.6150462	3.477493	Up
RPE	0.721476	6.946977	4.914949	5.2991479	2.6150462	3.476053	Up
PTCRA	-0.58621	6.233706	-4.91475	5.3031114	2.6163976	3.47533	Down
UBE2C	1.271158	4.106956	4.915188	5.3162392	2.6222687	3.473486	Up
ADAMTSL	0.899106	6.929608	4.913271	5.3333145	2.6288698	3.469842	Up
EIF2B3	-0.51783	9.056959	-4.91096	5.3807349	2.6510204	3.461288	Down
ZNF727	-2.12108	3.517992	-4.9131	5.3989176	2.6587522	3.459551	Down
TRIM28	-0.5461	8.769645	-4.90853	5.4309496	2.6732939	3.452312	Down
HMGCS2	3.956912	6.156552	4.91615	5.4343089	2.6743311	3.45564	Up
PTRH1	-0.55168	5.883718	-4.90744	5.4537387	2.6832746	3.448266	Down
HK1	-0.6184	9.430499	-4.90721	5.4585230	2.6850100	3.447419	Down
CFL1	0.530526	11.47032	4.906863	5.4657923	2.6879666	3.446133	Up
BEND5	-0.72526	5.752309	-4.90672	5.4686928	2.6887740	3.44562	Down
BCO2	-1.18976	6.95741	-4.90729	5.4903752	2.6988132	3.442605	Down
HMCN1	1.187775	6.97203	4.905525	5.5275291	2.7152016	3.436087	Up
FOXN4	-0.57607	4.822198	-4.90344	5.5379404	2.7196903	3.433462	Down
TMEM52	-1.09977	9.814963	-4.90578	5.5451743	2.7226168	3.43356	Down
PRRG3	1.271748	5.483686	4.899503	5.6562564	2.7758806	3.413843	Up
FGF7P6	0.99325	6.664749	4.895309	5.7248143	2.8082357	3.401674	Up
PYGO1	-1.01517	6.961713	-4.89479	5.7470400	2.8184909	3.398181	Down
ROCK1	0.764204	8.766902	4.892558	5.7732624	2.8300513	3.393255	Up
CAV2	0.769021	9.516407	4.892179	5.7816212	2.8334985	3.391857	Up

IL3	-0.97221	3.947862	-4.89183	5.7893738	2.8366471	3.390563	Down
DSPP	-0.54223	4.415708	-4.89158	5.7947747	2.8386422	3.389662	Down
KLHDC3	-0.86686	10.2099	-4.89139	5.8278297	2.8541799	3.384824	Down
AIF1L	0.775497	7.961339	4.889477	5.8416568	2.8602958	3.381878	Up
NPR3	-2.28682	5.799077	-4.89359	5.8617249	2.8692889	3.381109	Down
PREP	-0.76867	8.219336	-4.88824	5.8693024	2.8718570	3.377317	Down
SPATA8	-0.63148	3.910789	-4.88791	5.8766929	2.8748146	3.376101	Down
P3H2	1.469636	4.760748	4.889401	5.8979097	2.8845329	3.373855	Up
WIPF3	-0.94237	9.157899	-4.88831	5.9032020	2.8864603	3.372551	Down
SLC52A3	-0.85018	5.322276	-4.8863	5.9129102	2.8905456	3.370166	Down
ASPA	0.713465	7.851847	4.885942	5.9211164	2.8938948	3.368826	Up
EHBP1	0.57029	7.986261	4.88588	5.9225000	2.8939088	3.3686	Up
PIBF1	0.665675	6.892967	4.884977	5.9429771	2.9032503	3.365266	Up
AKR7A3	-0.5956	6.372308	-4.88421	5.9603274	2.9105990	3.36245	Down
SH3BP5	0.984361	9.497551	4.885716	5.9620881	2.9105990	3.362967	Up
TPD52L3	-0.81599	4.837098	-4.88414	5.9621081	2.9105990	3.362161	Down
COL7A1	-0.5802	5.433305	-4.88397	5.9657755	2.9117238	3.361567	Down
MATCAP2	1.314247	4.497146	4.884365	5.9926606	2.9241774	3.358021	Up
DSTN	0.610527	11.16641	4.88034	6.0491794	2.9504081	3.348156	Up
EDEM1	0.526953	6.745145	4.879217	6.0751730	2.9610576	3.344015	Up
TASOR2	0.520751	7.876927	4.878715	6.0868338	2.9660642	3.342163	Up
TRAPPC2	0.519297	6.862689	4.87847	6.0925424	2.9681688	3.341257	Up
IL18R1	1.167616	3.104151	4.877767	6.1089118	2.9754650	3.338665	Up
CYP4X1	-1.18786	5.83693	-4.87861	6.1192282	2.9798102	3.33769	Down
NUDT17	-0.60107	6.044296	-4.87676	6.1324688	2.9855771	3.334948	Down
C6orf62	0.573489	9.534446	4.875994	6.1504144	2.9936315	3.332125	Up
F3	1.11883	5.985813	4.876494	6.1628364	2.9983954	3.330701	Up
ARHGAP2	0.8773	6.967742	4.875458	6.1630097	2.9983954	3.330149	Up
ZNF117	0.794836	5.293476	4.8747	6.1808629	3.0057119	3.327356	Up
SERPINH1	1.205735	7.707475	4.877013	6.1887050	3.0088404	3.327478	Up
SNX10	1.441949	5.776664	4.874278	6.2535266	3.0396636	3.317413	Up
COX19	-0.79041	5.418785	-4.86761	6.3502844	3.0845889	3.301238	Down
SCUBE1	-0.97309	3.648764	-4.86726	6.3588976	3.0880704	3.299929	Down
NEXN	-0.53697	12.06358	-4.86719	6.3604703	3.0881319	3.29969	Down
TTC39C	1.343588	3.832234	4.867522	6.3835406	3.0986286	3.296846	Up
C7	1.996131	6.275255	4.870891	6.3909300	3.1015106	3.297602	Up
HOXC8	-0.75938	4.774018	-4.86339	6.4534095	3.1304092	3.285681	Down
CALCOCC	-0.65659	8.849458	-4.86274	6.4694402	3.1374728	3.283285	Down
FLNC	-0.73333	11.46151	-4.86361	6.4748548	3.1393859	3.283032	Down
PPP1R3B	-0.83128	9.577732	-4.86263	6.4908398	3.1464221	3.280481	Down
TERT	-0.69842	4.368206	-4.86157	6.4983505	3.1493480	3.278979	Down
LAGE3	-0.51621	7.122246	-4.86093	6.5141346	3.1562814	3.276636	Down
GLI4	-0.64573	4.073703	-4.86024	6.5314225	3.1639402	3.274077	Down
EEF2K	-0.62022	9.254939	-4.85772	6.5944930	3.1915971	3.264797	Down
IKZF2	-0.7848	6.315336	-4.85658	6.6231772	3.2047534	3.260605	Down
ZNF513	-0.72995	4.86093	-4.85616	6.6337112	3.2091235	3.259071	Down
HES1	-0.87801	6.791652	-4.856	6.6378466	3.2103969	3.258469	Down
CCDC127	-0.50213	7.93775	-4.85557	6.6486863	3.2149115	3.256893	Down
KCNK6	0.835495	4.131029	4.85544	6.6519658	3.2157692	3.256417	Up
MTHFS	-0.57573	7.281746	-4.85162	6.7494965	3.2599671	3.242362	Down
AHSA1	-0.52809	8.913422	-4.84882	6.8218278	3.2919249	3.23207	Down
EIF5A2	0.999857	4.702496	4.84809	6.8408244	3.3003462	3.229385	Up
FSD2	-0.70615	9.253891	-4.84511	6.9188625	3.3357351	3.218433	Down
BCL6B	-0.67689	6.532714	-4.84498	6.9224208	3.3366974	3.217937	Down
PCDHB15	1.107421	4.152189	4.844636	6.9313870	3.3391710	3.216687	Up
TAF8	-0.61547	5.781972	-4.84403	6.9475076	3.3457693	3.214444	Down
LOC73009	-0.93101	5.214297	-4.84385	6.9522646	3.3473054	3.213783	Down
RIC3	-0.75621	5.377904	-4.8433	6.9667901	3.3527873	3.211768	Down

ITPRIPL2	0.578687	9.200849	4.84247	6.9887694	3.3626073	3.208727	Up
NHLH2	1.982066	3.933913	4.844279	7.0179109	3.3758680	3.2062	Up
TMEM168	0.581979	7.633804	4.841242	7.0215066	3.3768371	3.204215	Up
RAB9B	0.73391	8.250035	4.840682	7.0364707	3.3825104	3.20216	Up
CLDN5	0.60155	8.001806	4.840368	7.0448963	3.3857987	3.201005	Up
UBE2G1	-0.51164	11.10728	-4.84031	7.0464959	3.3858056	3.200786	Down
ESPN	-0.70426	4.519986	-4.84019	7.0497379	3.3866015	3.200341	Down
XK	-0.84993	6.135144	-4.83983	7.0593516	3.3904571	3.199026	Down
REEP3	0.616092	7.858384	4.839552	7.0667999	3.3932714	3.198008	Up
CDC7	1.30524	3.996334	4.83906	7.1139920	3.4143964	3.192229	Up
IRF9	0.92502	7.452532	4.838421	7.1157239	3.4144604	3.191698	Up
SIAH2	-0.62198	6.619891	-4.83638	7.1524640	3.4305485	3.186376	Down
GNAL	-1.20269	7.416601	-4.83845	7.1666044	3.4365590	3.1858	Down
UTRN	0.637186	9.123228	4.835115	7.1871003	3.4456136	3.181712	Up
CD99L2	-0.69863	8.167593	-4.83479	7.1958779	3.4490475	3.180534	Down
ATP7A	0.673259	6.120349	4.833466	7.2323103	3.4657322	3.175658	Up
ALG10B	0.794632	6.251853	4.833265	7.2378466	3.4676072	3.17492	Up
DHPS	-0.71132	8.246345	-4.83214	7.2689461	3.4817257	3.170781	Down
PPP1R37	-0.74464	3.819034	-4.83126	7.2932106	3.4925648	3.167564	Down
KLC3	-0.7554	4.717643	-4.8302	7.3227030	3.5027612	3.163668	Down
MMP24	-0.75452	4.019783	-4.82965	7.3380575	3.5093200	3.161646	Down
NKX2-5	-0.80685	4.497876	-4.82735	7.4024472	3.5393209	3.153213	Down
PMM2	0.603726	5.948364	4.826939	7.4140204	3.5435861	3.151705	Up
TMEM59L	-0.70081	4.647245	-4.82634	7.4307845	3.5493059	3.149525	Down
RGMB	0.509006	8.775059	4.826314	7.4316398	3.5493059	3.149414	Up
CACTIN	-0.59801	6.267071	-4.82522	7.4625613	3.5625455	3.145406	Down
CHD2	-0.55805	8.569551	-4.82522	7.4626971	3.5625455	3.145389	Down
ARMT1	0.525268	8.548385	4.822697	7.5344916	3.5944085	3.136147	Up
APIP	-0.63414	8.740418	-4.82173	7.5621904	3.6068169	3.132606	Down
ZNF124	1.110805	5.227458	4.820498	7.6128365	3.6288037	3.126434	Up
TNFRSF4	-0.70879	4.45076	-4.81995	7.6133858	3.6288037	3.126093	Down
DAP	1.019911	6.255534	4.819121	7.6527416	3.6467482	3.121388	Up
FHL5	-1.19451	5.763762	-4.81925	7.6698112	3.6532521	3.119609	Down
TRIL	0.936509	6.854927	4.817509	7.6927609	3.6625498	3.116231	Up
LYAR	-0.88788	7.006972	-4.81345	7.8037733	3.7129202	3.102255	Down
NUSAP1	0.624594	7.12736	4.812166	7.8418697	3.7302150	3.097556	Up
SHISA5	0.735682	7.920416	4.810566	7.8896472	3.7512708	3.091694	Up
PPP1R3A	-0.90072	10.25012	-4.81197	7.8929506	3.7520062	3.092074	Down
CTU2	-0.59061	6.127299	-4.80955	7.9200293	3.7622346	3.087985	Down
NATD1	0.532429	9.289956	4.80916	7.9318588	3.7663103	3.086544	Up
NEK2-DT	-0.55166	8.061714	-4.8084	7.9547312	3.7763314	3.083766	Down
SSNA1	-0.51347	6.720458	-4.8079	7.9698893	3.7826865	3.081929	Down
GALNT17	1.455116	4.938506	4.80821	8.0345485	3.8108347	3.075396	Up
GPR176	0.902743	5.073576	4.805574	8.0404798	3.8128012	3.073419	Up
RAP1GDS1	0.938542	6.598288	4.805031	8.0598865	3.8211555	3.071141	Up
PIKFYVE	0.593327	8.390827	4.803994	8.0888215	3.8340224	3.067635	Up
TMEM220	-0.57034	6.683455	-4.80285	8.1240321	3.8490035	3.063444	Down
UBR5-DT	0.734995	5.828932	4.802702	8.1285577	3.8502935	3.062906	Up
SRSF5	0.541343	10.10547	4.802073	8.1479546	3.8586256	3.060606	Up
IQCC	-0.75632	4.157659	-4.80058	8.1941792	3.8787961	3.055148	Down
VPS11	-0.51292	6.994723	-4.79663	8.3179721	3.9356502	3.04068	Down
ZNF554	-0.53837	5.533734	-4.79578	8.3447310	3.9465625	3.037581	Down
ZC2HC1C	-0.73289	4.193902	-4.7954	8.3565618	3.9504083	3.036214	Down
THBS4	1.408774	10.89317	4.799322	8.3830560	3.9615193	3.035628	Up
TAF6	-0.63503	7.35056	-4.79455	8.3837753	3.9615193	3.033077	Down
ACAT1	-0.6561	11.81671	-4.79382	8.4236973	3.9795029	3.028769	Down
LOC10192	1.169261	3.325796	4.79159	8.4781815	4.0034711	3.022273	Up
USP28	-0.62744	8.342058	-4.79062	8.5093106	4.0155072	3.018738	Down

LARP6	-0.64896	7.652434	-4.78916	8.5567327	4.0361019	3.013376	Down
FGFR1	0.864649	8.853772	4.788911	8.5934707	4.0525358	3.009705	Up
HNRNPR	0.511786	10.35848	4.784649	8.7039800	4.1037440	2.996916	Up
GIGYF1	-0.57703	4.79684	-4.78417	8.7196262	4.1102135	2.995183	Down
DMRT3	1.432101	4.028385	4.785447	8.7268823	4.1123885	2.995158	Up
GPR6	0.728113	4.54059	4.783918	8.7280912	4.1123885	2.994247	Up
TINAGL1	-0.77349	6.136365	-4.7837	8.7353094	4.1142373	2.99345	Down
MNS1	0.780311	5.392552	4.783683	8.7358678	4.1142373	2.993388	Up
EXOC3	-0.52959	7.709945	-4.7825	8.7750147	4.1317629	2.989075	Down
LSMEM2	-0.91612	5.229953	-4.78184	8.7969011	4.1411552	2.986672	Down
CIT	0.980372	4.419701	4.780126	8.8542616	4.1663212	2.980402	Up
MPV17L	-0.65417	6.591825	-4.77928	8.8824923	4.1768443	2.977331	Down
RNF151	-0.88969	4.5668	-4.77737	8.9472051	4.2054226	2.970329	Down
SWSAP1	-0.53655	4.920386	-4.77714	8.9548438	4.2080200	2.969506	Down
GADL1	1.20886	5.169487	4.778301	8.9566715	4.2080200	2.969943	Up
USP32P2	0.77039	8.749797	4.776756	8.9678618	4.2123508	2.968105	Up
PNKP	-0.53488	5.9527	-4.77552	9.0098246	4.2302008	2.963602	Down
PLEKHF1	-1.02158	7.664179	-4.77644	9.0290327	4.2382875	2.962322	Down
SMIM8	-0.67298	6.844638	-4.77352	9.0783798	4.2605149	2.95629	Down
ATG10	-0.64075	6.072697	-4.77226	9.1217896	4.2784058	2.951689	Down
RHOA	0.588601	11.6965	4.772236	9.1225116	4.2784058	2.951613	Up
PRICKLE1	0.828752	7.935404	4.771021	9.1708792	4.2992019	2.946609	Up
THBS1	2.55507	7.294738	4.774761	9.2157390	4.3192836	2.94453	Up
GNB5	0.562976	5.599659	4.769108	9.2310275	4.3254999	2.940207	Up
ARFGAP3	0.505768	8.195597	4.766723	9.3146049	4.3617920	2.931514	Up
SRSF11	0.527372	10.49639	4.765829	9.3461180	4.3747352	2.928257	Up
FENDRR	-0.63287	4.66136	-4.76576	9.3483901	4.3747352	2.928023	Down
MMP19	1.051896	5.403155	4.764105	9.4153935	4.4051254	2.921255	Up
SAPCD1	0.759939	3.947691	4.763436	9.4310235	4.4105058	2.919535	Up
CBR3	0.788018	6.199827	4.763059	9.4444485	4.4158172	2.918163	Up
PIWIL4	0.89355	3.822139	4.762057	9.4802541	4.4315882	2.914514	Up
MAPK13	0.747864	4.716353	4.761238	9.5096493	4.4443564	2.911528	Up
UPB1	-1.06827	4.137221	-4.76027	9.5444170	4.4596294	2.908009	Down
GRIN2D	1.001909	3.759731	4.759752	9.5631732	4.4674158	2.906116	Up
C8orf88	1.370294	5.888614	4.760034	9.6418503	4.5022002	2.8995	Up
SCARNA1	-0.83743	4.95679	-4.75715	9.6575766	4.5075726	2.896643	Down
MMAB	-0.64208	7.305734	-4.75409	9.7696333	4.5568863	2.885518	Down
MAFIP	-1.11532	6.01698	-4.75313	9.8458948	4.5914546	2.878594	Down
LOC1019	-0.87743	3.502291	-4.75181	9.8543106	4.5933733	2.877197	Down
FAM83G	-0.93285	4.230688	-4.75171	9.8578101	4.5940019	2.876854	Down
GALNT14	0.793197	3.826545	4.751412	9.8690441	4.5982339	2.875756	Up
AKIRIN2	-0.59996	9.064014	-4.75019	9.9148299	4.6185591	2.871293	Down
COL26A1	-1.02589	3.681756	-4.74954	9.9388405	4.6287343	2.868961	Down
BRF2	-0.56404	7.712124	-4.74893	9.9617944	4.6384130	2.866737	Down
SEPTIN7P	-0.65959	6.676277	-4.74786	1.0002356	4.6552698	2.862818	Down
RHOBTB3	0.649652	9.347502	4.747084	1.0031538	4.6668176	2.86001	Up
RIPOR3	-1.02821	5.691982	-4.74646	1.0064563	4.6806134	2.856968	Down
MAGIX	-0.62636	5.251281	-4.74619	1.0065576	4.6806134	2.856744	Down
DOCK7	0.632173	6.782821	4.744354	1.0135335	4.7099760	2.850084	Up
MMP25	-0.52149	4.441965	-4.74351	1.0167541	4.7228871	2.847026	Down
EZR	0.832101	7.980779	4.743582	1.0175089	4.7253656	2.84645	Up
CCDC14	0.907202	7.206864	4.742177	1.0238201	4.7526084	2.840612	Up
TACSTD2	1.006535	3.958606	4.741427	1.0247828	4.7560433	2.839442	Up
GMDS	0.911687	6.010541	4.741223	1.0255713	4.7586689	2.838701	Up
MECOM	0.540076	7.171703	4.741045	1.0262609	4.7608344	2.838053	Up
AFG2A	0.612297	4.604634	4.740494	1.0283928	4.7686527	2.836052	Up
HAMP	-0.68059	5.006244	-4.74035	1.0289333	4.7701232	2.835546	Down
STAC2	-0.53141	4.925516	-4.74	1.0303129	4.7754826	2.834254	Down

S1PR2	-0.58024	5.585636	-4.73978	1.0311667	4.7784029	2.833455	Down
CHTF18	-0.51685	5.273566	-4.73967	1.0315883	4.7793198	2.833061	Down
GRSF1	-0.53143	10.38267	-4.73905	1.0340034	4.7890553	2.830807	Down
VSIG10	-0.88324	6.900218	-4.73902	1.0341381	4.7890553	2.830681	Down
MAP3K4	-0.51121	8.172547	-4.73855	1.0359552	4.7964299	2.828989	Down
NAP1L2	-1.28786	3.898168	-4.73953	1.0367547	4.7990913	2.828871	Down
PCBD1	-0.54472	8.00551	-4.73818	1.0374132	4.8010990	2.827633	Down
ISG15	1.20137	7.45835	4.739015	1.0437148	4.8281702	2.823083	Up
LOC10192	-1.15207	4.407801	-4.73679	1.0453811	4.8346346	2.820596	Down
LOC10536	0.954373	7.443285	4.737115	1.0455650	4.8346346	2.820622	Up
FSIP1	-0.6819	4.216121	-4.73581	1.0466950	4.8388122	2.819046	Down
S100A13	0.81164	10.43532	4.734087	1.0582346	4.8858140	2.8091	Up
TUBA4A	-0.7544	10.70063	-4.73078	1.0703630	4.9396746	2.797969	Down
GABPA	0.573807	7.215894	4.728461	1.0760885	4.9650246	2.792348	Up
MTHFD2L	0.712364	7.317355	4.728297	1.0767540	4.9670220	2.791752	Up
TET1	0.951044	6.88367	4.728363	1.0790323	4.9753826	2.790046	Up
LIMS3	1.321946	5.513696	4.727847	1.0844531	4.9992985	2.785647	Up
ZNF582	0.649168	4.543362	4.726119	1.0856191	5.0035938	2.783848	Up
CDK1	1.185986	4.598824	4.725017	1.0940128	5.0411921	2.776924	Up
MIDN	-1.02842	6.526729	-4.72304	1.1021928	5.0766952	2.769743	Down
HIVEP3	-0.6219	5.525796	-4.72186	1.1031637	5.0789762	2.768396	Down
SLC17A2	1.353755	3.971677	4.72218	1.1078106	5.0992714	2.765105	Up
SLC16A4	1.195375	5.419677	4.720691	1.1129334	5.1217481	2.76052	Up
GTF2IRD1	-0.73177	8.544401	-4.71908	1.1147563	5.1290317	2.758321	Down
CMTR2	0.718469	7.162814	4.717263	1.1224031	5.1631026	2.751732	Up
HTR4	-0.69642	5.611652	-4.71584	1.1284422	5.1897652	2.74656	Down
INSR	-0.70322	7.768886	-4.71546	1.1300282	5.1959406	2.745207	Down
MRPL28	-0.58448	6.932856	-4.71371	1.1374870	5.2291106	2.738866	Down
STAB1	0.862363	6.998634	4.713509	1.1383606	5.2309301	2.738126	Up
ARHGDIA	-0.59846	7.531148	-4.71351	1.1383726	5.2309301	2.738116	Down
TPD52	0.840523	7.963731	4.712585	1.1440518	5.2558956	2.733534	Up
TSC22D1-	-0.69457	4.482924	-4.70932	1.1564403	5.3093838	2.722941	Down
ZNF71	-0.81794	5.057324	-4.70857	1.1597108	5.3221204	2.72022	Down
LHPP	-0.54193	4.768805	-4.70857	1.1597129	5.3221204	2.720218	Down
SLC37A4	-1.05489	7.050065	-4.70952	1.1617557	5.3303498	2.71928	Down
SAMD4B	0.577651	7.089612	4.708033	1.1620342	5.3304828	2.718291	Up
ATR	0.600893	7.166228	4.704877	1.1758968	5.3905997	2.706863	Up
POLM	-0.59808	4.388843	-4.70361	1.1815092	5.4151664	2.702275	Down
AK4	0.885768	6.323821	4.702811	1.1850566	5.4290947	2.699386	Up
HSPA9	-0.60985	10.24931	-4.70031	1.1962266	5.4767434	2.690347	Down
MAML3	0.724801	5.47683	4.698601	1.2039396	5.5108748	2.684154	Up
DECR2	-0.51493	7.504617	-4.69752	1.2088369	5.5321063	2.680243	Down
LINC0134:	-0.52034	5.231264	-4.69725	1.2100785	5.5366018	2.679254	Down
ZNF205	-0.75255	5.000181	-4.69691	1.2115985	5.5411825	2.678044	Down
ENTPD1	0.67909	6.503924	4.693869	1.2255143	5.5976339	2.667041	Up
FCHO2	0.510138	8.647949	4.692126	1.2335582	5.6319660	2.660738	Up
BAZ2A	-0.50885	7.328439	-4.69147	1.2365748	5.6445319	2.658385	Down
HEPH	1.104436	4.730723	4.691198	1.2401239	5.6595230	2.655883	Up
VWA5B2	-0.79825	3.604591	-4.69043	1.2414387	5.6643130	2.654603	Down
ZNF503-A	-0.93689	4.779629	-4.68878	1.2491570	5.6946629	2.648632	Down
PTN	2.101181	6.476723	4.692337	1.2534204	5.7128794	2.647732	Up
ACVR2B	-0.76562	7.230931	-4.68482	1.2678180	5.7735730	2.634347	Down
HECTD2	0.892427	6.315847	4.682396	1.2794005	5.8225945	2.625586	Up
NUP98	-0.76812	7.363269	-4.67885	1.2965104	5.8966931	2.61279	Down
RNF122	-0.50588	5.912198	-4.67873	1.2970897	5.8980717	2.612359	Down
SMURF1	-0.66759	9.240734	-4.67839	1.2987502	5.9043653	2.611127	Down
CDKN2C	0.601256	7.869328	4.678328	1.2990560	5.9044985	2.6109	Up
DPH3	-0.52688	8.771586	-4.67748	1.3031718	5.9219455	2.607853	Down

CHRNA2	-0.50977	4.44117	-4.67634	1.3087754	5.9461445	2.603721	Down
FABP3	-1.26108	10.10388	-4.67816	1.3143259	5.9675538	2.601224	Down
ZNF532	-0.53447	8.793743	-4.67207	1.3298769	6.0355954	2.588315	Down
COPG2IT1	0.905358	5.270357	4.670993	1.3352397	6.0586467	2.584439	Up
TMOD4	-0.54638	12.20042	-4.6709	1.3357008	6.0594515	2.584107	Down
PNMA1	0.586242	8.302642	4.670049	1.3399679	6.0775186	2.581035	Up
TANGO6	-0.54441	5.196618	-4.66833	1.3485955	6.1140531	2.574853	Down
LIG3	-0.53376	6.624937	-4.66794	1.3505650	6.1216827	2.573448	Down
IFI27	0.800306	8.886478	4.667828	1.3535559	6.1339376	2.571572	Up
KRT79	-0.53491	4.141999	-4.66724	1.3541223	6.1352027	2.570915	Down
CHIC2	0.599632	7.418282	4.666609	1.3573306	6.1458276	2.568635	Up
RRP7A	-0.60815	6.298512	-4.66566	1.3621683	6.1650265	2.565209	Down
EZH2	1.457435	4.230065	4.667572	1.3641766	6.1709579	2.565028	Up
MIGA2	-0.54215	6.506163	-4.66524	1.3643254	6.1709579	2.563685	Down
MIR100HC	0.887436	7.630003	4.665023	1.3688559	6.1901388	2.560854	Up
CABP1	-0.90118	3.755635	-4.66413	1.3699778	6.1939007	2.559704	Down
ATP1A1	0.793722	8.547036	4.664222	1.3707319	6.1959985	2.559302	Up
ANKRD36	0.992434	5.20403	4.662944	1.3760738	6.2188290	2.555428	Up
CXCR2	-1.6959	3.477979	-4.66457	1.3797572	6.2341560	2.55411	Down
CEP76	0.553832	5.604008	4.6619	1.3814598	6.2402792	2.551666	Up
AMHR2	-0.67976	3.724402	-4.66185	1.3816968	6.2402792	2.551501	Down
MGLL	0.550684	10.32222	4.661755	1.3822098	6.2412764	2.551144	Up
TRAF2	-0.86269	3.663697	-4.66164	1.3828230	6.2427256	2.550716	Down
IFIT3	1.132498	8.031757	4.663564	1.3849558	6.2510329	2.550488	Up
PPP2R2C	-0.74953	3.638842	-4.65808	1.4013113	6.3208463	2.537927	Down
H2AC18	0.592644	8.567564	4.657989	1.4018122	6.3217705	2.537583	Up
FAM86B3I	-0.70084	6.544353	-4.65703	1.4068581	6.3418481	2.534123	Down
NQO2	-0.9929	8.766312	-4.65805	1.4087615	6.3477487	2.533566	Down
HERC4	0.515101	7.48666	4.656026	1.4121395	6.3616274	2.530515	Up
SLC2A14	0.819784	4.375596	4.65585	1.4130687	6.3644713	2.529881	Up
LPP	0.676567	9.704502	4.653069	1.4278347	6.4296212	2.519872	Up
AK6	-0.5322	9.047741	-4.65249	1.4309450	6.4409112	2.517777	Down
SCN7A	1.178956	8.258842	4.654751	1.4312844	6.4410814	2.518801	Up
USP32	0.538078	9.091931	4.650094	1.4437981	6.4960271	2.509167	Up
ZNF335	-0.56358	5.690053	-4.64894	1.4500111	6.5226069	2.505033	Down
C4A	1.234541	6.362762	4.650248	1.4530083	6.5319619	2.504044	Up
SPRYD4	-0.62463	6.610943	-4.64793	1.4554938	6.5403823	2.501399	Down
MX1	1.064503	7.347556	4.64887	1.4578986	6.5498104	2.500552	Up
PAQR3	0.646094	6.571179	4.647342	1.4587168	6.5507303	2.499269	Up
CRACR2A	0.754945	5.510106	4.647105	1.4600074	6.5551478	2.498418	Up
MXRA7	0.596932	10.84864	4.646728	1.4620666	6.5616344	2.497061	Up
RAB27B	0.721634	4.129404	4.644358	1.4750635	6.6144041	2.48854	Up
CCDC102I	1.877872	3.973517	4.646295	1.4786578	6.6274348	2.487593	Up
HRAS	-0.54468	8.030995	-4.64366	1.4789003	6.6274348	2.486039	Down
DNAJB1	-0.54678	8.379642	-4.64223	1.4868587	6.6617010	2.480873	Down
PDGFB	-0.55057	6.653063	-4.64078	1.4949242	6.6964328	2.475665	Down
CALCRL	0.823298	6.204761	4.640444	1.4967769	6.7033260	2.474472	Up
ZFYVE16	0.574881	8.089048	4.640312	1.4975135	6.7052189	2.473999	Up
RCAN3	0.846838	6.493504	4.640114	1.4986199	6.7087665	2.473288	Up
NFIX	-0.65019	9.998819	-4.63866	1.5067682	6.7438299	2.468068	Down
LOC1005C	0.51557	5.605633	4.638298	1.5088126	6.7515651	2.466762	Up
PCNX4	0.730862	6.940052	4.637922	1.5109326	6.7596353	2.465411	Up
CCNL1	0.6	8.248823	4.63636	1.5197632	6.7948721	2.459801	Up
IFT57	0.531654	6.942486	4.63591	1.5223158	6.8048603	2.458186	Up
IRX2	-0.68015	3.851307	-4.63306	1.5385960	6.8726429	2.447946	Down
PTGDS	1.070425	10.44159	4.63533	1.5387666	6.8726429	2.449083	Up
LYRM7	-0.53586	9.901201	-4.63259	1.5412944	6.8824936	2.446259	Down
COL6A2	0.901805	10.11375	4.633223	1.5455038	6.8998478	2.444372	Up

HAGLR	0.750657	4.976843	4.631686	1.5464961	6.9016533	2.443016	Up
LUC7L	0.50795	7.736824	4.631065	1.5500798	6.9159400	2.440788	Up
FAM114A:	0.502045	8.772378	4.630168	1.5552739	6.9376650	2.437568	Up
TBC1D32	1.501182	3.548268	4.629318	1.5681499	6.9921807	2.43037	Up
VCX2	-0.97168	4.642883	-4.62757	1.5704071	7.0007837	2.428248	Down
ACAP1	-1.07013	5.284697	-4.62709	1.5760487	7.0244677	2.425058	Down
NAPRT	-0.98339	6.217725	-4.62692	1.5769552	7.0270416	2.424497	Down
ADGRB1	-0.61146	4.816208	-4.62433	1.5894641	7.0798278	2.416639	Down
SLC25A37	-0.75504	8.08349	-4.62258	1.5998655	7.1202186	2.410361	Down
CENPF	0.661768	4.943622	4.622411	1.6008981	7.1233296	2.40974	Up
AKAP11	0.570883	8.604302	4.618785	1.6226649	7.2126698	2.396743	Up
LOC10012	-0.55075	4.796209	-4.61823	1.6260074	7.2260232	2.394763	Down
PRPF38B	0.752084	8.420021	4.617158	1.6325324	7.2520025	2.390909	Up
LOC43993	-0.9976	3.896578	-4.61188	1.6648980	7.3896287	2.372017	Down
ID2	1.080659	8.134648	4.613991	1.6657944	7.3920710	2.372723	Up
ADRA1D	-0.53906	3.795377	-4.61162	1.6665663	7.3939602	2.371053	Down
STARD7-A	-0.76747	4.904754	-4.61154	1.6670379	7.3945164	2.370781	Down
MTMR3	-0.53925	7.824312	-4.61112	1.6696592	7.4046061	2.369269	Down
FXYD5	0.808164	7.050364	4.610153	1.6756612	7.4277754	2.365816	Up
TRPV6	-0.97146	6.177184	-4.61045	1.6759270	7.4277754	2.365847	Down
BCAS3	-0.60631	5.567795	-4.60974	1.6782243	7.4364138	2.364346	Down
MFN2	-0.955	9.879007	-4.60949	1.6920475	7.4945567	2.357513	Down
SLC37A3	0.86858	6.228431	4.607176	1.6943254	7.5015345	2.355158	Up
CROCCP2	0.690619	8.315127	4.60708	1.6949270	7.5026430	2.354817	Up
ATP2B2	-1.4147	7.141281	-4.60818	1.7033211	7.5382372	2.351387	Down
LACTB2	-0.69688	8.518624	-4.60543	1.7053549	7.5444978	2.348915	Down
HSPB3	0.505537	11.53904	4.605417	1.7054422	7.5444978	2.348866	Up
PRO2268	-0.5163	3.889562	-4.60506	1.7076953	7.5529006	2.347595	Down
CNOT9	-0.55898	6.716711	-4.60472	1.7098808	7.5594361	2.346365	Down
NAT1	0.656797	5.234373	4.603779	1.7158684	7.5843373	2.343002	Up
SQOR	-0.52611	9.590535	-4.60368	1.7165009	7.5855632	2.342647	Down
PLPP5	1.016793	5.755094	4.603845	1.7182396	7.5916763	2.341911	Up
CFAP73	-1.62559	5.519928	-4.6056	1.7186706	7.5920098	2.342663	Down
EXOC6B	-0.53741	6.33998	-4.60313	1.7200102	7.5963564	2.340682	Down
FAM107B	1.009465	7.175689	4.603433	1.7266653	7.6241715	2.337697	Up
GLI1	-0.86097	4.475938	-4.60157	1.7300184	7.6373985	2.3351	Down
DNASE1L1	-0.60169	9.399472	-4.60103	1.7334961	7.6495891	2.333168	Down
PABPC5	0.943193	3.363574	4.599103	1.7459541	7.7016563	2.326278	Up
APOL6	0.751111	6.657332	4.599094	1.7460164	7.7016563	2.326244	Up
SH3GL1	0.575873	6.718271	4.597629	1.7555509	7.7405154	2.321005	Up
KCNMA1	-0.88126	7.801633	-4.5978	1.7600523	7.7587611	2.319011	Down
STAG3	-0.62477	4.66493	-4.59653	1.7627222	7.7689271	2.317083	Down
TAF15	0.861039	7.368316	4.596713	1.7643914	7.7746797	2.31641	Up
AKAP6	-0.67844	7.009919	-4.59211	1.7919275	7.8895048	2.301275	Down
CCDC71L	1.175922	6.032214	4.593045	1.7945683	7.8995032	2.300586	Up
GDE1	-0.67801	9.540614	-4.5906	1.8020183	7.9306630	2.295873	Down
VPS53	-0.53944	5.999737	-4.58973	1.8077987	7.9544634	2.292792	Down
HAVCR2	1.657274	4.62172	4.590572	1.8172398	7.9943583	2.289007	Up
OFD1	0.513659	7.345971	4.588093	1.8188439	7.9997669	2.286933	Up
ZPLD1	1.148767	2.75536	4.587842	1.8205401	8.0055786	2.286037	Up
MACROH:	-0.62875	5.867757	-4.58711	1.8255132	8.0257951	2.283412	Down
LRRC52	-0.69594	3.89349	-4.58505	1.8394957	8.0822771	2.276073	Down
CTRB2	-0.75526	4.516577	-4.58421	1.8452615	8.1053587	2.273063	Down
ARHGEF9	0.532732	6.060496	4.582711	1.8555474	8.1444269	2.267716	Up
SUGP1	-0.55323	5.987759	-4.58153	1.8636633	8.1783691	2.263519	Down
ATAD3A	-0.58385	5.570528	-4.58044	1.8712351	8.2082234	2.259619	Down
GSTM5	0.699382	6.624962	4.58014	1.8733331	8.2140524	2.258541	Up
MAN2A1	0.588854	8.131903	4.579345	1.8788613	8.2366010	2.255707	Up

H2BC5	-0.85465	7.440793	-4.57865	1.8868664	8.2699965	2.251865	Down
RAD51AP1	0.758196	4.653758	4.576612	1.8980067	8.3154109	2.245957	Up
RGS17	0.841076	3.830395	4.575797	1.9037503	8.3367349	2.243051	Up
DSCR4	-0.79243	3.511327	-4.57576	1.9040450	8.3367349	2.242902	Down
DUSP6	0.862422	8.344929	4.576419	1.9056316	8.3419720	2.242588	Up
TARS2	-0.60889	6.685063	-4.57541	1.9065169	8.3441370	2.241654	Down
NMRK2	-1.12729	9.660123	-4.57747	1.9076293	8.3472950	2.242312	Down
GTSF1L	-0.88142	6.434261	-4.57195	1.9310894	8.4482198	2.229338	Down
TAMALIN	-0.57718	6.347758	-4.57133	1.9355382	8.4659482	2.227126	Down
MYO5B	0.984975	5.210731	4.571005	1.9378703	8.4744135	2.225967	Up
KBTBD8	-0.94365	5.127942	-4.57035	1.9425843	8.4932887	2.223631	Down
NEU4	-0.64312	5.029584	-4.56914	1.9513234	8.5297517	2.219315	Down
MED28	-0.55544	8.057628	-4.5679	1.9602836	8.5671657	2.214909	Down
NFAM1	-0.78872	4.778678	-4.5674	1.9639270	8.5813326	2.213124	Down
LINC02091	-0.72057	3.597264	-4.5673	1.9646938	8.5829270	2.212748	Down
LOXL1	0.769297	7.550279	4.564694	1.9837143	8.6624760	2.203484	Up
SLC7A8	-0.57241	6.348633	-4.56396	1.9891142	8.6825059	2.20087	Down
LOC10272	0.893517	4.538214	4.563331	1.9937529	8.7009756	2.19863	Up
RASGRP3	-0.58816	7.696196	-4.56321	1.9946302	8.7030262	2.198207	Down
ACOX1	-0.78003	7.154258	-4.56247	2.0001035	8.7251247	2.195573	Down
USP6	0.830759	8.680984	4.563078	2.0013757	8.7288917	2.195389	Up
CEP120	0.576047	6.755083	4.562088	2.0029490	8.7339700	2.194206	Up
KIAA0753	0.514616	5.233531	4.560859	2.0120849	8.7720167	2.18983	Up
PGAM2	-0.81319	11.63793	-4.56255	2.0147701	8.7816224	2.189677	Down
CDC42EP3	0.722616	8.997877	4.560453	2.0151105	8.7816224	2.188385	Up
PPP1R21	0.507904	6.995048	4.560319	2.0161158	8.7842110	2.187906	Up
RP2	1.212056	5.376207	4.556576	2.0541267	8.9407051	2.170667	Up
RBM47	1.116954	5.388648	4.554113	2.0699141	9.0075849	2.163089	Up
ANXA2R	0.719878	5.342757	4.553146	2.0703435	9.0076181	2.162388	Up
PCDH1	-0.60191	5.157602	-4.55245	2.0756625	9.0270823	2.159921	Down
HLA-G	0.815517	9.9007	4.553124	2.0786780	9.0383563	2.159112	Up
ARHGAP1	0.582009	5.771889	4.551403	2.0837375	9.0585112	2.156188	Up
ARMCX1	0.601577	10.11195	4.547668	2.1127153	9.1807477	2.142912	Up
UFM1	0.560099	8.84036	4.546009	2.1257121	9.2334674	2.137016	Up
POLR3A	-0.50728	6.216582	-4.54496	2.1339928	9.2656678	2.133279	Down
PPM1A	-0.60608	9.997915	-4.54469	2.1360848	9.2728659	2.132337	Down
KDELR2	0.511375	9.537489	4.544249	2.1395822	9.2856614	2.130765	Up
ZSWIM3	-0.714	4.526069	-4.54261	2.1525493	9.3348525	2.124957	Down
ZNF408	-0.50616	5.482916	-4.54104	2.1650981	9.3873664	2.119369	Down
ALPG	-0.73411	4.69541	-4.54091	2.1661325	9.3899449	2.11891	Down
IKBIP	0.845749	8.271976	4.541599	2.1670397	9.3919715	2.11895	Up
CNN1	-1.88863	5.244355	-4.54363	2.1727734	9.4130012	2.11793	Down
ITPR1	0.808357	6.531158	4.53866	2.1842213	9.4568415	2.110917	Up
WNT5B	1.193895	4.265013	4.539043	2.1896500	9.4770453	2.109115	Up
SENP7	0.790456	6.552176	4.537973	2.1897751	9.4770453	2.108476	Up
MTERF2	0.548667	7.693717	4.537637	2.1924912	9.4868775	2.107285	Up
SPAG8	-0.77617	4.275561	-4.53742	2.1942114	9.4923980	2.106531	Down
IGF2BP3	1.192161	4.076591	4.534246	2.2280017	9.6307753	2.092375	Up
DUSP28	-0.62943	8.945795	-4.53239	2.2354143	9.6608618	2.088651	Down
CAVIN4	0.565826	11.38029	4.531936	2.2391269	9.6729919	2.087056	Up
CD93	0.534803	9.648989	4.531289	2.2444845	9.6922161	2.084759	Up
KLF11	0.614399	7.339533	4.530655	2.2497413	9.7129523	2.082511	Up
EPHB6	-0.83004	4.769991	-4.52894	2.2640491	9.7707744	2.076419	Down
LINC01119	-0.55758	5.974093	-4.52805	2.2714960	9.8009316	2.073263	Down
CYYR1	0.549328	8.322998	4.526288	2.2862789	9.8627239	2.067029	Up
ADHFE1	-0.73469	9.019858	-4.52561	2.2920217	9.8855009	2.064619	Down
ATP8A1	-0.62213	9.209878	-4.52525	2.2950931	9.8947518	2.063332	Down
TAGLN	-1.08724	9.390448	-4.52449	2.3198328	9.9896655	2.054226	Down

LCK	0.587251	4.165707	4.522328	2.3199148	9.9896655	2.052995	Up
B4GALT1	-0.63741	6.583813	-4.52194	2.3232480	0.0001	2.051616	Down
TONSL	-0.69637	4.813613	-4.52163	2.3258877	0.0001	2.050524	Down
CNTFR	-0.72744	4.860823	-4.52101	2.3311810	0.0001	2.04834	Down
PCDHGA1	0.59622	7.75944	4.520149	2.3386315	0.000101	2.045274	Up
PDLIM1	-0.73746	10.53915	-4.51836	2.3613205	0.000101	2.036462	Down
EXD2	-0.50882	7.006074	-4.5165	2.3703247	0.000102	2.03234	Down
ZNF598	-0.50508	6.040898	-4.51589	2.3756645	0.000102	2.030178	Down
RIC8B	-0.56254	6.489396	-4.5108	2.4205578	0.000104	2.012192	Down
RASSF8	-0.67748	7.763595	-4.51043	2.4238987	0.000104	2.010867	Down
TIMM44	-0.56275	7.544073	-4.50848	2.4413177	0.000105	2.003988	Down
TNFRSF1B	0.532928	6.886077	4.507667	2.4486677	0.000105	2.0011	Up
IBSP	-0.85256	4.453745	-4.50716	2.4532890	0.000105	1.999289	Down
ABCC2	-0.8886	3.421077	-4.50687	2.4559036	0.000105	1.998265	Down
CCDC39	1.01525	3.84949	4.505004	2.4727899	0.000106	1.991683	Up
ST14	-0.91386	4.888295	-4.50426	2.4795941	0.000106	1.989043	Down
SOX7	0.70278	8.049857	4.504032	2.4816553	0.000106	1.988245	Up
MACO1	-0.57156	7.717887	-4.50364	2.4852618	0.000106	1.98685	Down
CNN3	0.91994	7.884089	4.502846	2.5026447	0.000107	1.980771	Up
XBP1	0.50561	8.112692	4.500977	2.5097070	0.000107	1.977448	Up
USP44	0.763392	3.670622	4.500528	2.5138499	0.000107	1.975864	Up
CIB3	-1.00689	5.021488	-4.49972	2.5227938	0.000108	1.97254	Down
UNKL	-0.61045	7.378917	-4.49956	2.5228059	0.000108	1.972448	Down
OMD	1.466593	5.788548	4.501104	2.5281973	0.000108	1.971584	Up
ZNF862	0.675262	6.957081	4.498663	2.5311582	0.000108	1.969273	Up
ART3	-1.15242	9.833671	-4.49796	2.5575494	0.000109	1.960495	Down
ABCF2-H2	-0.5901	7.945505	-4.49494	2.5660544	0.000109	1.956121	Down
LAMB2P1	-0.56891	4.571021	-4.4947	2.5682940	0.00011	1.955283	Down
MLLT3	0.593502	5.483687	4.493242	2.5821111	0.00011	1.95013	Up
TWNK	-0.77018	5.537553	-4.49217	2.5923238	0.000111	1.946339	Down
RFLNB	0.659325	8.79839	4.491176	2.6017948	0.000111	1.942837	Up
BMP5	1.286143	6.834165	4.492649	2.6079035	0.000111	1.941767	Up
RANGAP1	-0.60128	5.446646	-4.48947	2.6181564	0.000112	1.936816	Down
DUSP8	-0.76255	6.150529	-4.48699	2.6420905	0.000113	1.928077	Down
OSBPL11	-0.68848	9.459675	-4.48668	2.6451113	0.000113	1.926979	Down
PRND	1.743054	3.970337	4.488762	2.6458631	0.000113	1.927917	Up
LYL1	-0.50105	6.1675	-4.48616	2.6501423	0.000113	1.925155	Down
GPAT4	-0.50826	7.359312	-4.48565	2.6551870	0.000113	1.923328	Down
PKD2	0.578422	7.9147	4.485225	2.6592923	0.000113	1.921845	Up
SNPH	-0.8202	4.610304	-4.48344	2.6767527	0.000114	1.91556	Down
LINC0093	0.565535	8.998558	4.479296	2.7178187	0.000116	1.90094	Up
FBXL19	-0.72558	4.14833	-4.47807	2.7300712	0.000116	1.896621	Down
TBXT	-0.77299	4.420605	-4.47749	2.7358453	0.000116	1.894593	Down
MSS51	-1.16702	9.688142	-4.47937	2.7385930	0.000116	1.894835	Down
CPT1A	0.635795	7.530778	4.476653	2.7443019	0.000117	1.891629	Up
TEAD4	-0.74912	8.929674	-4.47685	2.7447133	0.000117	1.891616	Down
UBE2Q2	0.855437	9.408885	4.47569	2.7665866	0.000117	1.884562	Up
ME1	-0.54221	9.569053	-4.47419	2.7692359	0.000118	1.882945	Down
DNASE1L3	0.741043	4.207924	4.471433	2.7973481	0.000119	1.873248	Up
DPEP2	1.118728	3.803968	4.470919	2.8069719	0.000119	1.870188	Up
DLSTP1	-0.74457	8.316338	-4.46585	2.8551637	0.000121	1.853608	Down
POM121L	-0.99275	2.999309	-4.46486	2.8655775	0.000121	1.850113	Down
CBX5	1.005296	8.041114	4.464459	2.8853360	0.000122	1.844349	Up
GLRA1	1.511986	2.807455	4.463312	2.8904057	0.000122	1.842287	Up
CDK6	1.320536	7.806438	4.463388	2.9056363	0.000123	1.838095	Up
ABCC10	0.58079	5.937911	4.459864	2.9184985	0.000123	1.832547	Up
MIR497HC	-0.56296	5.147751	-4.45803	2.9381330	0.000124	1.826111	Down
KRT6B	-0.7	4.913876	-4.45732	2.9458120	0.000124	1.823605	Down

RAMP2	-0.95156	6.040956	-4.45719	2.9504682	0.000125	1.822257	Down
GPR88	1.16598	3.549929	4.456272	2.9614711	0.000125	1.818743	Up
DMBT1	2.722853	4.938668	4.459665	2.9624837	0.000125	1.820384	Up
TTYH2	0.508731	8.279453	4.455481	2.9657067	0.000125	1.817145	Up
ARRB2	0.960395	5.382955	4.454804	2.9730698	0.000125	1.814765	Up
GNB1L	-0.51204	4.10901	-4.45259	2.9972117	0.000126	1.807002	Down
TMEFF1	0.887113	5.01433	4.450889	3.0159651	0.000127	1.801016	Up
SLC16A1-	-0.6284	4.386576	-4.45017	3.0239187	0.000127	1.798488	Down
PDE10A	0.941844	4.919341	4.447293	3.0558944	0.000129	1.788393	Up
PSMD6-A	0.689743	7.014046	4.446707	3.0624555	0.000129	1.786335	Up
SLC38A4	1.209743	5.805175	4.446513	3.0783243	0.00013	1.782066	Up
PLBD1	0.510527	8.085994	4.444634	3.0857528	0.00013	1.779062	Up
DIPK2B	-0.54295	5.767876	-4.44369	3.0964434	0.00013	1.775743	Down
TCEAL7	1.397115	7.976091	4.445888	3.1055052	0.000131	1.774639	Up
LOC10272	-0.54031	4.944179	-4.44247	3.1102083	0.000131	1.771487	Down
TMPRSS6	-0.55361	5.744606	-4.43999	3.1385960	0.000132	1.762768	Down
WDR25	-0.5705	5.033092	-4.43922	3.1473756	0.000132	1.760088	Down
KHDRBS3	-0.61805	8.254636	-4.43917	3.1480458	0.000132	1.759883	Down
FBXW8	0.865441	4.239685	4.436527	3.1785447	0.000134	1.750632	Up
TMPRSS2	-0.54288	5.724663	-4.43579	3.1870735	0.000134	1.748061	Down
IZUMO4	-0.58046	4.700062	-4.43555	3.1898554	0.000134	1.747224	Down
MRPS12	-0.65566	8.598724	-4.43539	3.1917310	0.000134	1.74666	Down
RPS4Y1	4.13531	10.18768	4.44125	3.1920974	0.000134	1.749892	Up
SLC66A1L	0.910758	3.613736	4.434444	3.2028117	0.000134	1.743334	Up
MECR	-0.61561	7.863367	-4.43333	3.2158872	0.000135	1.739425	Down
LEMD2	-0.50136	5.021055	-4.43297	3.2200683	0.000135	1.738179	Down
FAM182A	-0.62954	3.697513	-4.431	3.2434004	0.000136	1.731252	Down
SBK1	-0.80065	8.048528	-4.43081	3.2506355	0.000136	1.729358	Down
TMEM164	-0.52342	7.969416	-4.4303	3.2516683	0.000136	1.728809	Down
OCEL1	-0.57486	5.905488	-4.43024	3.2523374	0.000136	1.728612	Down
TTC28-AS	0.826085	4.271155	4.429033	3.2667131	0.000137	1.724381	Up
LOC10106	1.103245	2.803005	4.427485	3.2852252	0.000137	1.718959	Up
ASB12	-0.91295	8.69126	-4.42715	3.3037478	0.000138	1.714252	Down
MR1	0.652309	5.878184	4.425103	3.3138981	0.000139	1.710623	Up
PPL	1.109877	6.93019	4.426791	3.3145800	0.000139	1.711419	Up
SCAMP1-,	1.106665	4.32187	4.425601	3.3162984	0.000139	1.710325	Up
APOLD1	-0.97568	6.956571	-4.4253	3.3235191	0.000139	1.708408	Down
RPL23AP3	0.685349	8.496497	4.42393	3.3281045	0.000139	1.706519	Up
CD248	0.767774	7.502456	4.423791	3.3297928	0.000139	1.706032	Up
HTR2A	-1.04939	4.05887	-4.42352	3.3358071	0.000139	1.704427	Down
SNHG18	0.555493	5.582925	4.422746	3.3425148	0.00014	1.702374	Up
RPS6KA6	0.749955	5.417772	4.419946	3.3768106	0.000141	1.692582	Up
PITPNM3	0.700092	3.631552	4.418643	3.3929000	0.000141	1.688022	Up
MAB21L1	1.22227	5.654388	4.419538	3.3966626	0.000142	1.687644	Up
FGF9	-1.1014	5.695662	-4.41893	3.4014487	0.000142	1.686168	Down
MIR22HG	0.586348	9.474943	4.417152	3.4113868	0.000142	1.68281	Up
ASB4	-1.03855	7.729569	-4.41901	3.4114481	0.000142	1.683855	Down
CPNE8	1.160361	5.626124	4.416997	3.4282404	0.000143	1.678767	Up
GNAI2	0.695836	7.179436	4.415772	3.4285866	0.000143	1.677986	Up
SUCNR1	1.065707	4.43575	4.414386	3.4522208	0.000144	1.671682	Up
CFAP95	-2.21154	4.674357	-4.41681	3.4631031	0.000144	1.67054	Down
RAMP1	-0.5472	8.779588	-4.41089	3.4901007	0.000145	1.660931	Down
NOP9	-0.506	7.204442	-4.41074	3.4920823	0.000145	1.660386	Down
NMNAT3	-0.62739	7.173197	-4.41021	3.4987475	0.000145	1.658558	Down
CD37	1.405437	4.721451	4.410487	3.5208900	0.000146	1.653655	Up
MIR99AH(	1.06207	6.957685	4.408579	3.5349021	0.000147	1.64938	Up
ERAP2	1.929993	6.307694	4.410491	3.5435020	0.000147	1.648524	Up
PIK3C2A	0.613736	9.186625	4.405703	3.5566657	0.000148	1.642812	Up

CPAMD8	-0.9616	4.250069	-4.40525	3.5624699	0.000148	1.641248	Down
SZT2	-0.54772	5.217693	-4.40498	3.5660880	0.000148	1.640275	Down
PEX19	-0.50717	8.877427	-4.40436	3.5741227	0.000148	1.638116	Down
TLR8	1.398253	4.778969	4.40502	3.5915618	0.000149	1.634594	Up
TARP	0.947549	4.532091	4.402976	3.5921366	0.000149	1.633295	Up
ZMYM1	0.849896	4.906014	4.402775	3.5947690	0.000149	1.632593	Up
SUSD1	0.858644	4.566607	4.401719	3.6086004	0.000149	1.62891	Up
NFATC1	-0.69791	8.203243	-4.40135	3.6134604	0.00015	1.62762	Down
LEPROT	0.610117	9.640596	4.400936	3.6188986	0.00015	1.626178	Up
ZNF850	0.788225	3.607996	4.400651	3.6226560	0.00015	1.625182	Up
DMD	-1.55683	9.588356	-4.4042	3.6254169	0.00015	1.626604	Down
SESN2	-0.68615	5.469718	-4.39938	3.6394361	0.000151	1.620751	Down
CDKL5	0.884991	4.465513	4.399253	3.6411204	0.000151	1.620307	Up
PPIL4	0.668999	6.504208	4.396373	3.6794682	0.000152	1.610262	Up
PDE3A	0.81964	5.895064	4.395971	3.6848440	0.000152	1.608862	Up
NDUFS7	-0.82281	10.2177	-4.39672	3.6906585	0.000153	1.608028	Down
PTPN4	-0.96652	7.301469	-4.39641	3.6948331	0.000153	1.606944	Down
SETD9	-0.74773	6.311348	-4.39521	3.6950888	0.000153	1.6062	Down
ELMOD2	0.508677	7.149279	4.395189	3.6953321	0.000153	1.606137	Up
SWAP70	0.959167	8.611912	4.39369	3.7346463	0.000154	1.596801	Up
SMAD9	1.204482	8.365256	4.393928	3.7392186	0.000154	1.595958	Up
MSX1	0.919349	6.677072	4.391227	3.7575326	0.000155	1.590494	Up
APMAP	0.560425	8.180894	4.390368	3.7606636	0.000155	1.589334	Up
GUCY1B1	0.559769	7.884655	4.389301	3.7752730	0.000156	1.585617	Up
CAVIN2	0.647339	8.70755	4.388957	3.7799940	0.000156	1.584419	Up
ICAM3	-0.58709	6.840956	-4.38708	3.8058397	0.000157	1.577886	Down
SH3BP5L	-0.572	6.322618	-4.38679	3.8098627	0.000157	1.576873	Down
GNG11	0.759947	9.474447	4.387299	3.8120185	0.000157	1.576713	Up
MTRR	-0.59487	8.913321	-4.38501	3.8345750	0.000158	1.570675	Down
H4C13	0.776757	3.655569	4.382648	3.8676077	0.000159	1.562452	Up
TCF4	0.655147	10.75895	4.382839	3.8698015	0.000159	1.562109	Up
ANKRD26	-0.75406	5.423357	-4.38194	3.8775265	0.00016	1.559997	Down
CCL14	1.209359	8.531165	4.382853	3.8925042	0.00016	1.557438	Up
CERCAM	0.835897	6.078785	4.380566	3.8969432	0.00016	1.555209	Up
EPM2A-D	0.596473	5.433378	4.379946	3.9057241	0.000161	1.553051	Up
EFR3B	-0.70938	6.456684	-4.37609	3.9607971	0.000163	1.53963	Down
SHISA2	1.028661	8.334754	4.37711	3.9743697	0.000163	1.537485	Up
DLL4	-0.65069	5.467	-4.37498	3.9767475	0.000163	1.535778	Down
ASF1A	-0.50102	8.278287	-4.37403	3.9905286	0.000164	1.532462	Down
ENTPD7	0.64484	4.747678	4.372888	4.0070344	0.000164	1.528506	Up
ATAD2	0.810858	4.57012	4.372714	4.0095629	0.000165	1.527902	Up
GAREM2	-0.95651	4.796061	-4.37111	4.0329813	0.000165	1.52232	Down
HIVEP1	0.526662	6.497776	4.36994	4.0501021	0.000166	1.51826	Up
C2	1.395535	5.787462	4.369528	4.0850113	0.000167	1.511166	Up
MUC1	-0.50287	5.114725	-4.36666	4.0985332	0.000168	1.506868	Down
MCOLN1	0.510876	6.017867	4.364457	4.1313847	0.000169	1.499217	Up
RGS1	2.09923	3.597292	4.367616	4.1339896	0.000169	1.500541	Up
ALAS1	-0.55406	9.78839	-4.36426	4.1343009	0.000169	1.498541	Down
P2RY6	-0.50674	5.506883	-4.36401	4.1380600	0.000169	1.49767	Down
MVK	-0.50002	6.519923	-4.36315	4.1509619	0.00017	1.494687	Down
ACSF2	-0.5581	6.218453	-4.36178	4.1716294	0.000171	1.489928	Down
MMP14	-0.58289	7.273843	-4.36156	4.1749597	0.000171	1.489163	Down
MOV10	0.54866	5.18872	4.361354	4.1780785	0.000171	1.488448	Up
IMMP2L	-0.55696	7.483262	-4.36117	4.1808388	0.000171	1.487815	Down
FTHL17	-0.97988	3.976624	-4.35986	4.2007067	0.000172	1.483272	Down
MIR23AH(	0.675092	8.070638	4.358885	4.2156136	0.000172	1.479878	Up
TIA1	0.579922	8.957077	4.357876	4.2310287	0.000173	1.47638	Up
ZNF556	1.458702	7.612834	4.356546	4.3003758	0.000175	1.462637	Up

TFAP4	-0.64371	5.213245	-4.35259	4.3127589	0.000176	1.458049	Down
RBMS3-AS	-0.72665	6.243139	-4.35176	4.3257829	0.000176	1.45516	Down
CENPBD1P	0.503739	6.313047	4.351451	4.3305646	0.000177	1.454101	Up
SRRD	-0.54249	7.723087	-4.35084	4.3401714	0.000177	1.451978	Down
YIPF2	-0.67709	6.762426	-4.34849	4.3772538	0.000178	1.443828	Down
PSENEN	-0.63007	6.951995	-4.34798	4.3853552	0.000179	1.442056	Down
FBLIM1	1.080888	6.675799	4.345133	4.4490105	0.000181	1.428917	Up
SFRP1	1.387498	6.279582	4.345183	4.4607986	0.000181	1.426837	Up
HOXA11	-0.7493	5.26397	-4.34323	4.4613043	0.000181	1.425608	Down
MICA	0.618136	5.979531	4.342392	4.4747613	0.000182	1.422723	Up
TTC9B	-0.73191	3.357096	-4.3421	4.4795044	0.000182	1.421708	Down
MICB	1.114142	4.239447	4.342159	4.4914144	0.000182	1.419627	Up
FAM76B	0.60636	6.844591	4.340333	4.5081804	0.000183	1.415595	Up
ITGB1BP2	0.682398	9.519878	4.340124	4.5115847	0.000183	1.414872	Up
TRIM59	1.369218	3.811138	4.339252	4.5490967	0.000185	1.407769	Up
HEATR6	-0.5524	6.234395	-4.33359	4.6193820	0.000187	1.392256	Down
THBD	0.672801	7.984071	4.329492	4.6881517	0.00019	1.378104	Up
AQP4	-1.84429	9.005837	-4.33204	4.7057279	0.000191	1.376614	Down
DZIP1	0.661632	6.061362	4.328398	4.7066931	0.000191	1.374324	Up
NBDY	0.670581	7.986935	4.328394	4.7067474	0.000191	1.374313	Up
ATP23	-1.12199	7.046058	-4.32907	4.7277137	0.000191	1.37117	Down
KLHL6	0.642248	4.293523	4.326937	4.7315573	0.000192	1.369278	Up
BCDIN3D	0.513691	4.914715	4.324231	4.7779651	0.000193	1.359931	Up
LENG1	-0.50234	5.057464	-4.32324	4.7951069	0.000194	1.356502	Down
ODC1	-0.50829	10.13259	-4.32288	4.8012371	0.000194	1.355279	Down
ZNF174	-0.76671	7.193053	-4.32264	4.8053910	0.000194	1.354451	Down
STOML2	-0.50645	8.992062	-4.32163	4.8229160	0.000195	1.350965	Down
ENO3	-0.50506	13.25401	-4.32012	4.8539973	0.000196	1.344973	Down
TM4SF1	0.500007	10.28672	4.319647	4.8575858	0.000196	1.344106	Up
ENTPD6	-0.54936	5.947023	-4.31911	4.8669601	0.000197	1.34226	Down
CFAP206	-0.79155	4.148159	-4.31876	4.8731748	0.000197	1.341038	Down
PSMD3	-0.60417	8.675019	-4.31863	4.8754972	0.000197	1.340582	Down
PPM1M	0.603175	5.960296	4.316783	4.9079836	0.000198	1.334224	Up
CCL13	1.397652	4.658451	4.317559	4.9277643	0.000199	1.331481	Up
KLRK1	0.845982	5.124044	4.315479	4.9310923	0.000199	1.329726	Up
XPO4	-0.95183	9.66193	-4.31456	4.9811512	0.000201	1.321163	Down
TIGAR	-0.63684	6.936892	-4.3083	5.0601499	0.000204	1.304992	Down
FRG1HP	0.722028	5.599372	4.30817	5.0625885	0.000204	1.304531	Up
PTCD1	-0.58066	7.039068	-4.30634	5.0960929	0.000205	1.298217	Down
GDF5	-0.71419	3.777614	-4.3063	5.0968087	0.000205	1.298082	Down
CXCL9	1.256925	4.980549	4.307409	5.1009546	0.000205	1.298089	Up
CUL2	-0.55879	8.294256	-4.3045	5.1298759	0.000206	1.291892	Down
LINC0268!	-0.78685	4.078636	-4.30333	5.1515124	0.000207	1.287863	Down
YPEL3	-0.64194	8.421027	-4.30286	5.1601743	0.000207	1.286255	Down
RRAGD	-0.54813	10.58163	-4.29801	5.2509868	0.000211	1.269557	Down
BCL7A	-0.97273	5.782683	-4.2975	5.2700381	0.000212	1.26638	Down
CASR	-0.81102	4.532602	-4.29565	5.2958594	0.000213	1.261412	Down
ZNF667-A	0.73685	7.205559	4.294789	5.3121900	0.000213	1.258466	Up
LOC38983	-1.51452	7.759882	-4.29813	5.3154109	0.000213	1.259953	Down
PRKAA1	0.934363	6.478546	4.294595	5.3295545	0.000214	1.255763	Up
MYLK2	-1.20528	9.181386	-4.28993	5.4462422	0.000218	1.235842	Down
GOLGA8A	0.960743	8.681194	4.289178	5.4541707	0.000219	1.234246	Up
MOCS3	-0.71473	4.695382	-4.28627	5.4771516	0.000219	1.2292	Down
ZNF649	-0.5883	5.14608	-4.28608	5.4809715	0.00022	1.228533	Down
SLC9A1	-0.51814	6.468807	-4.28556	5.4913229	0.00022	1.226728	Down
SGTB	0.571408	7.133285	4.283741	5.5271948	0.000221	1.220497	Up
PLAC4	-0.69227	4.014969	-4.28221	5.5576613	0.000222	1.215238	Down
C6	1.068148	4.542367	4.282572	5.5647938	0.000223	1.214436	Up

IL31RA	-1.1113	4.973385	-4.28127	5.5944967	0.000224	1.209448	Down
BAIAP3	-0.72159	4.699907	-4.28014	5.5991485	0.000224	1.208122	Down
C14orf132	1.184829	7.366918	4.280266	5.6337829	0.000225	1.203315	Up
ANXA2P3	1.010028	5.189215	4.278852	5.6348300	0.000225	1.202332	Up
FAM124B	1.090292	3.683972	4.278034	5.6487707	0.000226	1.199892	Up
SLC27A3	0.798112	6.172119	4.277446	5.6534369	0.000226	1.19889	Up
PTPRJ	0.843316	5.763693	4.276175	5.6792669	0.000227	1.194529	Up
CSGALNA	0.751251	8.399556	4.276041	5.6896515	0.000227	1.193003	Up
CBX3	0.50374	10.21432	4.275439	5.6942632	0.000227	1.192006	Up
N4BP2	0.811776	5.83539	4.27492	5.7048809	0.000228	1.190224	Up
MYC	1.01144	7.626027	4.275121	5.7356039	0.000229	1.186092	Up
SMIM17	1.036589	3.282887	4.273187	5.7404370	0.000229	1.18428	Up
LCAT	-0.66611	5.069866	-4.27311	5.7419979	0.000229	1.184019	Down
AS3MT	-1.01118	5.698547	-4.27373	5.7440491	0.000229	1.184102	Down
KRTAP19-	1.451981	2.462121	4.27281	5.7523480	0.000229	1.182416	Up
FLYWCH2	-0.8133	5.254876	-4.2722	5.7608079	0.00023	1.180891	Down
FOXN3-A!	-0.55147	4.634639	-4.27195	5.7660310	0.00023	1.180024	Down
DRD2	-0.58694	5.546884	-4.27143	5.7767869	0.00023	1.178241	Down
LINC0300(	0.821776	5.90828	4.267128	5.8664128	0.000233	1.163514	Up
TNS2-AS1	-0.85333	3.824301	-4.26537	5.9033733	0.000235	1.157506	Down
HMGB3	0.774041	6.089852	4.265304	5.9048661	0.000235	1.157264	Up
LPCAT1	0.741568	5.897983	4.264193	5.9283935	0.000236	1.153461	Up
NDUFA7	-0.52981	9.788064	-4.26408	5.9307036	0.000236	1.153088	Down
PLAAT5	1.172587	4.034913	4.26445	5.9431197	0.000236	1.151652	Up
MCRIP1	0.528303	6.472746	4.263175	5.9500487	0.000236	1.149973	Up
CKAP4	0.625756	8.026093	4.2616	5.9836998	0.000238	1.144579	Up
GCSAM	0.914458	2.608593	4.261381	5.9883722	0.000238	1.143833	Up
LAMB2	0.513349	8.336648	4.259008	6.0394523	0.00024	1.135709	Up
EDN3	1.71648	5.221585	4.261668	6.0396498	0.00024	1.137264	Up
ZNF204P	-1.02621	4.267882	-4.25913	6.0449749	0.00024	1.135062	Down
TMEM151	-0.65339	3.961901	-4.25814	6.0581693	0.00024	1.13275	Down
CMA1	1.460791	4.714017	4.257136	6.1197844	0.000243	1.124155	Up
PADI4	-0.84873	4.691283	-4.2529	6.1728761	0.000245	1.114811	Down
ZNF107	0.76514	4.582993	4.251635	6.2008483	0.000246	1.110488	Up
HACE1	0.933942	5.812948	4.251064	6.2213268	0.000246	1.107545	Up
TMEM71	0.921947	4.131436	4.249701	6.2438488	0.000247	1.10388	Up
KCP	-0.73889	4.703016	-4.24892	6.2614072	0.000248	1.101194	Down
PRKAG2	-0.75362	6.307614	-4.2488	6.2639068	0.000248	1.100813	Down
TPX2	0.717589	3.971556	4.245227	6.3444749	0.000251	1.088593	Up
RPUSD4	-0.63798	9.751653	-4.23994	6.4653752	0.000256	1.070545	Down
NME1	0.541549	8.370201	4.238305	6.5032429	0.000257	1.064962	Up
PROSER2	0.543068	5.2294	4.237937	6.5117908	0.000257	1.063706	Up
FAM131B-	0.826049	6.856871	4.237869	6.5181864	0.000257	1.062891	Up
CLU	1.276301	8.466709	4.239955	6.5251622	0.000258	1.063294	Up
VCPKMT	0.510245	6.926034	4.236604	6.5428452	0.000258	1.059158	Up
MDGA1	-0.83247	4.368628	-4.23612	6.5542412	0.000259	1.057494	Down
RCOR2	-0.56161	4.950284	-4.23574	6.5630982	0.000259	1.056203	Down
SCARF2	-0.68296	4.289848	-4.23554	6.5677259	0.000259	1.055529	Down
UBE2S	0.634118	6.2267	4.234199	6.5992383	0.00026	1.050954	Up
KLRC4-KL	0.715528	3.579963	4.233445	6.6170124	0.000261	1.048382	Up
STXBP5	-0.73225	6.458961	-4.23319	6.6230278	0.000261	1.047514	Down
EFCAB2	-0.6738	6.890174	-4.2319	6.6536618	0.000262	1.043103	Down
IFNAR1	0.591255	5.907306	4.231662	6.6592145	0.000262	1.042305	Up
ZDHHC2	-0.6559	8.781858	-4.22981	6.7033761	0.000264	1.035987	Down
NFE2L3	0.650929	4.082571	4.229308	6.7153549	0.000264	1.034281	Up
GRM1	-0.69156	4.079517	-4.22756	6.7573882	0.000266	1.028316	Down
CREB3L1	0.961018	5.762193	4.227712	6.7656065	0.000266	1.027452	Up
CASZ1	-0.61767	7.610727	-4.22581	6.7997101	0.000267	1.022349	Down

PKNOX2	-0.52878	7.349247	-4.22218	6.8882505	0.00027	1.009984	Down
PTPA	-0.57067	8.880768	-4.22169	6.9002302	0.000271	1.008324	Down
VANGL1	0.615549	5.438794	4.220565	6.9278725	0.000272	1.004503	Up
MTNR1B	-0.84929	4.210141	-4.22052	6.9289640	0.000272	1.004352	Down
GFOD1	-0.60957	8.058945	-4.22036	6.9330439	0.000272	1.00379	Down
LMTK2	-0.64564	6.475779	-4.21997	6.9426344	0.000272	1.002469	Down
SSC5D	-0.78409	6.529121	-4.21912	6.9635722	0.000273	0.999591	Down
PI15	1.438777	4.96047	4.220303	6.9783799	0.000273	0.998629	Up
ZNF169	-0.55805	6.376947	-4.21851	6.9787912	0.000273	0.997505	Down
TPT1-AS1	0.846889	5.922535	4.218208	6.9862574	0.000274	0.996483	Up
DOCK1	0.519488	6.371407	4.217583	7.0018330	0.000274	0.994355	Up
INTU	0.924286	4.49961	4.216913	7.0185453	0.000275	0.992077	Up
NOC4L	-0.7699	5.618652	-4.21691	7.0186153	0.000275	0.992067	Down
CARMIL1	1.438331	5.047495	4.217005	7.0606980	0.000276	0.987421	Up
H2BC6	-0.5849	6.049325	-4.21362	7.1014012	0.000278	0.980862	Down
CHST7	0.52463	5.421512	4.212851	7.1207448	0.000278	0.978263	Up
MUC6	-0.81268	4.440479	-4.21109	7.1655117	0.00028	0.972274	Down
TELO2	-0.70222	5.784985	-4.20976	7.1995886	0.000281	0.967741	Down
SACM1L	0.516146	7.899126	4.20807	7.2428741	0.000283	0.962014	Up
PRKCB	0.955389	5.121473	4.207754	7.2562444	0.000283	0.960374	Up
CARMN	-0.60744	7.380257	-4.20753	7.2568119	0.000283	0.960177	Down
NIPSNAP1	0.536128	5.912745	4.205288	7.3148793	0.000285	0.952562	Up
LINC0304(	-0.99151	3.733992	-4.20422	7.3426994	0.000286	0.948936	Down
NLRP7	-0.69768	4.130356	-4.20298	7.3751683	0.000287	0.944721	Down
LINGO1	-0.74275	4.789348	-4.20262	7.3845065	0.000288	0.943512	Down
NOP16	-0.54856	8.302926	-4.20023	7.4476049	0.00029	0.935384	Down
BICDL2	-0.71863	4.920494	-4.20012	7.4504712	0.00029	0.935016	Down
JAK2	-0.7606	7.432989	-4.20001	7.4574627	0.00029	0.934213	Down
ARX	-0.68477	8.468037	-4.19939	7.4699086	0.000291	0.932527	Down
AGFG2	-0.6475	4.190404	-4.19547	7.5745501	0.000295	0.919239	Down
FZD6	0.722784	6.63227	4.194076	7.6120953	0.000296	0.914516	Up
MAPT	-0.68975	8.133912	-4.19304	7.6402209	0.000297	0.910993	Down
LRRC77P	-0.73163	4.243788	-4.19107	7.6938349	0.000299	0.904314	Down
SDHAF4	-0.61	6.740992	-4.19047	7.7102329	0.000299	0.902281	Down
RXRA	-0.54763	9.018754	-4.18884	7.7547839	0.000301	0.896778	Down
MFAP5	0.922264	9.459139	4.189809	7.7761857	0.000302	0.895201	Up
ABCG1	0.666821	8.410538	4.186948	7.8070839	0.000303	0.890359	Up
SFRP5	-0.80126	4.681539	-4.18669	7.8142838	0.000303	0.889478	Down
RBMS3	0.702507	9.075794	4.186755	7.8193492	0.000303	0.889011	Up
KIF6	-0.511	5.874889	-4.18456	7.8735636	0.000305	0.882261	Down
EFHD2	0.775466	6.86884	4.182774	7.9234910	0.000307	0.876224	Up
WIPI1	-0.54941	9.58413	-4.18	8.0017614	0.00031	0.866838	Down
DUSP18	0.554874	5.255015	4.17953	8.0151125	0.00031	0.865246	Up
DOK7	-0.59677	7.352564	-4.1773	8.0786578	0.000313	0.857705	Down
MYH4	2.102431	7.583759	4.179121	8.1196975	0.000314	0.854841	Up
C2orf88	-0.84445	6.973939	-4.17636	8.1200031	0.000314	0.853133	Down
SLC35B3	0.529725	7.497207	4.174438	8.1609858	0.000316	0.848024	Up
GIPC2	0.529439	5.066698	4.172036	8.2307093	0.000318	0.839902	Up
MIA	-0.75631	3.632298	-4.1697	8.2990579	0.00032	0.832007	Down
LINC0162(	-0.62727	4.773868	-4.16789	8.3524953	0.000322	0.825879	Down
ZNF667	0.573995	5.397428	4.167525	8.3631375	0.000323	0.824664	Up
CA13	1.146745	3.537531	4.16705	8.3943648	0.000324	0.821457	Up
NXPH3	0.605274	5.33082	4.1658	8.4143432	0.000325	0.818836	Up
RECK	0.73778	8.725925	4.160349	8.5917769	0.000331	0.799192	Up
CCAR2	-0.80245	6.260185	-4.15963	8.6000825	0.000331	0.797995	Down
PCDHB7	0.652664	4.510055	4.159086	8.6164865	0.000332	0.796176	Up
LINC0111(	-0.78333	4.109439	-4.15864	8.6300173	0.000332	0.794678	Down
MROH7	-0.80237	3.88284	-4.15859	8.6316994	0.000332	0.794492	Down

ATP2B4	0.54371	8.618147	4.156821	8.6857252	0.000334	0.788537	Up
WDR4	0.558996	5.143074	4.155629	8.7223959	0.000336	0.784516	Up
KIAA1958	0.640148	4.58748	4.155592	8.7235336	0.000336	0.784391	Up
AKIRIN1	-0.51504	7.169322	-4.15503	8.7409135	0.000336	0.782492	Down
CLHC1	0.840663	4.002693	4.155024	8.7410304	0.000336	0.782479	Up
TMEM182	0.513167	10.54198	4.15486	8.7461163	0.000336	0.781924	Up
XAF1	0.908225	7.949199	4.155101	8.7699692	0.000337	0.779942	Up
EFCC1	-0.61081	3.844834	-4.15387	8.7768135	0.000337	0.77858	Down
PLVAP	1.114422	6.627667	4.153719	8.8343756	0.000339	0.773381	Up
TOP3A	-1.04311	5.198679	-4.15144	8.8780785	0.000341	0.768136	Down
KBTBD12	-0.53116	9.280284	-4.15025	8.8895150	0.000341	0.766403	Down
NEFH	1.212552	3.867521	4.15097	8.8987249	0.000341	0.766032	Up
MYH1	2.053904	12.2574	4.154642	8.9056619	0.000342	0.767666	Up
GTF2F2	-0.60959	7.671555	-4.14879	8.9356900	0.000343	0.761459	Down
KYAT3	0.539018	8.065833	4.14795	8.9621204	0.000344	0.758641	Up
MYOCD	-0.98673	4.077431	-4.14771	8.9757200	0.000344	0.75731	Down
TTC14	0.649843	7.904766	4.146847	8.9970923	0.000345	0.754924	Up
NEDD9	-0.70331	7.518645	-4.14631	9.0142961	0.000345	0.753101	Down
LINC0047;	1.225224	3.279247	4.144644	9.0883808	0.000348	0.745695	Up
POMC	-1.19334	3.454503	-4.14449	9.0947303	0.000348	0.745055	Down
SERPINI1	1.02857	7.24828	4.144086	9.1389953	0.00035	0.741019	Up
AQP1	0.5975	10.23151	4.139665	9.2279149	0.000353	0.730754	Up
RPS6KL1	-0.67873	5.572825	-4.13677	9.3226863	0.000356	0.721005	Down
AKAP10	0.709273	7.342302	4.136104	9.3444615	0.000357	0.71878	Up
GARIN4	-0.53145	4.845474	-4.13478	9.3881678	0.000358	0.714328	Down
FRAT2	-0.54364	6.906063	-4.13457	9.3950435	0.000359	0.713629	Down
KLHL13	0.593503	6.674067	4.134563	9.3953320	0.000359	0.7136	Up
ADH1A	0.642883	4.306635	4.132757	9.4553201	0.000361	0.707528	Up
AAMDC	-0.62489	10.12361	-4.13269	9.4574428	0.000361	0.707314	Down
ANAPC1	-0.68255	7.613463	-4.13223	9.4728004	0.000361	0.705767	Down
S100A9	-1.6581	5.754263	-4.13476	9.4798200	0.000362	0.706741	Down
GPRASP3	-0.53283	5.241833	-4.13148	9.4977870	0.000362	0.703254	Down
TMEM267	0.644539	6.448794	4.131164	9.5085134	0.000363	0.702177	Up
MRAS	0.693704	8.624997	4.13017	9.5459970	0.000364	0.6985	Up
PLK5	-0.61491	4.439732	-4.12994	9.5495345	0.000364	0.69807	Down
CDK5R1	1.059112	4.70411	4.130684	9.5519459	0.000364	0.69833	Up
MBIP	0.517323	6.984262	4.12896	9.5826005	0.000365	0.694773	Up
MLKL	0.55972	6.389603	4.128311	9.6045103	0.000366	0.692595	Up
DAGLB	-0.51234	6.473763	-4.1256	9.6966085	0.000369	0.683492	Down
CACNA2D	-0.75959	5.654391	-4.12461	9.7303166	0.00037	0.680182	Down
OTUD1	-0.95352	10.12681	-4.12569	9.7507427	0.000371	0.67921	Down
KCNE5	1.927236	5.929606	4.124114	9.8555181	0.000375	0.669919	Up
H2BC8	0.584238	3.612349	4.120798	9.8618028	0.000375	0.66738	Up
MERTK	1.039381	5.140202	4.12114	9.8780246	0.000375	0.666311	Up
SH3KBP1	-0.57189	8.592662	-4.11903	9.9233730	0.000377	0.661444	Down
SLC25A42	-0.52667	6.848026	-4.11826	9.9501480	0.000378	0.658875	Down
ARID5B	0.570022	9.474191	4.117556	9.9748802	0.000379	0.656507	Up
IMPG2	-0.5148	4.452867	-4.11266	0.000101	0.000385	0.640096	Down
PWWP3B	-0.74968	8.019999	-4.10902	0.000103	0.00039	0.626781	Down
RPP25	-0.91382	5.458874	-4.10816	0.000103	0.000391	0.624539	Down
MDFI	-0.54137	5.396295	-4.1075	0.000103	0.000391	0.622804	Down
DBH-AS1	-0.66963	6.017164	-4.10603	0.000104	0.000393	0.617902	Down
VDR	0.906742	5.020914	4.105703	0.000104	0.000394	0.616804	Up
LMNTD2-.	-0.54933	5.616079	-4.10464	0.000104	0.000395	0.613242	Down
SEPSECS	0.52479	6.164972	4.102806	0.000105	0.000397	0.607113	Up
UPP1	-0.56585	5.214979	-4.1026	0.000105	0.000398	0.606432	Down
FANCE	-0.70338	5.568367	-4.10194	0.000105	0.000398	0.604213	Down
EOMES	1.097406	3.666945	4.102093	0.000106	0.000399	0.603217	Up

MANEAL	-0.54595	4.213318	-4.10038	0.000106	0.0004	0.598984	Down
RNASEL	0.611249	6.334886	4.09852	0.000107	0.000403	0.592779	Up
PLPPR1	1.014186	3.983621	4.097493	0.000107	0.000404	0.588404	Up
CYC1	-0.56519	11.30305	-4.0967	0.000107	0.000405	0.586351	Down
NTN3	-0.61335	3.547946	-4.0965	0.000107	0.000405	0.586012	Down
HHEX	0.722898	5.720402	4.096181	0.000108	0.000405	0.58496	Up
PLCL2	-0.57032	9.253601	-4.09602	0.000108	0.000406	0.584414	Down
QTRT2	0.613305	5.110602	4.095184	0.000108	0.000407	0.58163	Up
PDXK	0.504099	7.063731	4.094743	0.000108	0.000407	0.580155	Up
EGF	-0.73435	8.329239	-4.09371	0.000109	0.000409	0.575631	Down
NDRG4	-0.99454	4.777184	-4.09118	0.00011	0.000412	0.56686	Down
ACTL8	-0.50776	3.756049	-4.09005	0.00011	0.000413	0.564468	Down
FOXO6	0.640826	6.827703	4.089245	0.00011	0.000414	0.561794	Up
PRKAG1	-0.58327	9.136419	-4.08837	0.000111	0.000415	0.558888	Down
DIRAS3	1.214753	4.61723	4.088702	0.000111	0.000417	0.556705	Up
RPP14	-0.70786	9.123223	-4.08733	0.000111	0.000417	0.554252	Down
PI4K2B	0.637254	5.414585	4.083161	0.000113	0.000422	0.541494	Up
SCNN1A	-0.60679	5.413859	-4.08187	0.000113	0.000424	0.537178	Down
TPK1	0.620202	5.330487	4.081394	0.000113	0.000425	0.535601	Up
ARHGDIG	-0.83765	4.212608	-4.08082	0.000113	0.000425	0.533703	Down
MDK	1.869168	4.713676	4.082938	0.000114	0.000426	0.53248	Up
TMEM254	0.548855	4.850271	4.079488	0.000114	0.000427	0.529247	Up
GABRB3	-0.57659	3.852217	-4.07945	0.000114	0.000427	0.529129	Down
FAR1	0.53727	8.163264	4.078614	0.000114	0.000428	0.526334	Up
UGT3A1	-1.41125	5.895259	-4.07988	0.000114	0.000428	0.526153	Down
RARRES1	1.411407	4.656833	4.073183	0.000117	0.000438	0.503876	Up
PABPC4L	0.683961	4.080936	4.070533	0.000118	0.00044	0.499416	Up
PLAT	0.79622	7.494665	4.066735	0.000119	0.000446	0.48519	Up
EP300-AS	0.521846	4.468158	4.064864	0.00012	0.000448	0.480554	Up
HSD11B2	0.909253	4.052736	4.064211	0.00012	0.000449	0.478382	Up
MAP1B	0.811146	9.029495	4.06173	0.000122	0.000454	0.46757	Up
CTDSPL	0.542298	8.278171	4.060299	0.000122	0.000454	0.465376	Up
ELAVL4	-0.50084	4.257573	-4.05979	0.000122	0.000455	0.463672	Down
CD200R1	0.61722	4.136113	4.058939	0.000122	0.000456	0.460857	Up
TRIM9	1.483319	3.625099	4.060392	0.000123	0.000457	0.461374	Up
CTSB	0.690287	10.48471	4.058725	0.000123	0.000458	0.458054	Up
BMPER	1.008568	4.42935	4.058162	0.000123	0.000458	0.456796	Up
FAM72C	1.310885	4.106081	4.058721	0.000123	0.000458	0.455827	Up
TPSAB1	1.484732	7.653776	4.05946	0.000124	0.000459	0.454496	Up
NXPE3	-0.80907	8.049416	-4.05653	0.000124	0.000461	0.450754	Down
ADARB1	0.532688	6.987205	4.054865	0.000124	0.000462	0.447325	Up
AOC4P	-0.78425	4.051682	-4.05043	0.000126	0.000469	0.432607	Down
KREMEN1	0.601274	8.259342	4.048778	0.000127	0.000471	0.427119	Up
SLAMF8	0.653619	4.357984	4.0457	0.000128	0.000476	0.41691	Up
KIF25	-0.91251	4.112797	-4.04443	0.000129	0.000478	0.412689	Down
C5AR1	1.229331	5.14058	4.04391	0.00013	0.000481	0.406746	Up
SUSD4	0.646761	4.271458	4.040108	0.000131	0.000484	0.398375	Up
KLK7	-0.53593	4.543636	-4.04004	0.000131	0.000484	0.398149	Down
TGFB3	0.820372	7.594392	4.039838	0.000131	0.000486	0.395428	Up
H2AC8	0.928749	3.511259	4.038346	0.000132	0.000487	0.39254	Up
CD8A	1.327766	4.250506	4.037715	0.000133	0.00049	0.38623	Up
PC	-0.56368	5.469557	-4.03595	0.000133	0.00049	0.384615	Down
CEBPE	-0.67106	4.035808	-4.03575	0.000133	0.000491	0.383948	Down
C9orf40	0.535979	5.19922	4.034597	0.000133	0.000492	0.380127	Up
UCP3	-1.4236	8.304728	-4.03712	0.000134	0.000493	0.380562	Down
NAPSB	0.879194	3.974307	4.031262	0.000135	0.000498	0.369088	Up
KRBOX4	0.512669	5.977643	4.030856	0.000135	0.000498	0.367746	Up
QSOX1	-0.59365	5.838764	-4.0301	0.000135	0.000499	0.365248	Down

MYO5C	0.658601	8.03926	4.027689	0.000137	0.000503	0.357272	Up
DENND2C	-0.65208	8.254789	-4.02464	0.000138	0.000508	0.347189	Down
RAB7B	0.777266	4.640095	4.024638	0.000138	0.000508	0.347184	Up
CX3CR1	0.763988	8.35069	4.025025	0.000138	0.000509	0.346731	Up
STK11	-0.51713	6.800132	-4.02433	0.000138	0.000509	0.346176	Down
PGLS	0.501768	7.078197	4.02292	0.000139	0.000511	0.341508	Up
SPSB1	-0.67728	5.895055	-4.02075	0.00014	0.000515	0.33435	Down
SRC	-0.63968	4.812292	-4.02063	0.00014	0.000515	0.33395	Down
NICOL1	0.93132	6.384188	4.01984	0.000141	0.000518	0.329316	Up
KCNN3	0.677538	7.045239	4.018447	0.000141	0.000519	0.326734	Up
DNAJB14	0.938263	7.972358	4.017987	0.000142	0.000522	0.321251	Up
HNF1A	-0.56424	5.790477	-4.01615	0.000142	0.000522	0.319151	Down
VAV1	0.656013	4.271566	4.013242	0.000144	0.000528	0.309553	Up
ARHGEF26	0.615716	5.612259	4.012122	0.000144	0.000529	0.30586	Up
DUS3L	-0.55161	4.774755	-4.01144	0.000144	0.00053	0.303599	Down
MFSD14CI	-0.51691	6.277938	-4.0114	0.000144	0.00053	0.30348	Down
EBLN3P	0.641237	8.447985	4.011394	0.000145	0.00053	0.303458	Up
PRTG	0.557089	4.730209	4.011274	0.000145	0.00053	0.303063	Up
ST18	-0.61452	4.370303	-4.01002	0.000145	0.000533	0.298921	Down
SLC25A33	-0.92128	7.162033	-4.0108	0.000145	0.000533	0.299044	Down
LINC0135!	0.690851	5.109789	4.009836	0.000145	0.000533	0.29832	Up
PLEKHG4	0.818992	4.64325	4.009398	0.000146	0.000533	0.296875	Up
ASB15	-1.23697	5.452703	-4.00878	0.000147	0.000537	0.29072	Down
LRRC1	0.638235	4.038998	4.006894	0.000147	0.000538	0.288621	Up
IQGAP3	-0.55806	3.787364	-4.00686	0.000147	0.000538	0.288518	Down
FARP1	0.591767	6.851929	4.006063	0.000147	0.000539	0.285884	Up
HTRA3	0.699534	7.729188	4.005028	0.000148	0.000541	0.282434	Up
MAN2C1	-0.51693	6.953062	-4.00499	0.000148	0.000541	0.282338	Down
GIN1	-0.51044	7.527618	-4.00452	0.000148	0.000542	0.280785	Down
ALDH5A1	-0.60933	9.033116	-4.003	0.000149	0.000544	0.275792	Down
SFRP2	1.980642	7.145218	4.005458	0.000149	0.000545	0.276198	Up
RHBDD1	-0.50165	8.348528	-4.00251	0.000149	0.000545	0.27417	Down
PAN3-AS1	0.60096	4.172437	4.001507	0.00015	0.000547	0.270872	Up
CCDC28B	-0.59504	8.870272	-4.00046	0.00015	0.000549	0.267414	Down
EPB41L1	-0.74222	4.32949	-4.00014	0.00015	0.000549	0.266362	Down
FOXF2	0.556422	4.150248	3.999299	0.000151	0.00055	0.263604	Up
AOC3	1.05102	8.681312	4.000662	0.000151	0.000551	0.264014	Up
CNGA1	-0.84453	4.16323	-3.99892	0.000151	0.000551	0.26237	Down
EBLN2	0.71812	5.735032	3.99753	0.000152	0.000553	0.257783	Up
HBM	-0.91409	4.063356	-3.99644	0.000152	0.000555	0.254192	Down
CTSV	0.564074	4.369617	3.99453	0.000153	0.000559	0.247911	Up
ZNF577	-0.53645	5.527163	-3.99203	0.000155	0.000563	0.239693	Down
DDX3Y	2.73716	7.563608	3.993451	0.000156	0.000569	0.232712	Up
FAM184B	-1.31079	5.088841	-3.99026	0.000156	0.000569	0.229821	Down
DKFZP586	0.588673	8.115388	3.988617	0.000156	0.000569	0.228471	Up
SOX12	0.87995	5.272827	3.988436	0.000157	0.00057	0.227636	Up
PIGH	-0.57111	7.578245	-3.98807	0.000157	0.00057	0.226668	Down
ESR2	-0.78672	4.12074	-3.98783	0.000157	0.00057	0.225892	Down
NEUROG1	-0.67449	4.420388	-3.98245	0.00016	0.00058	0.20822	Down
SMAGP	0.770393	4.076044	3.982222	0.00016	0.000581	0.207467	Up
HTATIP2	-0.683	7.730695	-3.98186	0.00016	0.000581	0.206263	Down
FAM43A	0.68942	7.310079	3.980498	0.000161	0.000584	0.201807	Up
NNAT	0.901049	5.948332	3.978328	0.000162	0.000589	0.19342	Up
GPR75	-0.61843	3.180767	-3.97554	0.000164	0.000594	0.185545	Down
CALD1	0.702725	10.73335	3.975849	0.000164	0.000595	0.184191	Up
FSD1L	-0.71641	7.369029	-3.97434	0.000164	0.000596	0.181555	Down
DHX34	-0.58859	5.421795	-3.97177	0.000166	0.000601	0.17318	Down
PSPHP1	2.69334	6.317135	3.974425	0.000167	0.000604	0.170446	Up

TPSB2	1.292345	7.50866	3.972182	0.000167	0.000604	0.168733	Up
FBXL2	0.987267	4.649754	3.969693	0.000167	0.000606	0.164836	Up
APOB	-1.10275	3.311191	-3.96858	0.000168	0.000607	0.161936	Down
CTBS	0.571426	6.457736	3.963591	0.00017	0.000617	0.146394	Up
CEND1	-0.57515	3.707752	-3.96241	0.000171	0.000619	0.142521	Down
LINC01718	-0.62204	3.610363	-3.96102	0.000172	0.000622	0.137997	Down
PTCHD1	1.063514	4.275724	3.960446	0.000173	0.000625	0.134195	Up
LRCH2	0.94465	4.3894	3.95529	0.000176	0.000634	0.118429	Up
GBP1	0.842918	7.806918	3.95546	0.000176	0.000635	0.11748	Up
CERS6	-0.70237	6.855699	-3.95326	0.000177	0.000638	0.112598	Down
SLCO1C1	0.626134	3.494895	3.952529	0.000177	0.000639	0.110221	Up
MATCAP1	0.605844	4.500766	3.952375	0.000177	0.000639	0.109716	Up
TUBB4A	-0.91435	3.044882	-3.95101	0.000178	0.000642	0.105256	Down
PFKP	0.517096	7.411012	3.95069	0.000178	0.000642	0.104214	Up
CAVIN3	0.628121	8.028213	3.950047	0.000179	0.000644	0.102111	Up
IGHM	-0.74614	6.670276	-3.95001	0.000179	0.000644	0.101981	Down
MARK1	-0.82506	5.597499	-3.94846	0.00018	0.000647	0.09692	Down
HERC6	0.722582	5.342239	3.947615	0.00018	0.000649	0.094171	Up
TNFAIP6	1.024367	6.027451	3.946871	0.000181	0.000652	0.089442	Up
PTGFR	1.928122	5.104263	3.946964	0.000182	0.000656	0.084773	Up
PPP2R1B	0.60175	6.342003	3.943995	0.000182	0.000656	0.082357	Up
LOC10192	0.526405	4.465754	3.943387	0.000183	0.000658	0.080373	Up
IRS1	-0.65523	9.100363	-3.94197	0.000184	0.000661	0.075318	Down
ZNF682	1.040671	5.018539	3.939578	0.000186	0.000668	0.065682	Up
ANKRD29	0.756509	6.521796	3.938065	0.000186	0.000669	0.063019	Up
ZIC1	-0.84515	7.067565	-3.9373	0.000187	0.000672	0.058671	Down
LHX6	0.663417	6.659969	3.934496	0.000188	0.000677	0.051389	Up
SCN4B	-0.83121	7.687677	-3.93347	0.00019	0.000681	0.045787	Down
LSP1P4	0.618249	4.657611	3.932514	0.00019	0.000681	0.044936	Up
NR5A2	0.989645	3.879756	3.927329	0.000193	0.000694	0.027135	Up
LINP1	-0.80835	7.677893	-3.92684	0.000194	0.000695	0.024633	Down
FCGRT	0.544947	8.302769	3.926033	0.000194	0.000695	0.02384	Up
JAML	0.7138	5.675853	3.923002	0.000196	0.000702	0.013981	Up
SLC8A1	0.516867	5.210067	3.920324	0.000198	0.000708	0.005277	Up
OIP5	0.936394	6.828843	3.919733	0.000199	0.000711	0.001117	Up
LINC0294	-1.49025	4.470679	-3.91976	0.000199	0.000713	-0.0006	Down
APOL4	1.084522	4.609785	3.918729	0.000199	0.000714	-0.00214	Up
RBMXL2	-0.64718	3.001398	-3.91582	0.000201	0.000718	-0.00937	Down
FMC1	-0.52331	9.896837	-3.91225	0.000203	0.000727	-0.02094	Down
NRROS	0.965611	4.890987	3.911785	0.000204	0.000729	-0.02391	Up
EGR1	1.568683	9.714465	3.912176	0.000205	0.000733	-0.02821	Up
OSR2	0.686024	6.193314	3.90846	0.000206	0.000735	-0.03324	Up
CDK18	0.927365	5.008217	3.907357	0.000207	0.000738	-0.0378	Up
LINC0061	-1.2214	1.650455	-3.90681	0.000207	0.000739	-0.0386	Down
LCOR	0.66467	8.094479	3.906392	0.000207	0.00074	-0.03995	Up
RABL2B	0.611079	6.020102	3.905553	0.000208	0.000741	-0.04267	Up
C19orf18	-0.62808	5.411686	-3.90478	0.000209	0.000743	-0.04517	Down
SMIM32	-0.62783	4.529426	-3.90393	0.000209	0.000745	-0.04794	Down
TMEM100	0.959456	5.443254	3.904373	0.000209	0.000745	-0.04812	Up
CSAD	0.713796	6.709159	3.903344	0.00021	0.000746	-0.04983	Up
CELF4	-0.80082	4.061106	-3.9029	0.00021	0.000747	-0.05126	Down
EXOC3L2	-0.53199	6.714822	-3.90223	0.00021	0.000749	-0.05344	Down
DCTPP1	-0.50907	8.08395	-3.90177	0.000211	0.00075	-0.05493	Down
ASPHD2	1.070136	3.662689	3.901208	0.000212	0.000753	-0.05823	Up
MROH6	-0.7299	4.650302	-3.89866	0.000213	0.000757	-0.06499	Down
GGNBP2	0.587027	8.422415	3.897894	0.000214	0.000759	-0.06748	Up
FGF6	-0.65775	5.179003	-3.89747	0.000214	0.00076	-0.06885	Down
BTK	1.099933	4.400364	3.897816	0.000214	0.000761	-0.06993	Up

TMEM87B	0.606824	5.982617	3.894444	0.000216	0.000767	-0.07865	Up
DOK1	-0.52336	5.75084	-3.89309	0.000217	0.000771	-0.08303	Down
BCAN	-0.72974	6.191651	-3.89114	0.000218	0.000775	-0.08933	Down
DNAJC10	0.510388	8.110817	3.89099	0.000219	0.000776	-0.08983	Up
PREX2	0.716657	6.901152	3.890021	0.000219	0.000778	-0.09296	Up
SLC45A3	-0.81739	5.994032	-3.88943	0.00022	0.00078	-0.0952	Down
DENND2D	1.098468	3.870963	3.890089	0.00022	0.00078	-0.0949	Up
MYLK3	-1.41083	7.919843	-3.89153	0.00022	0.000781	-0.09498	Down
CTTNBP2N	0.621004	7.795715	3.887048	0.000222	0.000785	-0.10257	Up
SCARA5	0.794768	8.59781	3.88437	0.000224	0.000794	-0.11339	Up
LRATD2	0.607025	6.663975	3.883057	0.000225	0.000795	-0.11546	Up
DAND5	-0.66422	4.818018	-3.8816	0.000226	0.000799	-0.12018	Down
CASP6	0.592529	8.90131	3.880557	0.000227	0.000802	-0.12354	Up
ZNF662	0.508467	4.426828	3.878552	0.000228	0.000806	-0.13001	Up
MAP3K9	-0.61629	3.108529	-3.87852	0.000228	0.000806	-0.13011	Down
PCSK6	-0.95115	5.591213	-3.87897	0.000228	0.000807	-0.1304	Down
ITGB6	-0.61275	10.13266	-3.87617	0.00023	0.000813	-0.13834	Down
NDRG1	0.508634	8.471778	3.875617	0.00023	0.000814	-0.13948	Up
EXTL1	-0.86491	6.352444	-3.87529	0.000231	0.000816	-0.14193	Down
CWF19L2	0.518035	6.118648	3.873839	0.000232	0.000818	-0.14521	Up
MICU3	-0.56342	6.087147	-3.87291	0.000232	0.00082	-0.14822	Down
GJB1	-0.6044	3.792791	-3.87046	0.000234	0.000827	-0.15609	Down
CSF3	-0.74966	3.994473	-3.86865	0.000236	0.000831	-0.16194	Down
GTPBP2	-0.51646	6.544556	-3.86705	0.000237	0.000835	-0.16709	Down
POLE2	0.911	4.107812	3.867112	0.000237	0.000835	-0.16725	Up
BCKDHA	-0.51381	8.330947	-3.86625	0.000238	0.000837	-0.16967	Down
FCGR1BP	1.439842	3.915777	3.866956	0.000238	0.000839	-0.17097	Up
GTF2H2B	0.733118	6.634311	3.864665	0.000239	0.000841	-0.17477	Up
PRG3	-0.83851	3.594498	-3.86345	0.00024	0.000844	-0.17868	Down
ZNF608	0.717401	6.145425	3.863096	0.00024	0.000845	-0.17982	Up
TRIM32	-0.52974	7.502468	-3.86075	0.000242	0.000852	-0.18738	Down
PARP11	0.600202	5.312846	3.859468	0.000243	0.000855	-0.1915	Up
SLITRK5	1.339848	3.306145	3.859855	0.000244	0.000857	-0.19278	Up
MBTD1	0.506824	9.172408	3.857748	0.000245	0.000859	-0.19703	Up
SLCO4A1	-0.68963	5.083522	-3.8577	0.000245	0.000859	-0.1972	Down
CNKSR3	0.515861	6.096788	3.856444	0.000246	0.000863	-0.20123	Up
YPEL3-DT	-0.58395	4.166674	-3.85521	0.000247	0.000866	-0.20519	Down
NRP1	0.509368	9.262203	3.853712	0.000248	0.000871	-0.21001	Up
ARVCF	-0.61398	5.966339	-3.84898	0.000252	0.000883	-0.22521	Down
LOC10024	0.840758	3.802593	3.848945	0.000252	0.000883	-0.22532	Up
AGPAT2	-0.62094	7.014887	-3.84767	0.000253	0.000887	-0.22941	Down
GTPBP8	-0.55794	9.024473	-3.84736	0.000254	0.000888	-0.2304	Down
LOC1005C	-0.77903	4.078784	-3.84679	0.000254	0.000889	-0.23225	Down
ATP6V1E2	-0.69991	6.239674	-3.84618	0.000255	0.000891	-0.23421	Down
PHKG1	-0.87528	8.338161	-3.84727	0.000255	0.000891	-0.23397	Down
TRA2A	0.775576	8.19788	3.84416	0.000257	0.000899	-0.24273	Up
ALDH1A3	1.034835	7.016535	3.843567	0.000258	0.000902	-0.24608	Up
TSPAN9	0.837896	7.243783	3.842665	0.000258	0.000903	-0.24755	Up
METRN	-0.5908	6.106182	-3.84028	0.00026	0.000908	-0.25311	Down
LOC37444	0.958098	3.566706	3.839407	0.00026	0.00091	-0.25592	Up
OSBPL8	0.655735	9.731392	3.839053	0.000261	0.000912	-0.25823	Up
POMZP3	-1.14656	6.536766	-3.8396	0.000261	0.000913	-0.25878	Down
PGBD5	0.681525	5.758651	3.837969	0.000262	0.000914	-0.26053	Up
ARF6	0.51981	8.725875	3.837375	0.000262	0.000916	-0.26244	Up
VSTM2A-(	0.855499	2.719191	3.835792	0.000264	0.00092	-0.26751	Up
GLI3	0.748056	6.583962	3.834311	0.000265	0.000924	-0.27229	Up
TM4SF18	0.854756	6.770059	3.834214	0.000266	0.000926	-0.27425	Up
TPRN	-0.52606	5.605542	-3.8327	0.000266	0.000928	-0.27741	Down

TRIM2	0.633561	6.503722	3.832452	0.000267	0.000929	-0.27821	Up
SHROOM4	-0.65302	4.495252	-3.83159	0.000267	0.000931	-0.28098	Down
ENG	-0.52957	8.090137	-3.83132	0.000268	0.000932	-0.28183	Down
OLFM1	0.744707	6.749561	3.83005	0.000269	0.000936	-0.28622	Up
LILRA4	-0.86297	2.849291	-3.82621	0.000272	0.000948	-0.29817	Down
SLC39A8	0.775522	4.784904	3.822385	0.000276	0.000959	-0.31041	Up
EIF1AY	3.199027	8.383794	3.82442	0.000278	0.000965	-0.31378	Up
UAP1L1	0.657324	4.540554	3.820488	0.000278	0.000965	-0.31648	Up
YBEY	-0.59824	7.465501	-3.81928	0.000279	0.000968	-0.32033	Down
ZDHHC19	-0.55324	5.136931	-3.81889	0.000279	0.000969	-0.32158	Down
IL7R	0.826793	4.099791	3.81845	0.00028	0.000971	-0.32299	Up
BAIAP2L2	-0.72133	3.914773	-3.81833	0.00028	0.000971	-0.32337	Down
FAIM2	0.635623	4.638682	3.815874	0.000282	0.000978	-0.33121	Up
P2RX5	-0.75423	7.961294	-3.81499	0.000283	0.000983	-0.3357	Down
CYP4V2	-1.17309	4.610409	-3.81407	0.000285	0.000988	-0.34033	Down
LCN10	-0.64879	3.981413	-3.81115	0.000287	0.000993	-0.3463	Down
EMC3	-0.5346	10.62906	-3.81032	0.000287	0.000995	-0.34893	Down
GRIP2	-0.59764	6.98168	-3.8087	0.000289	0.001	-0.35412	Down
COL13A1	-0.57294	4.819969	-3.80749	0.00029	0.001004	-0.35796	Down
DLEU1	0.55389	7.311942	3.806477	0.000291	0.001007	-0.36119	Up
PAGE4	-0.84102	3.925816	-3.80176	0.000296	0.001023	-0.37622	Down
CRIP2	0.84346	7.393623	3.800268	0.000298	0.00103	-0.38296	Up
USP9Y	2.431633	6.809139	3.802871	0.000298	0.001031	-0.38234	Up
C1QTNF12	-0.88444	5.428455	-3.79875	0.000299	0.001033	-0.38669	Down
SCLT1	0.932475	4.541245	3.79665	0.000301	0.00104	-0.39357	Up
C14orf93	-0.56206	5.091567	-3.79599	0.000302	0.001041	-0.3946	Down
SUSD2	0.850921	4.385551	3.795128	0.000302	0.001043	-0.39734	Up
SMTNL2	-0.71256	10.01936	-3.79503	0.000303	0.001046	-0.39963	Down
CIP2A	0.573681	3.793368	3.793184	0.000304	0.001049	-0.40352	Up
SLC16A10	0.603405	8.427423	3.792592	0.000305	0.001051	-0.4054	Up
B3GNT9	0.748208	5.777182	3.790779	0.000307	0.001057	-0.41116	Up
PLEK	1.288686	6.197406	3.791744	0.000307	0.001059	-0.41166	Up
SEMA4D	-0.54085	9.109813	-3.78993	0.000308	0.00106	-0.41385	Down
ZNF765	0.655363	6.889109	3.789084	0.000309	0.001062	-0.41655	Up
ACOX3	-0.61585	5.614624	-3.78904	0.000309	0.001062	-0.41671	Down
DRAM1	0.557902	6.416614	3.787098	0.000311	0.001068	-0.42286	Up
TAL1	-0.60911	6.672581	-3.78506	0.000313	0.001075	-0.42933	Down
UBE2T	-0.57736	7.381388	-3.78477	0.000313	0.001076	-0.43026	Down
RHOF	-0.54433	5.441677	-3.78401	0.000314	0.001078	-0.43266	Down
ZNF571	0.574149	4.447862	3.78287	0.000315	0.001082	-0.43629	Up
OVOS2	1.102206	6.80772	3.784098	0.000315	0.001082	-0.43568	Up
LINC0293	2.270648	3.589446	3.783083	0.000318	0.00109	-0.44179	Up
HBS1L	-0.60508	9.18607	-3.779	0.000319	0.001095	-0.44856	Down
CD52	0.897721	6.726221	3.779196	0.00032	0.001097	-0.44989	Up
LTK	1.617763	3.644174	3.779215	0.000321	0.001099	-0.45155	Up
LINC0116	-0.57023	3.294521	-3.7774	0.000321	0.0011	-0.45365	Down
DHRS3	-0.5449	8.183751	-3.77696	0.000321	0.001102	-0.45504	Down
SLC5A3	0.839397	8.674944	3.777966	0.000322	0.001102	-0.45474	Up
DLG4	-1.42217	3.50196	-3.77746	0.000322	0.001104	-0.45672	Down
ARMCX2	0.582404	8.150044	3.773994	0.000325	0.001111	-0.46444	Up
TGM5	-0.52136	5.619126	-3.77098	0.000328	0.001122	-0.474	Down
CELSR2	-0.57859	5.703114	-3.76778	0.000331	0.001133	-0.48414	Down
CD276	0.60496	6.471003	3.767749	0.000332	0.001133	-0.48423	Up
TAP2	0.659548	6.582013	3.767331	0.000332	0.001135	-0.48555	Up
COL18A1	0.64677	7.626372	3.766752	0.000333	0.001137	-0.48738	Up
SOX9	-0.88711	6.699065	-3.76707	0.000333	0.001138	-0.48829	Down
LRP1B	-1.25964	6.408296	-3.76699	0.000334	0.00114	-0.4901	Down
TRAF3IP2	0.534159	6.205399	3.764403	0.000335	0.001145	-0.49482	Up

OR2A20P	1.0641	3.588062	3.764758	0.000335	0.001145	-0.49512	Up
TFEC	1.517235	3.952224	3.764861	0.000336	0.001148	-0.49697	Up
ELF3	-0.53085	5.054779	-3.76297	0.000337	0.001149	-0.49935	Down
KNTC1	0.700612	4.884227	3.757965	0.000343	0.001168	-0.51517	Up
SPRY4-IT1	-0.59308	4.957074	-3.75719	0.000343	0.001171	-0.51761	Down
TMCO6	0.557619	4.448825	3.755793	0.000345	0.001175	-0.52204	Up
CTRL	-0.56152	3.554192	-3.75553	0.000345	0.001176	-0.52287	Down
SIRT2	-0.57909	10.0585	-3.75389	0.000347	0.001182	-0.52823	Down
MAGEL2	1.657939	3.666063	3.749658	0.000354	0.001205	-0.54605	Up
PDK4	1.431654	10.84939	3.748538	0.000356	0.001211	-0.5509	Up
TADA2B	-0.52424	8.602713	-3.74582	0.000357	0.001212	-0.5535	Down
HOXA4	0.643647	6.445865	3.744955	0.000358	0.001215	-0.55624	Up
CEMP1	-0.87205	3.572895	-3.74461	0.000358	0.001217	-0.55735	Down
CDC20	1.149586	3.533198	3.745264	0.000358	0.001217	-0.55715	Up
TBC1D21	-0.54408	3.924608	-3.74295	0.00036	0.001223	-0.56257	Down
STPG3	-0.87138	5.001247	-3.74234	0.000361	0.001226	-0.56514	Down
PUDP	-0.56481	8.331813	-3.74162	0.000362	0.001227	-0.56674	Down
RAD51B	0.753491	4.043325	3.740458	0.000363	0.001232	-0.57042	Up
RGS19	0.861755	6.034227	3.740653	0.000364	0.001233	-0.57103	Up
MAG	-0.70889	3.633777	-3.73981	0.000364	0.001234	-0.57247	Down
DNAH3	-0.65435	8.277467	-3.73801	0.000366	0.001242	-0.5786	Down
NRCAM	1.413484	4.710108	3.738829	0.000367	0.001243	-0.57892	Up
PURG	0.555194	4.434583	3.736721	0.000368	0.001246	-0.58219	Up
TCP11L2	0.507746	4.729838	3.736359	0.000368	0.001247	-0.58333	Up
APLNR	0.905533	7.777237	3.737559	0.000368	0.001247	-0.58268	Up
MBP	-0.77817	8.64753	-3.73672	0.000369	0.001248	-0.58404	Down
FAM27E3	-0.60189	4.700853	-3.73503	0.00037	0.001252	-0.58751	Down
SMTNL1	-0.75988	10.67687	-3.7351	0.000371	0.001256	-0.59041	Down
ZNF181	0.728379	3.362907	3.733158	0.000372	0.001259	-0.5934	Up
SLC44A5	-1.81661	3.727356	-3.73545	0.000372	0.001259	-0.59206	Down
ACACB	-0.77446	8.569566	-3.7335	0.000373	0.00126	-0.59418	Down
EVI5	0.579806	6.384273	3.730871	0.000375	0.001267	-0.60059	Up
DCLRE1A	-0.60462	5.0778	-3.73081	0.000375	0.001267	-0.6008	Down
COQ10A	-0.60518	10.90699	-3.73101	0.000376	0.001269	-0.60166	Down
SLC25A29	-0.65561	5.944887	-3.73032	0.000376	0.001269	-0.60233	Down
GAB3	0.523034	5.31381	3.726689	0.00038	0.001284	-0.61374	Up
CPLANE1	0.98009	3.858185	3.724808	0.000383	0.001293	-0.62086	Up
ZNF396	-0.94154	4.933258	-3.72464	0.000383	0.001294	-0.62154	Down
NPM3	0.521337	4.652645	3.723676	0.000384	0.001296	-0.62321	Up
ZNRF1	-0.50645	8.979048	-3.72144	0.000387	0.001305	-0.63024	Down
CPB1	0.626388	3.562592	3.721395	0.000387	0.001305	-0.63037	Up
ANKRD36	1.004797	6.700947	3.721043	0.000389	0.001312	-0.63456	Up
TUBB3	0.561371	9.733096	3.719528	0.000389	0.001312	-0.63623	Up
PON3	0.525975	4.649993	3.719009	0.00039	0.001314	-0.63786	Up
CYP2D7	-0.53551	4.387263	-3.71473	0.000396	0.001332	-0.65128	Down
NME7	-1.0529	11.55315	-3.71586	0.000397	0.001337	-0.65349	Down
FCGR1A	1.097707	4.286368	3.714075	0.000397	0.001338	-0.65515	Up
B9D1	-0.8957	4.612798	-3.71319	0.000398	0.00134	-0.65692	Down
SLC25A26	-0.52447	7.75412	-3.71114	0.0004	0.001347	-0.66253	Down
UBQLN4	-0.5501	8.830459	-3.71101	0.000401	0.001347	-0.66293	Down
DPF1	-0.55583	4.992095	-3.70841	0.000404	0.001358	-0.67108	Down
TMEM178	0.885814	5.392349	3.706106	0.000408	0.00137	-0.67937	Up
CPT1B	-0.67883	9.463263	-3.70222	0.000413	0.001387	-0.69193	Down
LINC0109	1.028498	3.311918	3.701036	0.000414	0.001391	-0.69479	Up
BST1	0.754848	4.552485	3.700026	0.000415	0.001394	-0.69733	Up
ANKRD19	-0.56391	3.951313	-3.69812	0.000418	0.001402	-0.70329	Down
PDE4D	-0.65792	9.158519	-3.69749	0.00042	0.001406	-0.70637	Down
IRS2	-0.79349	8.761244	-3.69751	0.00042	0.001408	-0.70711	Down

LOC10537	-0.75095	3.137673	-3.69272	0.000426	0.001425	-0.72016	Down
STEAP3	-0.55537	8.205501	-3.69154	0.000427	0.00143	-0.72385	Down
HR	0.692003	4.727316	3.69103	0.000428	0.001433	-0.72544	Up
DCX	1.603141	3.67962	3.690943	0.00043	0.00144	-0.72961	Up
UACA	-0.58176	10.49476	-3.68954	0.000431	0.001441	-0.73088	Down
ZNF276	-0.51001	4.890336	-3.68907	0.000431	0.001441	-0.73157	Down
MARK2	-0.52621	4.924946	-3.68898	0.000431	0.001441	-0.73184	Down
SLC22A17	0.809034	4.267172	3.685234	0.000436	0.001458	-0.74353	Up
PDCD1LG2	0.50008	3.466382	3.684235	0.000438	0.001463	-0.74664	Up
CALY	-1.0475	3.041502	-3.68409	0.000438	0.001463	-0.74711	Down
ADNP2	0.531203	6.531654	3.682376	0.00044	0.001471	-0.75244	Up
SHCBP1	0.861729	4.316297	3.682348	0.000441	0.001472	-0.75286	Up
HCP5	0.769283	4.948926	3.680166	0.000444	0.001481	-0.75932	Up
G0S2	-1.11214	10.4357	-3.68164	0.000445	0.001484	-0.76027	Down
PTGER3	1.301647	4.571901	3.680059	0.000446	0.001486	-0.76261	Up
DDX11	-0.52556	5.8919	-3.6781	0.000447	0.001489	-0.76577	Down
LOC1005C	-0.53473	3.635376	-3.67758	0.000447	0.001491	-0.76739	Down
ITGA9	0.786738	7.153453	3.675933	0.000451	0.001502	-0.77394	Up
ZNF35	-0.53258	5.52772	-3.67475	0.000452	0.001504	-0.77619	Down
TMSB15B-	1.281194	2.17802	3.671758	0.000456	0.001518	-0.7855	Up
ZNF135	0.754938	3.893808	3.670122	0.000459	0.001525	-0.79059	Up
RNF152	-0.82174	3.970118	-3.669	0.00046	0.00153	-0.7941	Down
PBK	1.492035	3.818524	3.67031	0.00046	0.00153	-0.79327	Up
ITGB1BP1	0.595211	8.958007	3.668872	0.00046	0.001531	-0.79448	Up
MAP4K2	-0.66193	2.478649	-3.6672	0.000463	0.001538	-0.79967	Down
NUPR1	0.542728	8.310281	3.66228	0.000471	0.001562	-0.81496	Up
ANO1	-0.52908	5.876086	-3.66162	0.000472	0.001565	-0.81701	Down
ZNF273	0.622502	5.998754	3.660752	0.000473	0.001569	-0.81971	Up
CCR1	1.004453	6.056067	3.659969	0.000476	0.001577	-0.82451	Up
FGFBP3	0.509881	4.529644	3.657365	0.000478	0.001585	-0.83022	Up
CCL4	1.241871	3.845031	3.658222	0.000479	0.001586	-0.83044	Up
ADGRL2	0.527465	6.973241	3.656993	0.000479	0.001586	-0.83138	Up
MGAT4A	0.574824	6.584028	3.656943	0.000479	0.001586	-0.83153	Up
SLC9A8	-0.66427	5.387008	-3.65594	0.00048	0.00159	-0.83463	Down
FOXP3	-1.02946	4.850285	-3.656	0.000482	0.001593	-0.83616	Down
TIMM8A	-0.72524	6.02092	-3.65414	0.000483	0.001598	-0.84023	Down
XRCC3	-0.76404	3.427683	-3.65274	0.000486	0.001605	-0.84456	Down
PCDHB13	-0.51496	5.279302	-3.65	0.00049	0.001617	-0.85305	Down
F7	-0.64275	4.823245	-3.64947	0.000491	0.00162	-0.85469	Down
MYBPH	2.493855	8.054553	3.652222	0.000492	0.001623	-0.85442	Up
LINC0189(	-0.60277	4.301727	-3.64875	0.000492	0.001623	-0.85692	Down
PPARA	-0.51988	9.187584	-3.64767	0.000494	0.001628	-0.86027	Down
EPHX2	-0.74936	4.859858	-3.64763	0.000494	0.001628	-0.8604	Down
RGS18	1.153741	4.765442	3.648046	0.000495	0.001632	-0.86196	Up
ADIPOQ	2.523518	7.95867	3.648266	0.000498	0.001642	-0.86664	Up
RIMKLB	0.676751	7.225726	3.644769	0.000499	0.001643	-0.86954	Up
WSB1	0.674493	10.30068	3.645045	0.000499	0.001644	-0.87009	Up
OSBPL10	0.626676	6.089552	3.644151	0.000499	0.001645	-0.87117	Up
ABAT	0.563056	5.58487	3.641747	0.000503	0.001657	-0.87861	Up
ZDHHC24	-0.54155	5.020862	-3.64105	0.000505	0.00166	-0.88076	Down
TMEM223	-0.99779	7.563669	-3.64165	0.000506	0.001663	-0.88174	Down
HMX1	-0.97101	3.888211	-3.64003	0.000507	0.001667	-0.88509	Down
ETV1	0.900106	4.704669	3.638981	0.000509	0.001672	-0.88824	Up
ETV5	0.649631	6.450512	3.637723	0.00051	0.001676	-0.89105	Up
CTSG	0.664077	6.318484	3.637702	0.00051	0.001676	-0.89111	Up
APOE	0.889504	8.828543	3.638499	0.000511	0.001678	-0.89147	Up
PPP4C	-0.60605	6.952038	-3.63589	0.000513	0.001685	-0.89671	Down
EGR3	1.278983	5.952876	3.635871	0.000515	0.001691	-0.89984	Up

ZNF780A	0.762822	4.582749	3.634095	0.000516	0.001693	-0.90226	Up
STEAP1	1.011126	5.359333	3.632207	0.000521	0.001707	-0.90976	Up
ZFP82	0.50698	6.289538	3.630442	0.000522	0.001712	-0.91354	Up
ACE	-0.56419	5.455419	-3.63033	0.000523	0.001712	-0.91388	Down
POLD2	-0.52214	8.327026	-3.63019	0.000523	0.001713	-0.91433	Down
E2F7	-0.59613	4.470039	-3.62855	0.000526	0.001721	-0.91939	Down
PSMA3-A:	0.563615	7.441089	3.627997	0.000527	0.001724	-0.92109	Up
DENND6B	-0.51494	4.321915	-3.62715	0.000528	0.001728	-0.9237	Down
NT5C3A	-0.53317	9.591135	-3.62625	0.00053	0.001733	-0.92648	Down
SERF1A	0.510383	8.719219	3.621826	0.000537	0.001756	-0.94012	Up
UFL1	0.665795	8.987132	3.622106	0.000538	0.001757	-0.94041	Up
PANK1	-0.53535	7.308273	-3.62	0.000541	0.001765	-0.94573	Down
HEY2	-0.65779	7.027081	-3.61993	0.000541	0.001766	-0.94595	Down
CEP83	0.657975	5.738065	3.618496	0.000543	0.001774	-0.95038	Up
ARHGAP1	0.870165	4.882501	3.616367	0.000548	0.001787	-0.95772	Up
SLC1A7	-1.42663	5.900603	-3.61653	0.00055	0.001795	-0.96091	Down
MTFR2	0.66635	3.585524	3.613034	0.000553	0.001803	-0.96719	Up
TCTN2	-0.70172	6.1538	-3.60979	0.000559	0.001822	-0.97716	Down
KDM5D	2.976795	7.118523	3.612791	0.00056	0.001824	-0.97584	Up
LINC0283	0.883453	2.986762	3.609016	0.00056	0.001826	-0.97955	Up
ZNF367	0.87021	4.490897	3.609242	0.00056	0.001826	-0.97955	Up
ACSL6	-0.55041	3.757681	-3.60852	0.000561	0.001828	-0.98107	Down
TMEM231	0.614819	4.984357	3.607747	0.000563	0.001832	-0.98345	Up
RUFY2	0.662319	7.794998	3.607211	0.000564	0.001837	-0.98567	Up
ST6GALN/	-0.53634	6.711098	-3.60458	0.000569	0.001851	-0.99319	Down
CDC42EP5	0.855355	6.096478	3.6037	0.000571	0.001858	-0.9972	Up
SLC15A3	0.643441	5.384915	3.602716	0.000572	0.001861	-0.9989	Up
FGF7	0.837978	5.103376	3.599171	0.000579	0.001883	-1.01035	Up
EIF5-DT	0.68509	5.122011	3.595855	0.000585	0.001901	-1.01995	Up
LINC0177	-0.51621	4.272675	-3.59326	0.00059	0.001916	-1.0279	Down
SH3BP5-A	-0.55204	6.548881	-3.59226	0.000592	0.001921	-1.03096	Down
HSPA1B	-1.02231	8.078938	-3.59314	0.000592	0.001923	-1.03103	Down
HDX	0.822741	3.369363	3.5913	0.000594	0.001927	-1.03392	Up
CBLN4	1.102767	3.660121	3.591469	0.000595	0.001929	-1.03499	Up
SLC39A11	0.561858	5.131781	3.589417	0.000597	0.001937	-1.03968	Up
GAS1RR	0.526099	4.132454	3.588182	0.0006	0.001944	-1.04347	Up
FGD3	0.591671	4.825661	3.587753	0.000601	0.001947	-1.04478	Up
SEC22B	0.57429	9.742693	3.587708	0.000601	0.001947	-1.04521	Up
EFCAB11	0.54341	7.470243	3.586422	0.000603	0.001954	-1.04885	Up
UBALD2	-0.61434	7.675598	-3.58618	0.000604	0.001955	-1.0496	Down
ZNF334	0.609315	3.061316	3.585144	0.000606	0.001961	-1.05277	Up
TUBGCP6	-0.55229	4.960163	-3.58261	0.000611	0.001976	-1.06051	Down
TNFRSF12	-1.0438	7.154095	-3.58331	0.000612	0.001979	-1.06103	Down
ACSM5	-0.78758	6.423574	-3.58075	0.000615	0.001989	-1.06722	Down
CXCR5	-0.79616	5.432543	-3.58024	0.000616	0.001989	-1.06807	Down
CATSPER2	-0.53814	4.531507	-3.57696	0.000622	0.002009	-1.07778	Down
MYOM3	-0.56972	9.401825	-3.57472	0.000627	0.002022	-1.08463	Down
PFKFB3	0.871325	9.004779	3.575171	0.000628	0.002025	-1.08589	Up
PLCB1	-0.58532	8.013176	-3.57289	0.00063	0.002032	-1.09023	Down
LIPF	-0.80488	4.624747	-3.57277	0.000631	0.002033	-1.0906	Down
TMEM107	0.508879	5.13397	3.572175	0.000632	0.002036	-1.0924	Up
SLC11A1	-0.60352	5.633265	-3.57121	0.000634	0.002042	-1.09535	Down
NR2F1	0.90275	3.799229	3.571228	0.000634	0.002043	-1.09584	Up
ARHGAP4	-0.76712	5.595954	-3.56998	0.000636	0.002049	-1.09912	Down
LINC0206	-1.1131	7.256894	-3.5711	0.000637	0.002049	-1.0986	Down
PARVG	0.719382	4.296059	3.568575	0.000639	0.002056	-1.10339	Up
KCNN1	-0.51581	5.337155	-3.56715	0.000642	0.002065	-1.10774	Down
PGR	-0.81183	4.964896	-3.56727	0.000642	0.002065	-1.10766	Down

HOXA5	-0.66229	6.545689	-3.56659	0.000643	0.002068	-1.10945	Down
LIPE-AS1	-0.64502	4.94835	-3.56656	0.000643	0.002068	-1.10954	Down
TNFSF8	1.040116	4.437579	3.565977	0.000646	0.002075	-1.11286	Up
KRBOX5	0.579735	5.967445	3.565186	0.000646	0.002076	-1.11372	Up
MYO7B	-0.7781	5.949202	-3.56144	0.000655	0.002101	-1.12566	Down
CKS2	0.846548	5.812548	3.561364	0.000655	0.002102	-1.12644	Up
ZC3H12D	1.054171	3.64403	3.561339	0.000656	0.002104	-1.12698	Up
LOC10537	0.894953	4.997574	3.558526	0.000661	0.002121	-1.13514	Up
C2CD4B	-0.71951	3.132561	-3.55632	0.000665	0.002132	-1.14073	Down
ZNF141	0.646509	4.51839	3.553982	0.00067	0.002147	-1.14784	Up
NPAS2	-0.65029	5.274998	-3.55336	0.000672	0.002151	-1.14973	Down
FLYWCH1	-0.52423	7.139055	-3.55299	0.000672	0.002153	-1.15086	Down
SLC25A16	-0.75067	8.627807	-3.55247	0.000675	0.002161	-1.15396	Down
CHI3L1	2.243831	4.918169	3.550384	0.000683	0.002185	-1.16359	Up
NPHP1	-0.6639	6.301436	-3.54662	0.000686	0.002195	-1.17022	Down
PMAIP1	0.945528	5.094863	3.546632	0.000688	0.002199	-1.17169	Up
AJM1	-0.77819	4.570984	-3.54424	0.000692	0.00221	-1.17745	Down
CLSTN3	-0.55429	4.383035	-3.54404	0.000692	0.002211	-1.17805	Down
PCDH17	0.628708	6.395207	3.540848	0.000699	0.002232	-1.18773	Up
TNC	1.820613	7.413369	3.541694	0.000702	0.00224	-1.18993	Up
LST1	0.797424	6.112817	3.536378	0.00071	0.002265	-1.20221	Up
SMAD5	0.553409	8.697692	3.535755	0.000711	0.002266	-1.20317	Up
PLCXD1	0.541895	5.789073	3.535741	0.000711	0.002266	-1.20321	Up
NUDT9P1	-0.71035	4.035086	-3.53429	0.000714	0.002276	-1.20761	Down
SESTD1	0.53614	7.940521	3.532744	0.000718	0.002287	-1.21229	Up
SLIT2	0.845718	7.304682	3.533115	0.000719	0.00229	-1.21305	Up
PDLIM4	0.713103	6.191443	3.531759	0.00072	0.002293	-1.21528	Up
SHPRH	0.587724	7.008131	3.529341	0.000726	0.00231	-1.2226	Up
CEP97	0.594714	6.280133	3.528488	0.000728	0.002316	-1.22518	Up
CHD3	0.638108	5.872538	3.527945	0.000729	0.002319	-1.22682	Up
PELI2	0.616813	5.873983	3.526973	0.000731	0.002326	-1.22976	Up
ZNF280B	0.872789	4.164983	3.527024	0.000732	0.002327	-1.2303	Up
VWA1	-0.50188	5.337353	-3.5257	0.000734	0.002334	-1.23361	Down
PRKCQ-A:	-0.56424	8.52849	-3.52494	0.000736	0.002339	-1.2359	Down
HOATZ	-0.53361	5.525258	-3.52423	0.000738	0.002344	-1.23805	Down
CNGA4	-0.72239	3.065326	-3.5201	0.000748	0.002373	-1.25054	Down
KERA	1.799209	4.601102	3.519882	0.000753	0.002387	-1.25585	Up
ARK2C	0.60524	4.66363	3.516217	0.000757	0.002399	-1.26227	Up
ANGPTL4	1.345228	4.928773	3.514932	0.000763	0.002417	-1.2689	Up
LINC0095:	-0.52072	4.973956	-3.50947	0.000774	0.002448	-1.28263	Down
LIAS	-0.60226	7.313904	-3.50912	0.000774	0.002451	-1.28368	Down
MUC20	-0.66973	4.865579	-3.50808	0.000777	0.002458	-1.28682	Down
TOX	0.846052	3.80463	3.507805	0.000778	0.00246	-1.28778	Up
CORO1A	0.707673	5.480857	3.506275	0.000782	0.002471	-1.29225	Up
TLN2	-0.51228	7.055742	-3.50584	0.000783	0.002474	-1.29355	Down
TRAF4	1.157641	4.547326	3.506149	0.000785	0.00248	-1.29507	Up
MYBL1	-0.68343	4.854971	-3.5042	0.000787	0.002486	-1.29849	Down
BBC3	-0.66003	5.747763	-3.50201	0.000792	0.002502	-1.30511	Down
GSTM2	-0.51867	9.646834	-3.49974	0.000798	0.002518	-1.31193	Down
USP6NL	0.797159	4.28938	3.498895	0.0008	0.002523	-1.31447	Up
REG3A	-0.71634	3.89167	-3.49531	0.00081	0.00255	-1.32525	Down
POLK	0.599635	8.37962	3.494552	0.000812	0.002556	-1.32777	Up
NCEH1	0.661198	6.459597	3.492322	0.000817	0.002573	-1.33423	Up
B4GALNT:	-0.72962	4.820938	-3.49134	0.00082	0.00258	-1.33719	Down
SLC2A3	1.018612	5.836537	3.492093	0.000821	0.002582	-1.33731	Up
TRAC	0.578905	6.68124	3.490566	0.000822	0.002585	-1.3395	Up
CCDC120	-0.53178	3.3167	-3.49015	0.000823	0.002589	-1.34074	Down
TOB2	-0.50678	8.651449	-3.48952	0.000825	0.002593	-1.34263	Down

DPEP3	-0.83466	3.098425	-3.48912	0.000826	0.002596	-1.34384	Down
RBAKDN	-0.91022	3.719059	-3.48841	0.000828	0.002603	-1.34663	Down
INTS6L	0.836436	4.902694	3.487476	0.000831	0.00261	-1.34945	Up
MUC2	-0.62006	4.97404	-3.48679	0.000832	0.002613	-1.35085	Down
HTN1	1.602861	3.121505	3.487519	0.000833	0.002615	-1.35103	Up
PEBP4	-0.78883	10.90094	-3.48626	0.000836	0.002624	-1.35479	Down
B3GALT2	1.016367	3.328246	3.485146	0.000837	0.002627	-1.35668	Up
HSD11B1	-1.0082	4.580537	-3.48426	0.00084	0.002636	-1.35984	Down
DPY19L3-	-0.61008	3.89239	-3.48334	0.000841	0.002638	-1.36119	Down
CHL1	1.098676	3.263456	3.483774	0.000841	0.002638	-1.36104	Up
ADD3-AS:	-0.5115	5.01385	-3.48316	0.000842	0.002639	-1.36173	Down
ZCCHC7	0.616476	7.453887	3.482987	0.000842	0.00264	-1.36224	Up
LOC10013	-0.61864	4.814403	-3.48291	0.000842	0.00264	-1.36246	Down
LOH12CR:	0.541899	4.426835	3.4821	0.000845	0.002647	-1.3649	Up
STRC	-0.67809	4.041341	-3.48122	0.000847	0.002654	-1.36752	Down
AMER1	-0.93983	3.746391	-3.4789	0.000854	0.002676	-1.37545	Down
TMEM106	0.629407	6.238398	3.477996	0.000856	0.002679	-1.3772	Up
MATN4	-0.58851	3.905424	-3.47768	0.000857	0.002682	-1.37815	Down
OAF	0.795743	7.295286	3.478138	0.000857	0.002683	-1.37818	Up
RTP4	0.626887	4.554864	3.476768	0.000859	0.002688	-1.38088	Up
ZFYVE9	-0.64535	6.384721	-3.47403	0.000867	0.00271	-1.38908	Down
IFI6	1.193626	6.615281	3.474533	0.000869	0.002715	-1.39051	Up
LCDR	0.671535	4.963719	3.471594	0.000873	0.002729	-1.39636	Up
RBM19	-0.54443	5.193433	-3.47139	0.000874	0.00273	-1.39696	Down
S100A8	-1.69861	6.834677	-3.46901	0.000886	0.002766	-1.40848	Down
GALNT12	0.676676	4.965169	3.46628	0.000888	0.002772	-1.41225	Up
SOD2	-0.5659	10.09101	-3.46568	0.000891	0.00278	-1.41464	Down
MICAL1	0.999932	6.278328	3.465426	0.000894	0.002787	-1.41713	Up
GSAP	0.530912	6.837707	3.462887	0.000898	0.0028	-1.42238	Up
AGMAT	-0.8455	7.72384	-3.46264	0.000902	0.002809	-1.42546	Down
WNT2B	0.543454	4.537408	3.461246	0.000903	0.002812	-1.42728	Up
GCA	0.636714	5.864881	3.460513	0.000905	0.002817	-1.42947	Up
STAC3	-0.66525	10.4627	-3.46014	0.000908	0.002826	-1.43204	Down
CEP55	0.986016	3.015057	3.45919	0.000909	0.002827	-1.43342	Up
SNCA	-0.53723	6.530398	-3.45846	0.000911	0.002833	-1.43558	Down
ZNF814	-0.70562	5.658478	-3.45742	0.000914	0.002841	-1.4387	Down
TTTY11	-0.53089	4.025679	-3.45522	0.00092	0.00286	-1.44525	Down
ADAMTS9	0.691153	6.358378	3.455156	0.00092	0.00286	-1.44547	Up
ZNF697	-0.52142	5.309953	-3.45406	0.000924	0.002869	-1.44872	Down
VNN1	0.866267	3.442838	3.453183	0.000926	0.002876	-1.45133	Up
MYL7	-0.8099	6.43691	-3.45148	0.000933	0.002896	-1.45768	Down
DYNC2LI1	0.540661	6.867812	3.45079	0.000933	0.002897	-1.45846	Up
CMBL	-0.5015	11.47674	-3.45023	0.000936	0.002903	-1.46077	Down
LIN28B-A:	-0.50409	4.351964	-3.44867	0.00094	0.002913	-1.46478	Down
TNFRSF10	-0.5196	4.35786	-3.44405	0.000953	0.002953	-1.47853	Down
FCGR3B	-0.95422	5.179429	-3.44435	0.000954	0.002956	-1.47897	Down
PGM2L1	-0.65542	7.940442	-3.44386	0.000955	0.002958	-1.47997	Down
BMX	0.559046	3.501478	3.443196	0.000956	0.00296	-1.48106	Up
CSPG5	0.525391	4.08075	3.443116	0.000956	0.00296	-1.4813	Up
LOC10192	0.738835	2.781665	3.443014	0.000957	0.002961	-1.4816	Up
TRIM62	-0.59063	4.364647	-3.44271	0.000957	0.002963	-1.48252	Down
HTR2B	0.634358	3.918444	3.436784	0.000976	0.003016	-1.50012	Up
SYT5	-0.56966	3.784755	-3.43617	0.000978	0.003022	-1.50194	Down
CD180	0.931183	3.550658	3.435973	0.000979	0.003026	-1.50317	Up
CITED2	0.653104	9.28231	3.435773	0.000981	0.00303	-1.50434	Up
MRGPRF	0.765609	5.786215	3.435151	0.000981	0.003032	-1.50544	Up
SEPT5-GP	-0.67463	4.534919	-3.43464	0.000982	0.003035	-1.50649	Down
CUTALP	0.533795	6.583784	3.4316	0.000992	0.003061	-1.51551	Up

LINC0122	0.582756	3.680339	3.430341	0.000996	0.003073	-1.51924	Up
USP53	-0.54874	8.549799	-3.42902	0.001	0.003085	-1.52315	Down
UBA6	0.588162	7.111921	3.428204	0.001003	0.003092	-1.52558	Up
BORCS6	-0.67799	5.046427	-3.42759	0.001005	0.003097	-1.52741	Down
GDPD2	-0.58185	4.28278	-3.42609	0.001009	0.00311	-1.53185	Down
EDEM3	0.509608	8.340187	3.426067	0.001009	0.00311	-1.53191	Up
LINS1	0.508409	6.65184	3.425732	0.00101	0.003113	-1.5329	Up
GK	-0.77048	4.89716	-3.42565	0.001011	0.003114	-1.5333	Down
PPFIBP2	0.602017	5.415789	3.425539	0.001011	0.003114	-1.53348	Up
KRT74	-0.55511	3.253974	-3.42397	0.001016	0.003128	-1.53811	Down
AVPI1	0.596408	5.793187	3.423409	0.001018	0.003134	-1.53979	Up
TONSL-AS	-1.05229	3.938653	-3.42293	0.001022	0.003144	-1.54278	Down
SMCR5	0.812034	3.808047	3.421681	0.001023	0.003149	-1.5449	Up
PMEPA1	-0.64437	7.452908	-3.4203	0.001029	0.003164	-1.54946	Down
B3GNT5	0.78087	8.063646	3.419064	0.001035	0.003182	-1.55454	Up
LXN	0.556812	6.680974	3.417598	0.001037	0.003188	-1.55699	Up
FTX	-0.76022	7.935222	-3.41603	0.001044	0.003207	-1.56292	Down
HDAC7	-0.53612	6.996805	-3.4142	0.001048	0.003218	-1.56704	Down
IRAK2	-0.54915	5.929111	-3.41271	0.001053	0.003232	-1.57142	Down
CPEB1	-0.59166	4.664112	-3.41225	0.001054	0.003237	-1.5728	Down
TBXAS1	0.771711	4.654874	3.412215	0.001055	0.003237	-1.57303	Up
TMEM178	0.658121	6.902974	3.410693	0.00106	0.003251	-1.57764	Up
GFRA4	-0.81981	3.45859	-3.40932	0.001064	0.003263	-1.58145	Down
NAT14	0.808326	4.263826	3.408507	0.001068	0.003272	-1.58421	Up
GPR82	0.675276	3.248974	3.407499	0.00107	0.003281	-1.58683	Up
ZNF296	-0.61437	3.274902	-3.40534	0.001078	0.003299	-1.5932	Down
SHB	-0.71992	7.523253	-3.40566	0.001079	0.003301	-1.59353	Down
MARCHF2	-0.54929	8.204632	-3.40459	0.00108	0.003305	-1.59542	Down
CEP170P1	-0.64453	5.141381	-3.40133	0.001091	0.003337	-1.60503	Down
XAGE1B	-0.61987	4.06693	-3.40063	0.001094	0.003343	-1.60709	Down
NPB	-0.54482	6.678803	-3.40009	0.001096	0.003348	-1.60869	Down
BMS1P2	-0.51656	6.586806	-3.39977	0.001097	0.003351	-1.60961	Down
HBG2	-1.39762	7.964963	-3.40126	0.001098	0.003354	-1.60929	Down
ZNF788P	0.772249	3.18531	3.39654	0.001108	0.00338	-1.61914	Up
TUBE1	0.572019	7.075274	3.396478	0.001108	0.00338	-1.61932	Up
NKX2-8	-0.85545	3.095637	-3.39039	0.00113	0.003442	-1.63724	Down
ZFYVE28	-0.55026	4.018771	-3.38996	0.001131	0.003446	-1.6385	Down
PTPRS	0.522964	6.672076	3.388074	0.001138	0.003465	-1.64404	Up
KIAA2012	-0.56647	5.160918	-3.38594	0.001146	0.003486	-1.6503	Down
MTBP	0.716853	3.397136	3.385109	0.001149	0.003493	-1.65276	Up
AKAP4	-0.67803	2.847682	-3.38494	0.001149	0.003494	-1.65324	Down
SNCG	-0.55519	5.552524	-3.38366	0.001154	0.003508	-1.65702	Down
LENG8	-0.53578	6.212019	-3.3818	0.001161	0.003526	-1.66246	Down
TBX4	-0.99419	4.49737	-3.38094	0.001166	0.003541	-1.66636	Down
GLRX	0.546871	9.61291	3.379772	0.001168	0.003547	-1.66866	Up
FAM118B	-0.5013	8.075402	-3.37858	0.001172	0.003558	-1.67192	Down
S100B	1.546041	4.092565	3.379172	0.001177	0.003571	-1.67413	Up
KLHL10	-0.8039	4.507487	-3.37684	0.00118	0.003578	-1.67745	Down
DNALI1	0.801791	4.56115	3.375707	0.001184	0.003589	-1.68078	Up
RNF182	0.742299	3.53491	3.375432	0.001184	0.003589	-1.68115	Up
MRPL20-A	-0.58857	6.225484	-3.37425	0.001189	0.003602	-1.68463	Down
ADAD2	-0.73641	3.885047	-3.37416	0.001189	0.003602	-1.68488	Down
GPATCH2	0.650536	5.578629	3.372421	0.001195	0.00362	-1.68998	Up
BGLT3	-0.55485	4.074503	-3.37132	0.001199	0.003631	-1.69319	Down
CLDN11	0.865796	4.979452	3.371408	0.001201	0.003634	-1.69396	Up
PKM	-0.60268	11.37063	-3.37105	0.001203	0.003641	-1.6955	Down
GJD3	-0.59542	6.416436	-3.36613	0.001219	0.003686	-1.70839	Down
ESRRG	-0.73604	7.939547	-3.36607	0.001222	0.003692	-1.7098	Down

SORL1	0.706384	6.200436	3.364982	0.001224	0.003697	-1.71205	Up
LINC0047	-0.50244	3.568969	-3.36353	0.001229	0.003711	-1.71601	Down
CTNNA3	-1.16816	8.54523	-3.36291	0.001238	0.003735	-1.7217	Down
ELOVL4	0.812711	3.110151	3.358732	0.001248	0.003761	-1.73002	Up
RETSAT	0.506764	8.928851	3.358301	0.001249	0.003765	-1.73128	Up
HBD	-1.45393	5.61147	-3.35951	0.001252	0.003771	-1.73159	Down
SCN2B	-0.92974	5.724243	-3.35773	0.001254	0.003778	-1.73436	Down
FGFBP2	1.05484	4.407319	3.357696	0.001255	0.003781	-1.73509	Up
SHROOM	-0.58278	5.281726	-3.35397	0.001266	0.003812	-1.74392	Down
SOX13	-0.57375	6.260901	-3.35287	0.001271	0.003824	-1.74712	Down
SMIM3	0.504316	7.318838	3.351888	0.001275	0.003835	-1.75	Up
PDX1	-0.54397	3.89305	-3.35168	0.001276	0.003837	-1.7506	Down
THNSL2	-0.87631	7.648793	-3.35126	0.001281	0.003852	-1.75385	Down
ZNF415	-0.6317	5.179691	-3.35019	0.001281	0.003853	-1.75496	Down
MINCR	-0.50186	5.513347	-3.34937	0.001285	0.003862	-1.75734	Down
TMEM132	1.253931	4.833564	3.350249	0.001285	0.003863	-1.75696	Up
ITGA10	-0.58253	5.086363	-3.3489	0.001287	0.003865	-1.7587	Down
B4GALNT	-0.58103	3.392801	-3.34857	0.001288	0.003867	-1.75966	Down
FBXO24	-0.57984	3.755274	-3.34606	0.001298	0.003896	-1.76698	Down
AGPAT4	0.773752	3.75246	3.345471	0.001301	0.0039	-1.7687	Up
LOC1001	-0.71517	8.405993	-3.34562	0.001302	0.003904	-1.76947	Down
ZDHHC13	0.852669	4.352173	3.342452	0.001314	0.003938	-1.77832	Up
KRT33A	-1.28121	2.264171	-3.34199	0.001316	0.003942	-1.77952	Down
MED25	-0.56587	8.094977	-3.34156	0.001316	0.003943	-1.78009	Down
INHBA	-1.16948	4.072505	-3.34173	0.00132	0.00395	-1.7816	Down
ZNF624	0.754456	3.664651	3.340421	0.001321	0.003953	-1.7834	Up
SMPDL3A	-0.53307	8.947084	-3.3404	0.001321	0.003953	-1.78345	Down
ABHD1	-0.59084	4.23625	-3.33999	0.001323	0.003956	-1.78466	Down
CPZ	0.536085	4.290797	3.336703	0.001337	0.003994	-1.79421	Up
LRRC7	-0.68516	4.931281	-3.33611	0.001339	0.004	-1.79592	Down
ALOX5AP	0.784276	6.928014	3.335875	0.001342	0.004009	-1.7978	Up
LINC0301	-0.60618	4.840149	-3.3325	0.001354	0.004041	-1.80643	Down
P2RY13	0.825195	4.050256	3.331876	0.001358	0.004051	-1.80873	Up
CSMD2	-0.72368	4.517011	-3.32894	0.001369	0.004084	-1.81677	Down
ZNF674-A	0.716142	3.959509	3.328381	0.001372	0.00409	-1.81838	Up
MRAP2	0.915266	5.58972	3.32792	0.001376	0.0041	-1.82089	Up
CLUL1	0.656628	2.381788	3.325337	0.001385	0.004123	-1.82721	Up
OCA2	0.7126	3.602873	3.32469	0.001387	0.004131	-1.82908	Up
HDHD3	-0.64795	3.926104	-3.32359	0.001392	0.004144	-1.83227	Down
PLA2G12A	-0.71641	8.105566	-3.32361	0.001395	0.00415	-1.83337	Down
MLLT11	-1.09912	9.420142	-3.32358	0.0014	0.004163	-1.836	Down
NRAP	-1.10326	11.27152	-3.32358	0.0014	0.004163	-1.836	Down
PTGER2	0.835095	3.683079	3.320827	0.001405	0.004177	-1.8405	Up
KCNH6	-1.01089	4.67532	-3.32062	0.001409	0.004187	-1.84271	Down
BORA	0.755522	4.186163	3.318938	0.001413	0.004196	-1.84575	Up
ZNF385C	-0.61973	4.190055	-3.31866	0.001414	0.004199	-1.84654	Down
LINC0011	-0.62045	3.209964	-3.31727	0.00142	0.004215	-1.85059	Down
AEBP1	1.130664	7.018413	3.314713	0.001437	0.004264	-1.86082	Up
MYRF	-0.70954	3.316677	-3.31024	0.001451	0.004303	-1.87092	Down
NHIP	-0.56286	3.463648	-3.309	0.001457	0.004318	-1.87449	Down
CNTN3	1.273125	3.667878	3.307039	0.00147	0.004355	-1.88208	Up
ECM1	0.503598	6.673199	3.305087	0.001475	0.004367	-1.8858	Up
IGLON5	-0.70228	2.919841	-3.30487	0.001476	0.004369	-1.88643	Down
BAAT	-0.61868	3.545835	-3.30474	0.001476	0.00437	-1.8868	Down
SBSN	-0.64376	4.78049	-3.30464	0.001477	0.00437	-1.88707	Down
PCDHB6	0.662801	3.670678	3.303708	0.001481	0.004381	-1.88978	Up
S1PR3	0.610532	6.37336	3.30265	0.001486	0.004394	-1.89283	Up
FGF4	-0.77196	6.233046	-3.3009	0.001496	0.00442	-1.89879	Down

KRT37	-0.60611	3.731033	-3.29605	0.001516	0.004478	-1.91186	Down
TAFA5	-0.97312	2.650622	-3.29575	0.001518	0.004482	-1.91271	Down
CEBPA	0.843866	6.547062	3.294822	0.001525	0.004502	-1.91674	Up
DLG3-AS1	-0.60306	5.02634	-3.289	0.00155	0.004566	-1.93216	Down
PRKCD	0.526878	4.249367	3.286924	0.00156	0.004592	-1.93812	Up
HBG1	-1.42853	7.513139	-3.28804	0.001562	0.0046	-1.93843	Down
GSTM1	-0.52206	9.159787	-3.28564	0.001566	0.004607	-1.94181	Down
ROBO2	0.888459	2.833422	3.285002	0.001569	0.004616	-1.94364	Up
HPCA	-0.78146	5.333442	-3.28489	0.001571	0.004619	-1.94461	Down
NSUN6	0.639907	7.206172	3.283926	0.001575	0.00463	-1.94723	Up
ZNF93	0.705923	6.662503	3.283265	0.001579	0.004641	-1.94936	Up
ALKBH8	-0.5243	5.700677	-3.2817	0.001585	0.004656	-1.95312	Down
WNT9A	0.81952	5.585421	3.279991	0.001596	0.004685	-1.95894	Up
TFRC	0.777135	11.45118	3.278541	0.001606	0.004712	-1.96444	Up
MESP1	-0.50698	6.026992	-3.27744	0.001606	0.004712	-1.96534	Down
CEP290	0.501543	5.814893	3.274299	0.001622	0.004754	-1.97436	Up
TFR2	-0.55746	4.049823	-3.27401	0.001623	0.004757	-1.97518	Down
ATP2A3	-0.65613	5.152064	-3.27086	0.001639	0.004802	-1.98422	Down
KLRB1	0.707693	4.213171	3.270669	0.00164	0.004803	-1.98476	Up
SCG2	1.050812	4.394397	3.269738	0.001649	0.004828	-1.98927	Up
UNC5B-A:	-0.57417	3.138061	-3.26854	0.001651	0.004832	-1.99086	Down
ASCL2	-1.10479	4.381326	-3.26823	0.001657	0.004848	-1.99357	Down
KLK1	-0.5704	4.191962	-3.26692	0.001659	0.004854	-1.9955	Down
C1orf115	-0.55238	7.590985	-3.26261	0.001681	0.004916	-2.00782	Down
MEX3B	0.623355	3.339024	3.260965	0.00169	0.004939	-2.01253	Up
SNHG14	-0.71165	5.522977	-3.25881	0.001701	0.00497	-2.01885	Down
LINC0110	-0.61911	5.342888	-3.25796	0.001705	0.004981	-2.02112	Down
CFAP61	-0.58152	3.994213	-3.25771	0.001707	0.004984	-2.02184	Down
POU6F2	-0.52206	4.011506	-3.25657	0.001713	0.004999	-2.0251	Down
H1-9P	-0.54083	4.173713	-3.25451	0.001723	0.005028	-2.03097	Down
SLC16A7	-0.92167	7.238786	-3.25328	0.001735	0.005058	-2.03627	Down
HEYL	0.602594	6.765382	3.250896	0.001743	0.005081	-2.04127	Up
SLC9A3-A	0.773069	4.325962	3.25054	0.001745	0.005088	-2.04264	Up
EFNA4	0.820714	3.706938	3.250215	0.001747	0.005092	-2.04353	Up
C1orf162	0.550255	6.003831	3.247829	0.001759	0.005124	-2.05002	Up
FAM13C	0.508771	9.2447	3.247633	0.00176	0.005126	-2.05058	Up
SHPK	0.577059	4.393129	3.240783	0.001798	0.005228	-2.07008	Up
VRTN	-0.68178	4.085417	-3.23489	0.00183	0.005316	-2.08684	Down
NR1I3	-0.61402	4.177345	-3.2348	0.001831	0.005317	-2.08708	Down
MYOC	-1.37274	7.349357	-3.23627	0.001832	0.005318	-2.08622	Down
HCST	0.609149	5.854944	3.234421	0.001833	0.005322	-2.08816	Up
PPM1H	0.613155	5.202923	3.232337	0.001845	0.005353	-2.09408	Up
RNF180	-0.50333	4.744482	-3.23031	0.001856	0.005384	-2.09982	Down
CDC37P1	-0.9027	2.572485	-3.22895	0.001864	0.005402	-2.10368	Down
LY6K	-0.97967	5.608579	-3.2271	0.001879	0.005444	-2.11069	Down
IGHV3-47	-0.92949	3.42665	-3.2241	0.001894	0.005481	-2.11814	Down
MYO1D	0.754852	5.794476	3.223149	0.001899	0.005496	-2.12081	Up
TLCD2	0.637094	6.127858	3.222724	0.0019	0.005497	-2.12135	Up
CHST8	-0.97459	5.156204	-3.22169	0.00191	0.005525	-2.126	Down
SH3BGRL3	0.586896	8.348978	3.220557	0.001914	0.005533	-2.12798	Up
MT1E	0.512661	9.299551	3.218942	0.001922	0.005552	-2.13206	Up
SCUBE3	-0.53134	4.833505	-3.21751	0.00193	0.005574	-2.13611	Down
HTR7P1	0.765645	3.893748	3.214101	0.00195	0.005629	-2.14585	Up
ITGB3BP	0.535076	7.054016	3.213298	0.001955	0.005641	-2.14802	Up
AFAP1L2	0.593829	4.063179	3.211614	0.001965	0.005669	-2.15278	Up
IL2RB	0.591491	4.604052	3.211195	0.001967	0.005675	-2.15396	Up
TSPAN8	-1.27275	8.126322	-3.20996	0.001984	0.005716	-2.16066	Down
PDIA4	0.52875	7.098898	3.207397	0.00199	0.005732	-2.16469	Up

DUSP5	0.621755	4.160317	3.205217	0.002004	0.005767	-2.17084	Up
GPX3	-0.66676	10.84288	-3.20542	0.002007	0.005776	-2.17196	Down
LOC10028	-0.60737	3.266421	-3.20457	0.002007	0.005776	-2.17266	Down
CYP39A1	-0.52813	4.109538	-3.20439	0.002009	0.005779	-2.17319	Down
FAXDC2	0.511151	7.532576	3.204203	0.00201	0.005781	-2.1737	Up
CORO2B	0.537814	5.564972	3.204047	0.002011	0.005783	-2.17414	Up
UPK3A	-1.61153	4.854223	-3.20461	0.002017	0.005799	-2.17573	Down
RGS11	-0.56535	5.709608	-3.20235	0.002021	0.00581	-2.17893	Down
TLX2	-0.6582	3.724499	-3.20073	0.002031	0.005835	-2.18349	Down
LRRC2-AS	-0.90844	3.239943	-3.20013	0.002036	0.005847	-2.18552	Down
MYL3	-0.63219	12.31018	-3.19758	0.002056	0.005898	-2.19404	Down
BUB1B	1.130354	3.231957	3.196217	0.002063	0.005917	-2.19739	Up
ALPK2	-0.52726	9.438585	-3.19506	0.002067	0.005925	-2.19974	Down
LINC0114:	0.720997	2.473029	3.190184	0.002097	0.006005	-2.21318	Up
FBP2	-1.16423	10.03827	-3.18982	0.002109	0.006036	-2.21733	Down
SLFN5	0.730014	9.045384	3.188658	0.002112	0.006041	-2.21913	Up
DNM1	0.843498	5.019273	3.18828	0.002112	0.006041	-2.21952	Up
RCN3	0.725833	6.268925	3.18684	0.002121	0.006061	-2.22327	Up
KCNC1	-0.70831	4.904195	-3.18495	0.002131	0.006082	-2.2279	Down
JUNB	1.070348	8.20687	3.183942	0.002147	0.006126	-2.23381	Up
CCL3	1.620921	3.758783	3.182422	0.002157	0.006152	-2.23807	Up
LINC0301:	0.635639	6.287045	3.179912	0.002163	0.006167	-2.24203	Up
MOXD1	0.521602	4.263795	3.17731	0.00218	0.006211	-2.24932	Up
CCNE2	0.636029	3.640434	3.173166	0.002208	0.006284	-2.26093	Up
RFX2	-0.57771	5.899037	-3.17202	0.002216	0.006304	-2.26414	Down
IL34	-0.58419	5.514014	-3.17145	0.002219	0.006313	-2.26574	Down
LOHAN2	-0.58254	3.338756	-3.16927	0.002234	0.00635	-2.27184	Down
GADD45B	0.948009	7.120043	3.16933	0.002239	0.006363	-2.27328	Up
TRBV19	0.52748	6.089747	3.166936	0.00225	0.00639	-2.27837	Up
ZNF616	0.648905	3.83427	3.165118	0.002262	0.006424	-2.28345	Up
LINC0195	0.508354	5.093891	3.164037	0.00227	0.006443	-2.28647	Up
GALNT9	-0.60107	3.412674	-3.16352	0.002273	0.006452	-2.2879	Down
NFKBIZ	0.670458	7.269749	3.162323	0.002284	0.006479	-2.29203	Up
LOC1005C	0.726061	4.412146	3.160207	0.002297	0.00651	-2.2973	Up
SNORD50	0.625919	7.375264	3.157394	0.002318	0.006566	-2.30562	Up
PKLR	-0.57123	3.858026	-3.15447	0.002336	0.006615	-2.31316	Down
DYNLT5	0.678645	3.416557	3.153324	0.002344	0.006637	-2.31636	Up
BEX2	1.054045	7.221717	3.152154	0.002361	0.006682	-2.32193	Up
LINC0031(	-1.42119	4.049149	-3.15093	0.002371	0.006707	-2.32571	Down
TTF2	0.532488	4.556121	3.143263	0.002416	0.006827	-2.34437	Up
IL17D	-0.88526	10.04774	-3.14385	0.00242	0.006836	-2.34501	Down
SSPOP	-0.51035	3.801088	-3.1423	0.002423	0.006842	-2.34703	Down
HOXB4	0.56819	5.290905	3.140667	0.002435	0.006872	-2.35159	Up
P2RY14	0.599693	6.981223	3.137418	0.00246	0.006937	-2.36078	Up
CDRT15	-0.55886	3.511813	-3.13663	0.002465	0.00695	-2.3628	Down
PI16	0.631106	6.818607	3.133388	0.00249	0.007016	-2.37212	Up
PTPRG	0.532371	7.227812	3.128457	0.002526	0.007108	-2.38546	Up
ANXA2P1	0.57003	4.527407	3.125898	0.002545	0.007161	-2.39255	Up
MSC	-0.54085	4.561658	-3.12268	0.00257	0.007226	-2.40147	Down
ARSD	0.64338	5.152414	3.122625	0.00257	0.007226	-2.4016	Up
NAALADL	0.685406	3.318354	3.120098	0.00259	0.007279	-2.40859	Up
BIRC5	0.685806	3.586967	3.117711	0.002608	0.007328	-2.41519	Up
MGAT5	0.532075	8.395777	3.116597	0.002618	0.00735	-2.41841	Up
OR4D1	-0.68267	3.097726	-3.11552	0.002626	0.007369	-2.42125	Down
PIEZO2	1.060439	4.393281	3.115738	0.00263	0.007378	-2.42214	Up
GSTO2	-0.51411	3.84532	-3.11282	0.002647	0.007418	-2.4287	Down
ADAMTS1	-0.99281	3.372477	-3.11327	0.002647	0.007418	-2.42835	Down
IGFBP2	-0.59085	5.788808	-3.112	0.002653	0.007435	-2.43097	Down

NTF4	-0.65858	5.483319	-3.11126	0.002659	0.007448	-2.43301	Down
C20orf181	-0.67175	5.261801	-3.10961	0.002672	0.00748	-2.43754	Down
SRGAP2B	0.553832	6.942469	3.106422	0.002698	0.007544	-2.44634	Up
UBAP1L	-0.80665	4.768301	-3.105	0.002712	0.007579	-2.45098	Down
LUZP2	0.644448	2.671608	3.10255	0.002729	0.00762	-2.457	Up
PPP1R1A	-0.71295	11.72064	-3.10169	0.002743	0.007655	-2.46107	Down
ADAMTS8	-0.86341	4.933333	-3.10031	0.002751	0.007674	-2.46404	Down
CRIM1	-0.52182	9.499218	-3.09992	0.002752	0.007675	-2.46456	Down
FADS1	0.608021	6.838416	3.099524	0.002755	0.007682	-2.46555	Up
NPC1L1	-0.51408	6.611988	-3.09766	0.002769	0.007719	-2.47046	Down
LINC0169	1.172791	3.821434	3.091776	0.002824	0.007857	-2.48808	Up
ASAP1-IT	0.667304	4.36789	3.087571	0.002854	0.007932	-2.49816	Up
EGR2	1.434862	3.985986	3.087514	0.002866	0.007961	-2.50103	Up
ARTN	-0.71476	4.137166	-3.08583	0.002869	0.007966	-2.50299	Down
LOC10537	-0.76556	3.413154	-3.08149	0.002906	0.008061	-2.51482	Down
IER2	0.640039	8.879629	3.081163	0.002912	0.008078	-2.51657	Up
HS3ST5	-0.63579	5.861464	-3.08062	0.002913	0.008079	-2.51722	Down
CRYGC	-0.62234	3.362705	-3.07968	0.002921	0.0081	-2.51977	Down
LOC10537	-0.77637	3.620899	-3.0777	0.002939	0.008148	-2.52535	Down
NANOS1	-0.78847	7.505438	-3.07799	0.002942	0.008156	-2.52584	Down
MIR4296	-0.62895	3.025495	-3.07711	0.002944	0.008158	-2.52682	Down
LINC0270	0.64442	3.096812	3.076593	0.002948	0.00817	-2.52823	Up
LINC0312	1.189807	2.970051	3.076275	0.002956	0.00819	-2.53018	Up
ZC2HC1B	0.872643	3.8737	3.075877	0.002958	0.008196	-2.53103	Up
KIF1C-AS	-0.50079	3.737155	-3.07463	0.002965	0.008212	-2.53361	Down
PSRC1	0.511195	3.672094	3.073896	0.002972	0.008228	-2.5356	Up
MUC19	-0.51818	4.876078	-3.07266	0.002983	0.008252	-2.53899	Down
DCAF12L2	0.761289	3.736145	3.072189	0.002987	0.008264	-2.54042	Up
PRAMEF1(	-0.68271	3.541446	-3.06875	0.003017	0.008341	-2.54967	Down
LOC7301C	0.639657	3.528497	3.068074	0.003023	0.008354	-2.5515	Up
TTC16	-0.57077	3.863978	-3.06715	0.003032	0.008374	-2.55403	Down
ZNF568	0.72551	4.910709	3.062254	0.003077	0.008488	-2.56771	Up
PTPRN2	0.554409	5.243634	3.058184	0.003113	0.008576	-2.57846	Up
ELFN2	-0.55924	4.752432	-3.05687	0.003125	0.008603	-2.58205	Down
PRRG4	-0.50967	3.695993	-3.05666	0.003127	0.008607	-2.58259	Down
TMEM67	1.128643	4.234594	3.056911	0.003132	0.008615	-2.58331	Up
MYSM1	0.775215	7.595603	3.055651	0.003143	0.008643	-2.58674	Up
ITIH4	-0.73603	8.441029	-3.0552	0.003147	0.008654	-2.58797	Down
CD36	0.654166	11.20148	3.055084	0.003148	0.008656	-2.58828	Up
AMFR	-1.21141	7.059219	-3.05481	0.003157	0.008675	-2.59023	Down
LINC0224	-0.53608	3.809271	-3.05335	0.003158	0.008676	-2.59161	Down
RADIL	-0.50819	5.189129	-3.05164	0.003174	0.008716	-2.59626	Down
CPLX1	0.850887	4.115411	3.051023	0.003184	0.00874	-2.59875	Up
HS6ST3	-0.53361	3.565671	-3.04983	0.003191	0.008758	-2.60118	Down
PPDPFL	-1.17705	11.20803	-3.04767	0.003224	0.008841	-2.60962	Down
IGSF11	-0.86363	4.776309	-3.04614	0.00323	0.008853	-2.612	Down
SLC2A9	-0.65985	3.885442	-3.04332	0.003253	0.008912	-2.61885	Down
LOC10536	0.545278	3.723255	3.042982	0.003256	0.008919	-2.61977	Up
KCNK1	0.527641	4.611429	3.041171	0.003273	0.008963	-2.62468	Up
CLEC2D	0.73601	3.661363	3.040464	0.00328	0.008977	-2.62659	Up
MMRN1	1.192188	3.70429	3.035395	0.003336	0.009121	-2.64167	Up
ACAN	0.635247	5.008816	3.034225	0.003341	0.009131	-2.64349	Up
PPP1R3B-	-0.51434	5.78718	-3.03322	0.003351	0.009153	-2.64621	Down
ATP2A1-A	-0.65904	6.124184	-3.02912	0.003393	0.009262	-2.6576	Down
FTCDNL1	-0.65578	4.857728	-3.02796	0.003403	0.009285	-2.66044	Down
WASHC4	0.796236	8.400694	3.024569	0.003444	0.009387	-2.67091	Up
SYCE1L	0.600966	5.315112	3.018749	0.003496	0.009516	-2.68528	Up
XIST	-3.79971	4.168369	-3.02058	0.003498	0.009521	-2.68414	Down

TC2N	0.736262	4.790048	3.017834	0.003508	0.009546	-2.68819	Up
MIR101-1	-0.75308	5.913275	-3.01499	0.003539	0.009624	-2.6962	Down
LCN1	-0.53161	2.963192	-3.01413	0.003543	0.009635	-2.69773	Down
SCG5	1.017749	3.26418	3.014199	0.003547	0.009641	-2.69832	Up
MIR155HC	0.697663	3.863735	3.010817	0.003578	0.009719	-2.70664	Up
DIRAS1	0.904229	4.250124	3.011152	0.00358	0.009722	-2.70667	Up
LINC0154	-0.59288	5.069129	-3.01026	0.003584	0.009731	-2.70815	Down
CHRND	0.885052	4.735362	3.009957	0.003592	0.00975	-2.70988	Up
COL8A1	0.902472	6.365962	3.009117	0.003603	0.009777	-2.71251	Up
KCNS3	-0.58013	8.421143	-3.00693	0.003622	0.009822	-2.71771	Down
ARFRP1	-0.63094	6.413743	-3.00555	0.003635	0.009851	-2.72106	Down
MAOA	-1.45349	6.815423	-3.0062	0.003641	0.009865	-2.72148	Down
KRT222	1.212069	3.247051	3.004269	0.003655	0.009899	-2.72553	Up
IL7	1.010004	3.144855	2.998659	0.003712	0.010045	-2.74005	Up
PRM1	-0.61147	3.154462	-2.9955	0.003742	0.010117	-2.74775	Down
ARL13B	0.812067	5.234287	2.99464	0.003755	0.010153	-2.75082	Up
ROBO3	1.041125	5.091857	2.993688	0.003769	0.010184	-2.75388	Up
CXCR3	-0.52658	3.736499	-2.99069	0.003794	0.010245	-2.76063	Down
ZNF345	0.603093	4.085929	2.988604	0.003818	0.010296	-2.76622	Up
TWIST2	0.614303	4.611044	2.987827	0.003826	0.010317	-2.7683	Up
CDKN2D	-0.62586	7.028991	-2.9848	0.003863	0.010409	-2.77689	Down
FBXL22	0.550877	5.906455	2.981168	0.003901	0.0105	-2.78609	Up
WDHD1	0.54727	3.145692	2.980024	0.003914	0.010531	-2.78914	Up
PTPN22	0.844382	3.184095	2.979855	0.003917	0.010538	-2.78976	Up
LPAR4	0.680488	4.645053	2.979256	0.003923	0.010554	-2.7913	Up
LRRC61	-0.59662	4.217208	-2.97844	0.003932	0.010574	-2.79335	Down
CD1E	1.050366	3.300693	2.97591	0.003967	0.010662	-2.80106	Up
PLOD2	0.724959	6.637251	2.973536	0.003993	0.010726	-2.80717	Up
CYP11B2	-0.81823	3.935414	-2.97274	0.004001	0.010744	-2.80916	Down
SMIM24	-0.62615	2.739765	-2.97172	0.004009	0.010758	-2.81127	Down
SPRR2D	-0.53167	3.546718	-2.97037	0.004025	0.010799	-2.81488	Down
TLR10	-0.51569	4.50828	-2.968	0.004053	0.010867	-2.82118	Down
MORN4	-0.54296	5.472904	-2.96671	0.004068	0.010906	-2.8246	Down
ATXN8OS	-0.52873	3.872638	-2.96553	0.004082	0.010936	-2.82774	Down
LINC0287	-0.82239	5.503948	-2.96094	0.004141	0.011083	-2.84066	Down
PPP1R27	-0.5279	9.216	-2.95555	0.004205	0.011236	-2.85471	Down
ANKRD42	-0.5295	4.611622	-2.95498	0.004209	0.011244	-2.85575	Down
E2F5	0.525682	3.556775	2.953665	0.004225	0.011284	-2.85923	Up
ZNF646	-0.52066	3.617438	-2.95314	0.004231	0.0113	-2.86063	Down
MARCHF1	-0.57516	3.556811	-2.95189	0.004246	0.011335	-2.86392	Down
LINC0158	-0.51055	3.796852	-2.95188	0.004246	0.011335	-2.86397	Down
NUDT11	0.727122	3.280399	2.949253	0.004279	0.011415	-2.87091	Up
TENT5C	-0.68742	7.885058	-2.946	0.004327	0.01153	-2.88062	Down
NLGN4Y	1.013051	3.727844	2.941858	0.004379	0.011662	-2.89164	Up
LOC10537	0.558945	2.867309	2.941181	0.004379	0.011662	-2.89226	Up
SIGLEC8	-0.63587	4.431623	-2.94055	0.004387	0.011678	-2.89393	Down
ITGA4	1.055131	4.657338	2.939992	0.004403	0.011714	-2.89657	Up
ASTN2	-0.6328	5.577618	-2.93924	0.004404	0.011716	-2.8974	Down
CDCA7	0.675695	4.747464	2.93904	0.004407	0.011723	-2.89804	Up
NAIP	0.695372	6.20096	2.936889	0.004438	0.011794	-2.90415	Up
PRKAR1B	-0.74629	5.730883	-2.93226	0.004498	0.011938	-2.91647	Down
AGBL3	0.67886	3.465065	2.931504	0.004503	0.011949	-2.91778	Up
GAD1	-0.68625	3.205666	-2.93117	0.004507	0.011956	-2.91865	Down
TLCD5	0.612164	5.055018	2.930696	0.004514	0.011968	-2.91991	Up
SOX14	0.875267	2.633919	2.930633	0.004514	0.011969	-2.92007	Up
PCK2	0.621019	4.635363	2.930187	0.00452	0.01198	-2.92125	Up
KCNQ1	0.859924	4.2637	2.93055	0.004521	0.01198	-2.92098	Up
MYEF2	0.597979	5.013203	2.927431	0.004556	0.01207	-2.9285	Up

KIF20A	1.154847	3.117059	2.926646	0.004575	0.012111	-2.93172	Up
ICOS	-0.76906	4.701641	-2.92501	0.004592	0.01215	-2.93542	Down
PARM1	-0.75696	6.868487	-2.92483	0.004599	0.012165	-2.93647	Down
CD34	0.514871	8.30703	2.923551	0.004609	0.012187	-2.93893	Up
LOC15176	-0.54465	3.895865	-2.91954	0.004661	0.012316	-2.94925	Down
DRD3	-0.83218	2.309244	-2.9181	0.00468	0.012364	-2.95303	Down
TMIE	-0.54952	6.673631	-2.91582	0.004711	0.012436	-2.95901	Down
LRRC74A	-0.56405	4.020815	-2.91511	0.00472	0.012457	-2.96087	Down
LIPC-AS1	-0.78093	2.158848	-2.91367	0.00474	0.012505	-2.96466	Down
HOTAIR	0.990478	2.949964	2.913376	0.004748	0.012524	-2.96596	Up
BARD1	0.505153	7.327799	2.912508	0.004755	0.012541	-2.96769	Up
MGC4859	0.771057	2.093376	2.9116	0.004768	0.012572	-2.97007	Up
STARD13-	1.006407	3.135988	2.911403	0.004776	0.012589	-2.97125	Up
CHD5	-0.72815	4.336639	-2.90763	0.004825	0.012711	-2.98087	Down
ANKRD60	-0.63529	3.387822	-2.90727	0.004827	0.012714	-2.98142	Down
CNTN1	0.678352	6.674691	2.906408	0.004844	0.012756	-2.98433	Up
EVC	0.632655	6.110755	2.90614	0.004845	0.012756	-2.98462	Up
COL8A2	0.967462	4.807296	2.904685	0.004872	0.012822	-2.98929	Up
CYLC1	-0.89489	4.65224	-2.90398	0.004882	0.012845	-2.99113	Down
LRRC69	0.728058	4.019185	2.900422	0.004925	0.01295	-2.99963	Up
PMS2P5	-0.50106	7.845028	-2.89983	0.004931	0.012962	-3.00085	Down
KIF22	-0.56312	7.053252	-2.8972	0.00497	0.01306	-3.00796	Down
TAS2R14	0.535531	4.014811	2.893887	0.005015	0.013171	-3.01637	Up
CHST11	0.728365	3.995357	2.893367	0.005025	0.013192	-3.01804	Up
CNTROB	-0.50534	6.389288	-2.89318	0.005025	0.013192	-3.0182	Down
ADGRG6	0.63071	4.346834	2.892134	0.00504	0.013228	-3.02094	Up
CXCL10	1.150764	5.630893	2.892133	0.005054	0.013262	-3.02255	Up
MUSK	0.638781	3.71158	2.89002	0.005071	0.013299	-3.02645	Up
FOS	1.654308	7.110168	2.890457	0.005087	0.013337	-3.02803	Up
BCAS1	0.993548	4.006588	2.888551	0.005101	0.01337	-3.03136	Up
TDRD6	-0.7921	3.347406	-2.88707	0.005116	0.013397	-3.03436	Down
LRRTM1	-0.58234	3.778943	-2.88612	0.005127	0.013424	-3.03659	Down
ZACN	-0.64973	3.771093	-2.88133	0.005198	0.01359	-3.04906	Down
TRIM61	0.521372	4.673184	2.878408	0.005241	0.01369	-3.05664	Up
LINC0159	0.604331	3.152872	2.8777	0.005252	0.013712	-3.05848	Up
NAV2	0.551328	7.118334	2.872751	0.005328	0.013891	-3.07153	Up
SLC18A2	0.935668	3.186659	2.870531	0.005365	0.013981	-3.07769	Up
RBP4	1.968356	5.449732	2.870323	0.005386	0.014028	-3.08026	Up
LRRC10B	-0.81675	4.163682	-2.86885	0.005391	0.014038	-3.08205	Down
VLDLR-AS	-0.72459	5.000357	-2.86753	0.00541	0.014086	-3.08533	Down
SNCAIP	0.708888	5.145141	2.866501	0.005425	0.014119	-3.08794	Up
LOC28581	-0.70852	3.621019	-2.86436	0.005455	0.01419	-3.09306	Down
XRRA1	-0.55911	3.810719	-2.86377	0.005463	0.014206	-3.09456	Down
CAMKK2	0.630997	7.165698	2.862634	0.005487	0.014255	-3.09813	Up
PRSS12	0.736094	4.161286	2.857211	0.00557	0.014454	-3.11193	Up
CCDC125	0.557437	4.40601	2.855798	0.005588	0.014497	-3.11517	Up
NPHP3-AS	-0.53738	3.902777	-2.85324	0.005629	0.014599	-3.12177	Down
TOP2A	1.235045	3.685876	2.848803	0.005712	0.01479	-3.13453	Up
SLC22A15	0.637585	4.046839	2.847687	0.005718	0.014802	-3.13607	Up
LINC0160	-0.54467	5.108162	-2.84622	0.005741	0.01486	-3.13984	Down
LOC10537	0.724478	2.320775	2.844026	0.005777	0.014943	-3.14549	Up
SSTR5	-0.71301	2.813804	-2.84235	0.005804	0.015012	-3.14979	Down
BMPR1B	0.731066	5.905984	2.842272	0.005812	0.015029	-3.1506	Up
THRSP	1.042644	7.229455	2.842966	0.005813	0.01503	-3.15012	Up
PM20D1	-0.50771	5.772025	-2.83551	0.005917	0.01528	-3.16736	Down
HOXD10	-0.73034	4.510507	-2.83512	0.005929	0.015307	-3.16884	Down
RRH	-0.63561	2.504592	-2.83359	0.005949	0.015356	-3.17229	Down
CA8	-0.56946	3.679671	-2.83347	0.005951	0.015359	-3.1726	Down

ATP8B1	0.529197	8.986605	2.83234	0.005975	0.015415	-3.17598	Up
KIAA1586	0.587347	4.323652	2.831196	0.00599	0.015444	-3.17842	Up
LINC0170(	-0.6177	2.275083	-2.83065	0.005999	0.015464	-3.17981	Down
NMNAT1	-0.5147	6.9432	-2.82962	0.006016	0.015503	-3.18245	Down
NPY6R	-0.54966	9.732763	-2.82308	0.006134	0.015779	-3.19978	Down
ZFP14	0.702609	3.07236	2.820036	0.00618	0.01589	-3.20698	Up
KRT36	0.855682	2.3011	2.818107	0.006214	0.015971	-3.2119	Up
LINC0100	0.521779	5.134053	2.818002	0.006216	0.015973	-3.21217	Up
MEF2C-AS	-0.6815	2.666684	-2.8169	0.006235	0.016017	-3.21498	Down
LOC33929	-0.52461	4.017176	-2.81513	0.006266	0.016091	-3.21949	Down
LSAMP	0.632275	3.288144	2.812866	0.006306	0.016181	-3.22527	Up
VPS9D1-A	0.798942	3.122044	2.811764	0.006326	0.016226	-3.22817	Up
ADAMTS3	0.697792	3.54065	2.808384	0.006385	0.016368	-3.23669	Up
TIMP4	-1.01642	7.31138	-2.80855	0.006402	0.016403	-3.23808	Down
PF4	-0.81339	4.327175	-2.80515	0.00645	0.016517	-3.24548	Down
USHBP1	-0.81031	3.658475	-2.80107	0.006522	0.016689	-3.25575	Down
LOC10192	-0.72202	1.629837	-2.79773	0.006578	0.016822	-3.26378	Down
SLC16A8	-0.57523	2.896056	-2.79594	0.006611	0.016898	-3.2683	Down
SCN9A	0.671988	3.455265	2.793765	0.006651	0.016991	-3.27383	Up
IGLV2-14	-0.54915	4.664241	-2.7933	0.00666	0.017009	-3.275	Down
WFDC1	-0.70589	3.980729	-2.79296	0.006669	0.017029	-3.27614	Down
RFPL1S	0.666331	3.035548	2.78941	0.006733	0.01718	-3.28486	Up
CSN3	-0.81375	2.78421	-2.78717	0.006775	0.017269	-3.29053	Down
ARL17A	-0.5542	6.940868	-2.78664	0.006787	0.017298	-3.29207	Down
C3orf62	0.504845	4.013345	2.783777	0.006839	0.017422	-3.29911	Up
FMOD	0.73246	8.024072	2.784305	0.00684	0.017422	-3.29871	Up
VXN	-0.53784	3.809328	-2.78367	0.006841	0.017424	-3.2994	Down
FBP1	0.600603	4.086637	2.781823	0.006876	0.017501	-3.30405	Up
TPPP	-0.77992	7.00254	-2.77991	0.006924	0.017611	-3.30981	Down
LEPR	0.915608	6.703218	2.77819	0.006958	0.017686	-3.31425	Up
LINC0030	-0.69939	2.482541	-2.77741	0.006961	0.017691	-3.31521	Down
FTCD	-0.67155	5.685691	-2.7773	0.006968	0.017702	-3.31587	Down
ZNF37BP	0.595317	4.695681	2.776852	0.006972	0.01771	-3.3166	Up
ADGRL1	0.675836	4.832285	2.772763	0.007055	0.017894	-3.32722	Up
CCDC68	-0.55929	4.01218	-2.7723	0.007061	0.017905	-3.32807	Down
IGFBP4	0.695008	9.049269	2.769465	0.007127	0.018044	-3.33612	Up
TMEM74	-0.56157	3.737796	-2.76863	0.007133	0.01805	-3.33733	Down
WFIKKN2	-0.64778	3.067726	-2.76799	0.007145	0.018074	-3.33893	Down
CRABP2	0.673615	5.940299	2.767701	0.007157	0.018097	-3.34009	Up
NCAM1	0.843351	6.866023	2.767388	0.007169	0.018125	-3.34135	Up
AHSG	-0.6774	3.632077	-2.76661	0.007173	0.018134	-3.3424	Down
PCK1	1.670808	3.936126	2.766208	0.007202	0.018199	-3.3451	Up
LOC10192	-0.51821	3.467974	-2.76497	0.007205	0.018204	-3.34652	Down
TXLNGY	1.829493	5.624188	2.763064	0.007272	0.018354	-3.35355	Up
LAD1	0.788641	4.964194	2.761182	0.007288	0.018387	-3.35657	Up
SHANK1	-0.6677	3.832757	-2.76081	0.00729	0.018389	-3.35706	Down
SPACA9	-0.62182	6.355598	-2.76015	0.007307	0.018427	-3.35899	Down
PIM1	0.619738	6.089984	2.759915	0.007311	0.018433	-3.35953	Up
CDKAL1	-0.92965	6.165528	-2.75948	0.007327	0.018465	-3.3612	Down
CACNA1I	-0.7638	3.891435	-2.75754	0.007361	0.018539	-3.36563	Down
KRTDAP	0.744892	2.133586	2.757232	0.007361	0.018539	-3.36596	Up
BEND6	0.665002	3.413946	2.755242	0.007402	0.01863	-3.37095	Up
LDOC1	0.50196	6.418691	2.755009	0.007407	0.01864	-3.37153	Up
PHLDA2	0.638132	4.178762	2.753339	0.007442	0.018717	-3.37577	Up
TPPP2	-0.86103	4.224297	-2.74717	0.00758	0.019027	-3.39201	Down
SATB2	0.516635	5.124664	2.744915	0.007616	0.019106	-3.3968	Up
ADAMTS7	-0.50012	3.983503	-2.74455	0.007624	0.019122	-3.3977	Down
HTR7	-0.76364	4.167456	-2.74293	0.007665	0.019211	-3.40227	Down

GAL	0.731981	3.623293	2.742726	0.007666	0.019211	-3.40252	Up
SORBS2	-0.5898	7.546217	-2.74162	0.007692	0.019277	-3.40554	Down
QPCT	0.657413	4.640412	2.741031	0.007701	0.019296	-3.40672	Up
LRP8	-0.52818	5.905187	-2.73997	0.007721	0.019336	-3.40914	Down
TTTY14	2.183728	6.394801	2.738032	0.007795	0.019493	-3.41645	Up
C20orf141	-0.53396	3.434257	-2.73306	0.007869	0.019659	-3.42638	Down
PRDM8	-0.85227	2.24126	-2.73138	0.007905	0.019745	-3.43055	Down
RPH3AL	-0.6269	5.788691	-2.73063	0.007925	0.019788	-3.43269	Down
PLEKHH2	0.79766	4.712341	2.730436	0.007934	0.019802	-3.43347	Up
CALCA	-0.83657	5.157064	-2.73029	0.007941	0.019815	-3.43411	Down
PTPRR	-0.52184	3.778545	-2.72911	0.007955	0.01984	-3.4362	Down
CYP51A1	0.514864	6.782499	2.727347	0.007993	0.019928	-3.44059	Up
SRRM3	-0.60464	3.242443	-2.72288	0.008092	0.02014	-3.45168	Down
MZB1	-0.58815	4.021284	-2.72123	0.008129	0.020224	-3.4558	Down
LDAF1	-0.65578	8.956983	-2.72072	0.008152	0.020275	-3.45789	Down
ST8SIA2	1.247434	5.472773	2.718018	0.008223	0.020436	-3.46532	Up
ZNF385D	0.688383	5.517255	2.71557	0.008261	0.020517	-3.47022	Up
MIR181A2	-0.54817	5.103362	-2.71006	0.008381	0.020777	-3.48345	Down
CCHCR1	-0.58384	6.715399	-2.70699	0.008456	0.020938	-3.49138	Down
PTGES	0.513004	4.633278	2.702232	0.008562	0.021187	-3.5028	Up
PDE4B	0.571818	8.993448	2.70168	0.008583	0.021228	-3.50467	Up
LOC10537	0.746653	3.26814	2.700954	0.008593	0.02125	-3.50605	Up
CPM	0.764201	6.305518	2.701272	0.008597	0.021256	-3.50598	Up
PRB3	-0.79909	3.031099	-2.6999	0.008618	0.021302	-3.50866	Down
FRMD8P1	-0.56193	4.316496	-2.69966	0.008622	0.021307	-3.50915	Down
FAM83B	-0.50167	3.7445	-2.69773	0.008668	0.021414	-3.5139	Down
KCNN2	-1.12522	4.473934	-2.69524	0.008745	0.021587	-3.5212	Down
FOXD2-AS	-0.67689	4.018985	-2.69275	0.00879	0.02168	-3.52639	Down
CRYM	-1.98317	4.729777	-2.69189	0.008843	0.021798	-3.53057	Down
LOC28478	-0.76962	2.977483	-2.68683	0.008929	0.022001	-3.54072	Down
CENPK	0.953524	3.109898	2.686579	0.008942	0.022028	-3.5418	Up
AMPD3	-0.53128	8.045997	-2.68413	0.009001	0.022155	-3.54773	Down
PLXNA4	-0.733	4.367577	-2.68398	0.009006	0.022162	-3.54819	Down
VASP	-0.58298	6.680295	-2.68092	0.009079	0.022326	-3.55554	Down
E2F8	0.671856	9.012991	2.679766	0.009115	0.022399	-3.55883	Up
CRYBB2P1	-0.57997	4.244605	-2.67809	0.009143	0.022459	-3.56215	Down
AIRE	-0.50683	6.747478	-2.67695	0.009172	0.022526	-3.56495	Down
STEAP2	0.945838	4.995429	2.676166	0.009204	0.022593	-3.56764	Up
VSTM2L	0.7988	3.318183	2.673693	0.009258	0.022716	-3.57324	Up
MIR133A1	0.506623	11.81	2.673297	0.009276	0.022753	-3.57466	Up
OR10A4	0.676605	2.741028	2.672367	0.009286	0.022769	-3.57616	Up
RPRML	-0.61471	3.634019	-2.67117	0.009316	0.022835	-3.57909	Down
LINC0207	-0.57396	4.169102	-2.67013	0.009343	0.022894	-3.58163	Down
PLAC8	0.61942	3.819738	2.668034	0.009396	0.023014	-3.58675	Up
LOC10537	0.50913	2.751657	2.667418	0.009412	0.023049	-3.58826	Up
LOC10537	-0.64741	3.208641	-2.66676	0.009428	0.023085	-3.58987	Down
CGB3	-0.68708	2.563386	-2.66623	0.009442	0.02311	-3.59115	Down
DOCK5	0.500529	6.222673	2.665264	0.009467	0.02316	-3.59351	Up
MRPL44	-0.5007	7.709888	-2.66454	0.009489	0.023211	-3.59548	Down
PLCH1	-0.58504	5.369992	-2.66331	0.009518	0.023272	-3.59833	Down
LOC10028	-0.61728	4.514219	-2.66313	0.009523	0.023281	-3.59879	Down
DERL3	-0.52751	5.968175	-2.66176	0.009557	0.023354	-3.60207	Down
LINC0207	0.752412	5.295046	2.661636	0.009568	0.023372	-3.60281	Up
IER3	0.541101	7.404774	2.658655	0.009643	0.023535	-3.60996	Up
RBMY3AP	-0.55843	3.669351	-2.65813	0.009651	0.023551	-3.6109	Down
MXD3	-0.55788	4.531376	-2.65662	0.00969	0.023639	-3.61457	Down
LINC0286	-0.53377	4.678759	-2.65497	0.009734	0.02374	-3.61859	Down
LINC0168	0.715929	2.831035	2.652167	0.009807	0.023906	-3.62541	Up

LINC0054:	-0.65982	2.240992	-2.65078	0.009844	0.023982	-3.62878	Down
BMP7	-0.60656	4.813314	-2.65003	0.009865	0.024031	-3.63067	Down
HBBP1	-0.83009	5.949882	-2.65024	0.009872	0.024044	-3.63083	Down
GLT1D1	-0.80912	5.211798	-2.64659	0.009969	0.024259	-3.63969	Down
HKDC1	-0.52198	3.826021	-2.64415	0.010021	0.024375	-3.64486	Down
TRIM17	-0.7543	2.424359	-2.64222	0.010074	0.024491	-3.64953	Down
SCD	1.464489	8.459089	2.640327	0.010164	0.024674	-3.65622	Up
ID4	0.684739	7.113696	2.639143	0.010171	0.024685	-3.65772	Up
RASSF5	0.58477	6.472438	2.635188	0.010271	0.024904	-3.66686	Up
TKTL1	0.562426	3.969539	2.634294	0.01029	0.024936	-3.66872	Up
NOX4	0.798006	4.438619	2.633686	0.010319	0.024996	-3.67081	Up
KLB	0.944405	4.582116	2.632127	0.010364	0.025094	-3.67467	Up
LRG1	-0.66717	4.328548	-2.63151	0.010373	0.02511	-3.67573	Down
CTSH	0.523679	8.131144	2.630203	0.010411	0.025196	-3.67895	Up
LOC10192	0.574707	3.362695	2.629426	0.010426	0.025229	-3.68047	Up
EDIL3	0.949197	6.682258	2.628369	0.010475	0.025338	-3.68407	Up
OSM	-0.5546	3.92996	-2.62728	0.010486	0.025352	-3.68565	Down
ADCYAP1I	0.521012	3.9172	2.624862	0.010554	0.025508	-3.69148	Up
LINC0068:	-0.50552	2.605973	-2.62255	0.01062	0.025652	-3.69706	Down
SYT13	-0.63218	4.179779	-2.62059	0.010678	0.025779	-3.7019	Down
FIBCD1	-0.9219	3.954995	-2.61656	0.010805	0.026052	-3.71215	Down
PPM1E	0.78172	3.218187	2.615018	0.010841	0.026129	-3.71541	Up
DPY19L2	0.511642	4.383092	2.614427	0.010853	0.026156	-3.71658	Up
SPATC1L	-0.70504	5.030669	-2.61411	0.01087	0.026195	-3.71777	Down
ALMS1-IT	0.590688	3.140803	2.610674	0.010962	0.026387	-3.72559	Up
NMUR1	-0.58526	3.331578	-2.60778	0.011047	0.02657	-3.73253	Down
RTN4RL2	-0.68173	3.483037	-2.60751	0.011057	0.02659	-3.73325	Down
PAX8-AS1	1.051674	5.009969	2.607712	0.01107	0.026615	-3.7337	Up
UNC119	-0.59675	4.357423	-2.60589	0.011104	0.026689	-3.7371	Down
SH2D1B	-1.30337	3.322778	-2.60488	0.011156	0.026797	-3.74058	Down
LINC0236:	0.745269	3.030506	2.603171	0.011184	0.026858	-3.74356	Up
LOC44089	0.789022	3.998468	2.599368	0.011306	0.027124	-3.75309	Up
PELATON	-0.52096	4.031975	-2.59595	0.011401	0.027334	-3.76083	Down
ZBTB48	-0.52194	4.672606	-2.59521	0.011423	0.027382	-3.7626	Down
RTN1	0.585856	5.681681	2.594504	0.011447	0.027434	-3.76442	Up
TFPI2	-0.84021	3.125194	-2.58808	0.011648	0.027863	-3.77991	Down
LINC0064!	0.791504	3.039879	2.587406	0.011666	0.027899	-3.78137	Up
RAB36	0.6397	2.84845	2.582759	0.011807	0.028204	-3.79225	Up
KANK4	0.673061	3.66521	2.581796	0.011841	0.028281	-3.7947	Up
TMEM255	-0.51508	4.554863	-2.58044	0.01188	0.028362	-3.79776	Down
MOB3B	0.514269	6.587017	2.579738	0.011903	0.028409	-3.7995	Up
ZFY	1.558934	6.005906	2.580515	0.011905	0.028409	-3.79882	Up
HS3ST3B1	0.714621	3.885423	2.579076	0.01193	0.028462	-3.80132	Up
LYVE1	0.737222	7.206514	2.579023	0.011939	0.02848	-3.80178	Up
LINC0224:	-0.58767	4.473041	-2.57825	0.01195	0.0285	-3.803	Down
NTSR1	-0.55733	3.220747	-2.57665	0.011999	0.028603	-3.80677	Down
NPTX2	-0.92622	4.417201	-2.5742	0.012092	0.028801	-3.81321	Down
ITGA11	0.527143	5.619254	2.57358	0.012097	0.02881	-3.81404	Up
SMOC2	0.514206	9.747917	2.573142	0.01212	0.028852	-3.81546	Up
APLN	-0.5833	4.06841	-2.56954	0.012227	0.029087	-3.82361	Down
LOC10537	0.642217	2.805777	2.567841	0.012282	0.029212	-3.82763	Up
MAP3K10	-0.5619	3.514247	-2.56589	0.012345	0.029346	-3.83224	Down
SOD3	0.602763	5.805349	2.564909	0.012383	0.029429	-3.83481	Up
AMIGO2	0.551241	5.646056	2.562433	0.012459	0.029601	-3.84045	Up
IL4R	0.586339	5.974043	2.56164	0.01249	0.02966	-3.84252	Up
ITLN1	-0.71373	3.654115	-2.55869	0.012589	0.029865	-3.84953	Down
LOC10537	0.542303	3.188811	2.558219	0.012598	0.029879	-3.85036	Up
MAP3K5-/	0.729273	3.147944	2.556614	0.012653	0.02999	-3.85422	Up

LINC0250:	-1.12105	2.380132	-2.55575	0.012689	0.030065	-3.85656	Down
DEFB114	0.651744	1.960147	2.551508	0.012822	0.030331	-3.86617	Up
GAS6-AS1	0.505309	4.216363	2.549912	0.012876	0.030448	-3.86993	Up
RS1	-0.5246	3.653891	-2.54921	0.0129	0.030501	-3.87157	Down
DLEU7	-0.57915	1.710152	-2.54872	0.012917	0.03053	-3.87274	Down
XCL1	0.67654	3.804942	2.54635	0.013003	0.030721	-3.87854	Up
SLC4A9	-0.60941	3.473406	-2.5434	0.013098	0.030929	-3.88524	Down
EME1	-0.69664	5.012096	-2.54166	0.013167	0.031085	-3.88968	Down
RTKN	0.515864	5.571158	2.540009	0.013215	0.031191	-3.89319	Up
GSDMC	-0.85763	6.303945	-2.54017	0.013226	0.031214	-3.89346	Down
RAB15	0.559056	6.753966	2.537852	0.013298	0.031368	-3.89853	Up
C5orf46	-0.57761	2.1083	-2.53647	0.013338	0.031458	-3.90148	Down
SGO2	0.742924	3.960867	2.534979	0.0134	0.031588	-3.90533	Up
SPATA31E	-0.50863	4.997133	-2.53465	0.013402	0.031589	-3.90574	Down
RRP12	-0.58144	6.694585	-2.53452	0.013416	0.031612	-3.9064	Down
TSKU-AS1	-0.57648	3.173525	-2.53319	0.013453	0.031687	-3.90916	Down
MTARC1	-0.52093	5.715457	-2.53231	0.013484	0.031749	-3.91122	Down
ITPR2	0.518747	5.850884	2.531391	0.013517	0.031811	-3.91336	Up
CRMP1	0.79628	3.875526	2.531052	0.013541	0.031861	-3.91461	Up
IGFBP6	0.537623	8.700574	2.530329	0.013564	0.031908	-3.9162	Up
SPATA6L	-0.51084	3.589918	-2.52987	0.013571	0.031921	-3.91692	Down
IGKC	0.965715	9.786953	2.530492	0.013578	0.031934	-3.91659	Up
MELK	0.744833	3.535724	2.52795	0.013647	0.032075	-3.92169	Up
CYFIP2	-0.5034	6.052459	-2.52563	0.013722	0.032248	-3.92682	Down
BAMBI	-0.54744	5.063595	-2.52524	0.013736	0.032274	-3.92772	Down
SHOX2	0.560853	5.341723	2.523065	0.013816	0.03244	-3.93286	Up
COL2A1	0.982676	3.962666	2.523136	0.013828	0.032465	-3.93324	Up
CYP1A1	-0.84476	3.356265	-2.52245	0.013846	0.0325	-3.93458	Down
DLK1	0.957827	10.05527	2.522019	0.013881	0.032562	-3.93634	Up
EFCAB7	0.656545	6.682166	2.51715	0.014044	0.032899	-3.94712	Up
TNMD	1.766775	4.943425	2.516702	0.014085	0.032989	-3.94908	Up
HOXC10	-1.30512	8.809188	-2.51398	0.014185	0.03319	-3.9554	Down
WFDC10A	-0.68977	2.492853	-2.5057	0.014453	0.033743	-3.97317	Down
LOC10013	-0.67437	3.892431	-2.50377	0.014533	0.033912	-3.9779	Down
TSSK4	-0.63764	2.738427	-2.49672	0.014794	0.034464	-3.99395	Down
CCDC150	0.642849	3.396539	2.494932	0.014863	0.034613	-3.99808	Up
GPR45	-0.60649	2.261694	-2.49382	0.014906	0.034698	-4.00066	Down
UNC13D	-0.6073	3.132513	-2.49247	0.014958	0.034797	-4.00377	Down
MYL10	-0.53945	8.474637	-2.49242	0.01497	0.03482	-4.00422	Down
CXCL11	0.813868	3.823015	2.488475	0.01513	0.035162	-4.01353	Up
RHOV	-0.65462	3.013986	-2.48707	0.015168	0.035225	-4.01623	Down
GAL3ST4	0.687236	6.168893	2.486343	0.015212	0.035311	-4.01841	Up
PLD5	0.75328	3.688924	2.483936	0.015301	0.035504	-4.02376	Up
COL28A1	0.73781	3.3305	2.480743	0.015425	0.035755	-4.031	Up
IFITM10	0.706139	4.347011	2.479008	0.015497	0.035891	-4.03509	Up
KDM6B	-0.50125	6.609373	-2.47814	0.015525	0.035945	-4.03687	Down
MAP9	0.840587	7.78664	2.476725	0.01561	0.036101	-4.041	Up
MIR4290H	-0.65612	2.238182	-2.4758	0.015616	0.036108	-4.04213	Down
CCKAR	-0.5478	3.483685	-2.4734	0.015713	0.036312	-4.04764	Down
PIP4K2A	0.616856	7.233659	2.47242	0.015769	0.036427	-4.05041	Up
LINC0213!	-0.64314	2.617819	-2.47034	0.015837	0.036571	-4.05465	Down
MILR1	0.771168	3.8689	2.470604	0.015839	0.036571	-4.05442	Up
PRXL2B	0.577079	5.505441	2.468347	0.015924	0.036739	-4.05936	Up
PDE1B	-0.84536	2.537907	-2.4669	0.015982	0.036862	-4.06264	Down
ASCL5	0.60825	3.767321	2.460717	0.016236	0.037399	-4.07671	Up
CRYBA4	-0.63288	2.498325	-2.45985	0.01627	0.037466	-4.07863	Down
PDXP-DT	-0.52715	4.751839	-2.45309	0.016555	0.038064	-4.09403	Down
TNRC6B-I	-0.70006	2.824378	-2.45273	0.01657	0.038091	-4.09487	Down

DOK6	0.793623	3.004819	2.452351	0.016593	0.038136	-4.09594	Up
HCAR1	0.880162	2.689252	2.449816	0.016703	0.038354	-4.10174	Up
NPAP1	0.574979	2.710506	2.446987	0.016816	0.038581	-4.10792	Up
GABRE	0.848694	4.378318	2.446742	0.016843	0.03864	-4.10899	Up
BMP2K	0.812868	6.365294	2.445855	0.016882	0.038708	-4.11101	Up
AOX1	0.713022	5.153898	2.445546	0.016891	0.038724	-4.11157	Up
LOC10537	-0.56029	4.900693	-2.44231	0.01702	0.039	-4.11861	Down
ZNF233	-0.54535	4.285794	-2.44164	0.017047	0.039053	-4.12005	Down
CH25H	0.916817	3.494349	2.441426	0.017074	0.039089	-4.12105	Up
ZCCHC2	-0.53671	7.43887	-2.44	0.017129	0.039192	-4.12408	Down
HMOX1	0.509193	6.220776	2.438289	0.017196	0.039326	-4.12771	Up
TKT	0.555526	7.122259	2.438054	0.017214	0.039364	-4.12848	Up
TRGC2	0.579779	3.853042	2.432234	0.017461	0.039865	-4.14136	Up
A1BG-AS1	-0.64963	2.185498	-2.43218	0.017463	0.039865	-4.14147	Down
CENPE	0.746735	2.558884	2.426521	0.017717	0.040377	-4.15426	Up
LHB	-0.5459	5.235111	-2.42009	0.018011	0.040998	-4.16881	Down
HHLA2	-0.52484	3.244659	-2.41992	0.018017	0.041006	-4.16913	Down
LINC0252	-0.53567	4.388411	-2.41896	0.018061	0.041096	-4.1713	Down
LINC0178	-0.55355	2.621163	-2.41727	0.018139	0.041244	-4.1751	Down
BFSP2	0.603775	3.75142	2.416183	0.018191	0.041355	-4.17761	Up
RARA-AS	0.557418	4.936917	2.412845	0.018347	0.041668	-4.18512	Up
GFRA2	0.538828	4.653953	2.4103	0.018462	0.041912	-4.19075	Up
RASA4	-0.50975	8.483509	-2.41016	0.018479	0.041938	-4.19134	Down
ANKRD20	-0.72739	3.409756	-2.40865	0.018548	0.042068	-4.19468	Down
PCDHB17I	-0.89556	3.367727	-2.40467	0.018745	0.04247	-4.20383	Down
ENO2	-0.53317	4.516026	-2.39737	0.019076	0.043123	-4.21969	Down
SLCO4C1	0.601009	2.980175	2.396955	0.019096	0.043147	-4.22061	Up
CLDN1	0.51692	4.272539	2.396525	0.019116	0.043187	-4.22157	Up
NKAIN2	-0.85826	2.484959	-2.39619	0.019138	0.043231	-4.22246	Down
NEUROG3	-0.60203	2.409033	-2.39469	0.019205	0.043365	-4.22568	Down
DLGAP5	0.750286	2.809263	2.390169	0.019428	0.043818	-4.2358	Up
FMO3	-0.57624	5.361043	-2.38732	0.019573	0.04411	-4.24227	Down
TENT5D	0.551488	2.426114	2.386871	0.019587	0.044136	-4.24308	Up
LMNB1	0.604778	4.447875	2.387003	0.019588	0.044136	-4.24297	Up
RASGRF1	-0.52806	3.678716	-2.38513	0.019674	0.044314	-4.24696	Down
CD33	0.832906	3.805019	2.384078	0.019744	0.044445	-4.24975	Up
SPAG1	0.526843	3.170292	2.37868	0.019995	0.044963	-4.26128	Up
ROR1-AS	0.696777	1.474493	2.377174	0.020071	0.0451	-4.26462	Up
LRP1	0.537285	6.37824	2.374567	0.020211	0.045386	-4.27059	Up
SLITRK4	-0.98166	5.393004	-2.3742	0.020252	0.045449	-4.27192	Down
BNC1	0.514311	3.070814	2.372699	0.020297	0.045537	-4.27453	Up
SLC5A12	0.586124	3.094535	2.370581	0.020406	0.045746	-4.27922	Up
ALDH1B1	-0.55426	6.77969	-2.37058	0.020417	0.045759	-4.27948	Down
LOC10192	0.617665	2.52291	2.370003	0.020435	0.045786	-4.28049	Up
SNORD11	-0.53937	3.406084	-2.36748	0.020565	0.046044	-4.28607	Down
RFX6	0.598367	2.247703	2.365929	0.020645	0.046204	-4.2895	Up
FGFR3	-0.55686	3.67695	-2.36313	0.02079	0.04648	-4.29567	Down
SLC38A5	0.67413	2.976305	2.361904	0.020854	0.046604	-4.29838	Up
C6orf15	-0.50157	5.788858	-2.36159	0.020871	0.046628	-4.29908	Down
L3MBTL4	0.54538	3.282158	2.358552	0.021029	0.046947	-4.30576	Up
AMACR	-0.51279	6.101046	-2.35172	0.021395	0.047704	-4.32088	Down
ARHGAP9	0.537171	6.897771	2.347828	0.021611	0.048149	-4.32958	Up
ABO	-0.63044	5.351745	-2.34616	0.0217	0.048319	-4.33323	Down
COL4A4	0.579661	6.727977	2.345247	0.02175	0.048414	-4.33523	Up
LOC10192	-0.54887	4.343854	-2.3384	0.022114	0.049147	-4.35002	Down
LINC0289	-0.67283	3.033074	-2.33251	0.022438	0.049807	-4.36286	Down
SAMD5	0.65767	3.577998	2.331387	0.022508	0.049929	-4.36546	Up
