# Supplementary material for: Transcriptomics-based analysis of muscle injury mechanism mediated by inflammatory imbalance in the Duchenne muscular dystrophy muscle microenvironment
Source: Front Neurol. 2026 Sep 15;17:1905030. doi: 10.3389/fneur.2026.1905030 (PMC13619489; doi:10.3389/fneur.2026.1905030)
Supplement: Supplementary file 4 [file Data_sheet_5.pdf]

Supplementary Table S5 746 intersecting genes from DMD DEGs, ECM subgroup DEGs, and the

x
FZD10
COL3A1
MYL5
CLCN5
MEGF10
PEG3
GPR137B
GNG2
MYH8
CELF2
GLT8D2
RGL1
DOCK8
CASP4
ERBB3
TENT5A
SRPX
CILP
SLC9A9
SPARC
CHRNA1
COL1A2
MED12L
CTSK
GAS1
NNMT
NPNT
SNAI2
TP53I3
ADAMTS2
PRTFDC1
COL6A3
MEST
JAM3
TMEM45A
CCDC8
MEIS2
LHFPL2
ENPP2
FST
SERINC5
RPL3
DACT1
TPST1
COL1A1
NOTCH2
MS4A4A
NBPF12
XYLT1
RAP2B
SYNE3
NBPF11
IQGAP2
FRMD6
DCLK1
PALM2AKAP2

COL6A6
CDC14B
COLEC12
MCUB
MYH3
GALNT7
TMED3
C3
THBS2
NBPF14
PIK3AP1
COL5A2
HNMT
NBPF1
BEX4
IGSF3
CFHR1
PNMA8A
AMMECR1
SLC2A10
PLPPR4
PDGFRA
CFI
TMEM176B
PTGFRN
PROS1
MS4A7
EMILIN2
COL5A1
CCNG2
DAPK1
CLEC11A
IL10RA
GNB4
TMSB10
CRYBG1
NDN
FAM171B
LRRC17
CFH
PLP1
BCAT1
VCAM1
FBN1
ABI3BP
RNF125
MATN2
HTRA1
CLSTN2
WIPF1
BLNK
CCDC80
GADD45A
TMEFF2
LAMA4
TNNT2
OLFML1
ADCY7

ZWINT
TMEM98
ADA2
CMTM3
TENM1
PEG10
TIMP2
CD163
CMTM7
FAT1
MS4A6A
HCLS1
LPAR1
SGCE
C1R
TRAM2
CLIC1
HDGFL3
FKBP11
LOX
BTG3
NID1
MRC2
C1RL
TIAM1
TNFRSF21
TNFSF13B
MBNL3
C1orf105
TANC2
ZNF521
MMP2
FMO1
PCDH18
PCOLCE
LGMN
RAB8B
SAT1
OGN
CDC25B
FGL2
PIK3CG
MPEG1
CCND2
PENK
AHR
CD14
PVALB
MYD88
CDON
RUNX2
VCAN
HACD4
CSRNP3
LY96
MARCKS
C1S
PDGFD

GPM6B
COL6A1
LAIR1
RGS2
CHN1
MRC1
STAMBPL1
PLAGL1
SPIN1
RNASE6
LAPTM5
TUBA1A
S100A10
LINC01140
TGFBR2
WWTR1
TBC1D2B
FRMD4B
COL14A1
STK26
PTPRC
MARVELD1
PSD3
KCNE3
PDGFRL
ADD3
PLXNB2
ITGB8
RRBP1
CHRDL1
GRK3
SPP1
MMP16
GOLM1
SHTN1
CD1C
NID2
PLA2G2A
CXCL12
CXCR4
PCDHB10
SLC25A43
EPB41L3
CD44
CRISPLD1
PTTG1
SDC2
LAMB1
RBP7
IL17RD
IRF8
MYMX
CMTM6
CD200
MXRA5
PLCE1
IL1R1
SLC16A14

CLIC4
TNFAIP8
DAB2
AHNAK2
IGFBP3
EVI2A
SRGAP1
CEP15
SLC7A7
VSIG4
PAG1
ACKR1
ANKRD44
ALOX5
GPR183
PCDH7
GXYLT2
CLEC10A
CENPU
GPC3
CD84
ACER3
ZFP36L1
KIF2A
PREPL
GPX8
FPR3
DSEL
ATP11C
PODN
HLA-DMB
ASS1
TRERF1
ABRACL
SESN3
NOTCH2NLA
ARMCX4
ATP1B3
VGLL3
LUM
CSF2RB
DMXL2
KDELR3
COL15A1
MAN1A1
IGF1
GPR34
SPIN4
ECM2
MARCHF1
HLA-DPB1
TPPP3
GIMAP2
ELAVL2
SERPING1
ANTXR1
ARL4C
STK17B

TYROBP
SH3BGRL
DDR2
BNC2
ECT2
EBF1
AIF1
PLAUR
VASH2
SEPTIN11
VWA5A
GASK1B
F13A1
ANXA1
ITGB2
ADAP2
GPNMB
FAP
KCTD12
ITGAM
IFI16
CD302
RND3
FAT4
TCEAL9
CTHRC1
NRK
JAM2
ABCA8
HIF1A
MID1
FSTL1
CD109
LSP1P5
MYOF
LIPA
RASSF2
PYGL
CYBRD1
PYCARD
HYMAI
PLSCR1
FCGR2B
RNASE4
NCF4
CSF1R
TIMP1
CASP4LP
SYTL2
CD4
DPY19L3
COL4A1
S100A4
DNM3OS
SLC2A13
ELK3
CDH11
RARRES2

HLA-DRB1
GJA1
PLK2
FCER1A
TSHZ2
IFI30
HLA-DRB5
CD53
TUBB6
CAPN6
EFEMP1
FN1
GZMA
CBX4
APBB1IP
IL33
SOCS2
ST8SIA1
GGTA1
TRIM14
MSR1
HLA-DMA
KCNQ4
SAMSN1
HLA-DRB4
ZNF738
MX2
PLTP
HLA-DRA
RAI14
CTSS
KAZN
ISLR
TWIST1
KYNU
L1CAM
CLMP
LGALS3BP
ASPN
ITGBL1
ZEB2
C1QA
ZNF177
CAPG
TMEM200A
DOCK11
C1QTNF2
OSMR
APOBEC3G
BCHE
NT5E
RAB31
IFI44
TDO2
FCER1G
LACC1
IL32
CMAHP

CKAP2
AJUBA
SULF2
FCGR2A
CLEC4A
ADRA2A
CPXM1
PLSCR4
SEC24D
MMD
DPYD
SERPINE2
OLFML2B
MAL
EMB
PCDHB14
LINC00597
FABP4
C3AR1
BASP1
PPIB
ARHGAP36
LFNG
ATP8B4
UGCG
IGSF10
LINC02802
S100A2
B3GALNT1
HRH1
ADAMTS12
SPON1
CASP8
TSPAN12
ZNF217
CARD16
ANLN
MGP
CHST6
HS6ST2
GPC6
C1QB
TUSC3
ELN
SLC16A2
LOXL2
TLR1
HLA-DQA1
COL12A1
COTL1
RASA2
KCNT2
PLAU
BNIP3L
OLFML2A
ADAMTSL1
HIP1
TYMS

FOLR2
EEIG2
CLEC7A
THSD7A
SLC30A7
HLA-DPA1
SOX4
ZC3HAV1L
ERRFI1
DPP4
SNORD114-3
GINS1
THY1
TMEM119
PROCR
NIPSNAP3A
NAV3
LCP2
LYZ
CYS1
CCR2
AQP3
PIEZO1
IL17B
MAP1LC3C
FYB1
KLHL2
COL21A1
POGLUT2
MEG3
FBLN1
EVI2B
MT1M
CD300A
LHFPL6
PRPS2
LYN
FGF2
SOX11
CYTOR
PRR5
IFI44L
MFAP4
TUBB2A
C1QTNF3
CEROX1
NCF2
MYCL
MAGED4B
EMP1
TMEM123
TMEM176A
CD24
CCR5
SGMS2
DLGAP1-AS1
ITM2A
KLF5

RRM2
WASF1
SOX8
SOHLH2
UHRF1
ANPEP
C16orf54
FAM20A
LMF1
RUNX1-IT1
LINC02397
SNX7
SOCS3
NAALAD2
ALDH1L2
DKK2
PHLDB2
P2RX7
CXCL14
CSTA
CFB
EPHA3
DOCK10
STMN2
CMKLR1
THEMIS2
C1QC
ZNF83
NES
CCL2
CDKN1A
CPA3
VAV3
S100A11
SFRP4
XG
RABGAP1L
XRCC4
MTHFD1L
LINC02984
LRRN3
PSAT1
ST8SIA4
MFAP2
ELOVL5
TRAF5
BHLHE22
PHLDA1
LY75
RASSF4
PTPRF
GLIPR1
TOP6BL
GLIS2
HSPA6
SLA
NTRK2
SERTAD4

CXCL16
POSTN
FBXL13
PTX3
FGF18
NAP1L3
NEGR1
MIR503HG
CYSLTR1
SERPINA3
DOCK2
DKK3
CCL18
SHD
MEG9
SERPINB9P1
GBP3
FNDC1
CCL8
CFHR2
P3H4
CD74
PCDHB16
SERPINB9
GAS2L3
MYL4
F2R
RUBCNL
LRRC70
IGF2
CNN3-DT
LVRN
LRTM1
HMGCLL1
MCTP1
ADAM28
NPL
OLFML3
ACTC1
CYP26B1
SECTM1
PLA2G4A
LILRB2
FRZB
ACP5
GPR65
FREM1
ADAM12
BGN
LZTS1
CHODL
PPIP5K2
LILRB5
PRKAR2B
ADIRF
NABP1
PERP
HMCN1

PRRG3
P3H2
SERPINH1
SNX10
C7
PCDHB15
NHLH2
CDC7
GALNT17
THBS4
DMRT3
THBS1
SLC16A4
HEPH
PTN
EZH2
C4A
CCDC102B
TBC1D32
ID2
CCDC71L
HAVCR2
RBM47
WNT5B
OMD
BMP5
PRND
DPEP2
DMBT1
TCEAL7
RPS4Y1
CD37
ERAP2
TLR8
C2
RGS1
ZNF556
FBLIM1
TRIM59
EDN3
CMA1
CLU
CARMIL1
MYH4
CA13
SERPINI1
KCNE5
DIRAS3
MDK
TRIM9
FAM72C
TPSAB1
C5AR1
CD8A
SFRP2
AOC3
DDX3Y
PSPHP1

TPSB2
PTGFR
DENND2D
FCGR1BP
SLITRK5
EIF1AY
USP9Y
LINC02937
LTK
OR2A20P
TFEC
MAGEL2
NRCAM
DCX
PTGER3
TMSB15B-AS1
PBK
MYBPH
RGS18
ADIPOQ
EGR3
KDM5D
CHI3L1
TNC
KERA
TRAF4
SLC2A3
HTN1
B3GALT2
S100B
FGFBP2
TMEM132C
AEBP1
CNTN3
BUB1B
CCL3
BEX2
EGR2
MMRN1
SCG5
KRT222
IL7
ROBO3
ITGA4
KIF20A
RBP4
TOP2A
PCK1
ST8SIA2
SCD
PAX8-AS1
TNMD

e WGCNA MEturquoise module.
